# The relationship between ECG predictors of cardiac resynchronization therapy benefit

**DOI:** 10.1371/journal.pone.0217097

**Published:** 2019-05-31

**Authors:** Josef Halamek, Pavel Leinveber, Ivo Viscor, Radovan Smisek, Filip Plesinger, Vlastimil Vondra, Jolana Lipoldova, Magdalena Matejkova, Pavel Jurak

**Affiliations:** 1 Institute of Scientific Instruments of the Czech Academy of Sciences, Brno, Czech Republic; 2 International Clinical Research Center, St. Anne’s University Hospital, Brno, Czech Republic; University Hospital *Paolo Giaccone*, ITALY

## Abstract

**Introduction:**

Cardiac resynchronization therapy (CRT) is an effective treatment that reduces mortality and improves cardiac function in patients with left bundle branch block (LBBB). However, about 30% of patients passing the current criteria do not benefit or benefit only a little from CRT. Three predictors of benefit based on different ECG properties were compared: 1) “strict” left bundle branch block classification (SLBBB); 2) QRS area; 3) ventricular electrical delay (VED) which defines the septal-lateral conduction delay. These predictors have never been analyzed concurrently. We analyzed the relationship between them on a subset of 602 records from the MADIT-CRT trial.

**Methods & results:**

SLBBB classification was performed by two experts; QRS area and VED were computed fully automatically. High-frequency QRS (HFQRS) maps were used to inspect conduction abnormalities. The correlation between SLBBB and other predictors was R = 0.613, 0.523 and 0.390 for VED, QRS area in Z lead, and QRS duration, respectively. Scatter plots were used to pick up disagreement between the predictors. The majority of SLBBB subjects– 295 of 330 (89%)–are supposed to respond positively to CRT according to the VED and QRS area, though 93 of 272 (34%) non-SLBBB should also benefit from CRT according to the VED and QRS area.

**Conclusion:**

SLBBB classification is limited by the proper setting of cut-off values. In addition, it is too “strict” and excludes patients that may benefit from CRT therapy. QRS area and VED are clearly defined parameters. They may be used to optimize biventricular stimulation. Detailed analysis of conduction irregularities with CRT optimization should be based on HFQRS maps.

## Introduction

Cardiac resynchronization therapy (CRT) is an effective treatment that reduces mortality and hospitalization and improves cardiac function in patients with left bundle branch block (LBBB) [1]. However, about 30% of patients passing the current clinical guidelines do not benefit or benefit only a little from CRT [2]. The prediction of CRT outcome based on the ECG signal is an important task. The criteria for strict LBBB (SLBBB) based on QRS shape and QRS duration (QRSd) have been previously proposed [3, 4] to identify patients with complete LBBB to benefit most from CRT. Two other parameters based on different ECG properties—QRS area [2, 5, 6, 7] and the analysis of the high-frequency QRS complex [8, 9]–have been recently suggested as CRT response predictors. The ability to predict CRT response was confirmed for all three predictors [4, 5, 6, 9, 10]. A relationship between these parameters is anticipated, but has not been assessed yet, mainly because the corresponding data was not available.

The classification of SLBBB depends on expert annotation, and disagreement between expert annotations may endanger the diagnostic validity. High-frequency QRS analysis requires a sufficient frequency and dynamic range of the ECG signal. To analyze the relationship between ECG CRT predictors, we used data from the Initiative for the Automated Detection of Strict Left Bundle Branch Block [4]. Our aim was to analyze the relationship between the predictors, and to assess their mutual contribution to patient selection for CRT.

## Materials and methods

### Data

Data and annotation were received from the Telemetric and Holter ECG Warehouse (THEW) within the Initiative for the Automated Detection of Strict Left Bundle Branch Block [4, 11]. Data containing 602 records came from the MADIT-CRT database [12]. Each record included 12-lead ECG signals, a 10-second strip and median beat with a sampling frequency of 1 kHz and amplitude resolution of 3.75 μV. The data was annotated by 2 experts from the Initiative [4], and information about the onset and end of the QRS complex, QRSd, occurrence of notch and slur in different leads and SLBBB criteria fulfillment was provided. 330 SLBBB patients and 272 non-SLBBB patients were identified in the whole dataset. 193 subjects with different forms of left ventricular conduction delay, such as left ventricular hypertrophy or left anterior fascicular block, that did not satisfy “strict” LBBB criteria and 79 other non-specified subjects were present in the non-SLBBB group [4].

### Strict LBBB classification

The strict LBBB criteria have been suggested to better select patients for CRT, as patients without complete LBBB benefit only a little from CRT [3, 4, 13). Evaluation was based on experimental and clinical observation together with computer simulation. These criteria include QRS duration ≥ 140 ms (men) or ≥ 130 ms (women), QS or rS in leads V1 and V2, and mid-QRS notching or slurring in ≥ 2 of leads V1, V2, V5, V6, I and aVL [13]. At the beginning of the Initiative, a slur or notch was required to begin after the first 40 ms and before 50% of the QRS duration, and had to end before 2/3 of the QRS duration [11, 13]. After the end of Initiative [4] the input definition was changed to “not to require the mid-QRS notch/slur to end before two-thirds of the QRS duration”. In our analysis we used this final definition and corresponding binary annotation: subjects that satisfied “strict” LBBB criteria (SLBBB) and subjects that did not satisfy all “strict” LBBB criteria (non-SLBBB). This annotation was given by two experts and we accept it as valid.

### QRS area

QRS area has been shown to be a promising electrophysiological predictor of CRT response [2, 5, 6, 7]. The provided median beat was used in the analysis of QRS area together with annotation of the onset and end of the QRS complex. Orthogonal signals were given by Dower transformation [14] of 12-lead signals. Areas in single leads (Xarea, Yarea, Zarea) were defined as the area between the baseline and the signal of the corresponding lead from the onset to the end of the QRS complex. QRSarea [5] was defined as:

$$QRSarea=\sqrt{{Xarea}^{2}+{Yarea}^{2}+{Zarea}^{2}}$$

Four parameters (Xarea, Yarea, Zarea, QRSarea) were analyzed and the parameter that best correlates with SLBBB classification was used in final analysis.

### High-frequency QRS analysis

**Fig 1:**
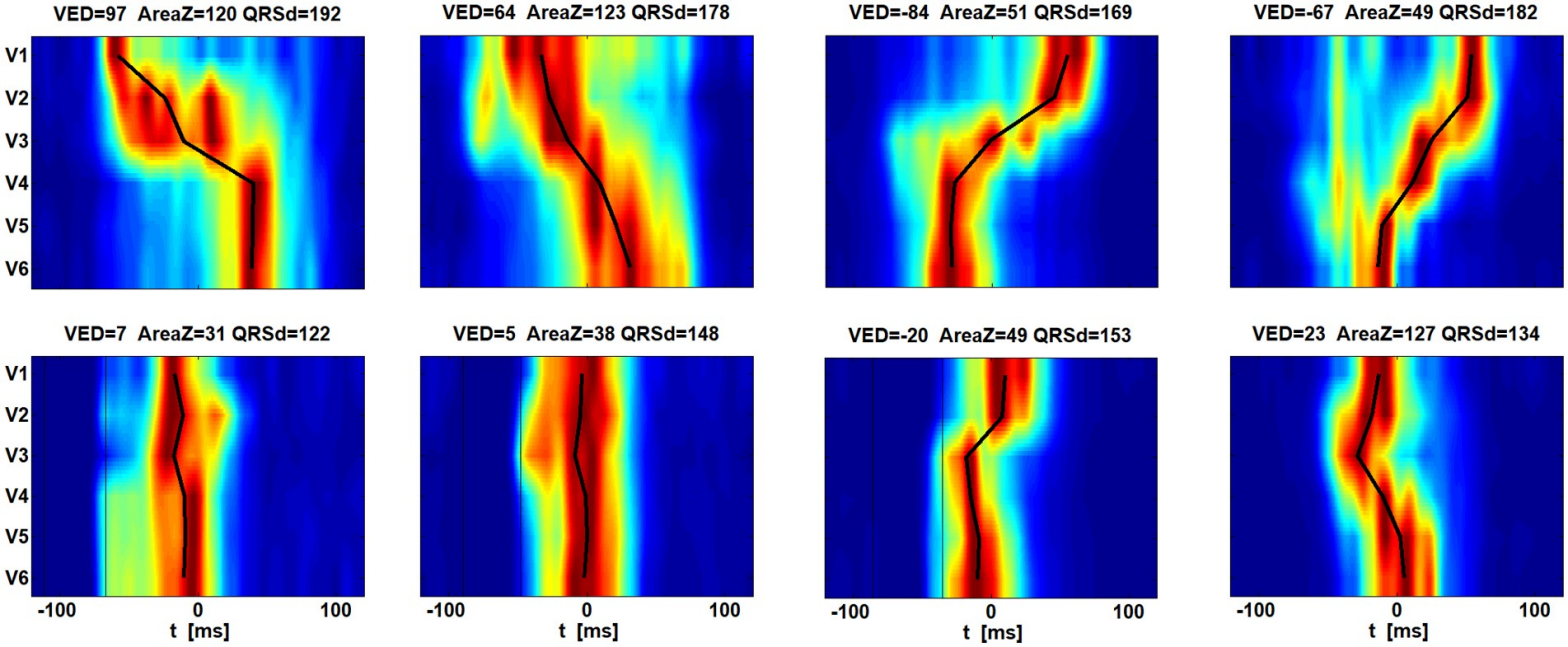
Example of HFQRS maps to demonstrate their contribution in dyssynchrony analysis. The data were measured at St. Anne’s University Hospital Brno. Two LBBB subjects (left two columns) and two RBBB subjects (right two columns) are presented. The measurement before implantation is shown in the first line, the measurement after implantation with optimized pacing is shown in the second line. Leads V1, V2 describe, above all, the activation of the septum and right ventricle, leads V5, V6 activation of the left ventricle lateral wall. Black lines connect points with maximal activity in corresponding leads.

High-frequency QRS (HFQRS) signals describe the time-spatial distribution of fast changes in depolarization (Phase 0 of action potentials). The theory, analysis and possible contribution to CRT have been described by Jurak et al. [8]. The value of ventricular electrical delay (VED), which represents the time distribution of HFQRS, can predict the benefit of CRT in LBBB patients [9]. HFQRS maps [8], as another output of the HFQRS analysis, offer information about the depolarization pattern of ventricles. In our analysis we used both—VED as a parameter that is compared with the SLBBB classification and QRS area, and HFQRS maps to evaluate conduction irregularities. An example of HFQRS maps and their contribution in dyssynchrony analysis is demonstrated in Fig 1. Data were measured at St. Anne’s University Hospital Brno. Two LBBB subjects (left two columns) and two RBBB subjects (right two columns) are presented. The measurement before implantation is shown in the first line, measurement after implantation with optimized pacing in the second line. Leads V1, V2 describe above all the activation of the septum and right ventricle (RV), leads V5, V6 the activation of the left ventricle (LV) lateral wall. The difference between activation in leads V1, 2 and V5, 6 define the dyssynchrony—the parameter VED.

Ventricular electrical delay (VED) [9], as the level of electrical dyssynchrony, was analyzed in the provided 10-sec strip. As the first step, QRS complexes were detected [15] and amplitude passband envelopes in three frequency bands (100–200; 150–250; 200–300 Hz) were computed using Hilbert transformation. After this, QRS envelopes were averaged according to the R-wave trigger. Averaged QRS envelopes in chest leads (V leads) define the time-spatial distribution of electrical myocardial activation [8]. VED was computed as follows: in every V lead the time that best defines the electrical activation was computed. This time was defined as the center of mass of the signal in the corresponding lead, neglecting signals below 0.5 of the maximal amplitude of the signal. The resulting delay was defined as the maximal delay between lead V1 or V2 versus lead V5 or V6 (VED_12-56_), and also as the maximal delay between all V leads (VED_ALL_). The analyzed delays are partly dependent on the frequency band used. For this reason, we also used the averaged normalized HFQRS envelopes over the three mentioned frequency bands and define delays in these averaged HFQRS envelopes. The mean and median VED was computed from all 8 VEDxx delays. The relatively large difference between the mean and median value is a marker of the low signal-to-noise ratio or some irregularity in activation that cannot be described by a simple numerical parameter such as VED. The median value of VED was used in further analysis. The HFQRS maps, as another output from the HFQRS analysis, represent the time-spatial distribution of the electrical activation of ventricles. The presented maps were created from normalized averaged HFQRS envelopes. Leads V1, V2 mainly reflect the electrical activation of the RV lateral wall and septum, while leads V5, V6 predominantly describe the activation of the LV lateral wall. The different pattern of HFQRS maps better describes the irregularities of myocardial activation than a simple numerical parameter such as VED, and may serve for more-detailed analysis of ventricular electrical depolarization.

## Results

The analyzed ECG data are accessible from the THEW database [16]. A table with all the presented numerical results together with important results from the Initiative is given in S1 Table. The linear Pearson correlation coefficient R between SLBBB classification and other predictors was R = 0.613, 0.532, 0.411 and 0.391 for VED, Zarea, QRSarea and QRSd, respectively. The correlation between VED and others was R = 0.633, 0.473 and 0.154 for Zarea, QRSarea and QRSd, respectively. All correlations are highly statistically significant. The mean levels of parameters for the SLBBB and non-SLBBB groups are presented in Table 1. These parameters are statistically significantly different, P<10^-10.

**Table 1:** Mean level ± STD of parameters in the groups SLBBB and non-SLBBB; all parameters are statistically significantly different between groups, P<10^-10.

	QRSd [ms]	VED [ms]	Zarea [ms*mV]	QRSarea [ms*mV]
SLBBB	160±17	70±25	127±43	150±45
non-SLBBB	143±21	17±43	71±46	107±48

**Fig 2:**
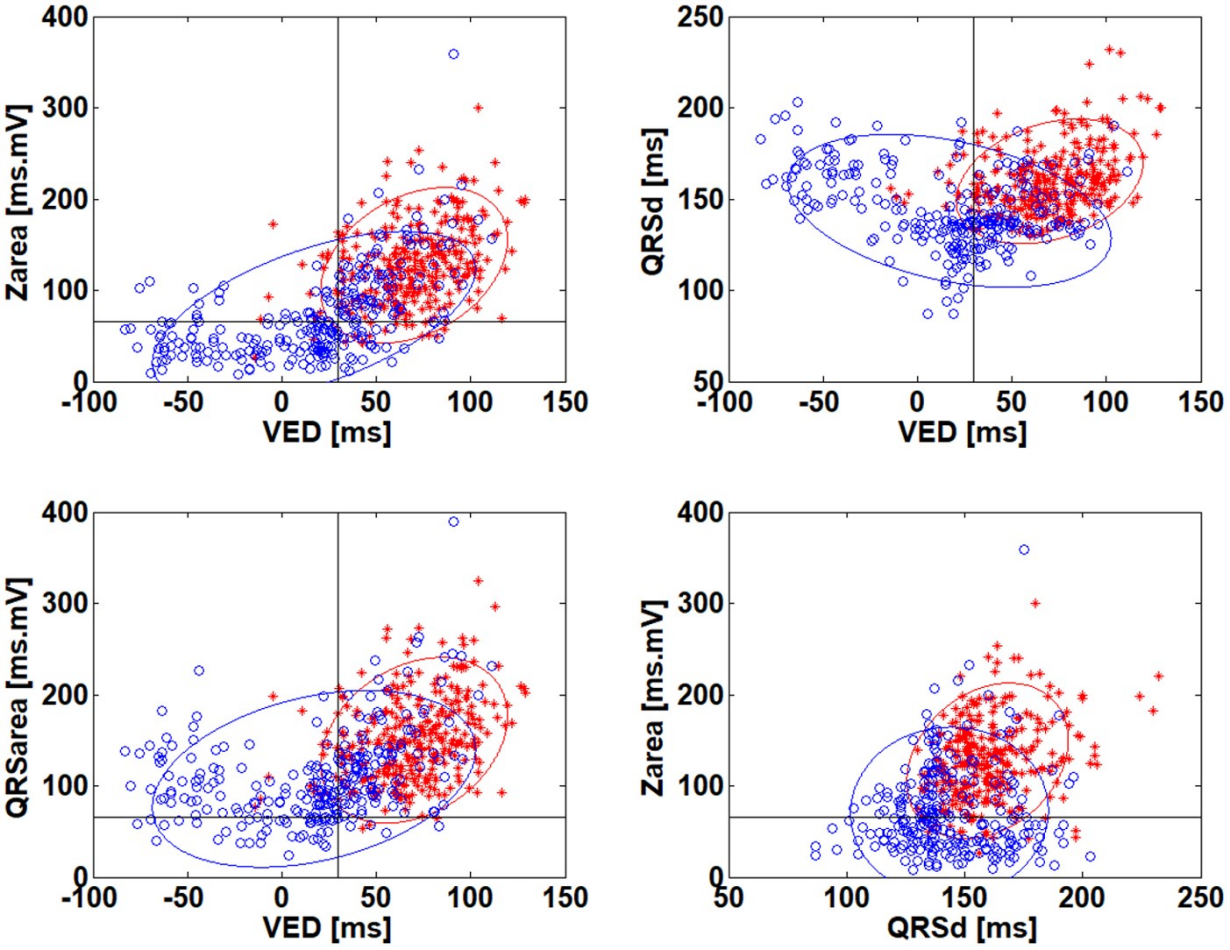
Red ‘*’ SLBBB subjects, blue ‘o’ non-SLBBB subjects. The lines (VED = 30 ms; Zarea = 66 ms*uV) represent published limits used to differentiate CRT responders according to the corresponding parameter. Ellipses express 95% confidence intervals.

**Fig 3:**
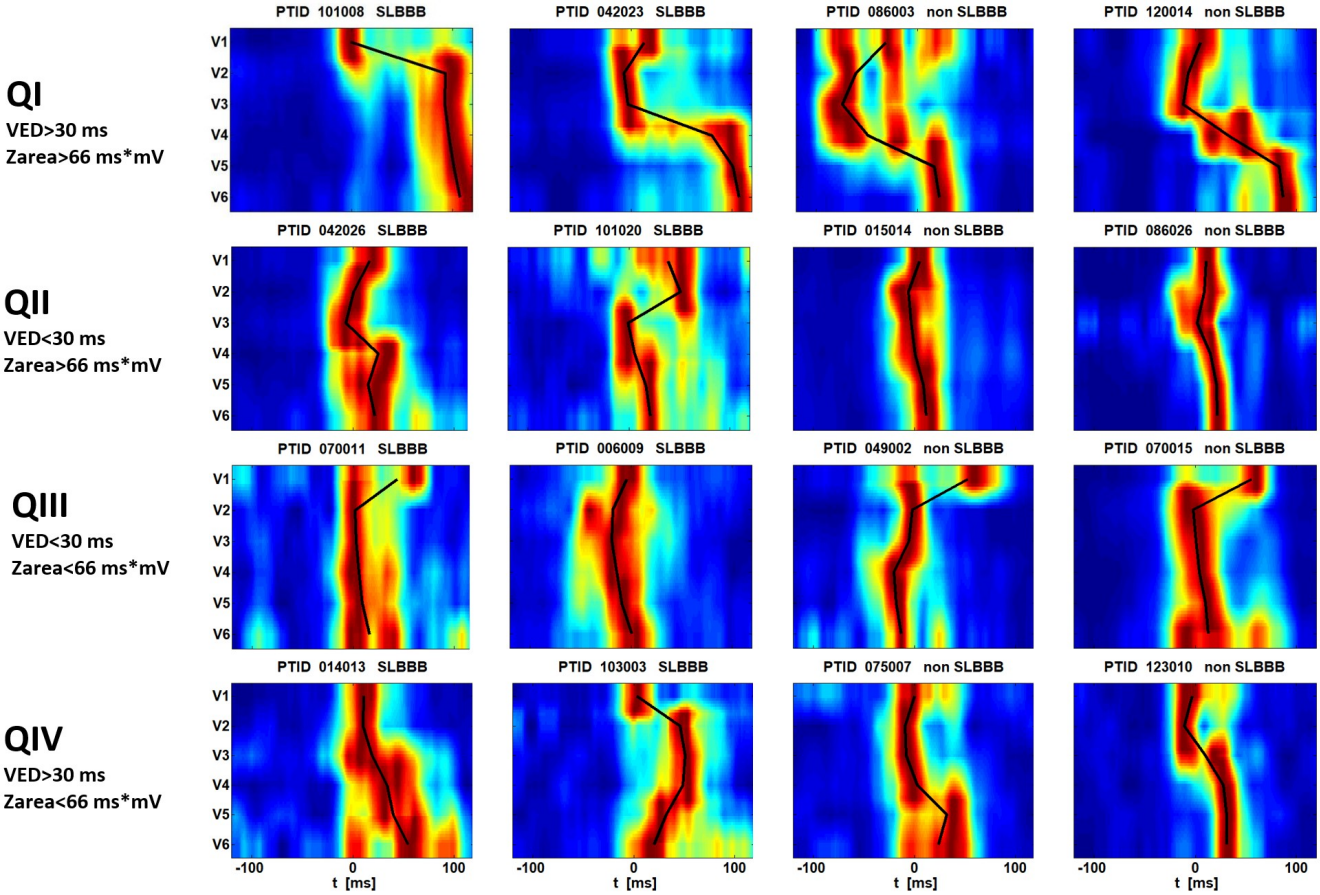
HFQRS maps of randomly selected subjects with parameters in Table 2. In each quadrant two SLBBB subjects and two non-SLBBB subjects were selected. HFQRS maps define the time-spatial distribution of electrical activation. Leads V1, V2 mainly describe the electrical activation of the RV lateral wall and septum, leads V5, V6 mainly describe the activation of the LV lateral wall. The black lines connected the centers of activation in leads.

Scatter plots to analyze the distribution of parameters for SLBBB and non-SLBBB subjects are given in Fig 2. Red ‘*’ are SLBBB subjects, blue ‘o’ are non-SLBBB subjects. The lines at VED = 30 ms and Zarea = 66 ms*mV represent previously published limits [6, 9] used to differentiate CRT responders. Ellipses express 95% confidence intervals for SLBBB and non-SLBBB. Both clusters, SLBBB and non-SLBBB, are significantly overlapped. We further focused on the scatter plot between VED and Zarea as these two parameters have a maximal correlation with SLBBB classification. The limit lines (VED = 30 ms, Zarea = 66 mV*ms) define four quadrants. The majority of SLBBB subjects (295 subjects, 89%) lie in the first quadrant (VED>30 ms, Zarea>66 mV*ms), but there is also a significant number of non-SLBBB subjects (93 subjects, 34%). Two SLBBB subjects and two non-SLBBB subjects were randomly selected in each quadrant. The corresponding levels of parameters for these subjects are shown in Table 2 and the corresponding HFQRS maps are presented in Fig 3. All HFQRS maps and parameters for all analyzed ECG are given in S1 Fig and S1 Table. The patients’ ID (PTID) is used to define the analyzed ECG file.

**Table 2:** Randomly selected subjects with HFQRS maps in Fig 2. In each quadrant two SLBBB subjects and two non-SLBBB subjects were selected.

PTID	SLBBB	QRSd [ms]	VED [ms]	Zarea [ms*mV]	QRSarea [ms*mV]
101008	1	180	104	300	324
42023	1	173	113	240	296
86003	0	175	91	358	390
12014	0	147	95	215	243
42026	1	146	15	96	100
101020	1	148	-7	93	110
15014	0	135	18	98	120
86026	0	138	18	127	170
70011	1	156	-14	26	76
6009	1	159	17	45	94
49002	0	162	-69	10	63
70015	0	192	-43	37	113
14013	1	149	46	50	57
103003	1	197	42	44	122
75007	0	128	42	54	73
123010	0	128	42	50	69

## Discussion

Three predictors of CRT response, based on different properties of the ECG signal, were compared. A significant correlation exists between them. This correlation is partly due to a predictor’s dependence on QRSd, but is given, first and foremost, by the dependence on conduction irregularities. The majority of SLBBB subjects (93%) fall in the 1^st^ quadrant of the scatter plot (Fig 2) and these subjects will benefit from CRT therapy according to QRS area, VED and HFQRS maps. However, 34% of non-SLBBB subjects also fall in the 1^st^ quadrant and these subjects should benefit too. Therefore, SLBBB classification may be used to confirm decisions about CRT therapy, but should not be used to exclude patients. According to Caputo [17], the simplest criteria to define LBBB provided the best association with clinical endpoints in CRT. Moreover, a false positive classification exists in SLBBB subjects 70011 in quadrant III and 101020 in quadrant II. Their HFQRS maps express a rather RBBB pattern, and special attention should be paid to the biventricular stimulator settings if ever implanted. The proper setting of cut-off values (QRSd, definitions of notches and slurs), as well as the debate about the best definition of SLBBB based on QRS shape, remains wide open [17, 18, 19, 20]. SLBBB classification is a binary parameter defined from multiple binary sub-parameters such as QRSd and the occurrence of notches and slurs. Any erroneous determination in sub-parameters may reverse the classification. This significantly limits the diagnostic contribution of SLBBB classification.

Both Zarea and VED are clearly defined, and their assessment can be fully automatic. Zarea combines QRS duration and the electrical force of ventricular activation [5, 6, 7], and improves CRT benefit prediction. According to Maas et al. [21], QRS area is a strong predictor of CRT benefit. According to our results, the maximal correlation is between VED and Zarea, R = 0.633. The possible limitation of QRS area may be that it does not differentiate between RBBB and LBBB, and that the QRS area is inversely associated with focal scar, indicating that myocardial scar leads to a smaller QRS area [6].

VED assesses the time difference of electrical activation of the left ventricle relative to the right ventricle and septum in milliseconds, i.e. the electrical dyssynchrony that can be corrected by CRT therapy. VED is a good predictor of CRT benefit according to the Plesinger study [9] on the MADIT-CRT trial limited to LBBB patients only. We used a modified algorithm for VED computing in this study since only 10-sec strips were analyzed and the maximal analyzed frequency was 300 Hz. Only VED measures electrical dyssynchrony directly in milliseconds. The main target of CRT treatment is the correction of the baseline electrical ventricular dyssynchrony [22, 23], and we can assume that the benefit of CRT will correspond to the level of baseline electrical dyssynchrony, i.e. to the absolute level of VED. Inaccurate assessment of electrical dyssynchrony based on indirect parameters such as prolonged QRS interval and the shape of QRS may partially stay behind a large number of non-responders in CRT.

VED and QRS area are promising parameters, but simple numerical parameters cannot define all the details in conduction irregularities. Selected subjects in quadrant IV represent an example. The values of VED and Zarea are similar in these subjects, but the HFQRS map of subject 103003 is significantly different from the others. The patterns of HFQRS maps simply indicate different types and details of ventricular conduction disturbances. HFQRS maps may be used to assess the optimal position of leads, and may partially replace echocardiographic mapping [8]. Corresponding analyses, based on the diagnosis of patients, are needed in the future.

QRS area, VED and, above all, HFQRS maps may be used not only to predict CRT response, but also to optimize biventricular pacing [7, 8]. A significant contribution may be expected in patients with short QRS and RBBB. According to Auricchio [24], several factors strongly suggest that CRT is delivered in the wrong way in hearts with RBBB. Patients with poor RV function may also benefit from CRT, and this is an unstudied area.

HFQRS analysis is promising, simple, cheap and easy to perform. The only requirement is the higher quality of ECG; this means a minimal sampling frequency of 1 kHz, a frequency bandwidth of at least 300 Hz, and a dynamic range of at least 16 bit. Such requirements should not represent a problem in the present day, and can be satisfied even by good Holter monitors. As the averaging of QRS complexes is used, recordings should be longer to obtain a larger number of complexes for analysis.

The optimal selection of patients for CRT is an important task for the clinical community involved in CRT. CRT non-response may have an adverse effect on cardiac function [24]. The definition of proper predictors and the analysis of their contribution represent a present challenge. The QRS narrowing index (QI) [25] and QRS duration normalized to LV end-diastolic volume [26] have been recently introduced and are potentially promising parameters. Other suggested parameter is the interventricular conduction delay [27–29] or the multi-parametric models, based on different properties including biomarkers [30]. The definition of the best CRT predictor or the best model is still an open question. The result is limited by access to corresponding data. According to review in Cardiac Electrophysiology [31], the promising predictors defined in the 2018 year are the QRS duration normalized to LV end-diastolic volume [26]; VED [9]; and interventricular delay [30]. The different imaging methods such as tissue Doppler imaging, 2D speckle echocardiography, cardiac computed tomography (CCT), single positron emission computed tomography (SPECT), and cardiac magnetic resonance (CMR) are being extensively studied in relation to CRT, but are still far from wide use due to their various limitations [32].

The clinical response to CRT depends not only on patient’s selection but also on other factors, such as the characteristics of the substrate, the site of insertion of the LV-lead and its relationship with the site of maximum intraventricular delay, and the progression of the underlying disease. Without any doubt, a right patient’s selection is the most crucial step in CRT. VED, QRS area, QI, and HFQRS maps may also be used to optimize biventricular pacing.

### Limitations

Only the relationship between predictors was analyzed, as no outcome to the CRT therapy was available. Unfortunately, we were not successful with a request for the blind analysis of our results with CRT outcome. All our results are in S1 Table and S1 Fig and we hope that the analysis of outcome, as a reaction to our manuscript, will be possible in future.

## Conclusion

The relationship between three ECG predictors of CRT response, based on different properties of the ECG signal, was analyzed. A significant correlation exists between them. The lack of a clear definition of cut-off values for SLBBB definition and binary classification is a significant limitation of the SLBBB parameter. SLBBB confirms the diagnosis, but should not be used to exclude patients from CRT. A significant proportion of non-SLBBBs are potential responders according to VED, Zarea and HFQRS maps. Zarea and VED are clearly defined, can be analyzed fully automatically and can be used not only for patient selection, but also for optimization of biventricular stimulation. HFQRS maps describe details of ventricular conduction irregularities that cannot be fully described by VED or QRS area. The optimal selection of patients for CRT and the optimal setting of stimulation should be based on HFQRS maps, as no numerical parameter may describe the details in conduction irregularities.

## Supporting information

S1 Table Numerical results for all subjects. (XLS)

S1 Fig HFQRS maps for all subjects. (PDF)
