# Supplementary material for: The relationship between ECG predictors of cardiac resynchronization therapy benefit
Source: PLoS One. 2019 May 31;14(5):e0217097. doi: 10.1371/journal.pone.0217097 (PMC6544221; doi:10.1371/journal.pone.0217097)
Supplement: S1 Fig — (PDF) [file pone.0217097.s002.pdf]

HFQRS maps of all subjects with parameters in Comparison_SLBBB_VED_QRSArea.xls. HFQRS maps define the time-spatial distribution of electrical activation. Leads V1,2 mainly describe the electrical activation of the RV lateral wall and septum, leads V5,6 mainly describe the activation of the LV lateral wall. The black lines connected centers of activation in leads.

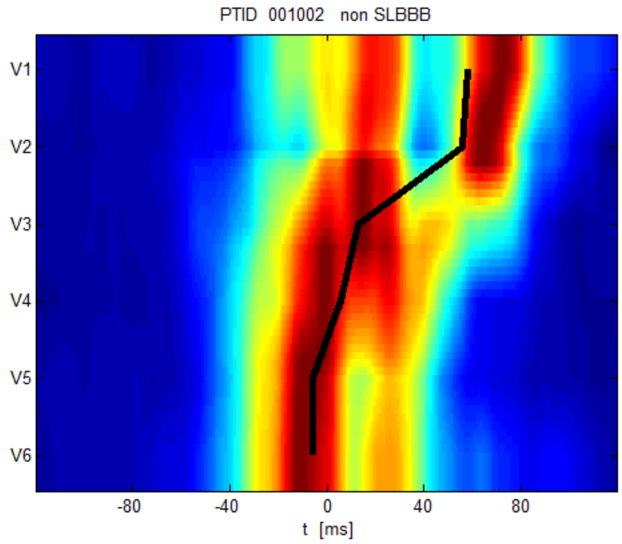

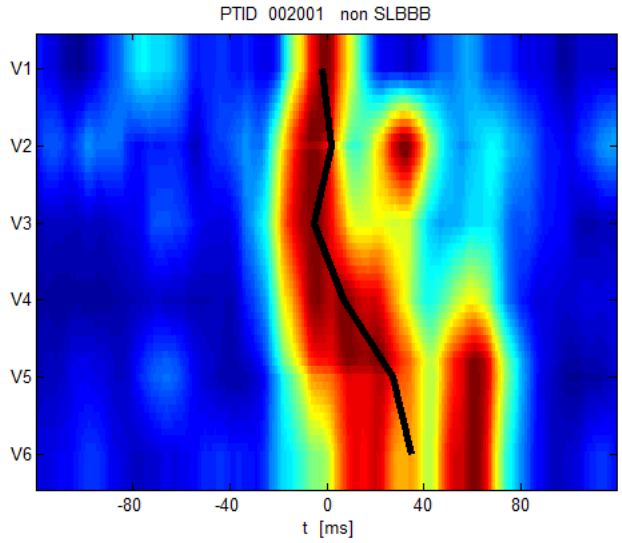

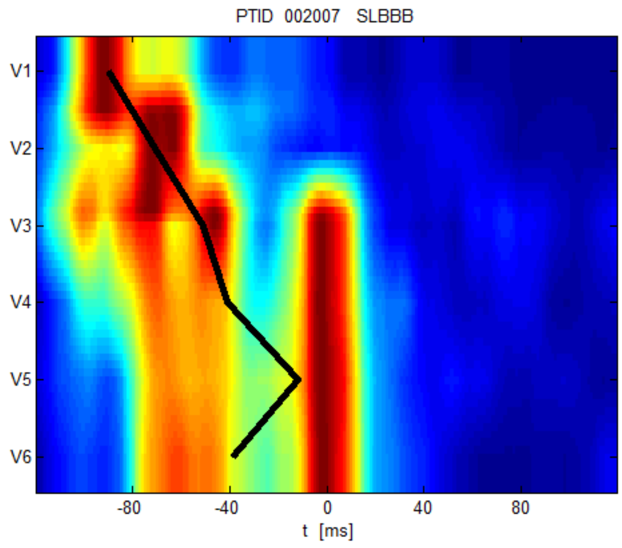
PTID 002007 SLBBB
V1
V2
V3
V4
V5
V6
-80
-40
0
40
80
t [ms]

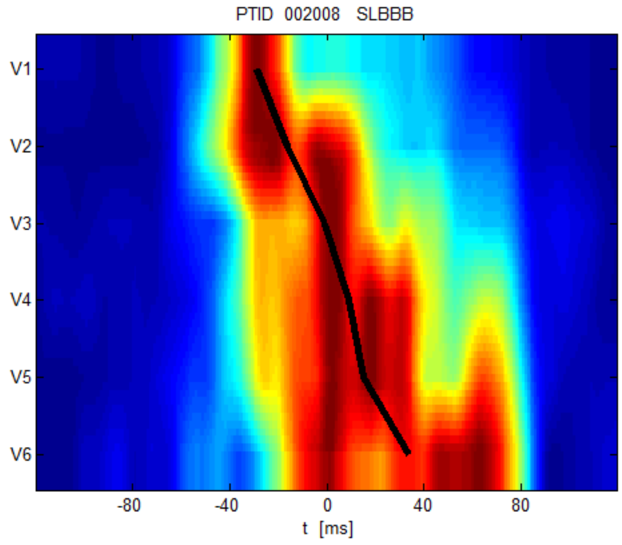
PTID 002008 SLBBB
V1
V2
V3
V4
V5
V6
-80
-40
0
40
80
t [ms]

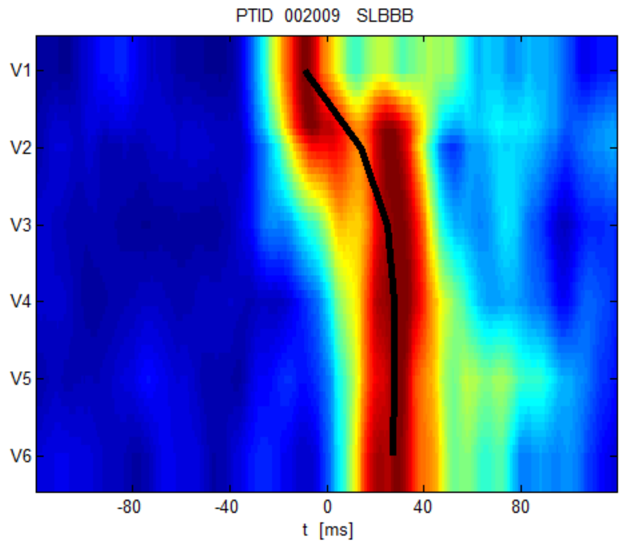
PTID 002009 SLBBB
V1
V2
V3
V4
V5
V6
-80
-40
0
40
80
t [ms]

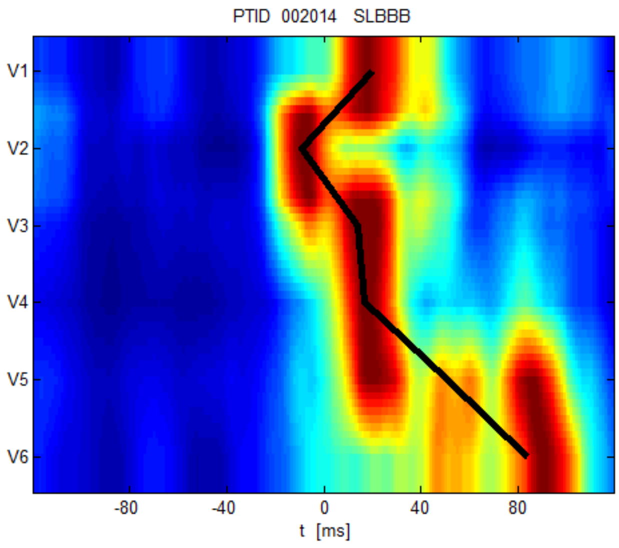

PTID 002014 SLBBB
V1
V2
V3
V4
V5
V6
-80
-40
0
40
80
t [ms]

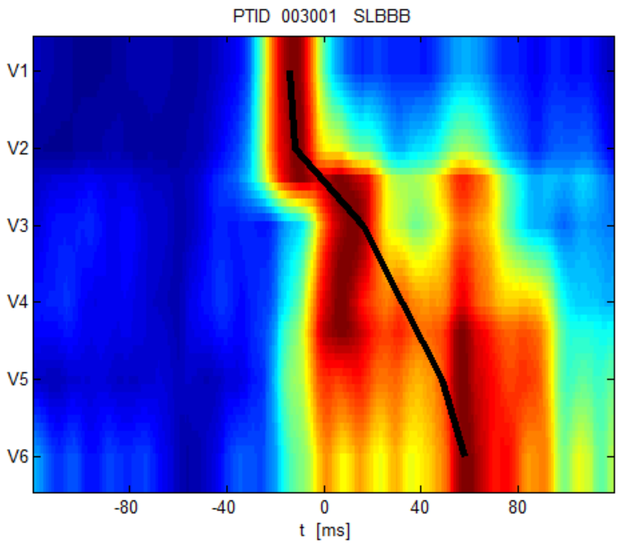

PTID 003001 SLBBB
V1
V2
V3
V4
V5
V6
-80
-40
0
40
80
t [ms]

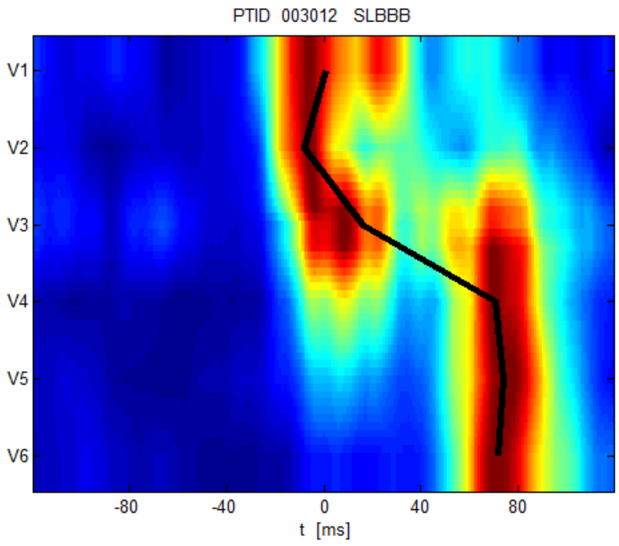

PTID 003012 SLBBB
V1
V2
V3
V4
V5
V6
-80
-40
0
40
80
t [ms]

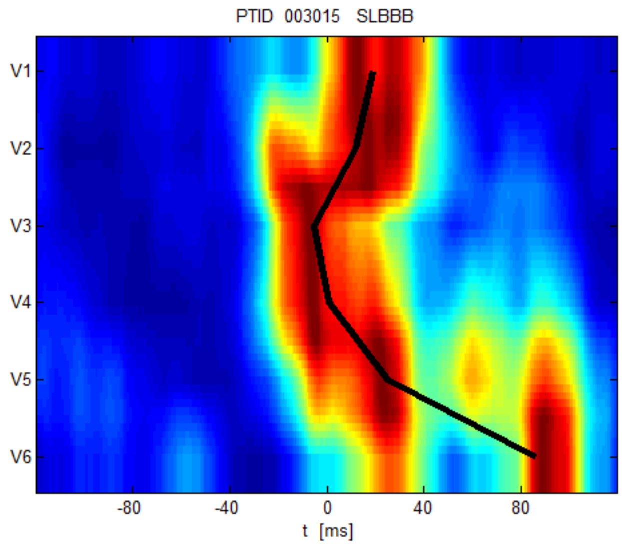
PTID 003015 SLBBB
V1
V2
V3
V4
V5
V6
-80
-40
0
40
80
t [ms]

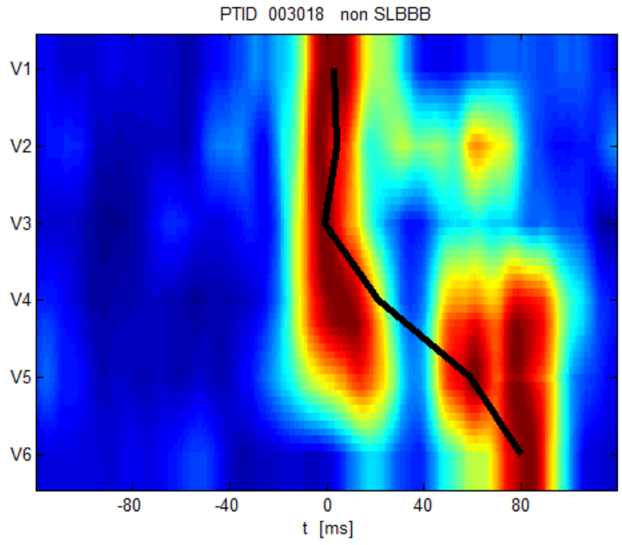
PTID 003018 non SLBBB
V1
V2
V3
V4
V5
V6
-80
-40
0
40
80
t [ms]

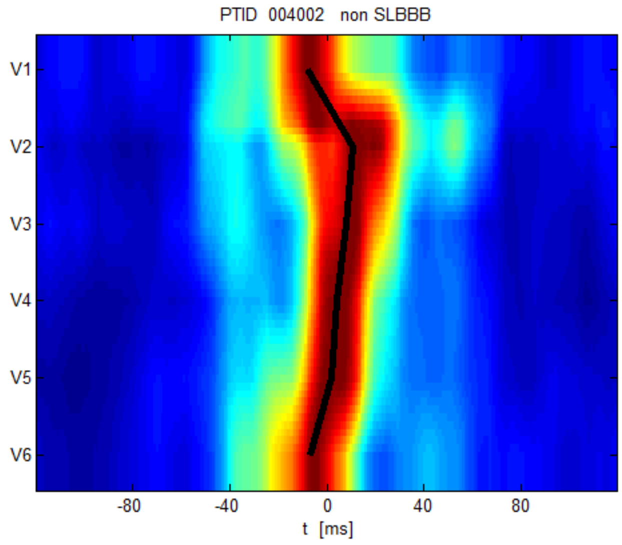
PTID 004002 non SLBBB
V1
V2
V3
V4
V5
V6
-80
-40
0
40
80
t [ms]

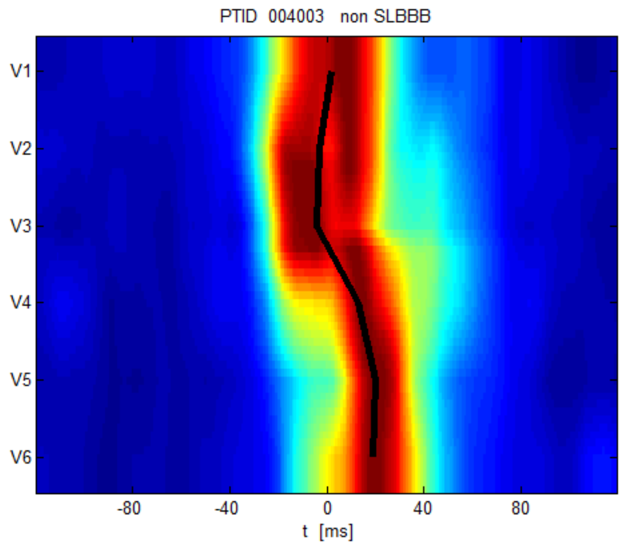

PTID 004003 non SLBBB
V1
V2
V3
V4
V5
V6
-80
-40
0
40
80
t [ms]

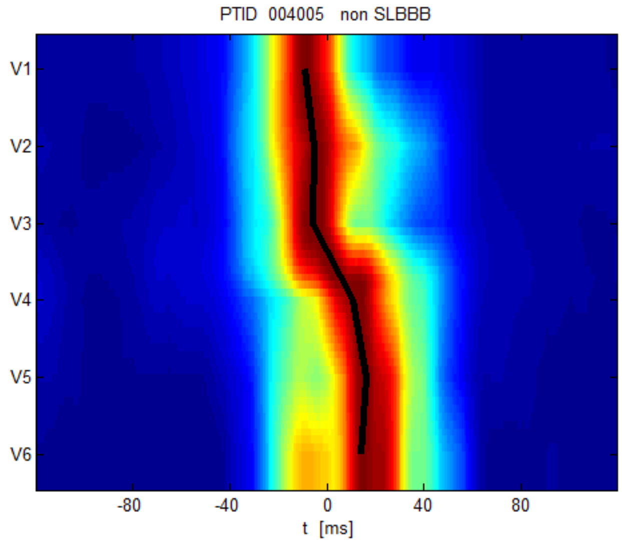

PTID 004005 non SLBBB
V1
V2
V3
V4
V5
V6
-80
-40
0
40
80
t [ms]

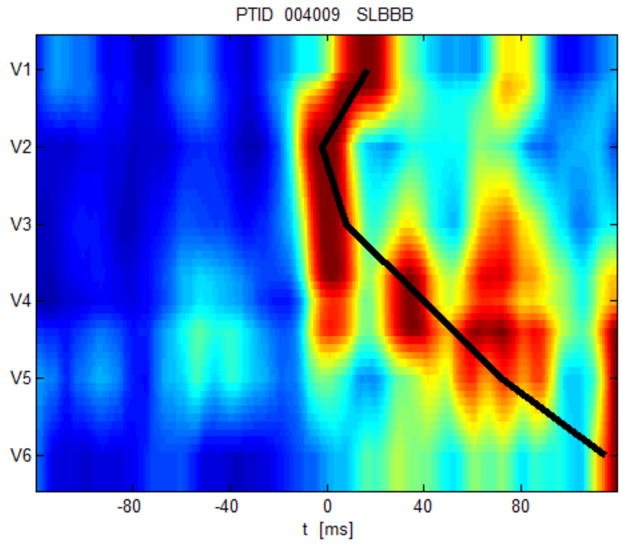

PTID 004009 SLBBB
V1
V2
V3
V4
V5
V6
-80
-40
0
40
80
t [ms]

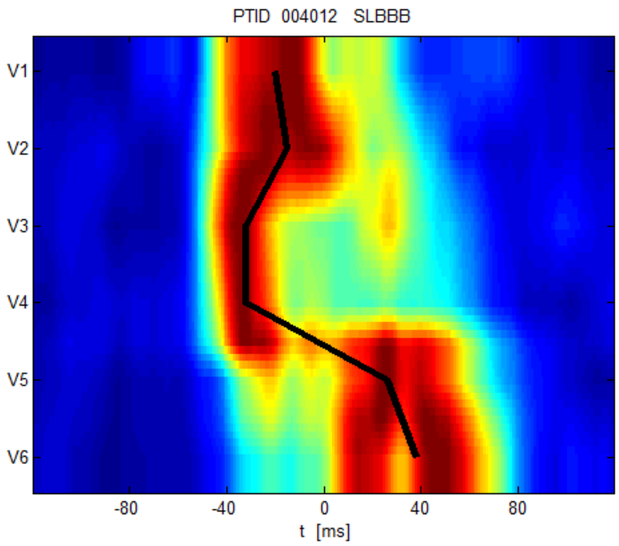
PTID 004012 SLBBB
V1
V2
V3
V4
V5
V6
-80
-40
0
40
80
t [ms]

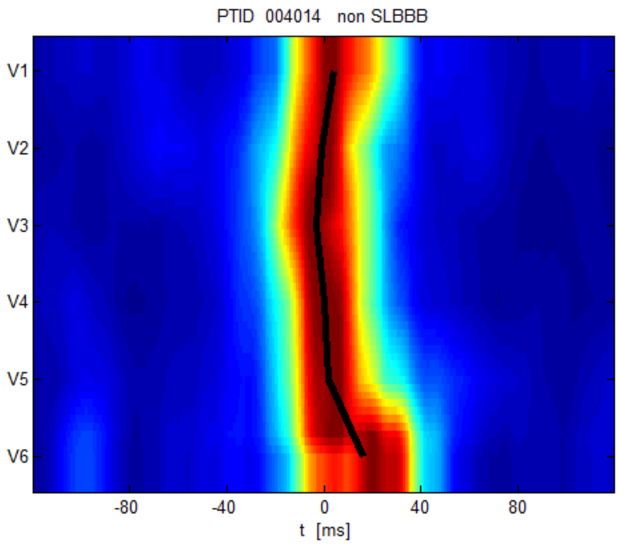
PTID 004014 non SLBBB
V1
V2
V3
V4
V5
V6
-80
-40
0
40
80
t [ms]

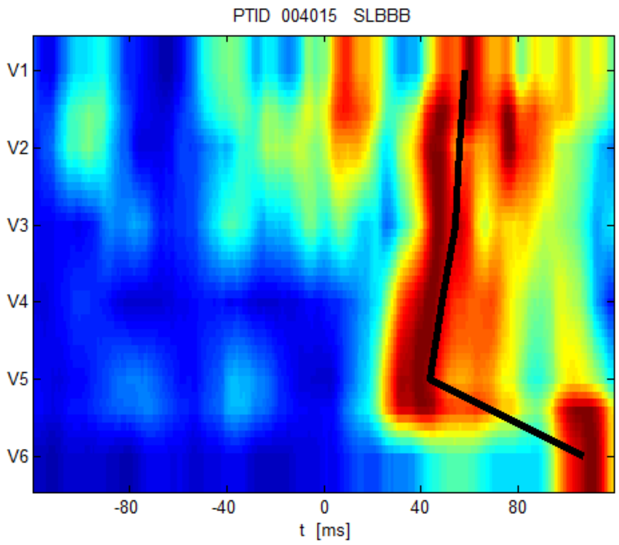
PTID 004015 SLBBB
V1
V2
V3
V4
V5
V6
-80
-40
0
40
80
t [ms]

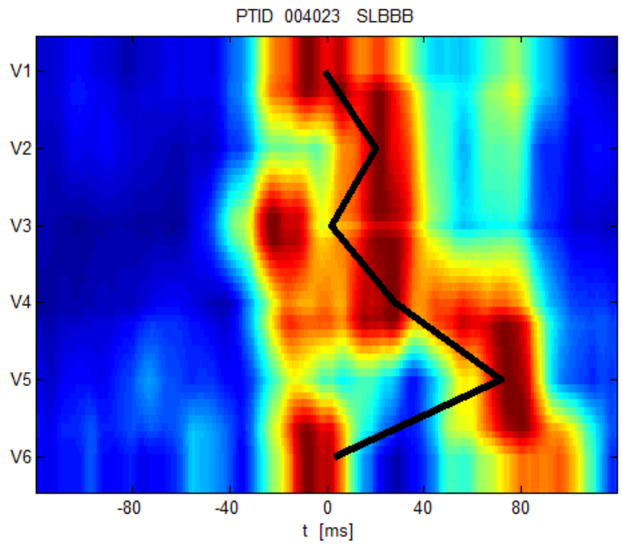
PTID 004023 SLBBB
V1
V2
V3
V4
V5
V6
-80
-40
0
40
80
t [ms]

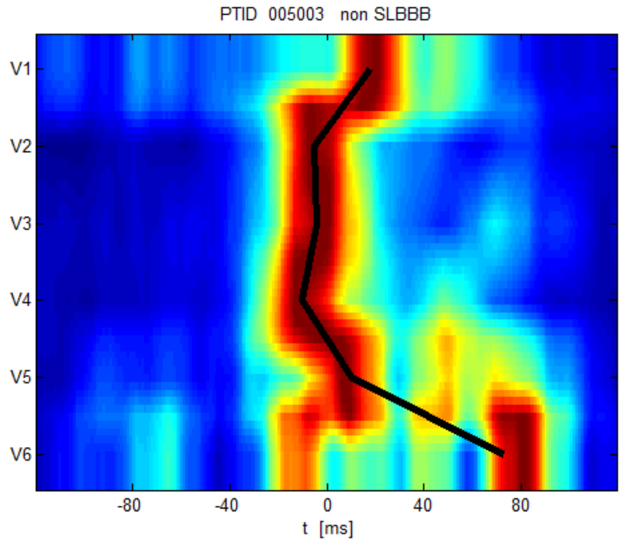
PTID 005003 non SLBBB
V1
V2
V3
V4
V5
V6
-80
-40
0
40
80
t [ms]

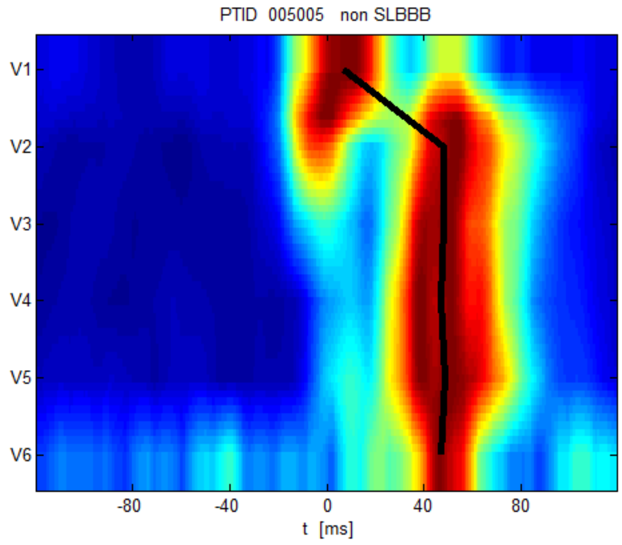
PTID 005005 non SLBBB
V1
V2
V3
V4
V5
V6
-80
-40
0
40
80
t [ms]

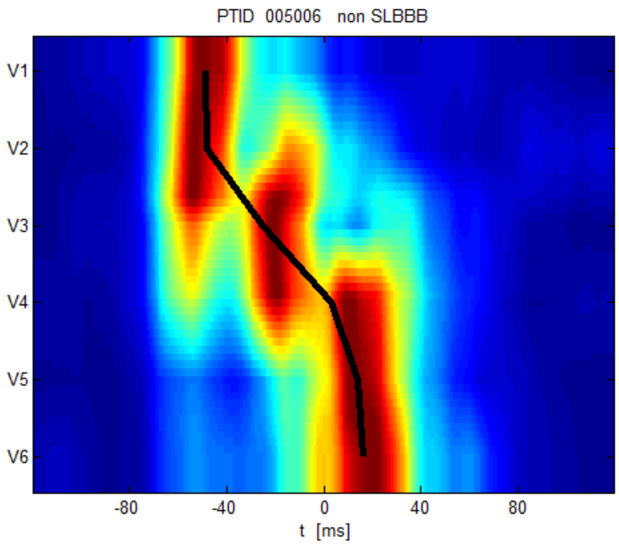
PTID 005006 non SLBBB
V1
V2
V3
V4
V5
V6
-80
-40
0
40
80
t [ms]

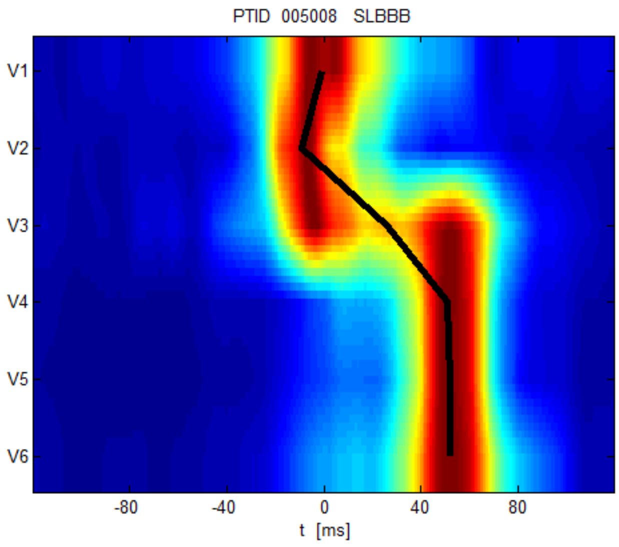
PTID 005008 SLBBB
V1
V2
V3
V4
V5
V6
-80
-40
0
40
80
t [ms]

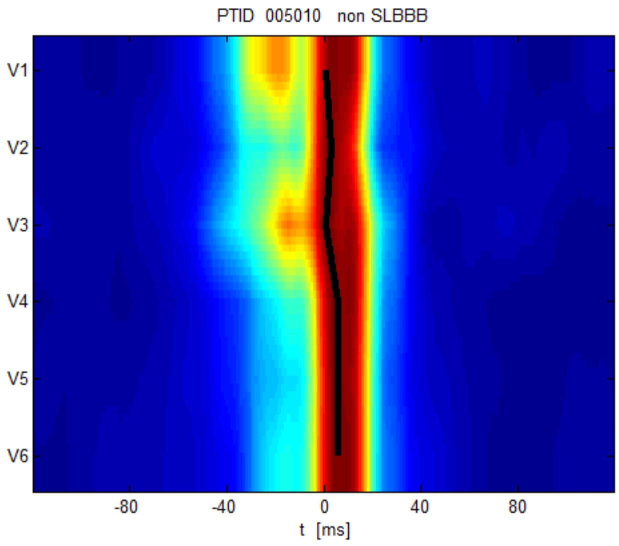
PTID 005010 non SLBBB
V1
V2
V3
V4
V5
V6
-80
-40
0
40
80
t [ms]

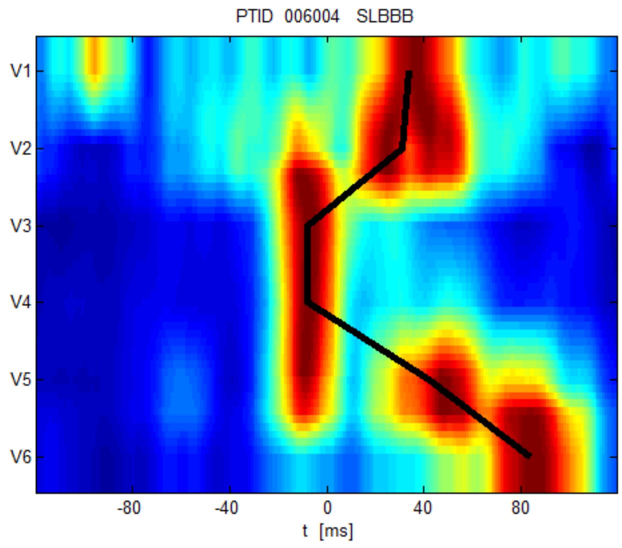
PTID 006004 SLBBB
V1
V2
V3
V4
V5
V6
-80
-40
0
40
80
t [ms]

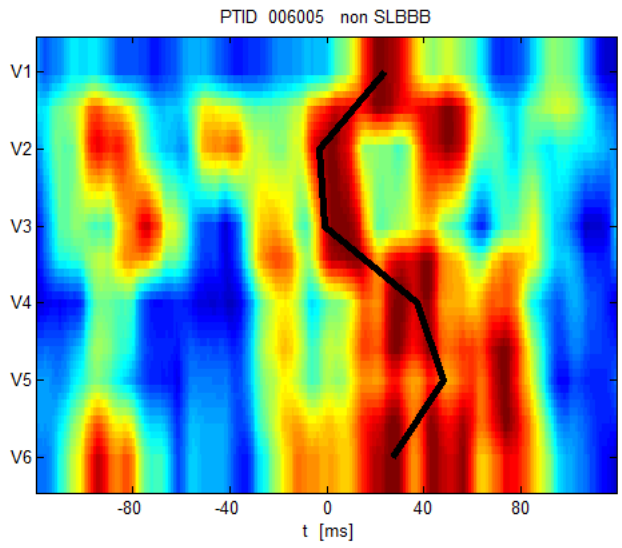
PTID 006005 non SLBBB
V1
V2
V3
V4
V5
V6
-80
-40
0
40
80
t [ms]

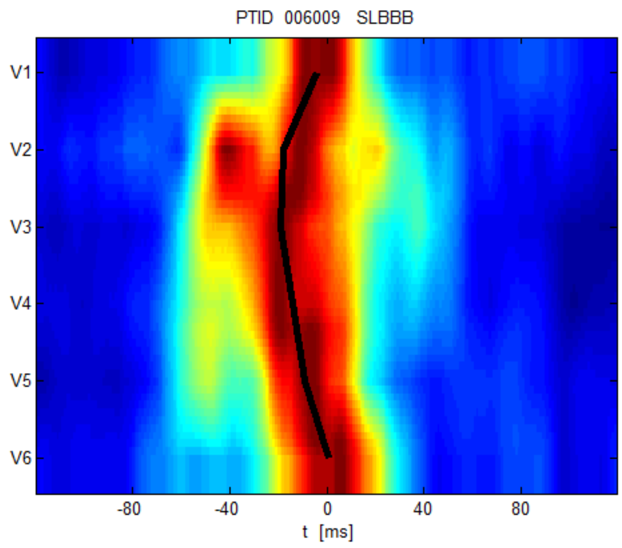
PTID 006009 SLBBB
V1
V2
V3
V4
V5
V6
-80
-40
0
40
80
t [ms]

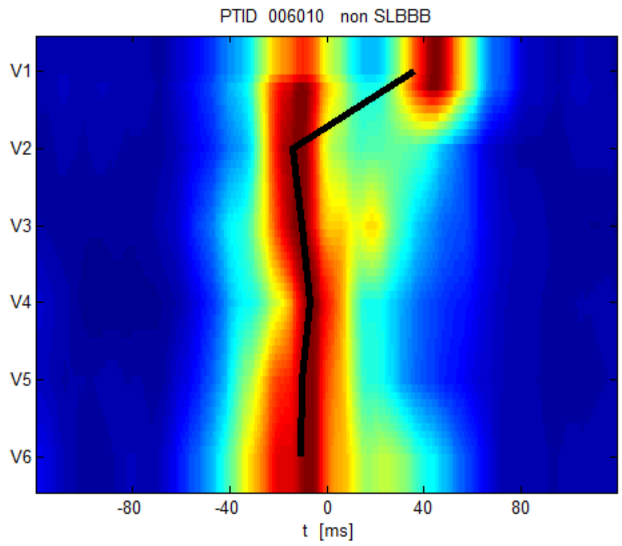
PTID 006010 non SLBBB
V1
V2
V3
V4
V5
V6
-80
-40
0
40
80
t [ms]

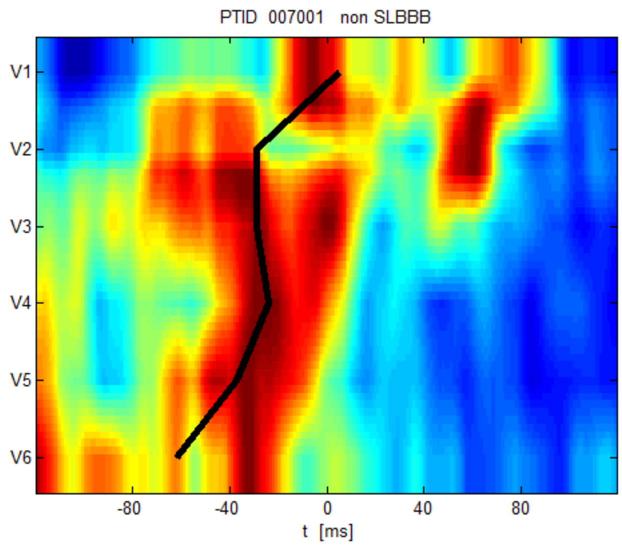
PTID 007001 non SLBBB
V1
V2
V3
V4
V5
V6
-80
-40
0
40
80
t [ms]

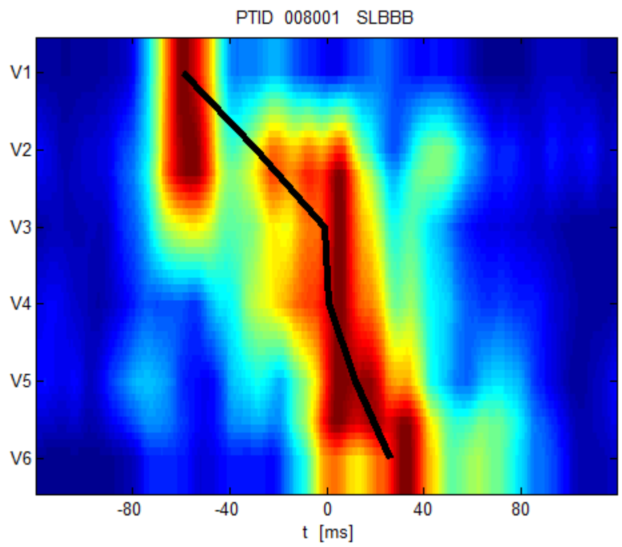
PTID 008001 SLBBB
V1
V2
V3
V4
V5
V6
-80
-40
0
40
80
t [ms]

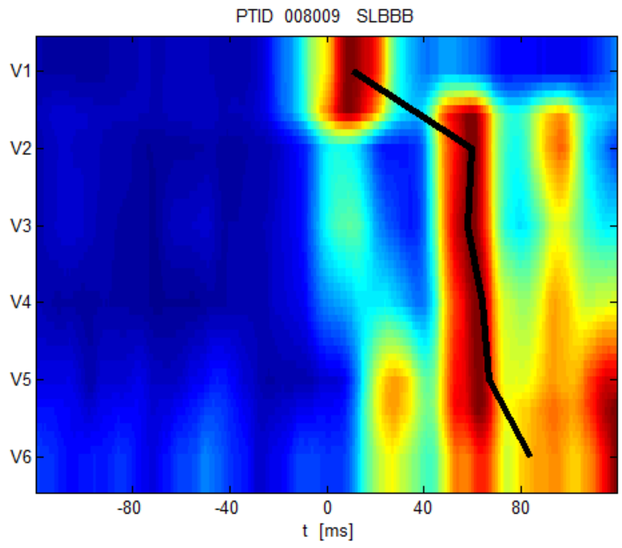
PTID 008009 SLBBB
V1
V2
V3
V4
V5
V6
-80
-40
0
40
80
t [ms]

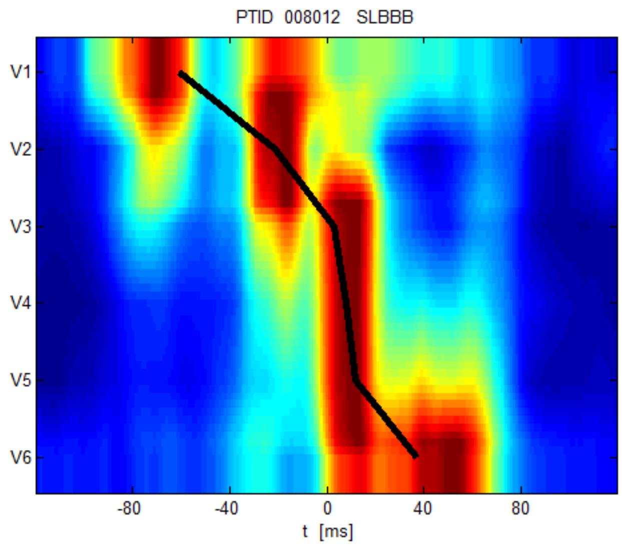
PTID 008012 SLBBB
V1
V2
V3
V4
V5
V6
-80
-40
0
40
80
t [ms]

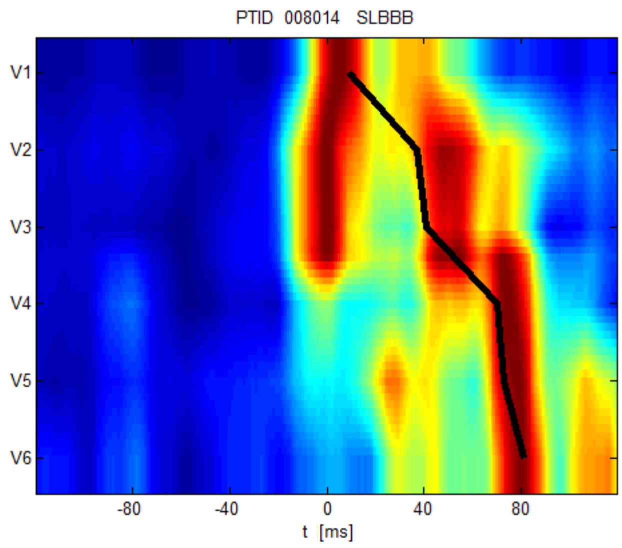
PTID 008014 SLBBB
V1
V2
V3
V4
V5
V6
-80
-40
0
40
80
t [ms]

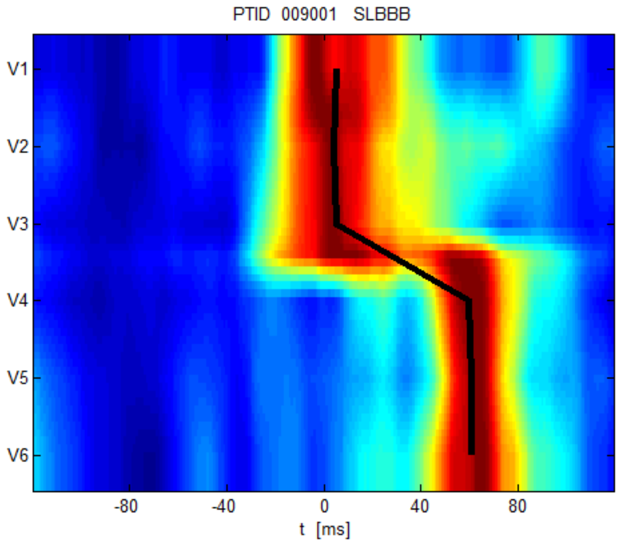

PTID 009001 SLBBB
V1
V2
V3
V4
V5
V6
-80
-40
0
40
80
t [ms]

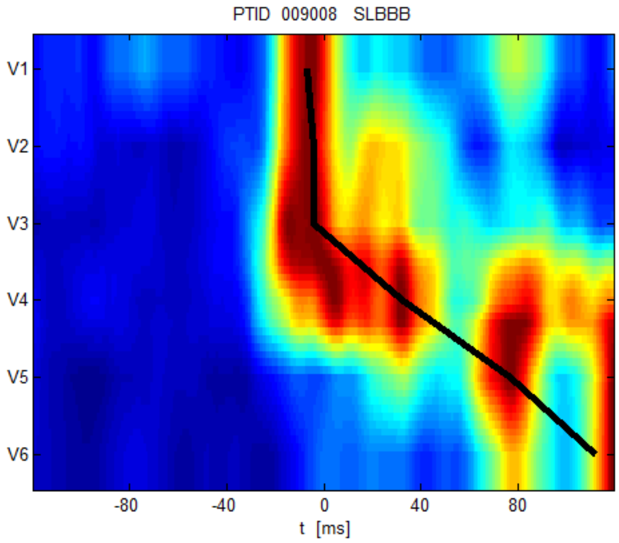

PTID 009008 SLBBB
V1
V2
V3
V4
V5
V6
-80
-40
0
40
80
t [ms]

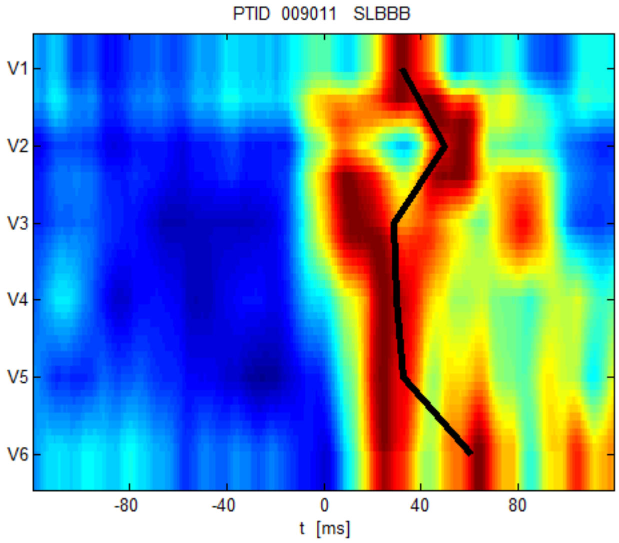

PTID 009011 SLBBB
V1
V2
V3
V4
V5
V6
-80
-40
0
40
80
t [ms]

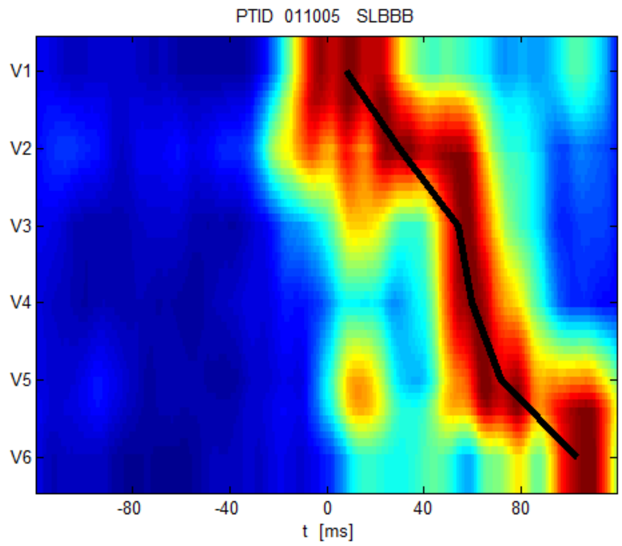
PTID 011005 SLBBB
V1
V2
V3
V4
V5
V6
-80
-40
0
40
80
t [ms]

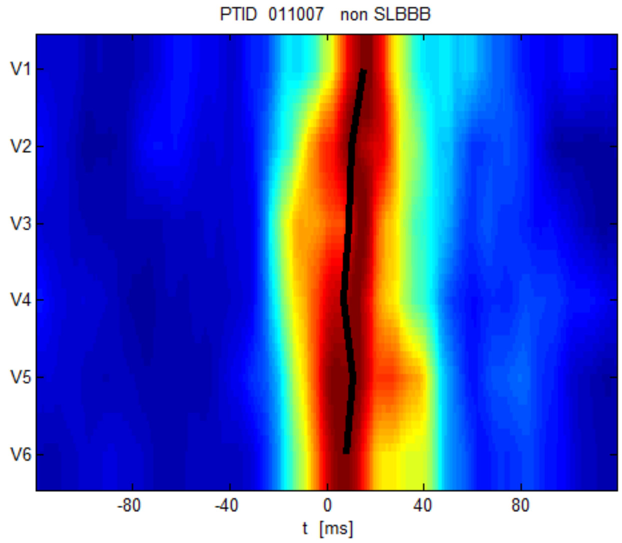
PTID 011007 non SLBBB
V1
V2
V3
V4
V5
V6
-80
-40
0
40
80
t [ms]

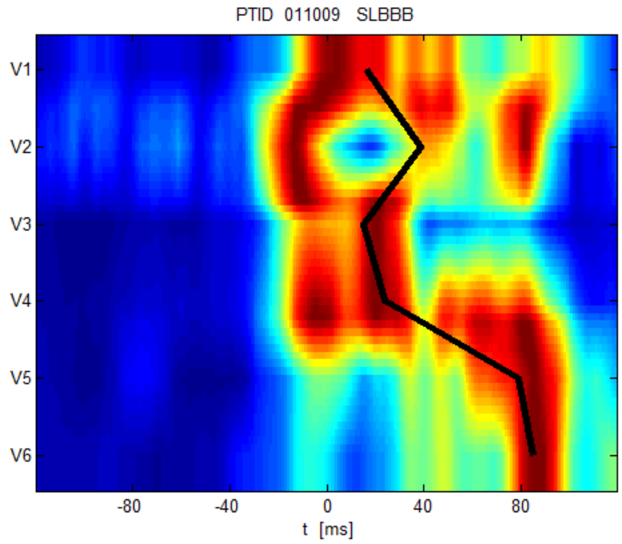
PTID 011009 SLBBB
V1
V2
V3
V4
V5
V6
-80
-40
0
40
80
t [ms]

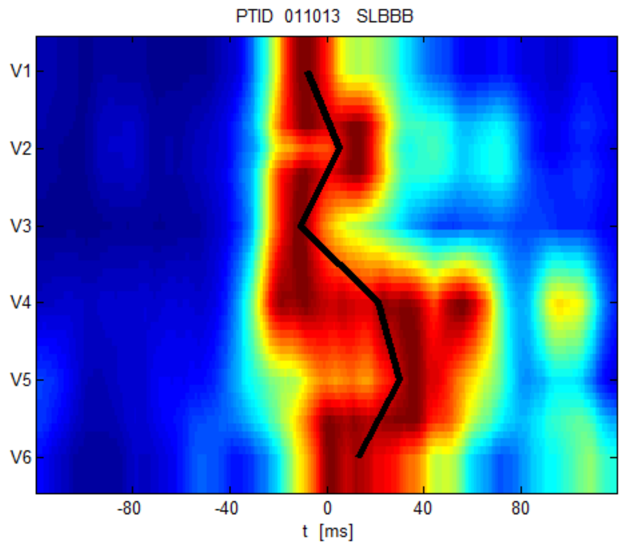
PTID 011013 SLBBB
V1
V2
V3
V4
V5
V6
-80
-40
0
40
80
t [ms]

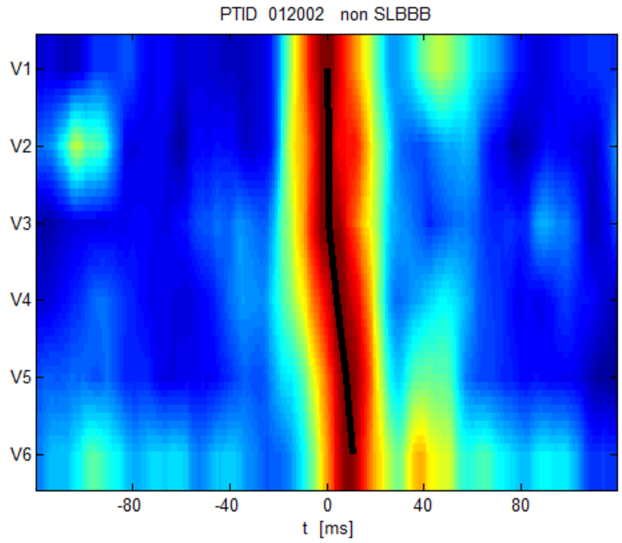
PTID 012002 non SLBBB
V1
V2
V3
V4
V5
V6
-80
-40
0
40
80
t [ms]

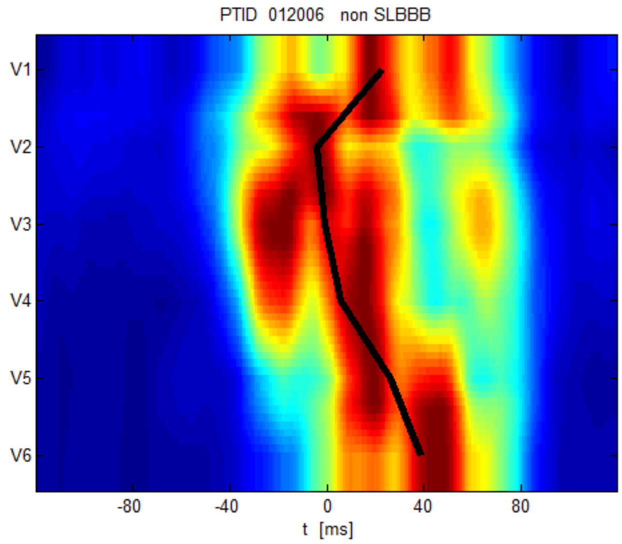
PTID 012006 non SLBBB
V1
V2
V3
V4
V5
V6
-80
-40
0
40
80
t [ms]

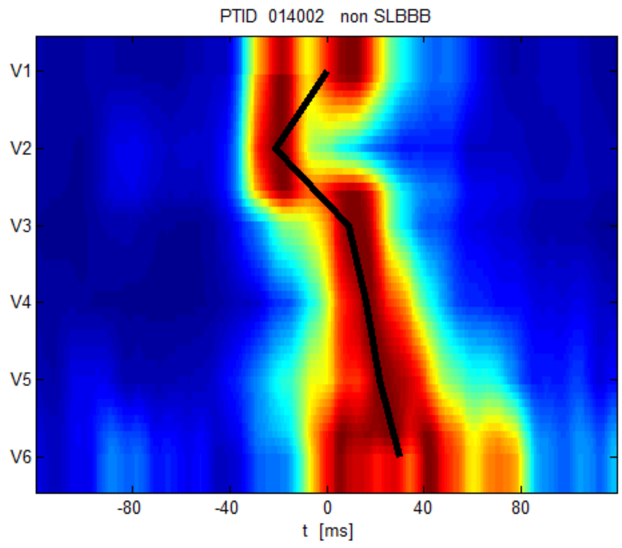
PTID 014002 non SLBBB
V1
V2
V3
V4
V5
V6
-80
-40
0
40
80
t [ms]

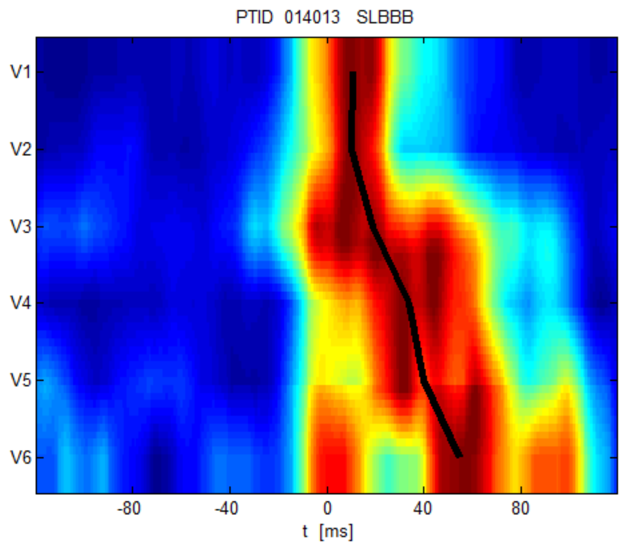
PTID 014013 SLBBB
V1
V2
V3
V4
V5
V6
-80
-40
0
40
80
t [ms]

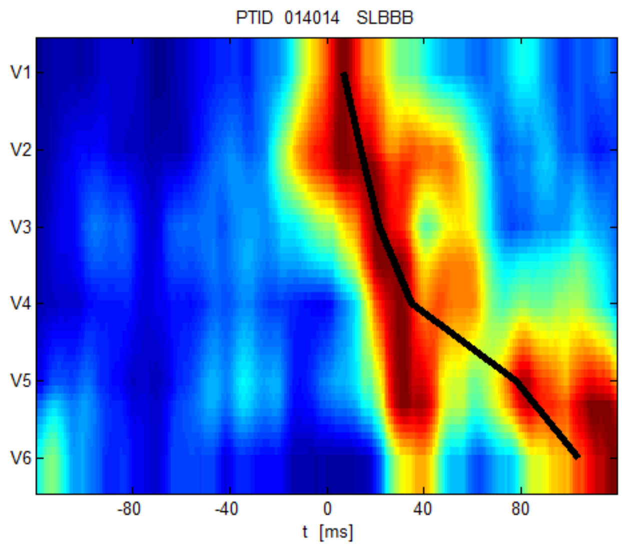
PTID 014014 SLBBB
V1
V2
V3
V4
V5
V6
-80
-40
0
40
80
t [ms]

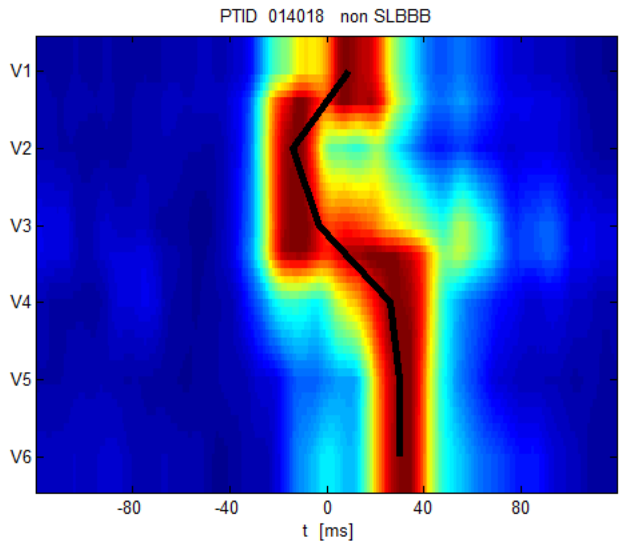
PTID 014018 non SLBBB
V1
V2
V3
V4
V5
V6
-80
-40
0
40
80
t [ms]

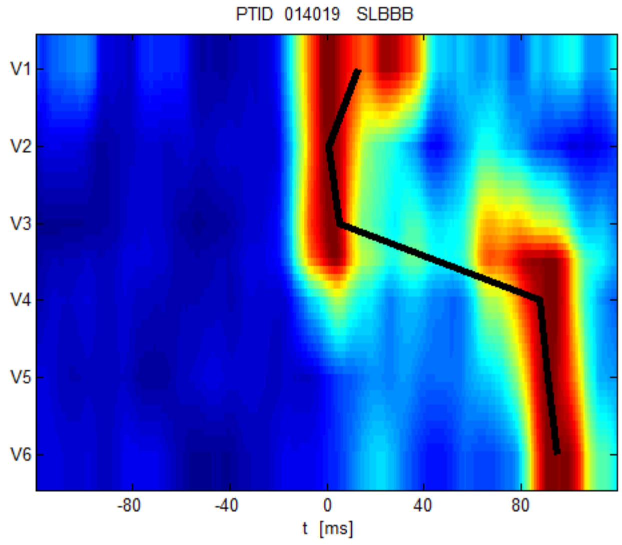
PTID 014019 SLBBB
V1
V2
V3
V4
V5
V6
-80
-40
0
40
80
t [ms]

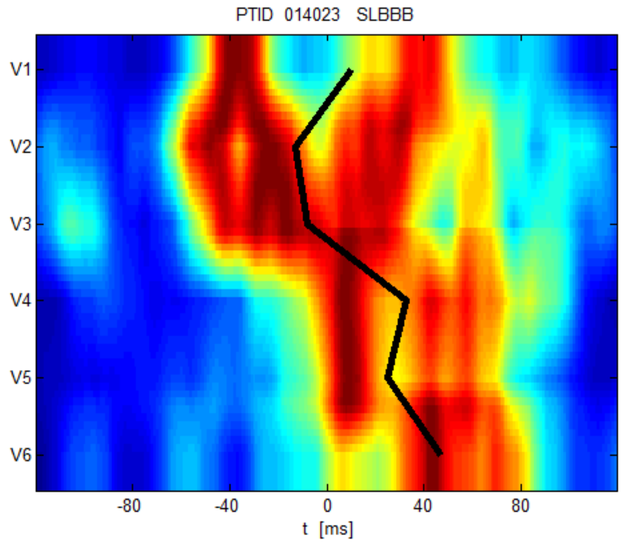
PTID 014023 SLBBB
V1
V2
V3
V4
V5
V6
-80
-40
0
40
80
t [ms]

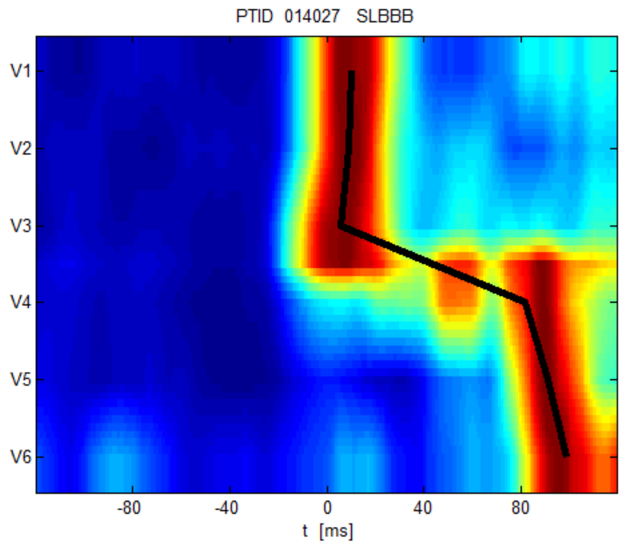
PTID 014027 SLBBB
V1
V2
V3
V4
V5
V6
-80
-40
0
40
80
t [ms]

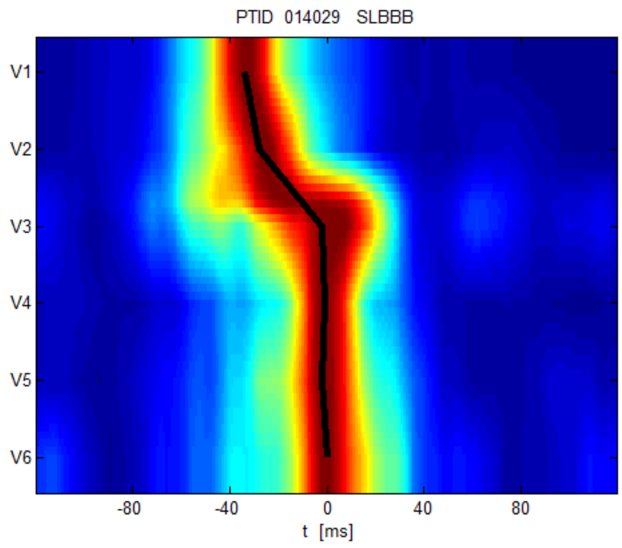
PTID 014029 SLBBB
V1
V2
V3
V4
V5
V6
-80
-40
0
40
80
t [ms]

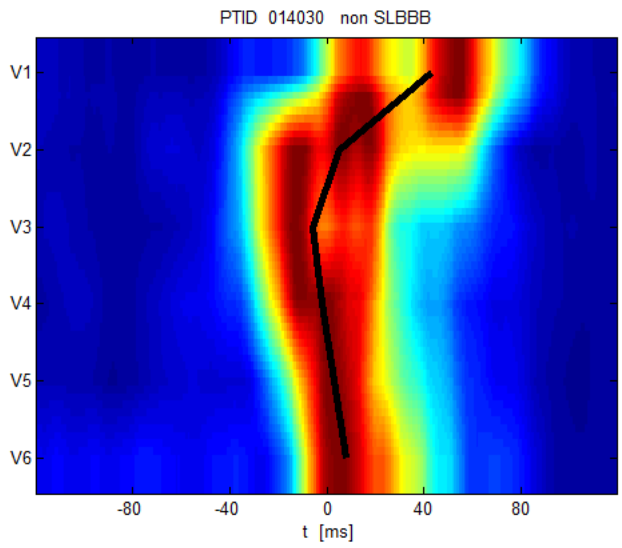
PTID 014030 non SLBBB
V1
V2
V3
V4
V5
V6
-80
-40
0
40
80
t [ms]

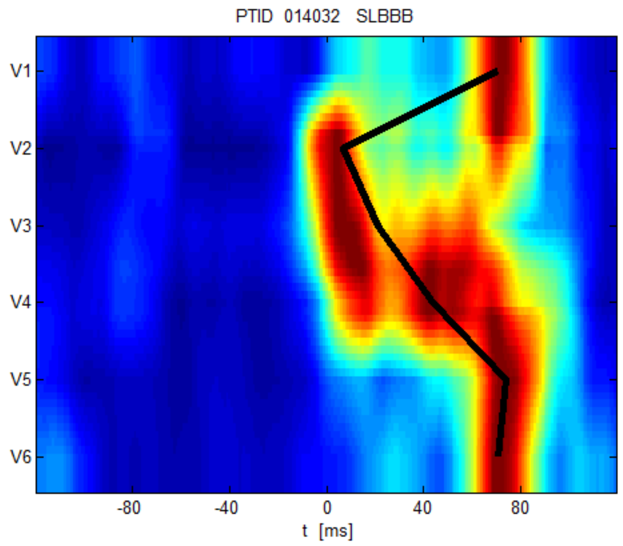
PTID 014032 SLBBB
V1
V2
V3
V4
V5
V6
-80
-40
0
40
80
t [ms]

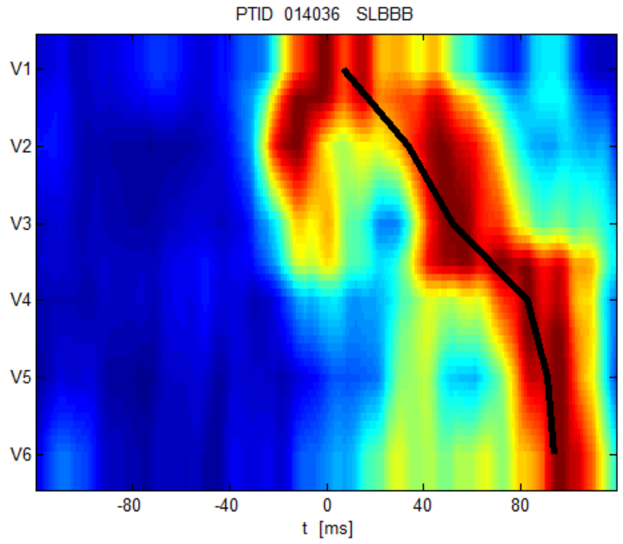
PTID 014036 SLBBB
V1
V2
V3
V4
V5
V6
-80
-40
0
40
80
t [ms]

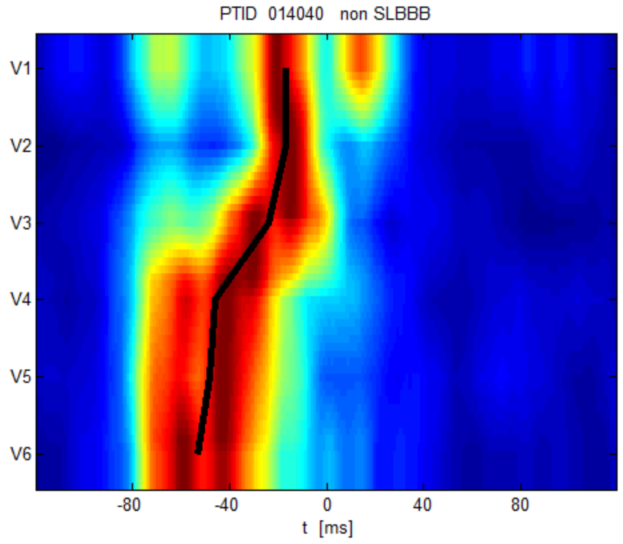
PTID 014040 non SLBBB
V1
V2
V3
V4
V5
V6
-80
-40
0
40
80
t [ms]

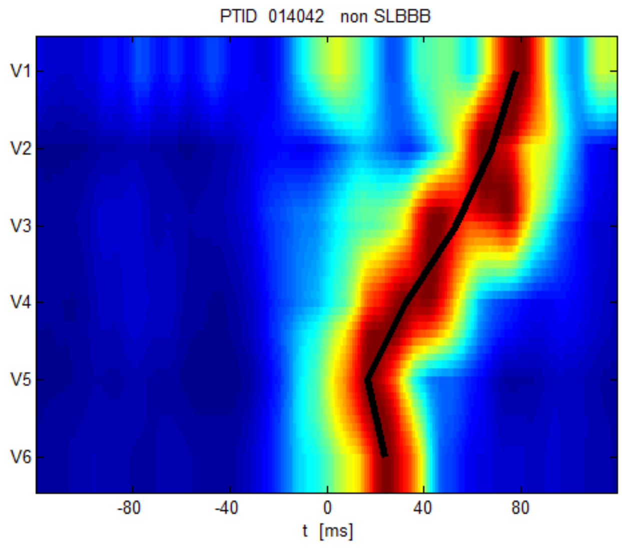
PTID 014042 non SLBBB
V1
V2
V3
V4
V5
V6
-80
-40
0
40
80
t [ms]

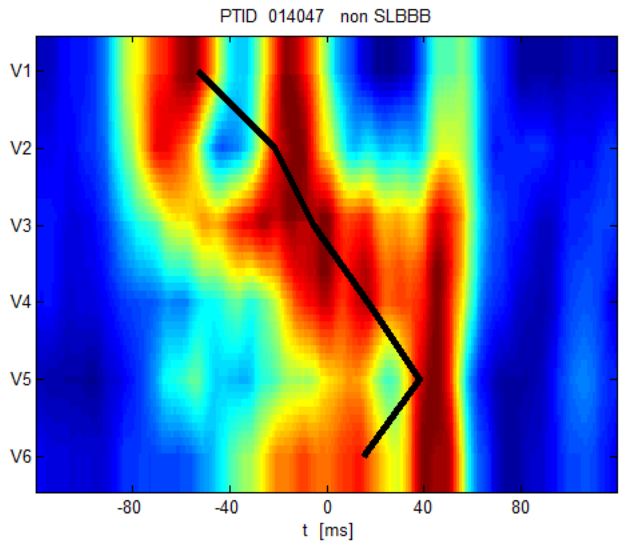
PTID 014047 non SLBBB
V1
V2
V3
V4
V5
V6
-80
-40
0
40
80
t [ms]

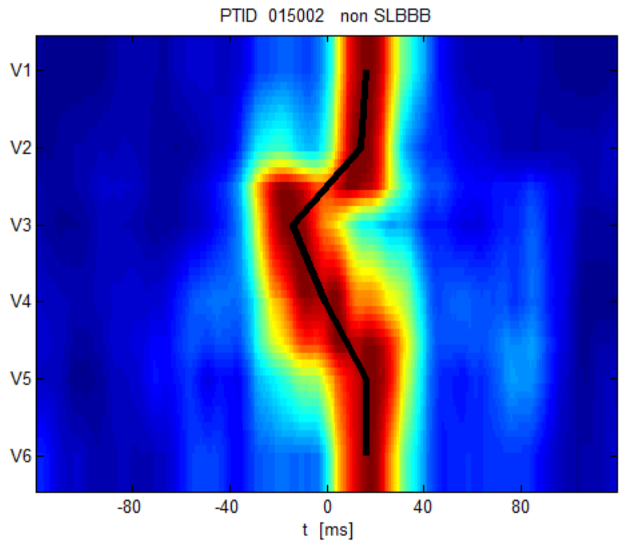
PTID 015002 non SLBBB
V1
V2
V3
V4
V5
V6
-80
-40
0
40
80
t [ms]

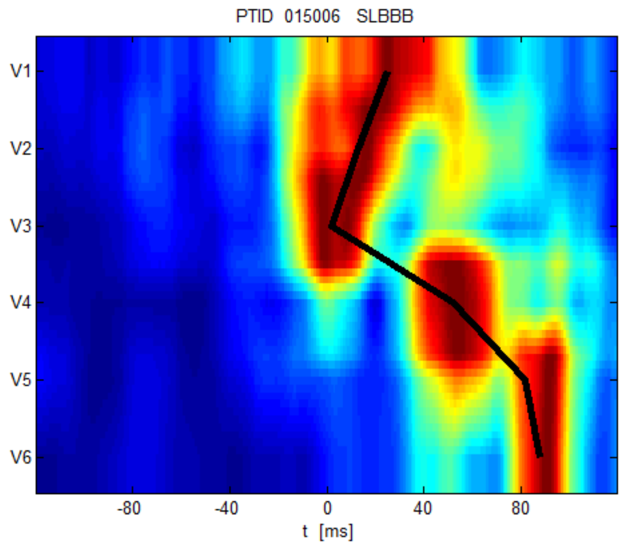
PTID 015006 SLBBB
V1
V2
V3
V4
V5
V6
-80
-40
0
40
80
t [ms]

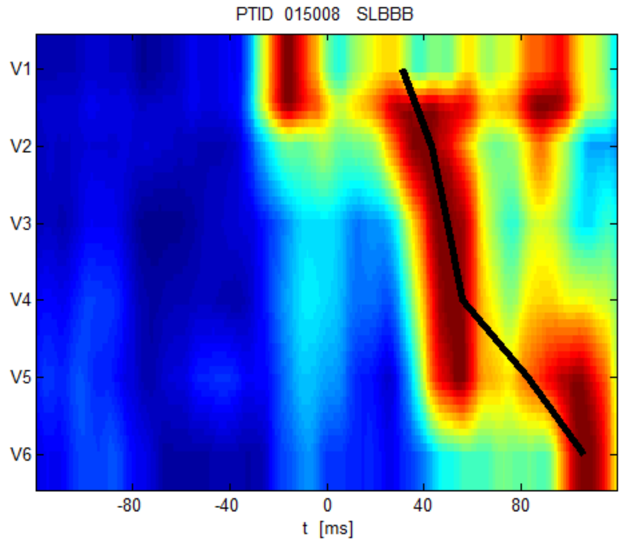
PTID 015008 SLBBB
V1
V2
V3
V4
V5
V6
-80
-40
0
40
80
t [ms]

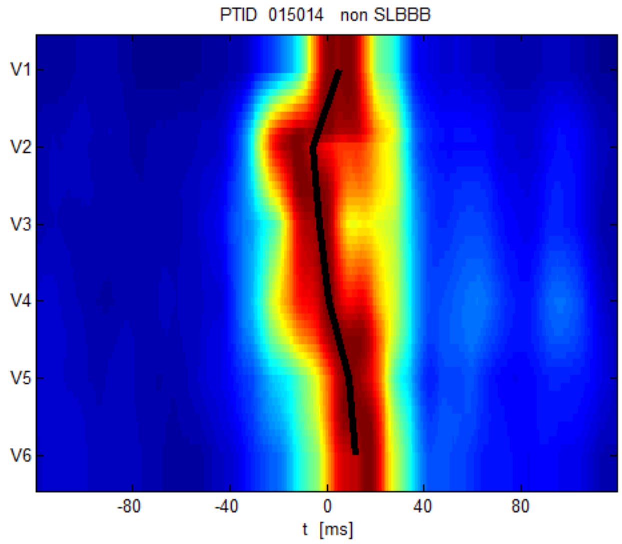
PTID 015014 non SLBBB
V1
V2
V3
V4
V5
V6
-80
-40
0
40
80
t [ms]

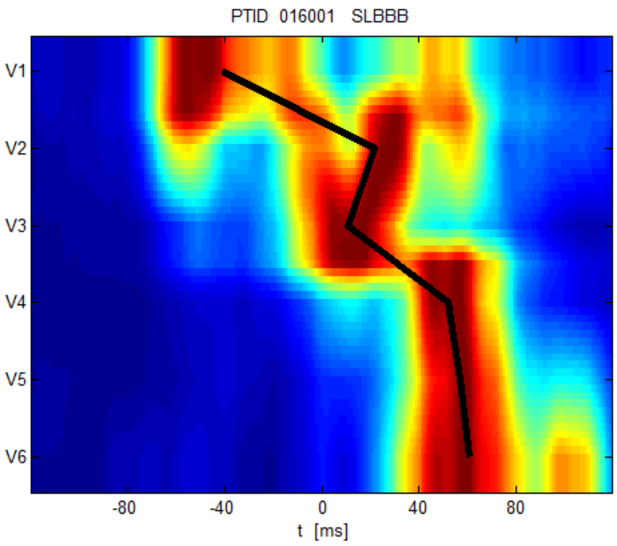
PTID 016001 SLBBB
V1
V2
V3
V4
V5
V6
-80
-40
0
40
80
t [ms]

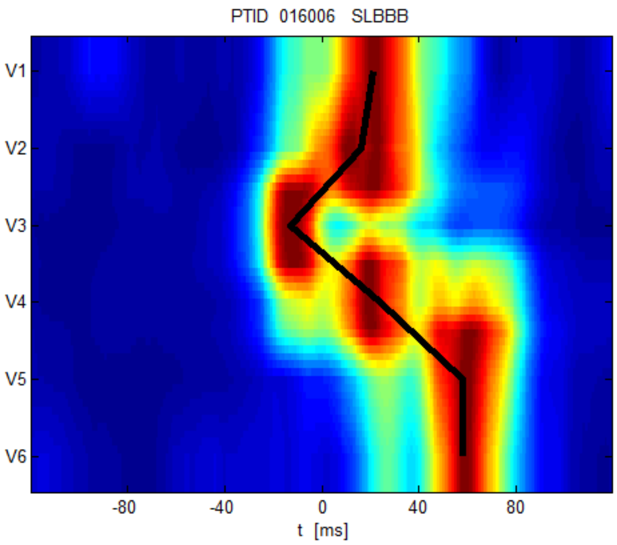
PTID 016006 SLBBB
V1
V2
V3
V4
V5
V6
-80
-40
0
40
80
t [ms]

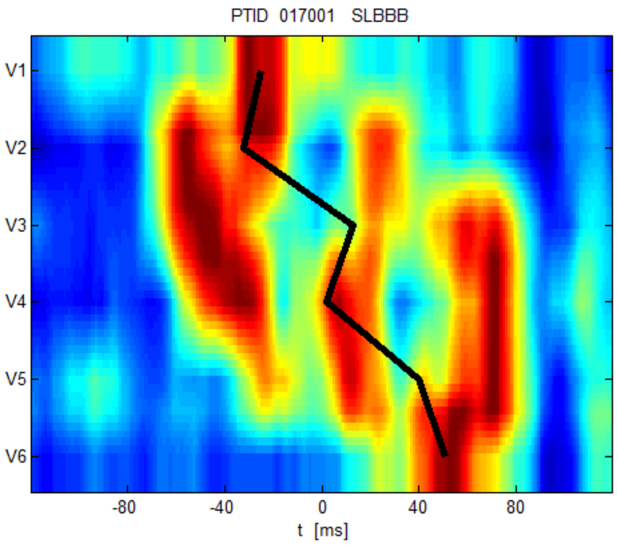
PTID 017001 SLBBB
V1
V2
V3
V4
V5
V6
-80
-40
0
40
80
t [ms]

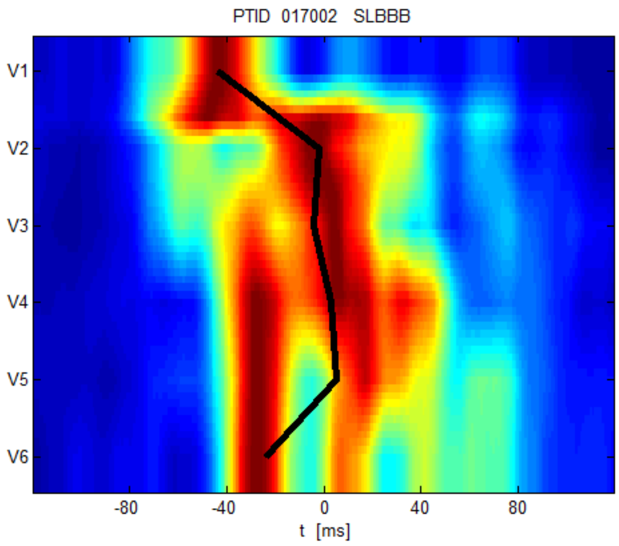
PTID 017002 SLBBB
V1
V2
V3
V4
V5
V6
-80
-40
0
40
80
t [ms]

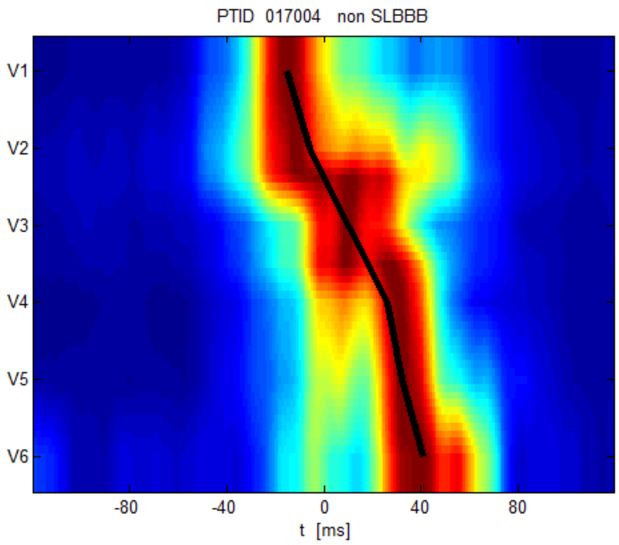
PTID 017004 non SLBBB
V1
V2
V3
V4
V5
V6
-80
-40
0
40
80
t [ms]

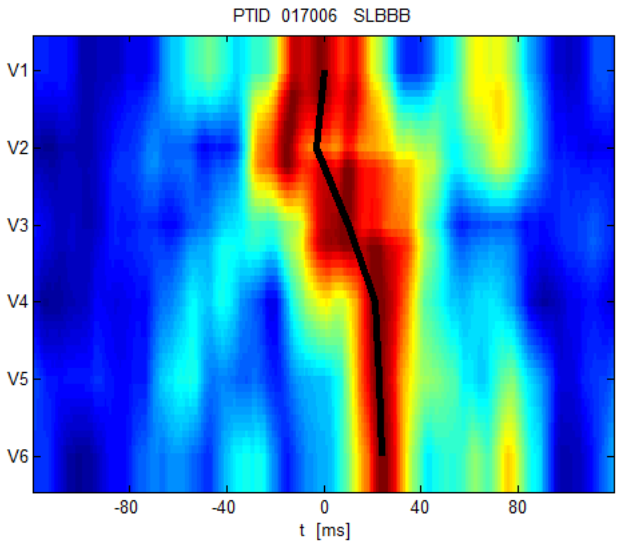
PTID 017006 SLBBB
V1
V2
V3
V4
V5
V6
-80
-40
0
40
80
t [ms]

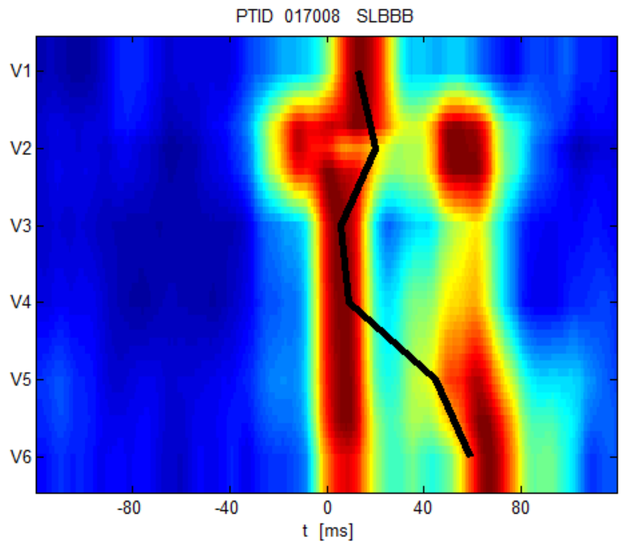
PTID 017008 SLBBB
V1
V2
V3
V4
V5
V6
-80
-40
0
40
80
t [ms]

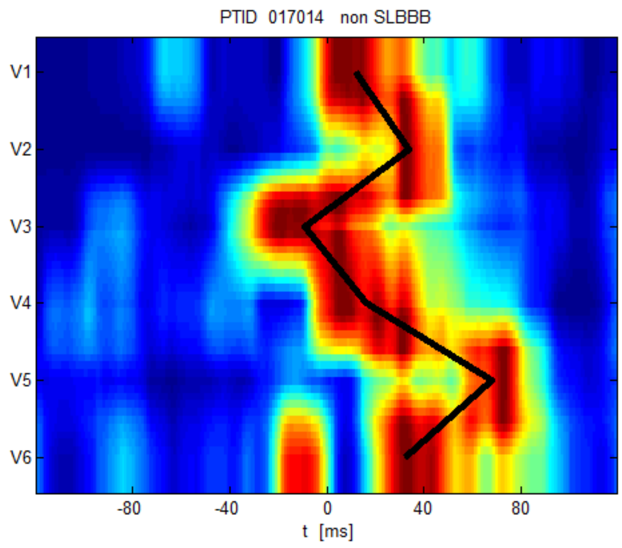
PTID 017014 non SLBBB
V1
V2
V3
V4
V5
V6
-80
-40
0
40
80
t [ms]

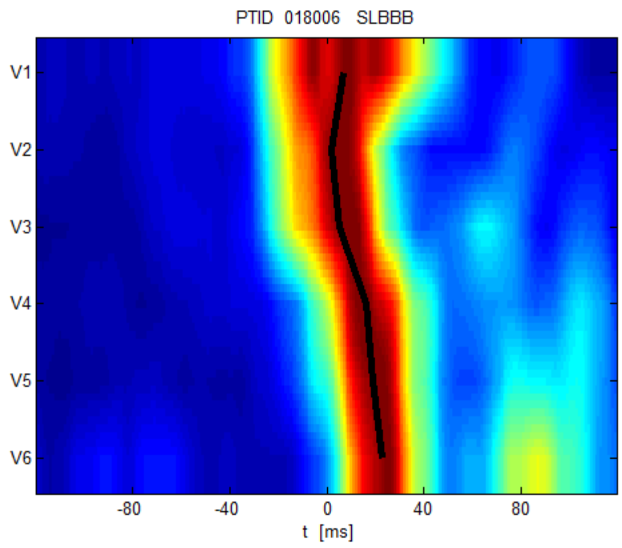
PTID 018006 SLBBB
V1
V2
V3
V4
V5
V6
-80
-40
0
40
80
t [ms]

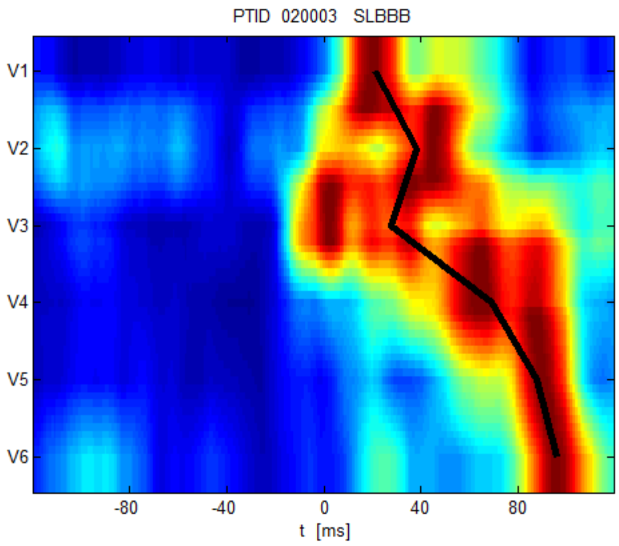
PTID 020003 SLBBB
V1
V2
V3
V4
V5
V6
-80
-40
0
40
80
t [ms]

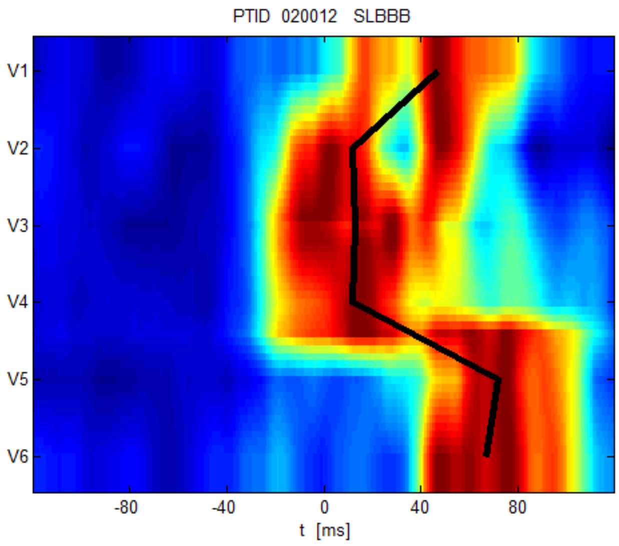
PTID 020012 SLBBB
V1
V2
V3
V4
V5
V6
-80
-40
0
40
80
t [ms]

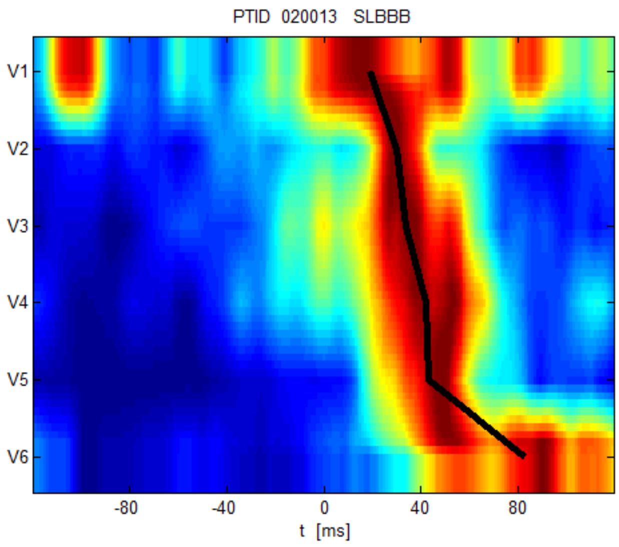
PTID 020013 SLBBB
V1
V2
V3
V4
V5
V6
-80
-40
0
40
80
t [ms]

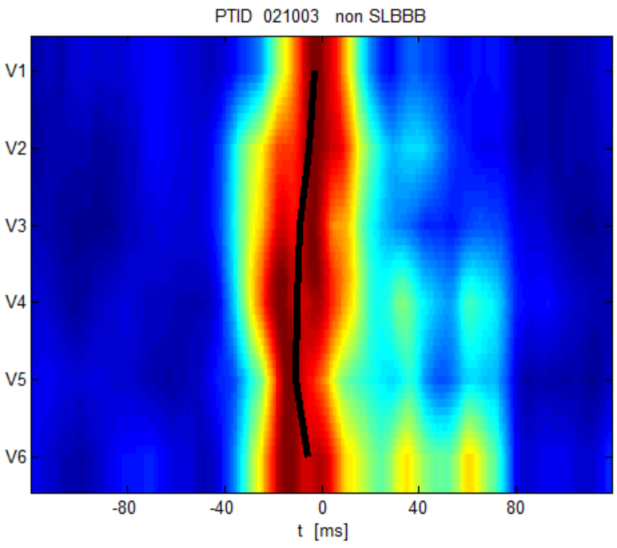
PTID 021003 non SLBBB
V1
V2
V3
V4
V5
V6
-80
-40
0
40
80
t [ms]

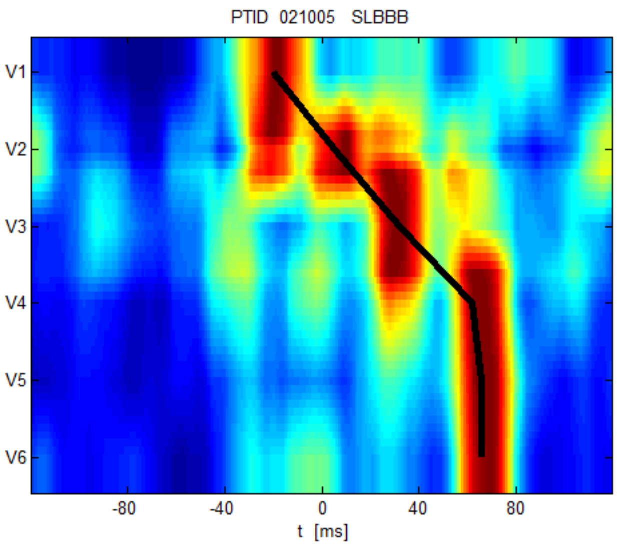
PTID 021005 SLBBB
V1
V2
V3
V4
V5
V6
-80
-40
0
40
80
t [ms]

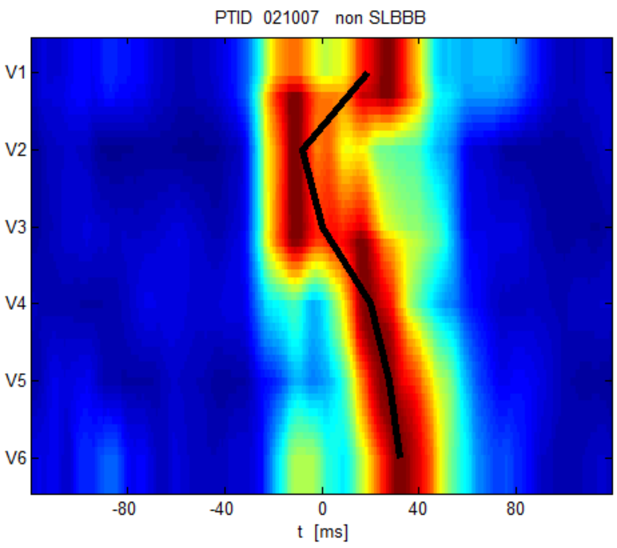
PTID 021007 non SLBBB
V1
V2
V3
V4
V5
V6
-80
-40
0
40
80
t [ms]

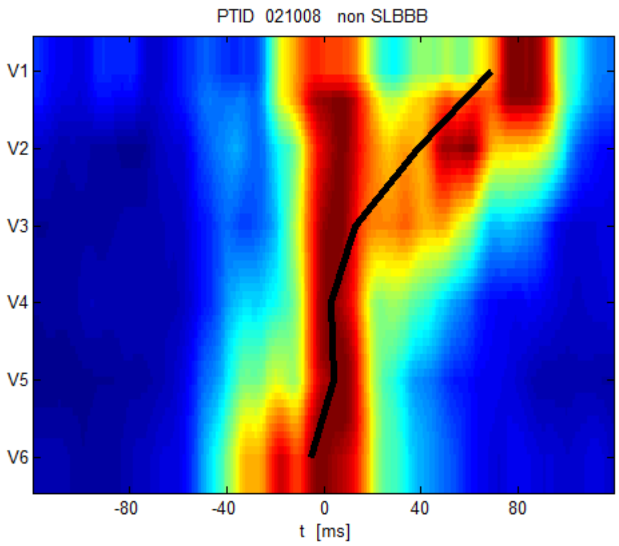
PTID 021008 non SLBBB
V1
V2
V3
V4
V5
V6
-80
-40
0
40
80
t [ms]

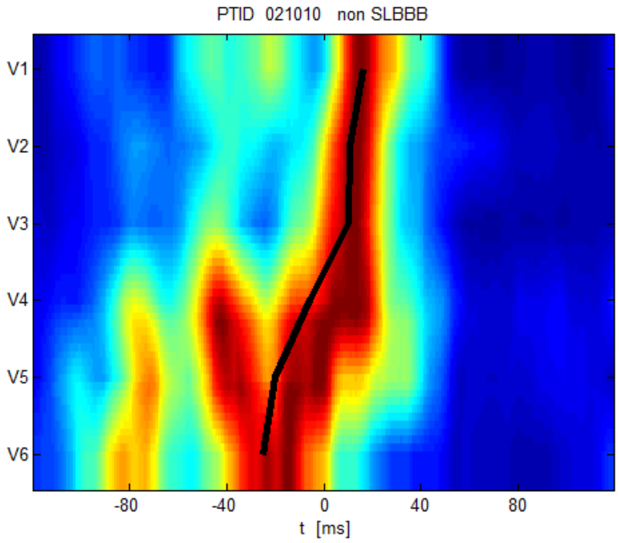
PTID 021010 non SLBBB
V1
V2
V3
V4
V5
V6
-80
-40
0
40
80
t [ms]

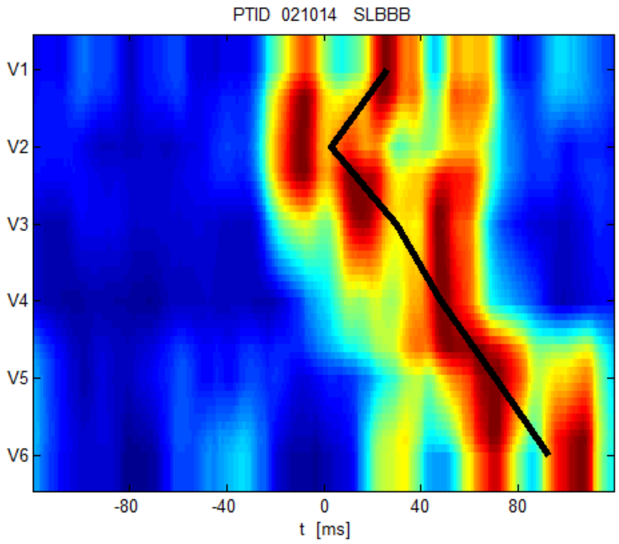
PTID 021014 SLBBB
V1
V2
V3
V4
V5
V6
-80
-40
0
40
80
t [ms]

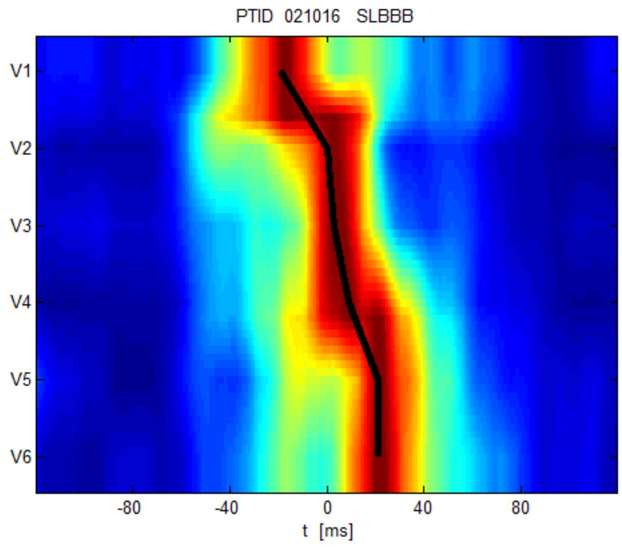
PTID 021016 SLBBB
V1
V2
V3
V4
V5
V6
-80
-40
0
40
80
t [ms]

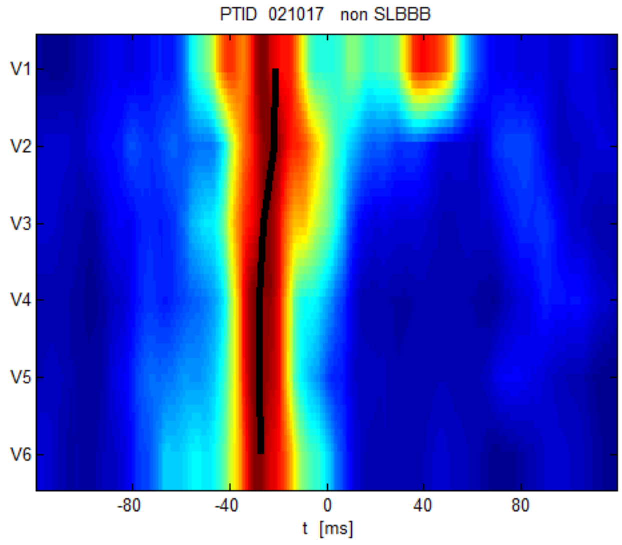
PTID 021017 non SLBBB
V1
V2
V3
V4
V5
V6
-80
-40
0
40
80
t [ms]

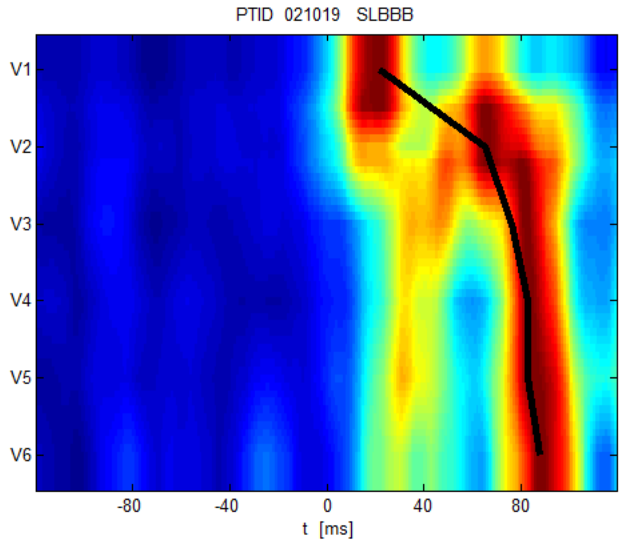
PTID 021019 SLBBB
V1
V2
V3
V4
V5
V6
-80
-40
0
40
80
t [ms]

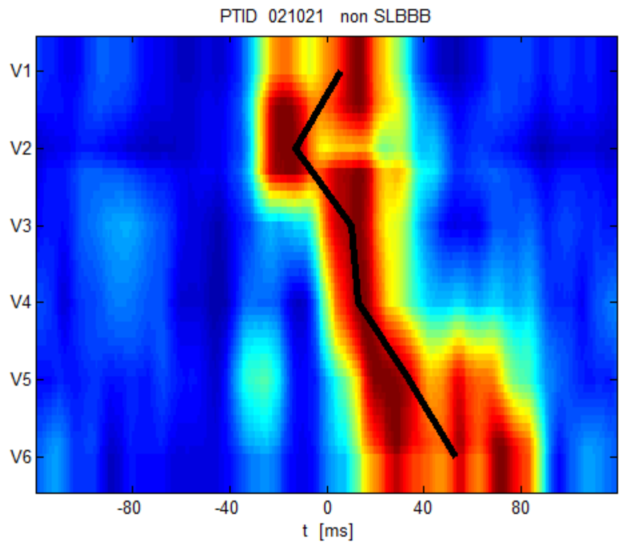
PTID 021021 non SLBBB
V1
V2
V3
V4
V5
V6
-80
-40
0
40
80
t [ms]

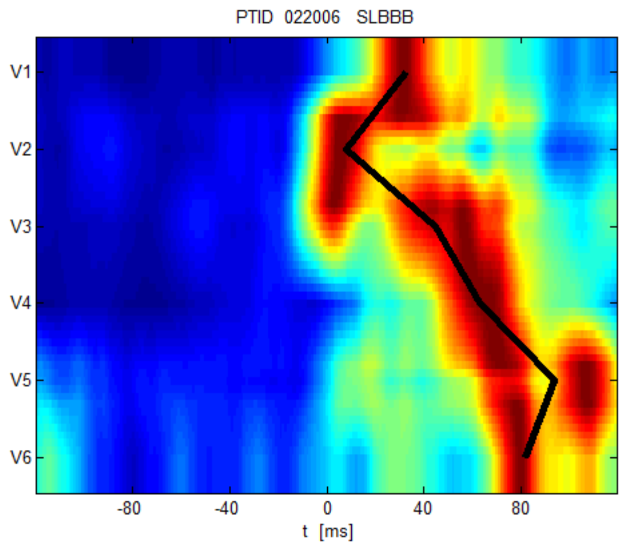
PTID 022006 SLBBB
V1
V2
V3
V4
V5
V6
-80
-40
0
40
80
t [ms]

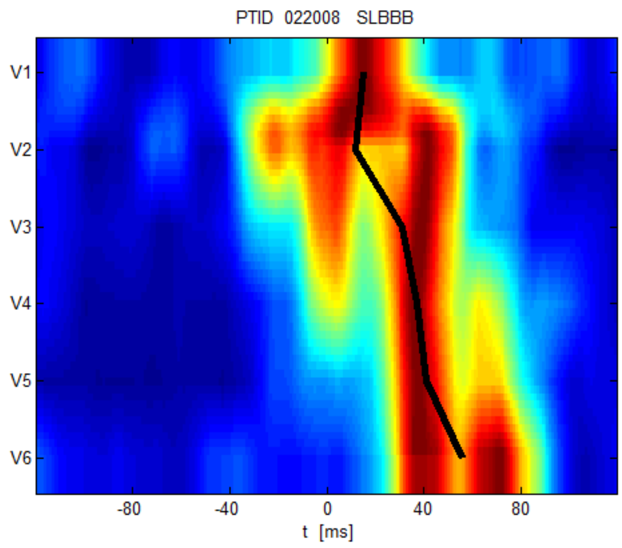
PTID 022008 SLBBB
V1
V2
V3
V4
V5
V6
-80
-40
0
40
80
t [ms]

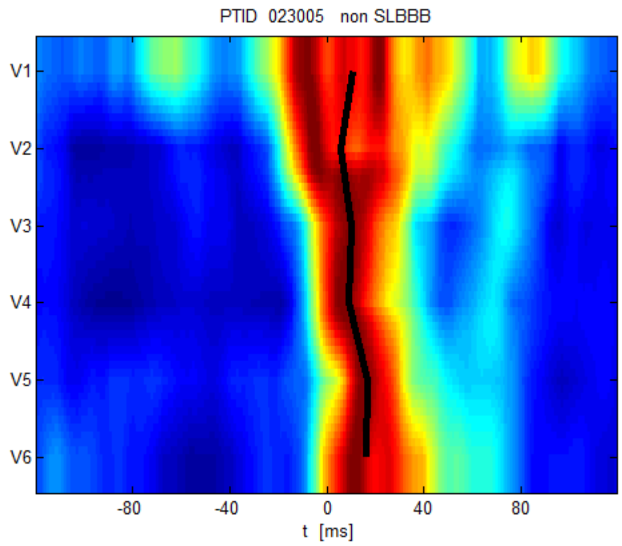
PTID 023005 non SLBBB
V1
V2
V3
V4
V5
V6
-80
-40
0
40
80
t [ms]

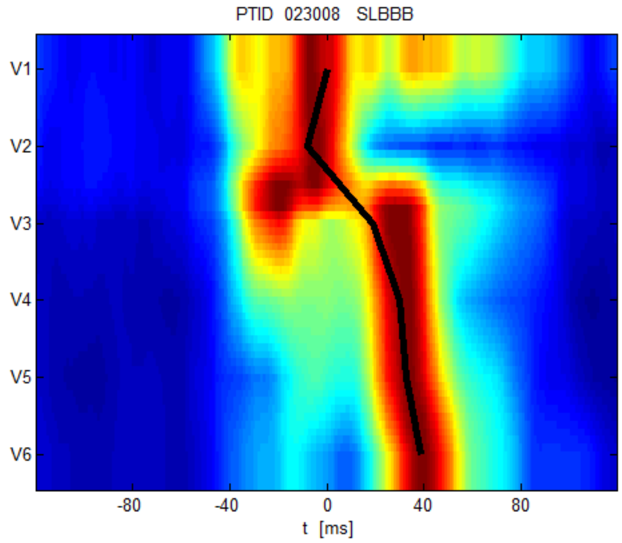
PTID 023008 SLBBB
V1
V2
V3
V4
V5
V6
-80
-40
0
40
80
t [ms]

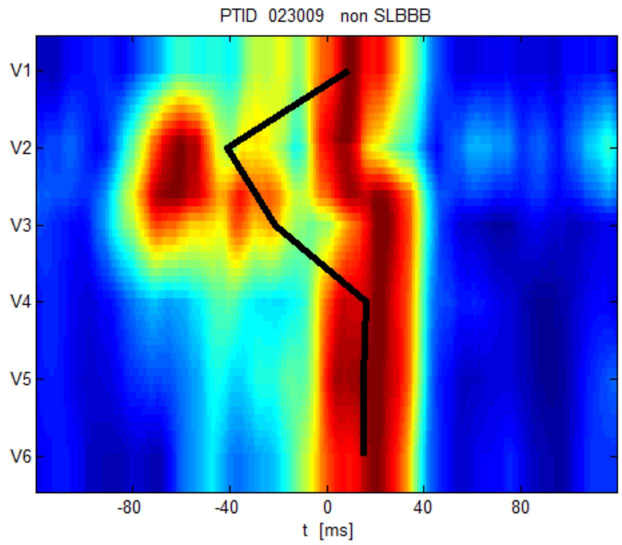
PTID 023009 non SLBBB
V1
V2
V3
V4
V5
V6
-80
-40
0
40
80
t [ms]

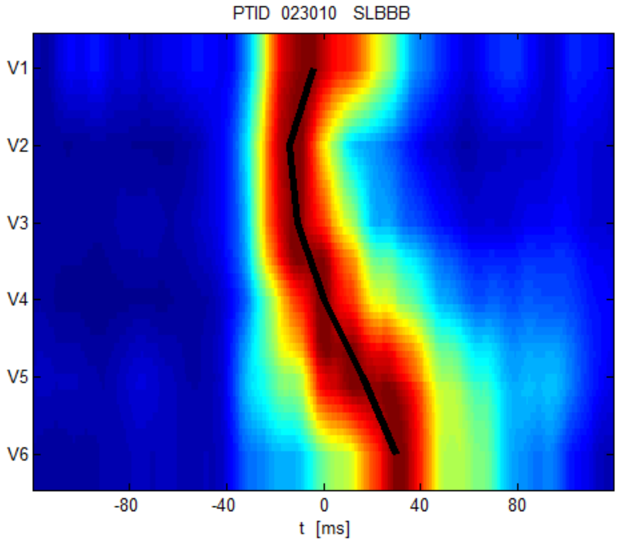
PTID 023010 SLBBB
V1
V2
V3
V4
V5
V6
-80
-40
0
40
80
t [ms]

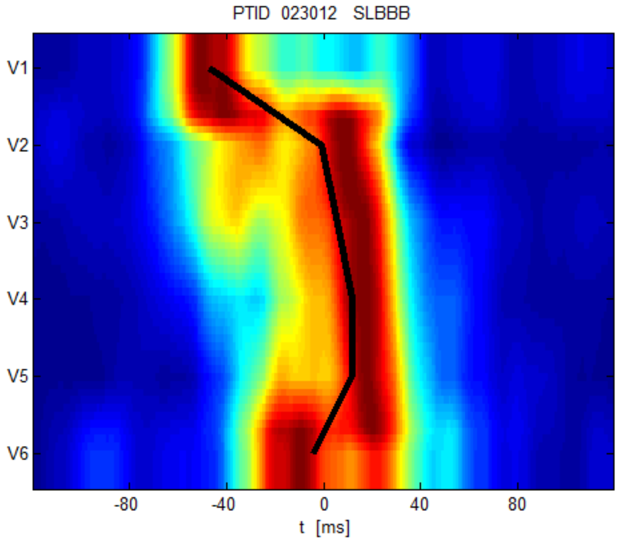
PTID 023012 SLBBB
V1
V2
V3
V4
V5
V6
-80
-40
0
40
80
t [ms]

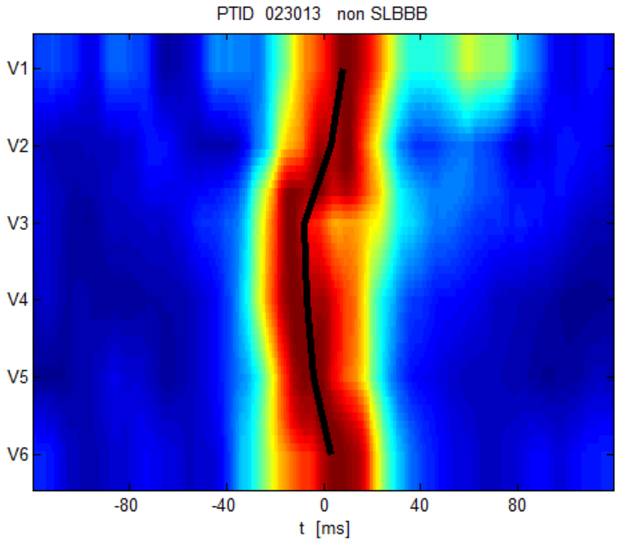
PTID 023013 non SLBBB
V1
V2
V3
V4
V5
V6
-80
-40
0
40
80
t [ms]

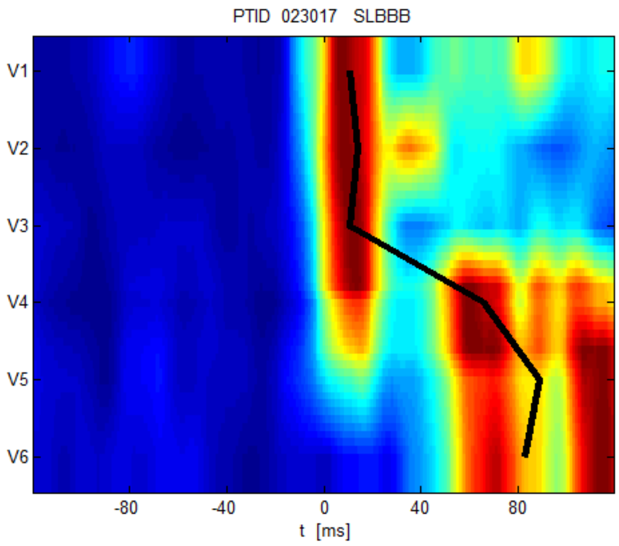
PTID 023017 SLBBB
V1
V2
V3
V4
V5
V6
-80
-40
0
40
80
t [ms]

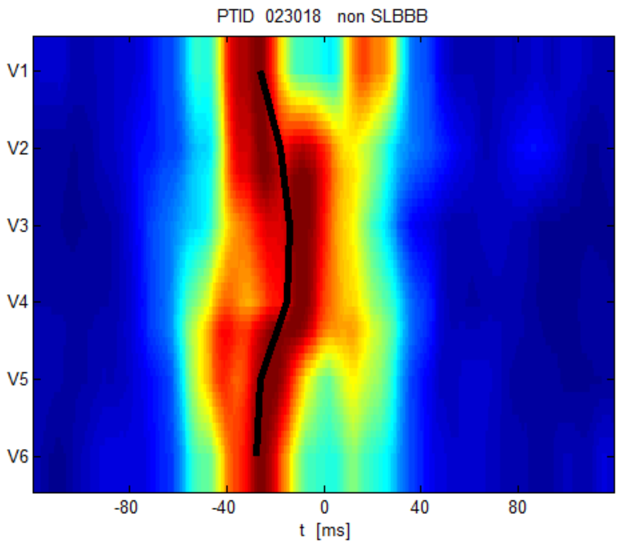
PTID 023018 non SLBBB
V1
V2
V3
V4
V5
V6
-80
-40
0
40
80
t [ms]

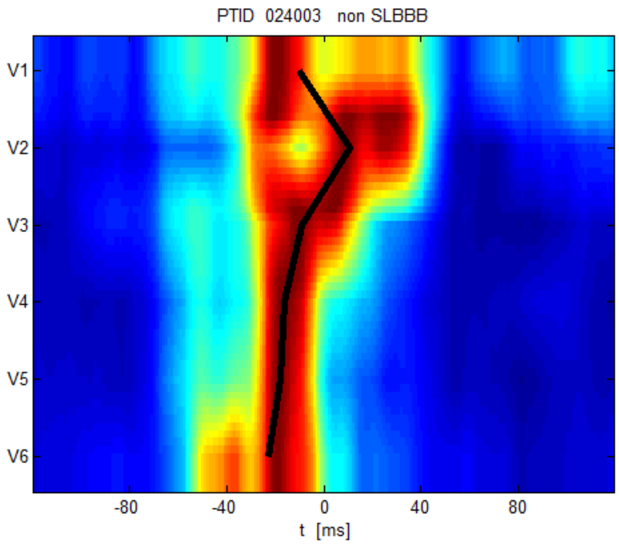
PTID 024003 non SLBBB
V1
V2
V3
V4
V5
V6
-80
-40
0
40
80
t [ms]

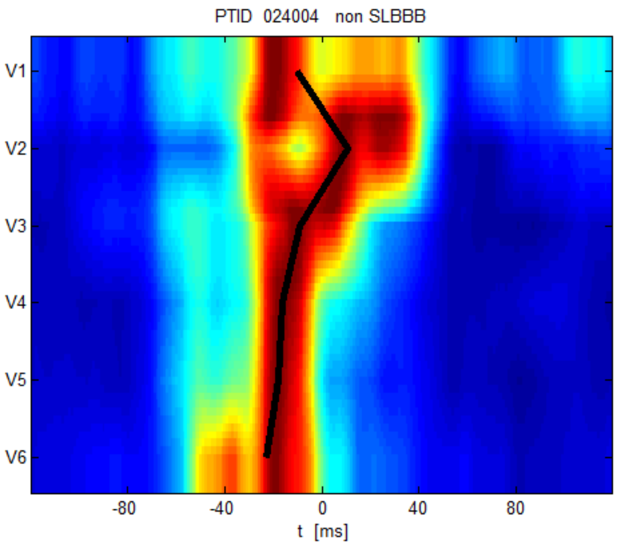
PTID 024004 non SLBBB
V1
V2
V3
V4
V5
V6
-80
-40
0
40
80
t [ms]

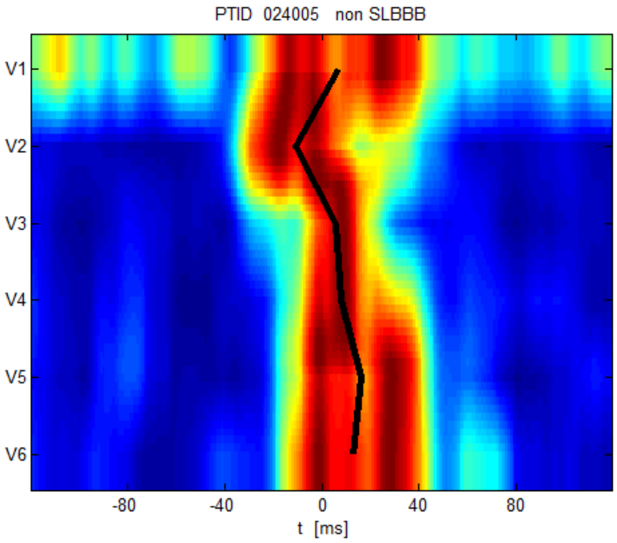
PTID 024005 non SLBBB
V1
V2
V3
V4
V5
V6
-80
-40
0
40
80
t [ms]

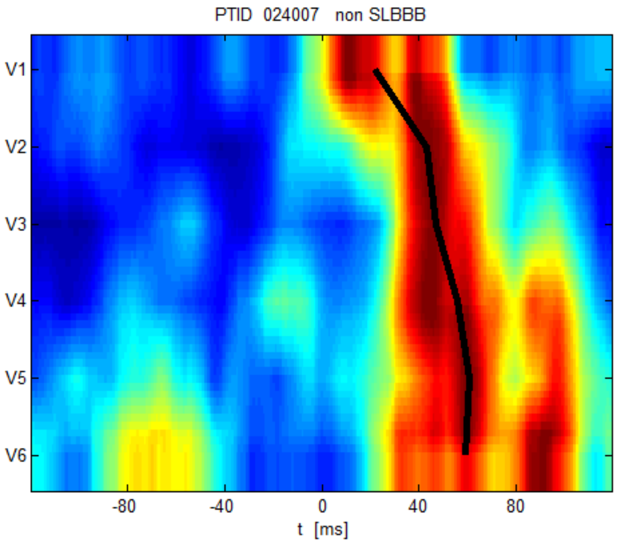
PTID 024007 non SLBBB
V1
V2
V3
V4
V5
V6
-80
-40
0
40
80
t [ms]

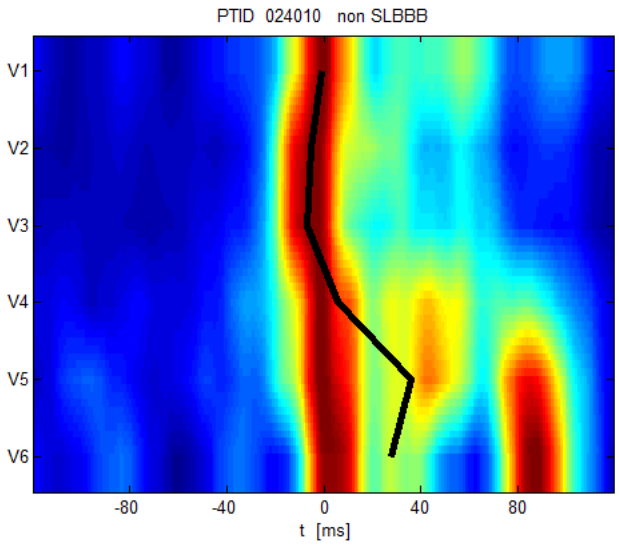
PTID 024010 non SLBBB
V1
V2
V3
V4
V5
V6
-80
-40
0
40
80
t [ms]

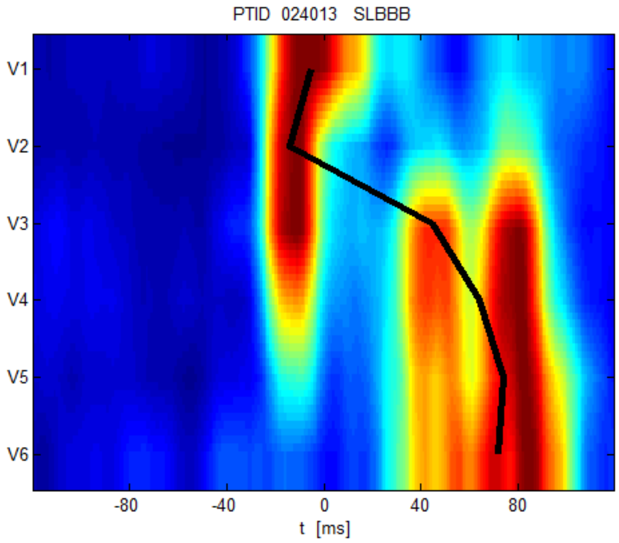
PTID 024013 SLBBB
V1
V2
V3
V4
V5
V6
-80
-40
0
40
80
t [ms]

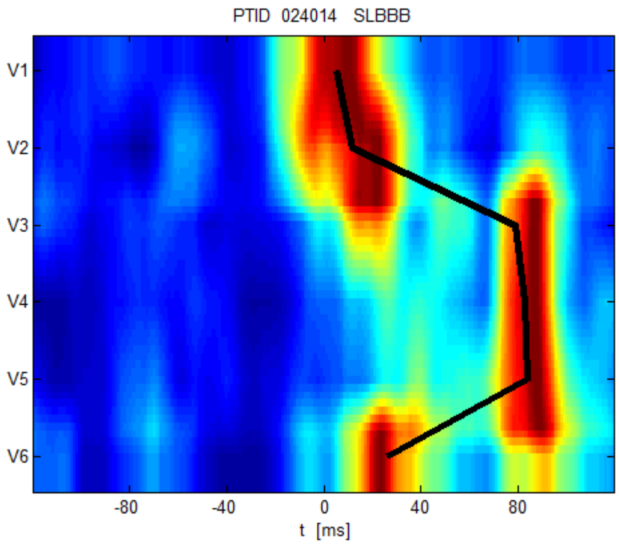
PTID 024014 SLBBB
V1
V2
V3
V4
V5
V6
-80
-40
0
40
80
t [ms]

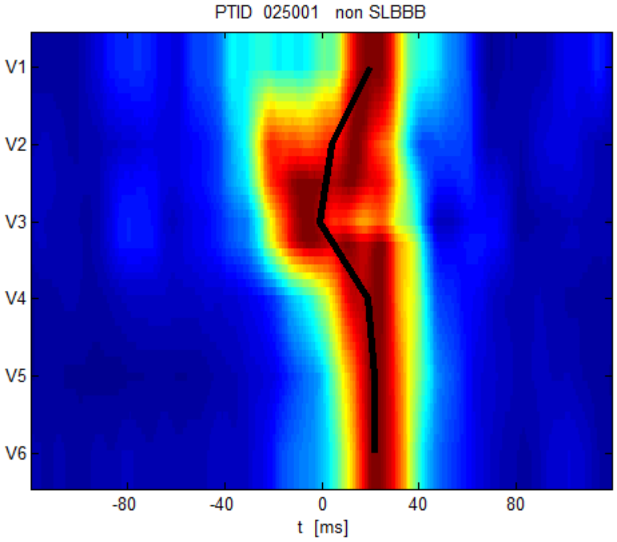
PTID 025001 non SLBBB
V1
V2
V3
V4
V5
V6
-80
-40
0
40
80
t [ms]

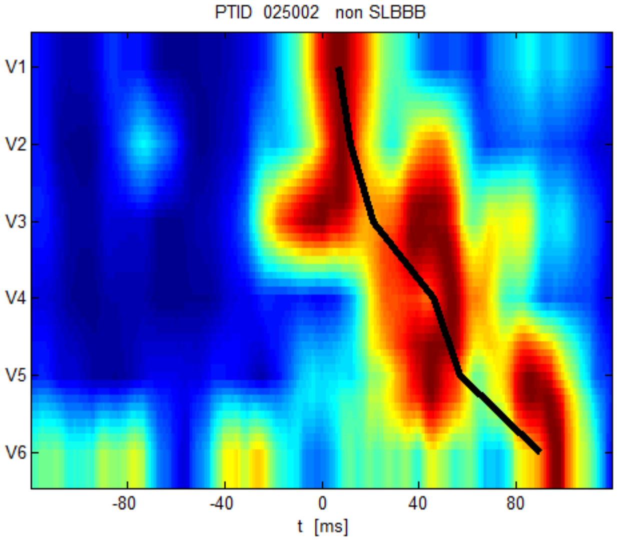
PTID 025002 non SLBBB
V1
V2
V3
V4
V5
V6
-80
-40
0
40
80
t [ms]

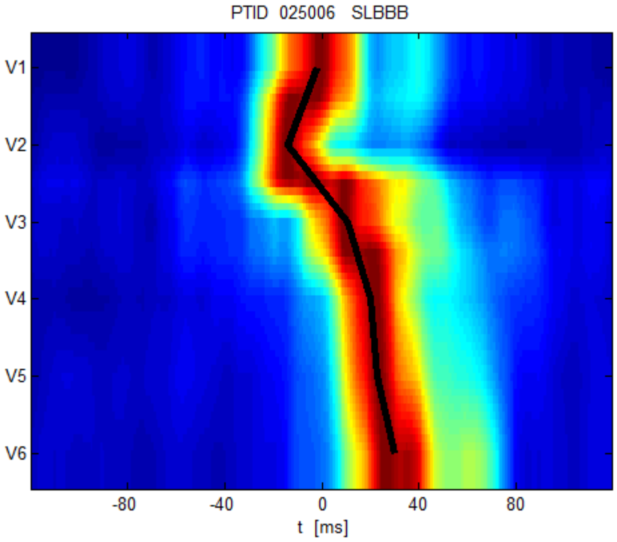
PTID 025006 SLBBB
V1
V2
V3
V4
V5
V6
-80
-40
0
40
80
t [ms]

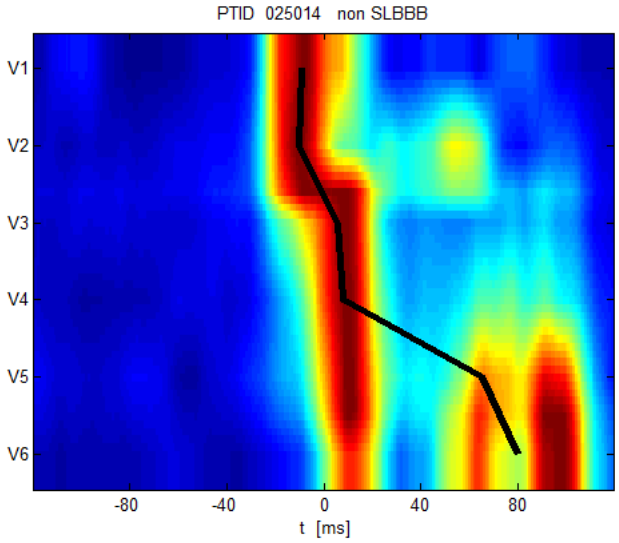
PTID 025014 non SLBBB
V1
V2
V3
V4
V5
V6
-80
-40
0
40
80
t [ms]

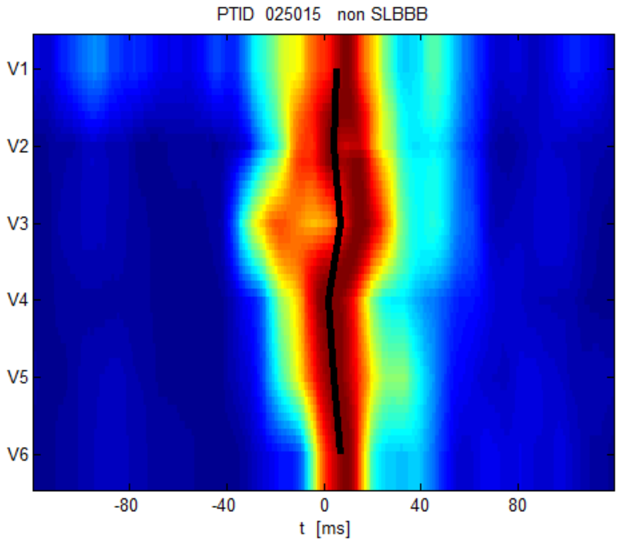
PTID 025015 non SLBBB
V1
V2
V3
V4
V5
V6
-80
-40
0
40
80
t [ms]

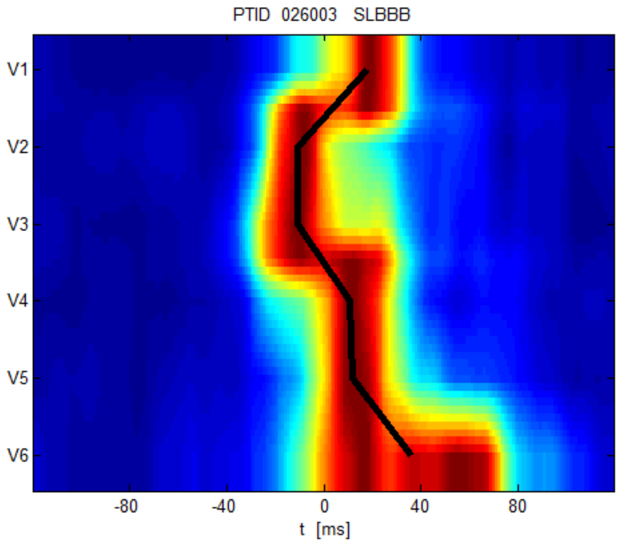
PTID 026003 SLBBB
V1
V2
V3
V4
V5
V6
-80
-40
0
40
80
t [ms]

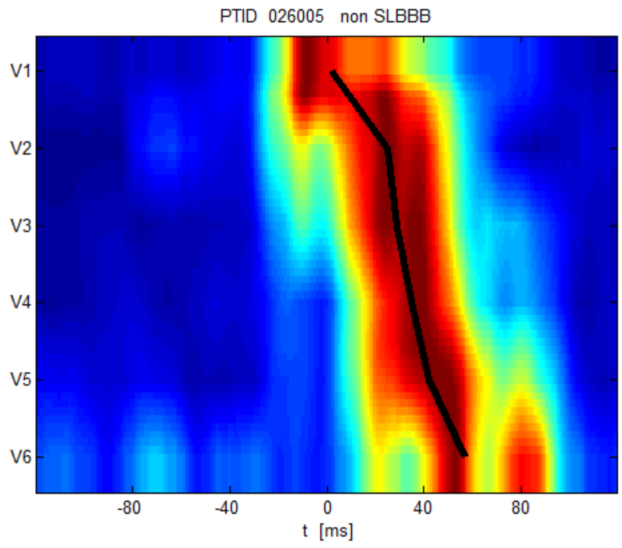
PTID 026005 non SLBBB
V1
V2
V3
V4
V5
V6
-80
-40
0
40
80
t [ms]

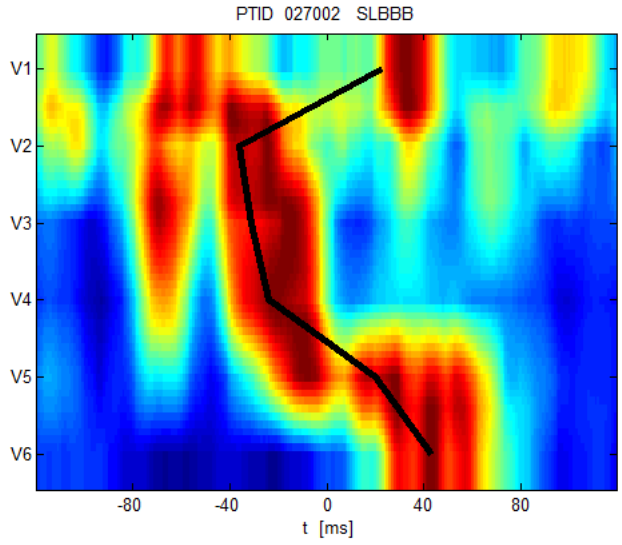
PTID 027002 SLBBB
V1
V2
V3
V4
V5
V6
-80
-40
0
40
80
t [ms]

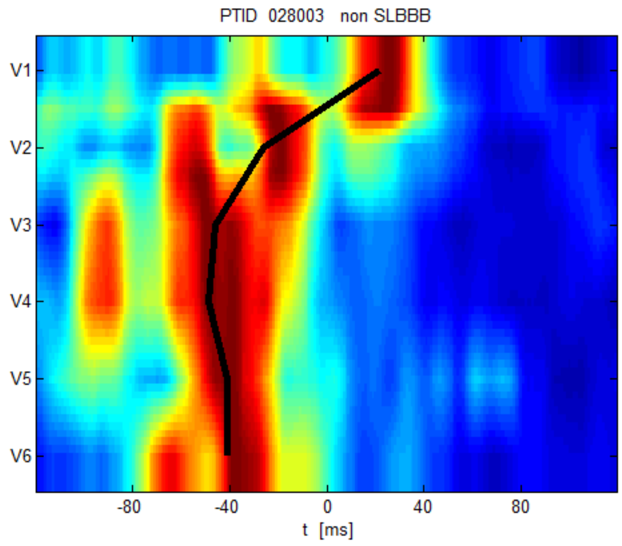
PTID 028003 non SLBBB
V1
V2
V3
V4
V5
V6
-80
-40
0
40
80
t [ms]

PTID 028007 SLBBB

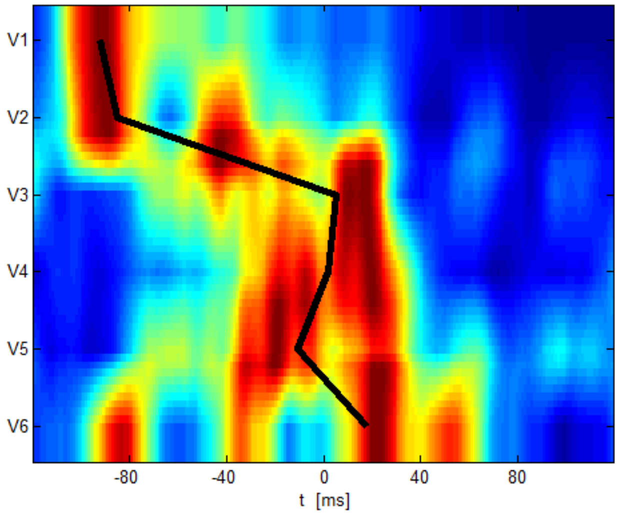

PTID 028015 non SLBBB

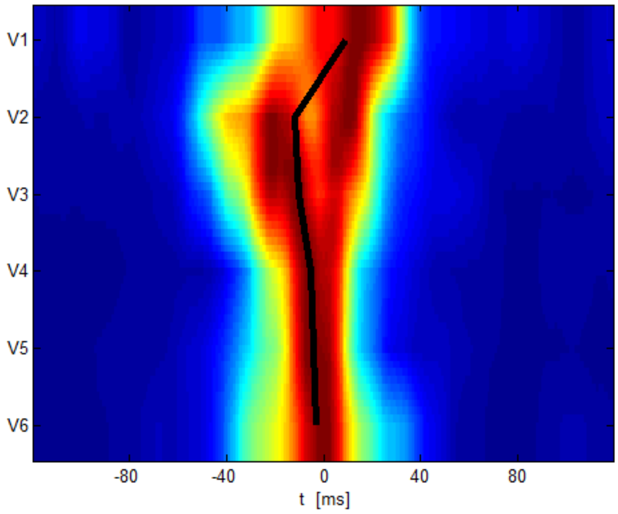

PTID 029002 non SLBBB

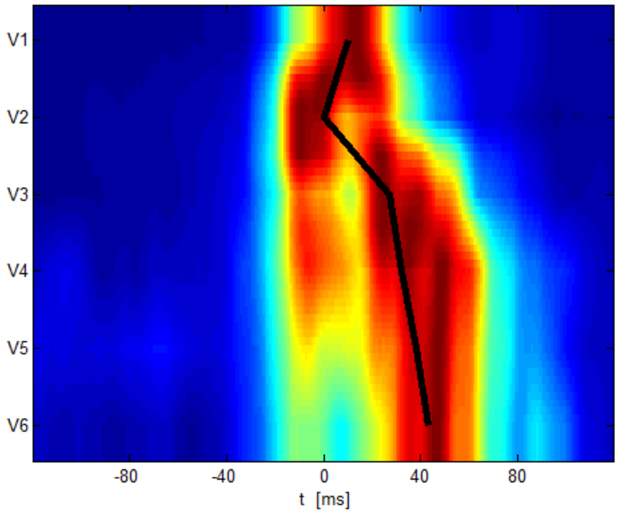

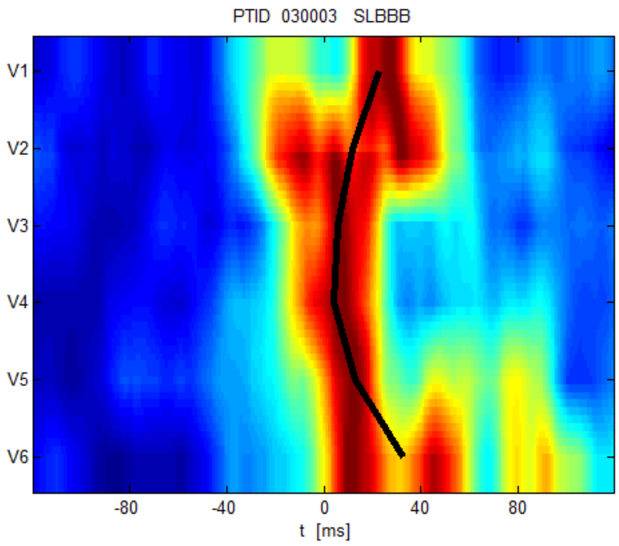
PTID 030003 SLBBB
V1
V2
V3
V4
V5
V6
-80
-40
0
40
80
t [ms]

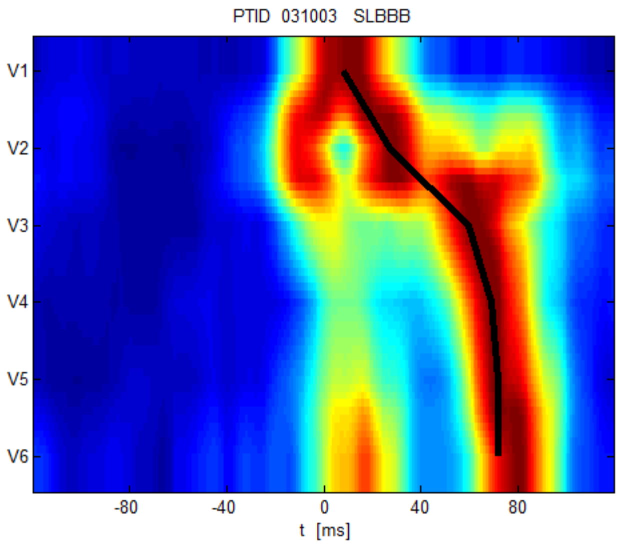
PTID 031003 SLBBB
V1
V2
V3
V4
V5
V6
-80
-40
0
40
80
t [ms]

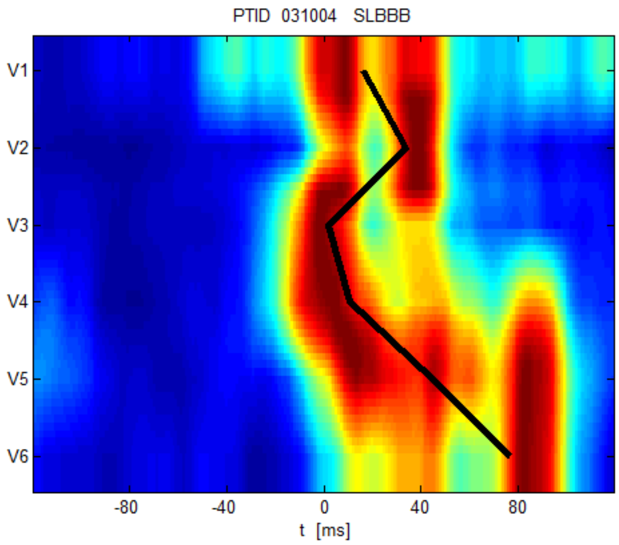
PTID 031004 SLBBB
V1
V2
V3
V4
V5
V6
-80
-40
0
40
80
t [ms]

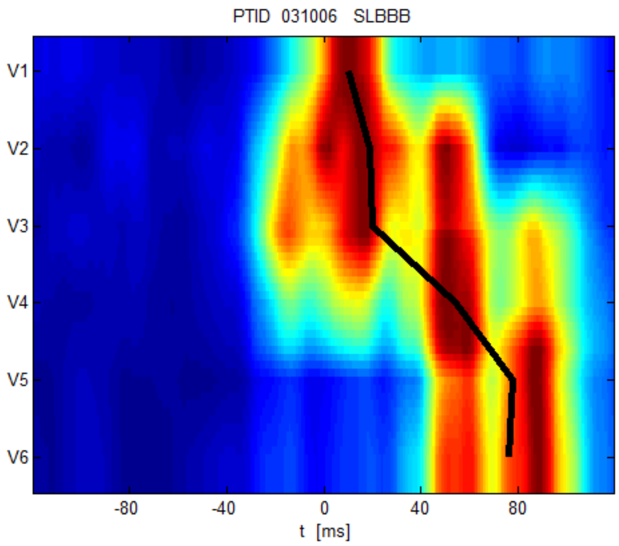
PTID 031006 SLBBB
V1
V2
V3
V4
V5
V6
-80
-40
0
40
80
t [ms]

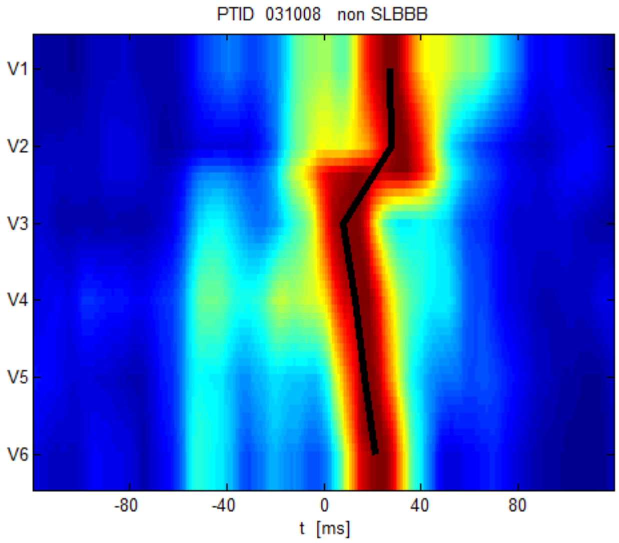
PTID 031008 non SLBBB
V1
V2
V3
V4
V5
V6
-80
-40
0
40
80
t [ms]

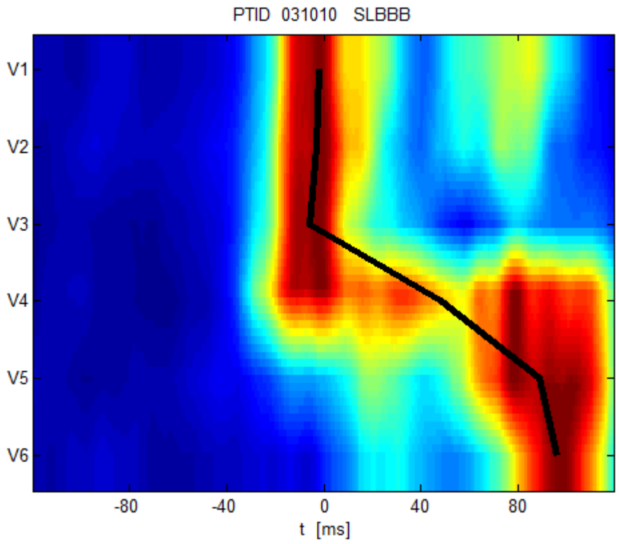
PTID 031010 SLBBB
V1
V2
V3
V4
V5
V6
-80
-40
0
40
80
t [ms]

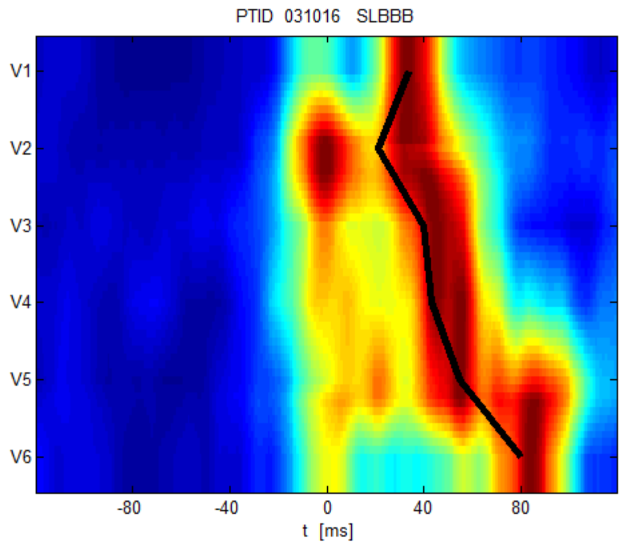
PTID 031016 SLBBB
V1
V2
V3
V4
V5
V6
-80
-40
0
40
80
t [ms]

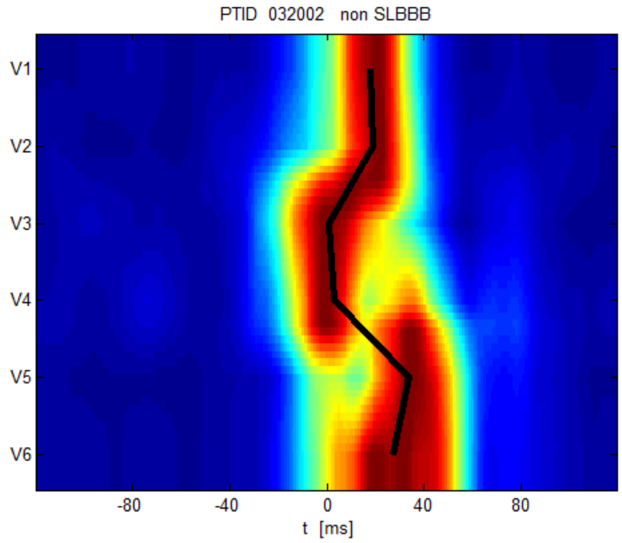
PTID 032002 non SLBBB
V1
V2
V3
V4
V5
V6
-80
-40
0
40
80
t [ms]

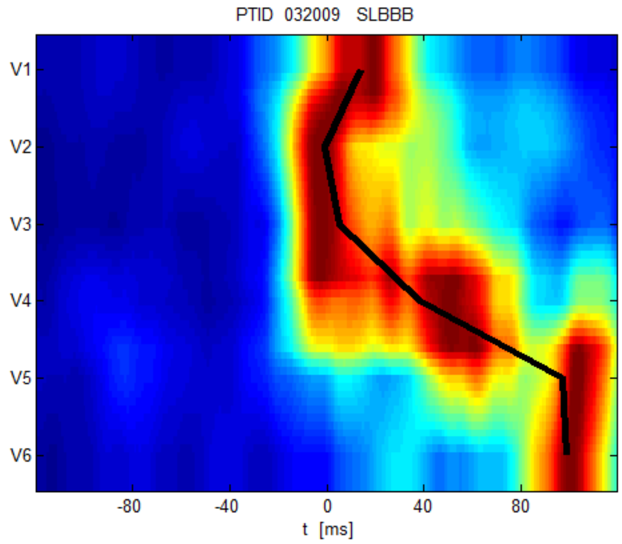
PTID 032009 SLBBB
V1
V2
V3
V4
V5
V6
-80
-40
0
40
80
t [ms]

PTID 032010 SLBBB

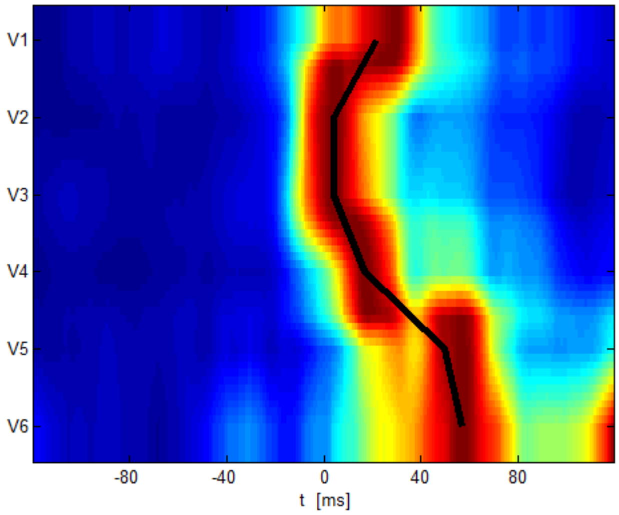

PTID 033002 non SLBBB

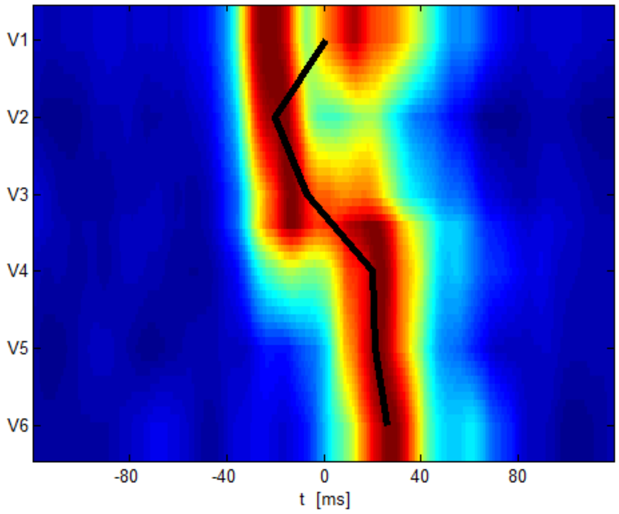

PTID 033003 non SLBBB

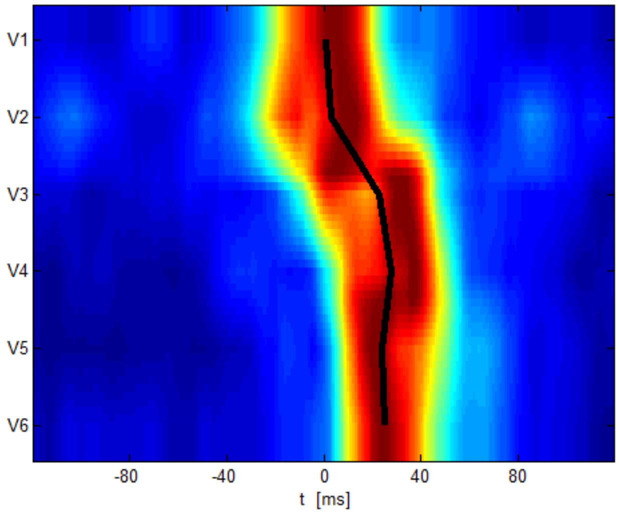

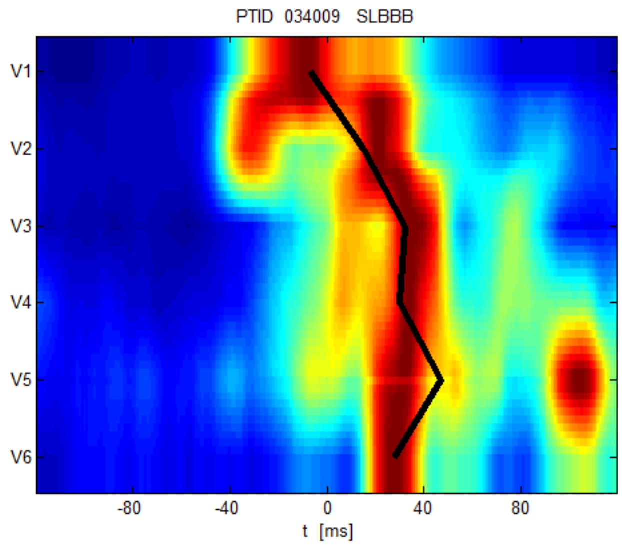
PTID 034009 SLBBB
V1
V2
V3
V4
V5
V6
-80
-40
0
40
80
t [ms]

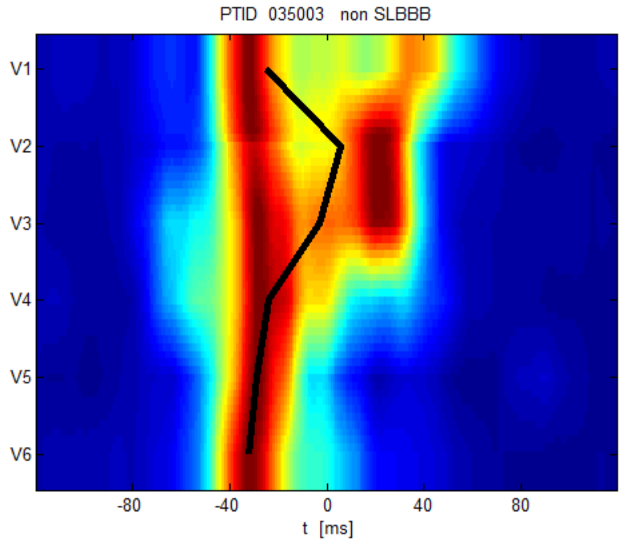
PTID 035003 non SLBBB
V1
V2
V3
V4
V5
V6
-80
-40
0
40
80
t [ms]

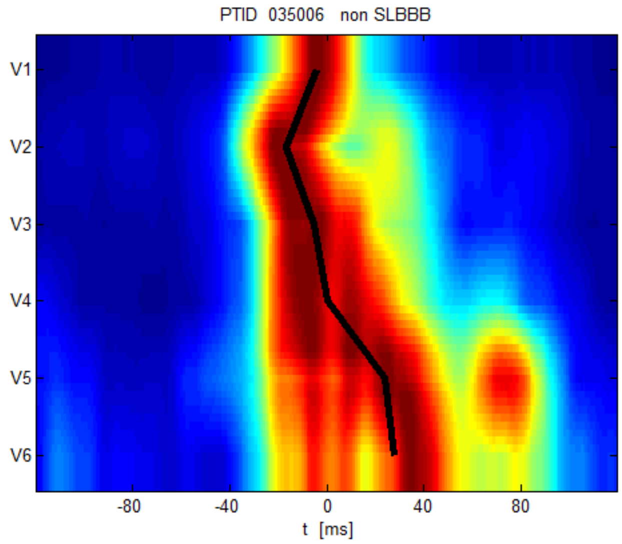
PTID 035006 non SLBBB
V1
V2
V3
V4
V5
V6
-80
-40
0
40
80
t [ms]

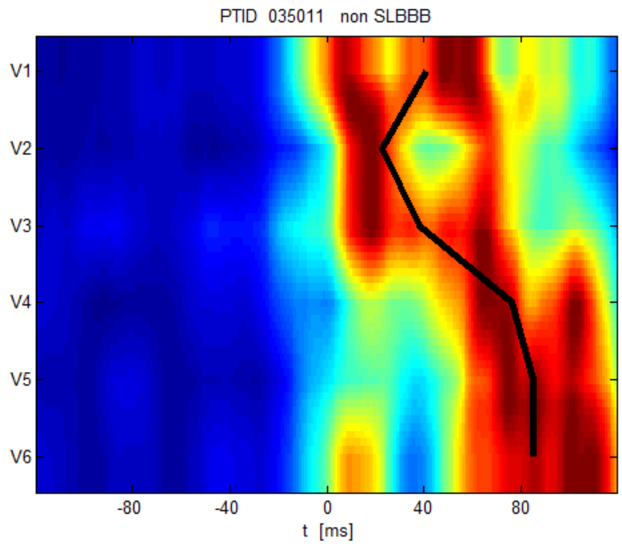
PTID 035011 non SLBBB
V1
V2
V3
V4
V5
V6
-80
-40
0
40
80
t [ms]

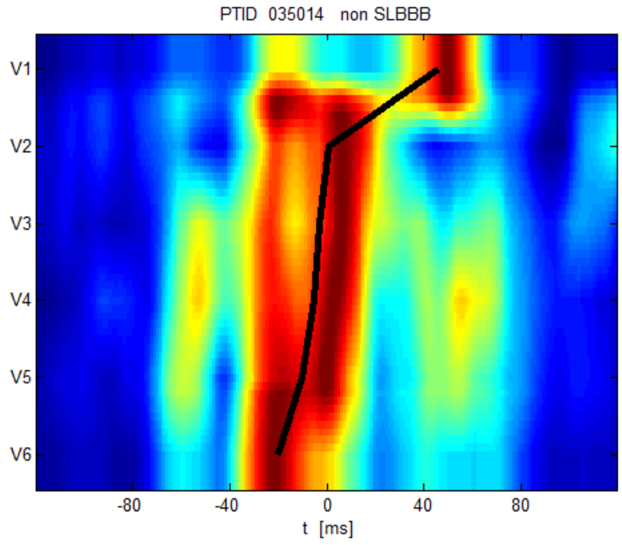
PTID 035014 non SLBBB
V1
V2
V3
V4
V5
V6
-80
-40
0
40
80
t [ms]

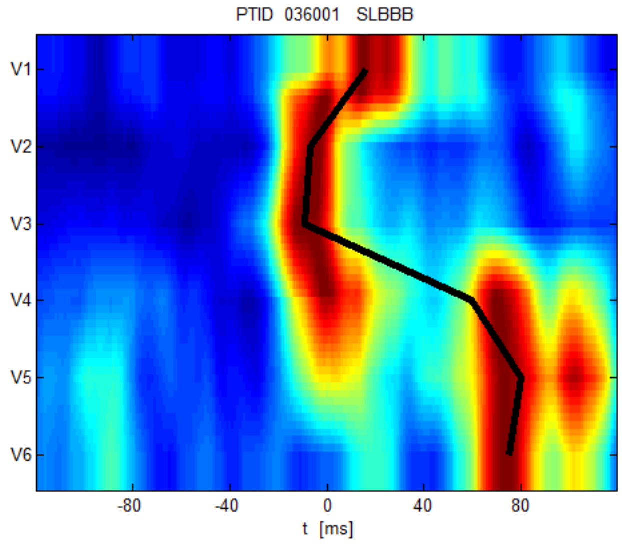
PTID 036001 SLBBB
V1
V2
V3
V4
V5
V6
-80
-40
0
40
80
t [ms]

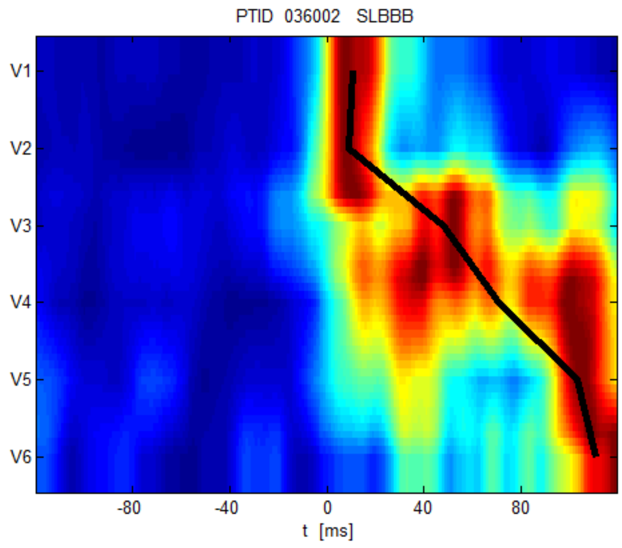
PTID 036002 SLBBB
V1
V2
V3
V4
V5
V6
-80
-40
0
40
80
t [ms]

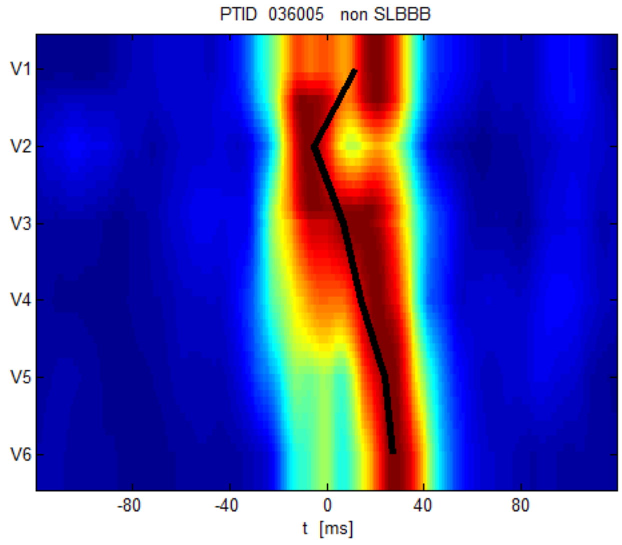
PTID 036005 non SLBBB
V1
V2
V3
V4
V5
V6
-80
-40
0
40
80
t [ms]

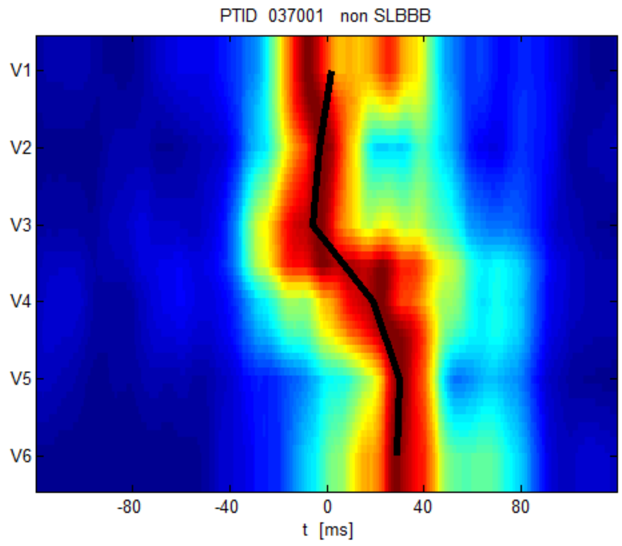
PTID 037001 non SLBBB
V1
V2
V3
V4
V5
V6
-80
-40
0
40
80
t [ms]

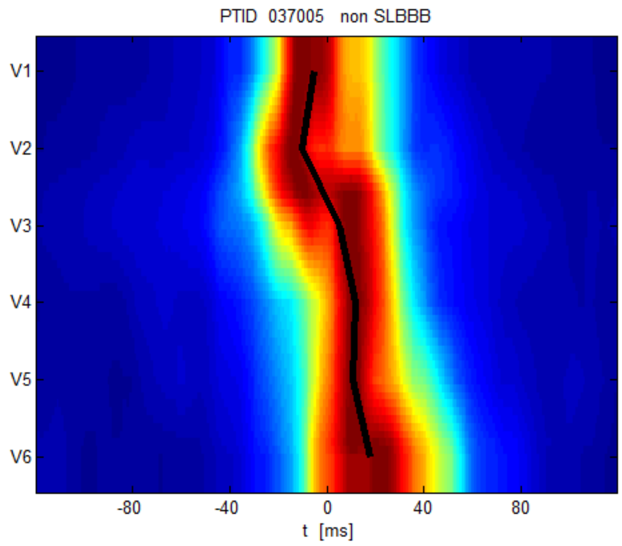
PTID 037005 non SLBBB
V1
V2
V3
V4
V5
V6
-80
-40
0
40
80
t [ms]

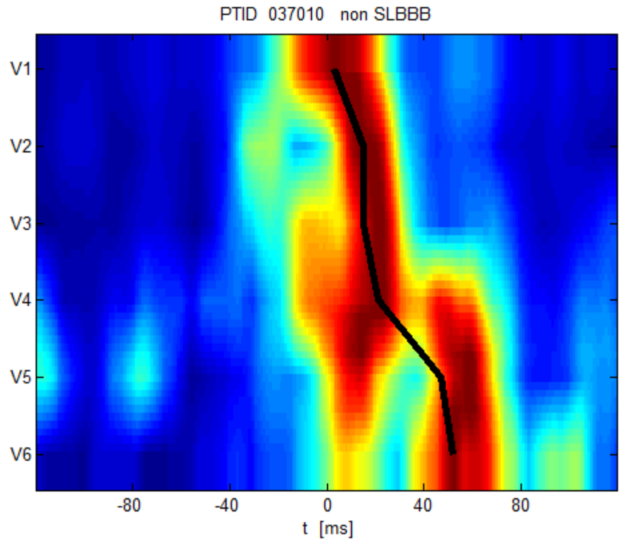
PTID 037010 non SLBBB
V1
V2
V3
V4
V5
V6
-80
-40
0
40
80
t [ms]

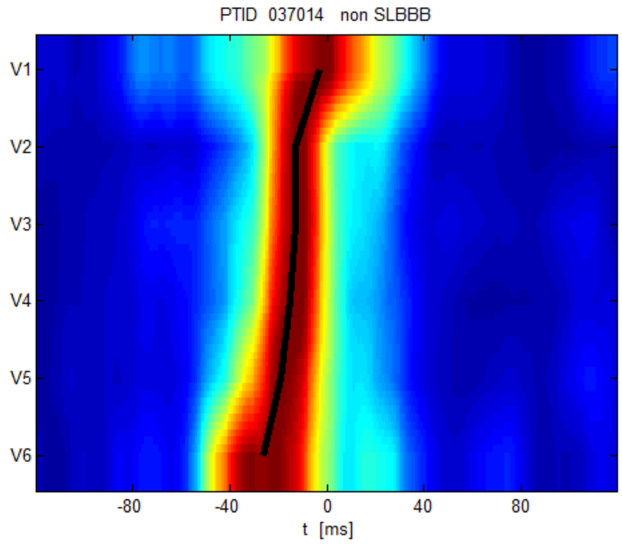
PTID 037014 non SLBBB
V1
V2
V3
V4
V5
V6
-80
-40
0
40
80
t [ms]

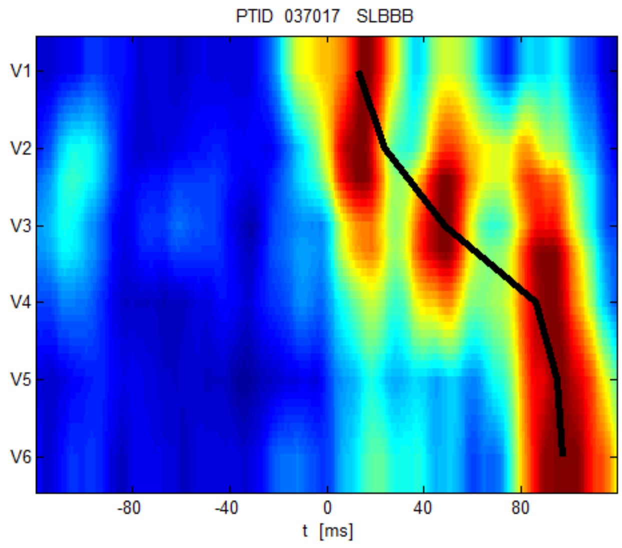
PTID 037017 SLBBB
V1
V2
V3
V4
V5
V6
-80
-40
0
40
80
t [ms]

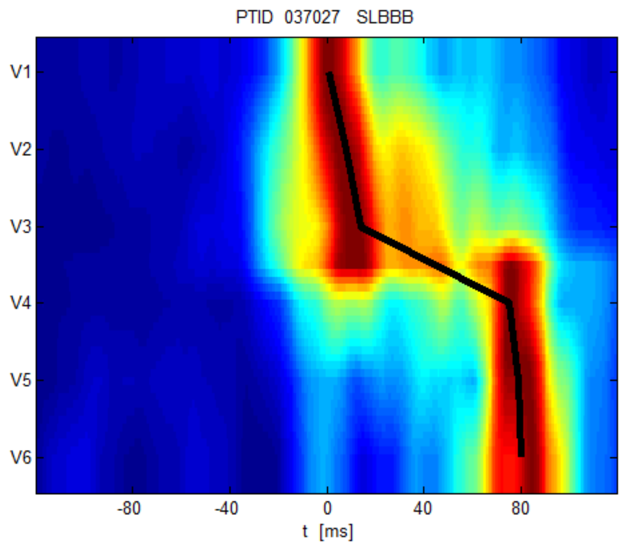
PTID 037027 SLBBB
V1
V2
V3
V4
V5
V6
-80
-40
0
40
80
t [ms]

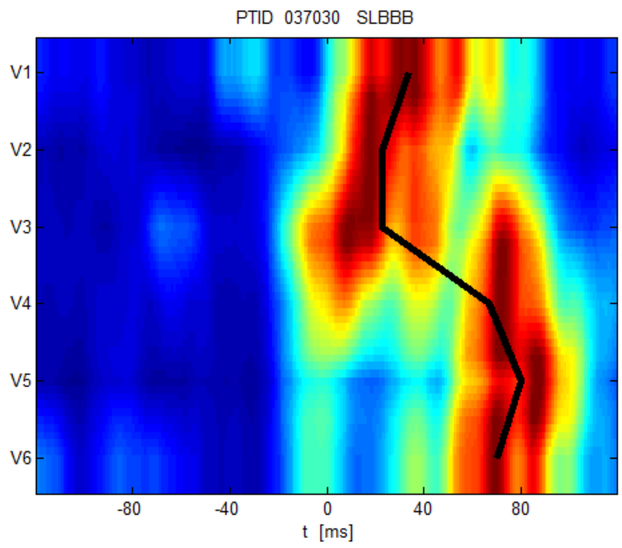
PTID 037030 SLBBB
V1
V2
V3
V4
V5
V6
-80
-40
0
40
80
t [ms]

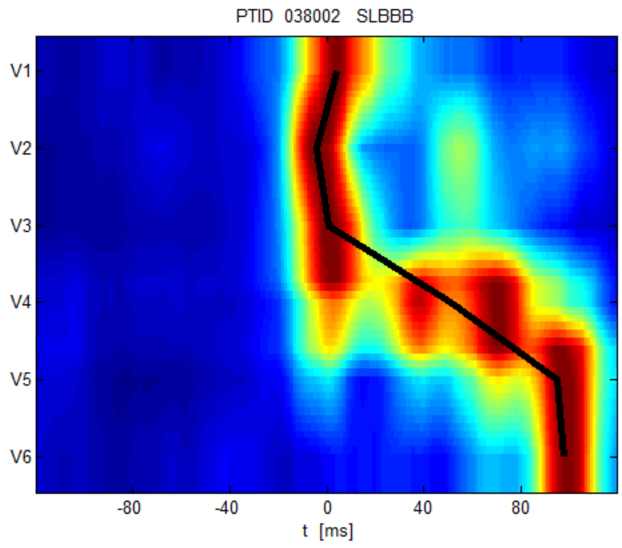
PTID 038002 SLBBB
V1
V2
V3
V4
V5
V6
-80
-40
0
40
80
t [ms]

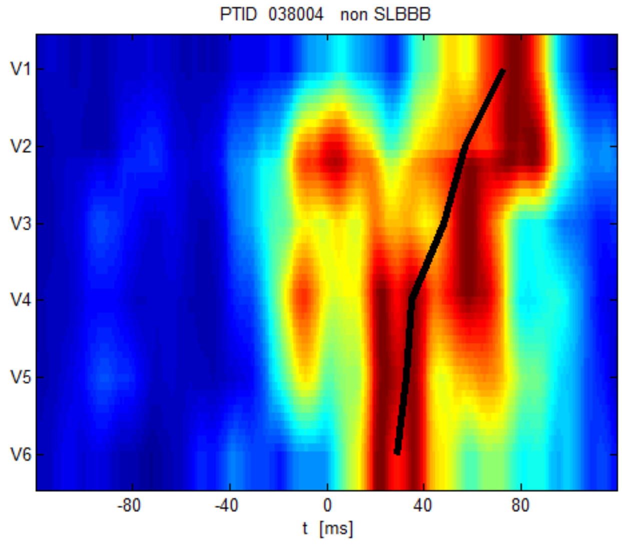
PTID 038004 non SLBBB
V1
V2
V3
V4
V5
V6
-80
-40
0
40
80
t [ms]

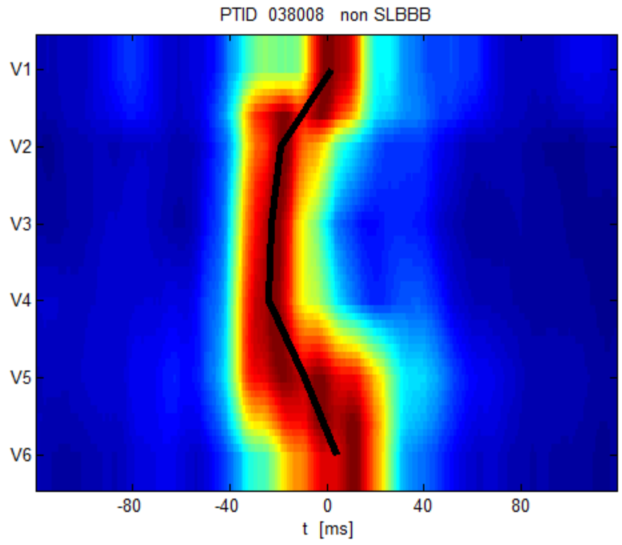
PTID 038008 non SLBBB
V1
V2
V3
V4
V5
V6
-80
-40
0
40
80
t [ms]

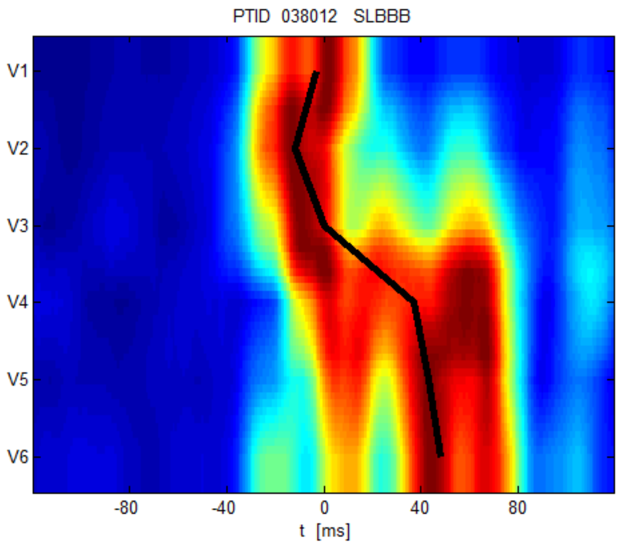
PTID 038012 SLBBB
V1
V2
V3
V4
V5
V6
-80
-40
0
40
80
t [ms]

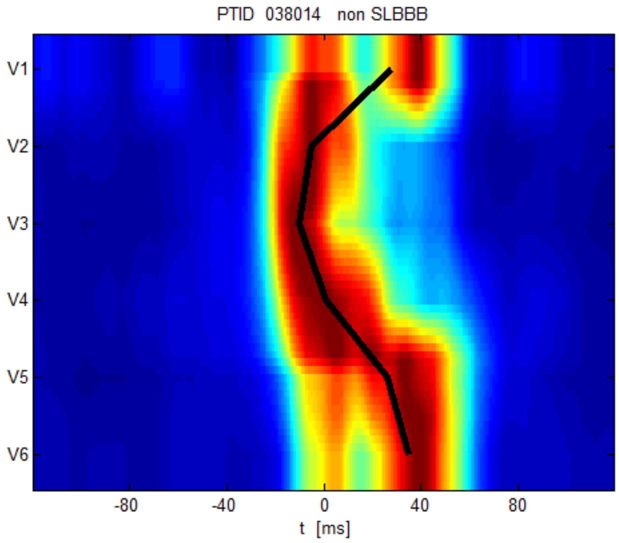
PTID 038014 non SLBBB
V1
V2
V3
V4
V5
V6
-80
-40
0
40
80
t [ms]

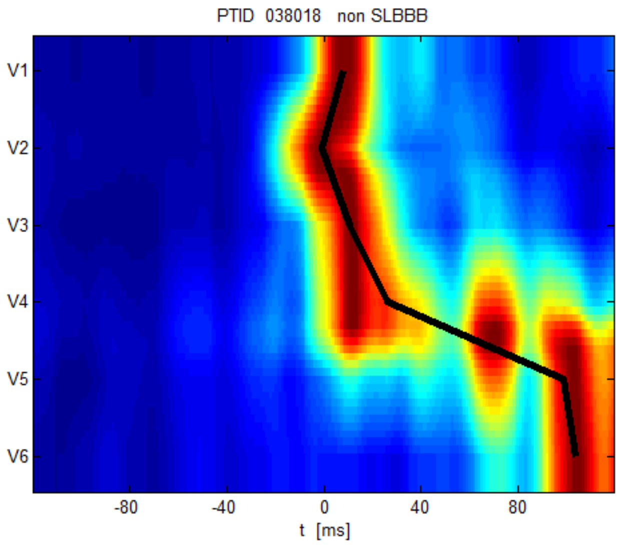
PTID 038018 non SLBBB
V1
V2
V3
V4
V5
V6
-80
-40
0
40
80
t [ms]

PTID 039001 non SLBBB

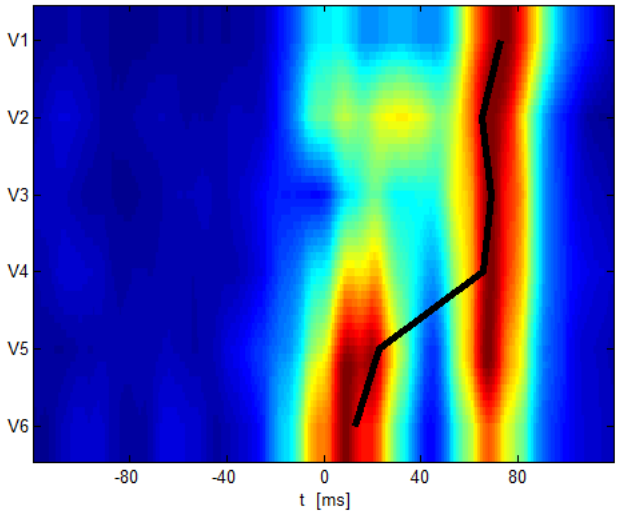

PTID 039003 SLBBB

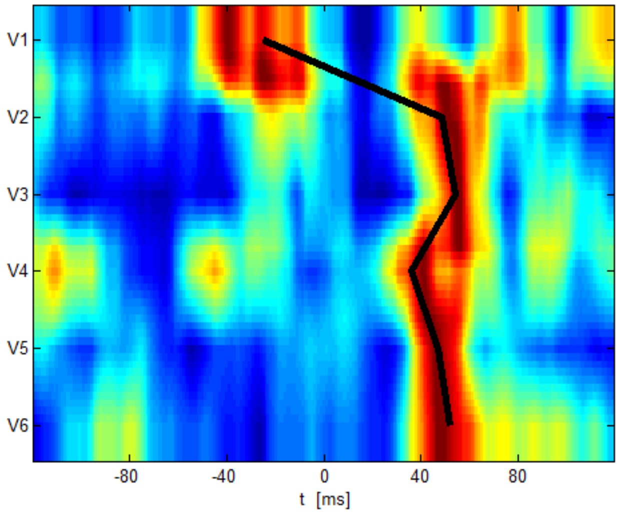

PTID 039006 SLBBB

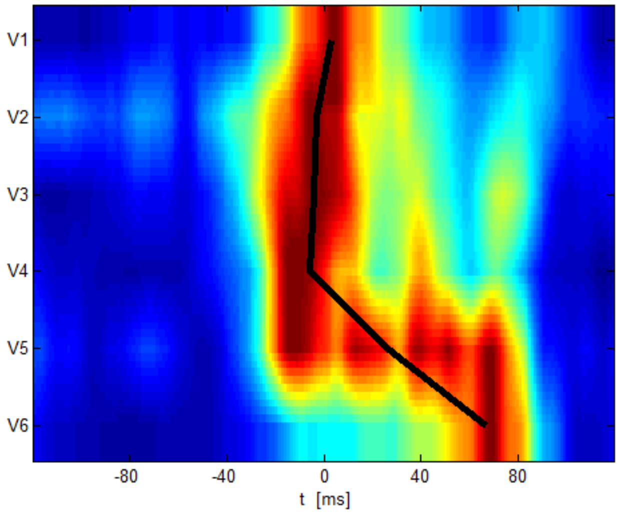

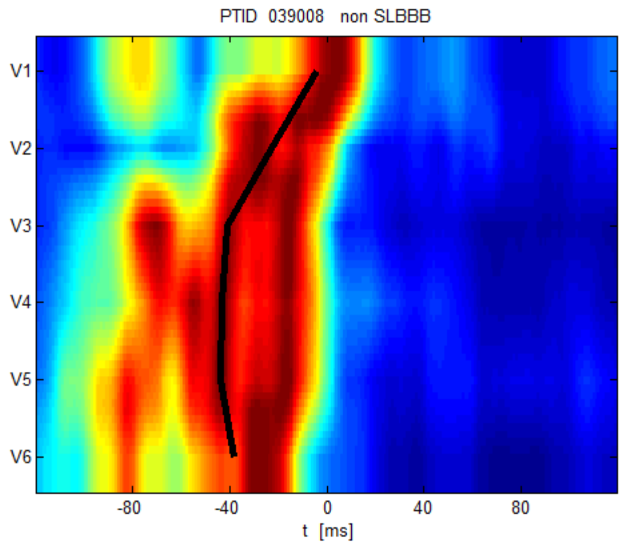
PTID 039008 non SLBBB
V1
V2
V3
V4
V5
V6
-80
-40
0
40
80
t [ms]

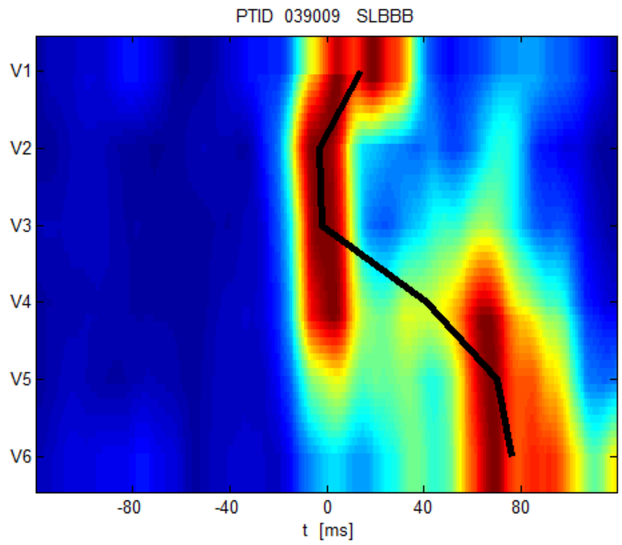
PTID 039009 SLBBB
V1
V2
V3
V4
V5
V6
-80
-40
0
40
80
t [ms]

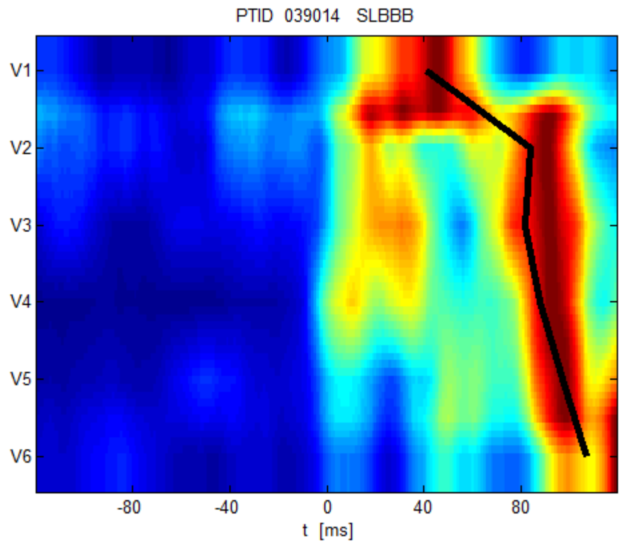
PTID 039014 SLBBB
V1
V2
V3
V4
V5
V6
-80
-40
0
40
80
t [ms]

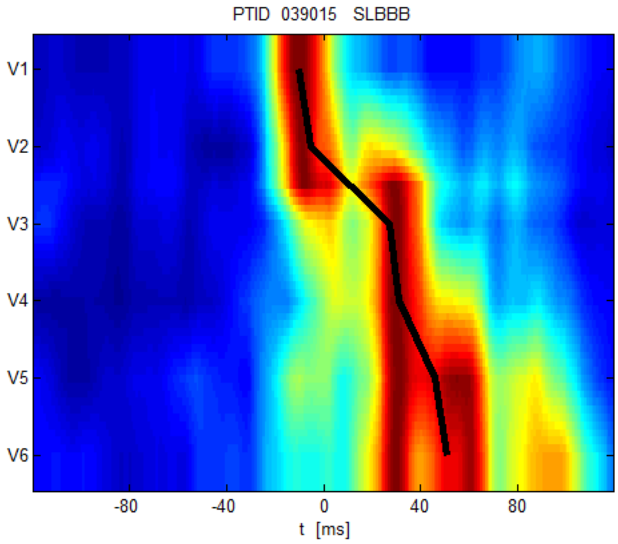
PTID 039015 SLBBB
V1
V2
V3
V4
V5
V6
-80
-40
0
40
80
t [ms]

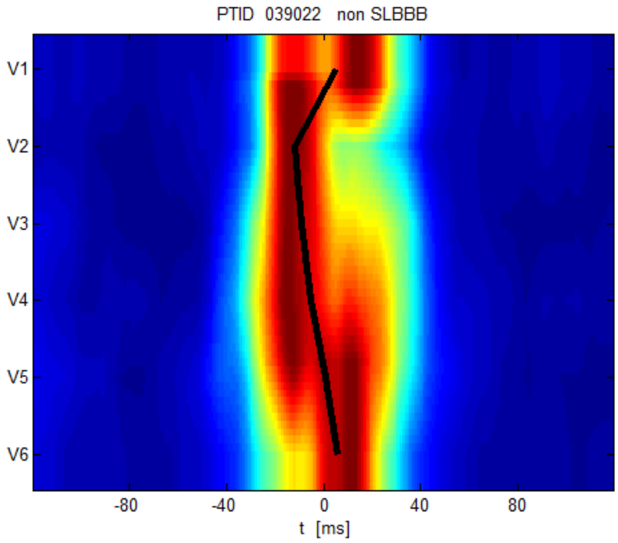
PTID 039022 non SLBBB
V1
V2
V3
V4
V5
V6
-80
-40
0
40
80
t [ms]

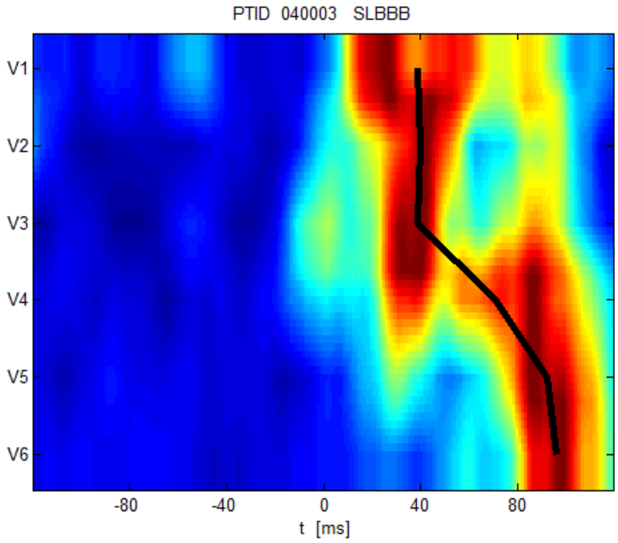
PTID 040003 SLBBB
V1
V2
V3
V4
V5
V6
-80
-40
0
40
80
t [ms]

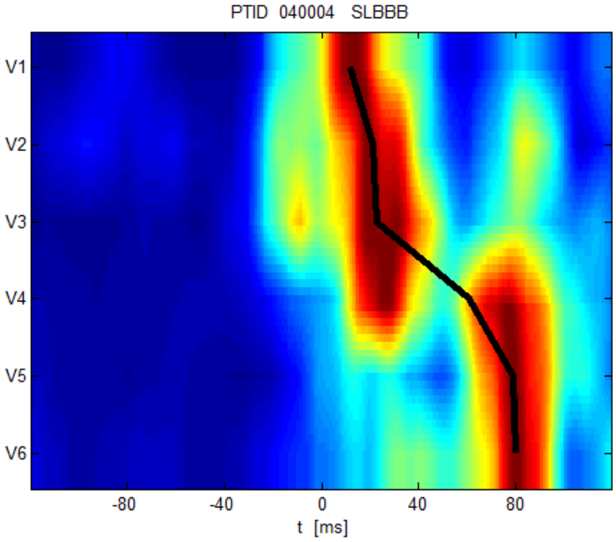
PTID 040004 SLBBB
V1
V2
V3
V4
V5
V6
-80
-40
0
40
80
t [ms]

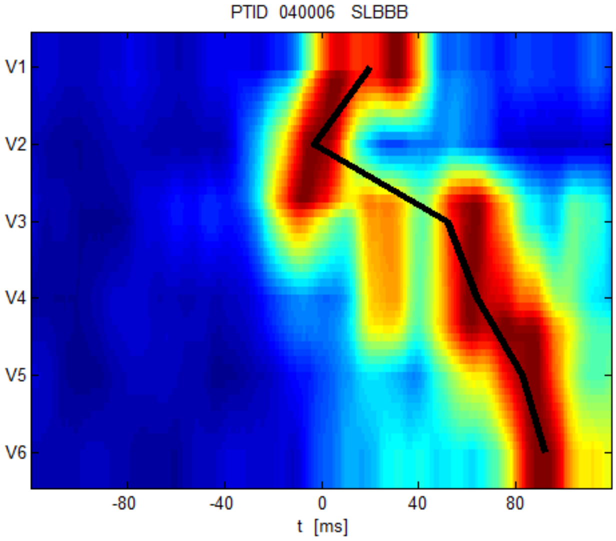
PTID 040006 SLBBB
V1
V2
V3
V4
V5
V6
-80
-40
0
40
80
t [ms]

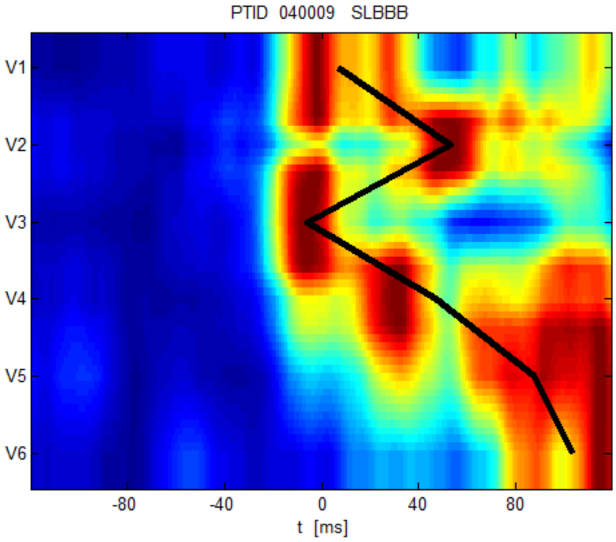
PTID 040009 SLBBB
V1
V2
V3
V4
V5
V6
-80
-40
0
40
80
t [ms]

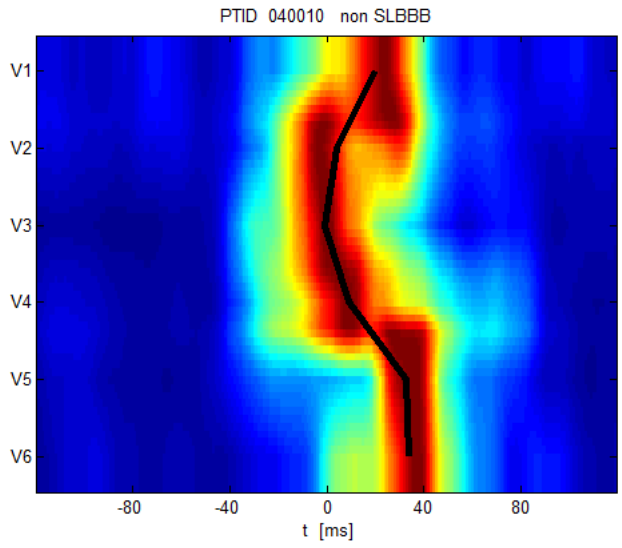
PTID 040010 non SLBBB
V1
V2
V3
V4
V5
V6
-80
-40
0
40
80
t [ms]

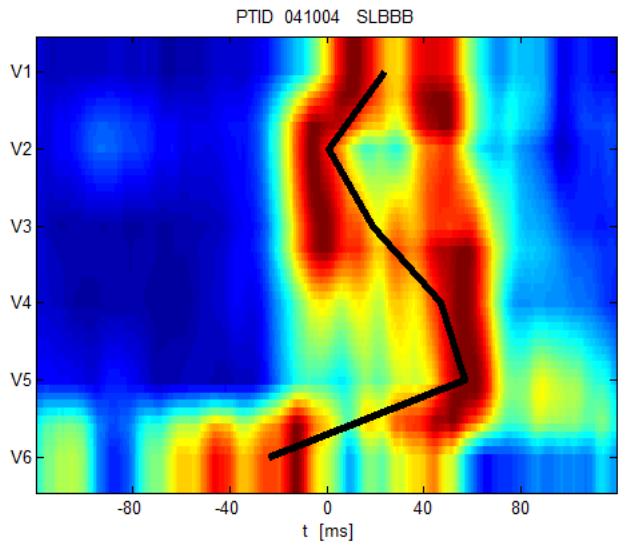
PTID 041004 SLBBB
V1
V2
V3
V4
V5
V6
-80
-40
0
40
80
t [ms]

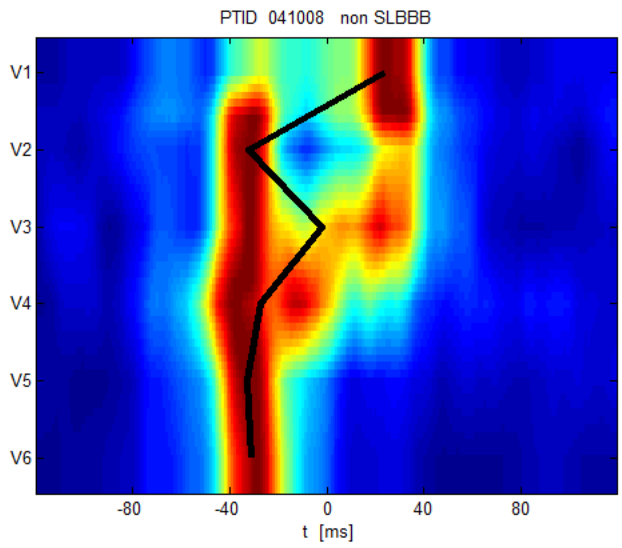
PTID 041008 non SLBBB
V1
V2
V3
V4
V5
V6
-80
-40
0
40
80
t [ms]

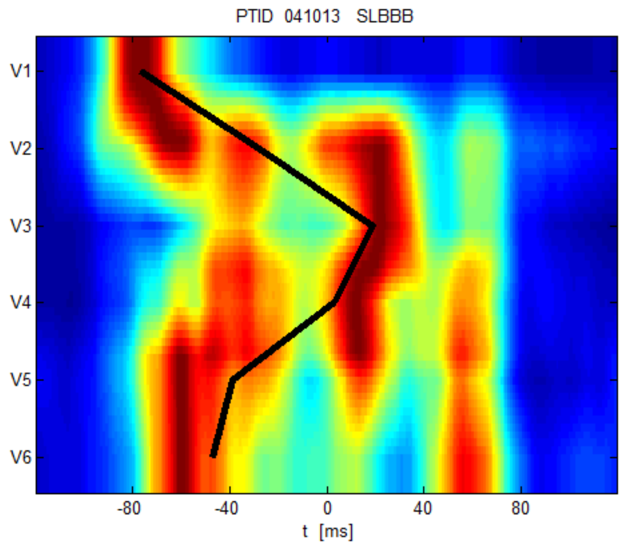

PTID 041013 SLBBB
V1
V2
V3
V4
V5
V6
-80
-40
0
40
80
t [ms]

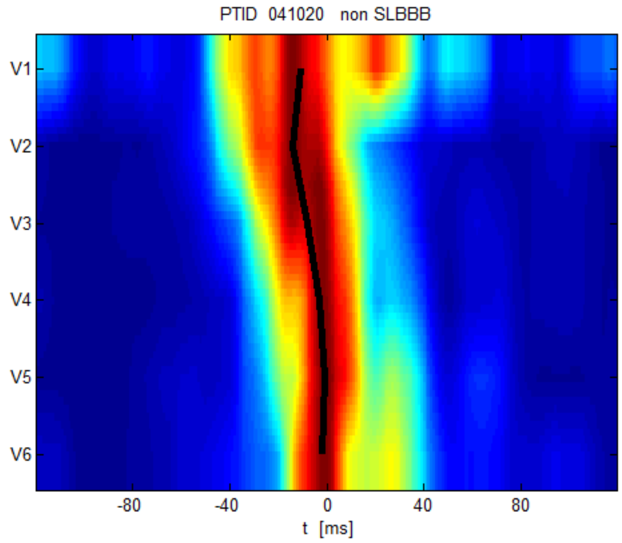

PTID 041020 non SLBBB
V1
V2
V3
V4
V5
V6
-80
-40
0
40
80
t [ms]

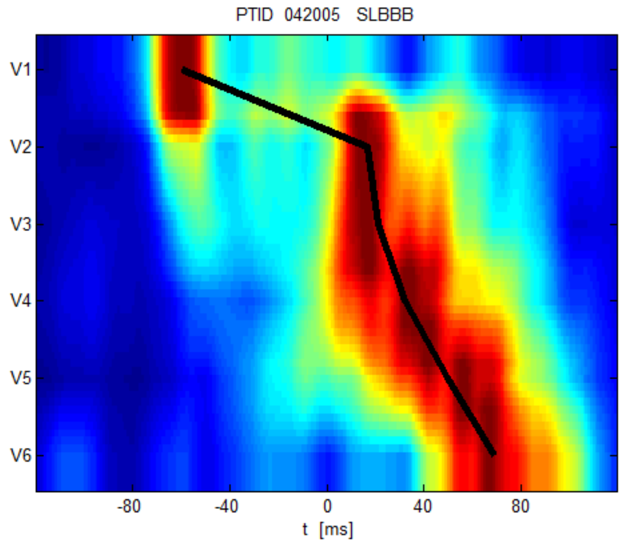

PTID 042005 SLBBB
V1
V2
V3
V4
V5
V6
-80
-40
0
40
80
t [ms]

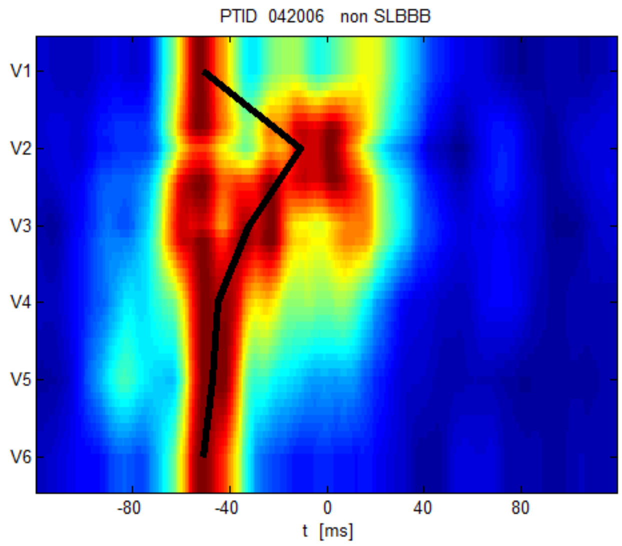
PTID 042006 non SLBBB
V1
V2
V3
V4
V5
V6
-80
-40
0
40
80
t [ms]

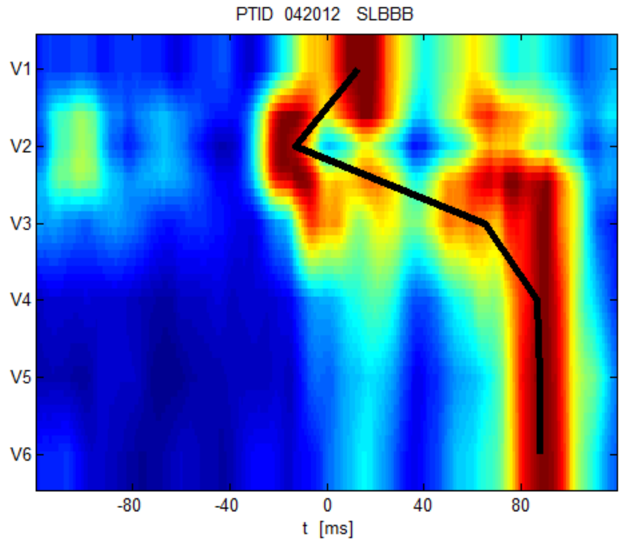
PTID 042012 SLBBB
V1
V2
V3
V4
V5
V6
-80
-40
0
40
80
t [ms]

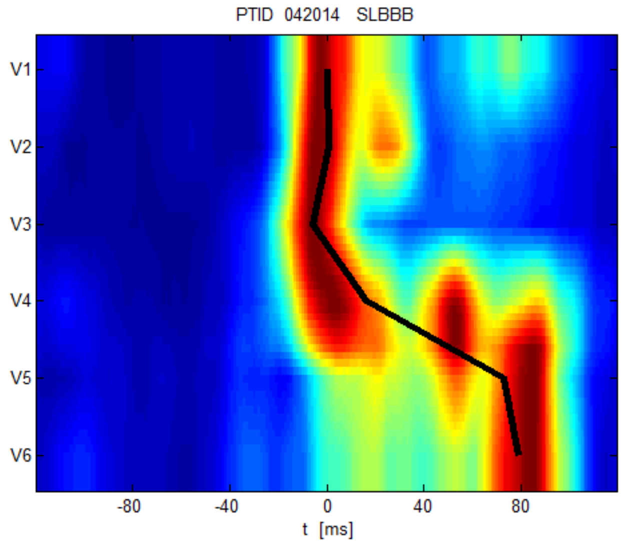
PTID 042014 SLBBB
V1
V2
V3
V4
V5
V6
-80
-40
0
40
80
t [ms]

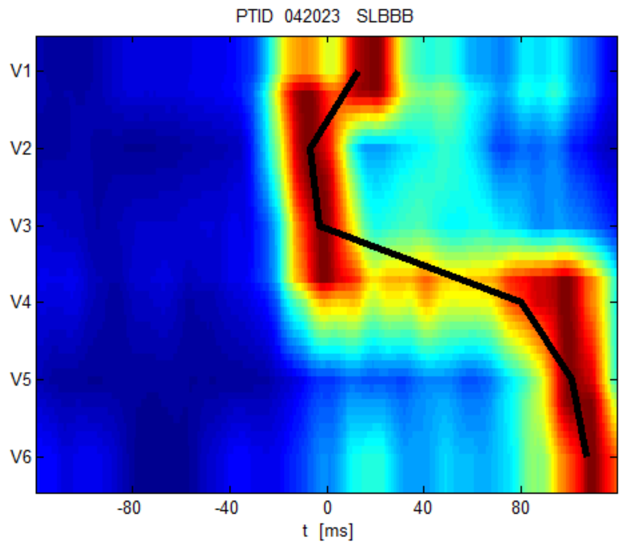
PTID 042023 SLBBB
V1
V2
V3
V4
V5
V6
-80
-40
0
40
80
t [ms]

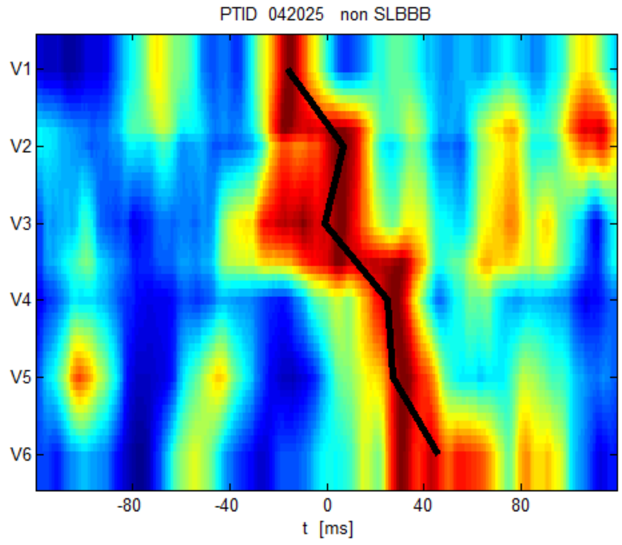
PTID 042025 non SLBBB
V1
V2
V3
V4
V5
V6
-80
-40
0
40
80
t [ms]

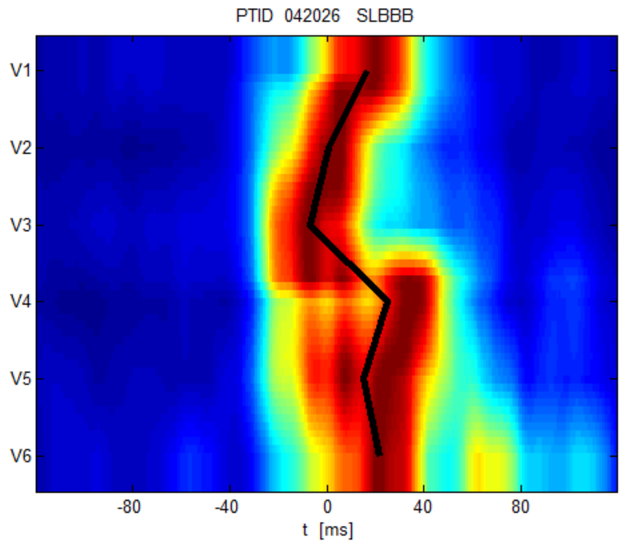
PTID 042026 SLBBB
V1
V2
V3
V4
V5
V6
-80
-40
0
40
80
t [ms]

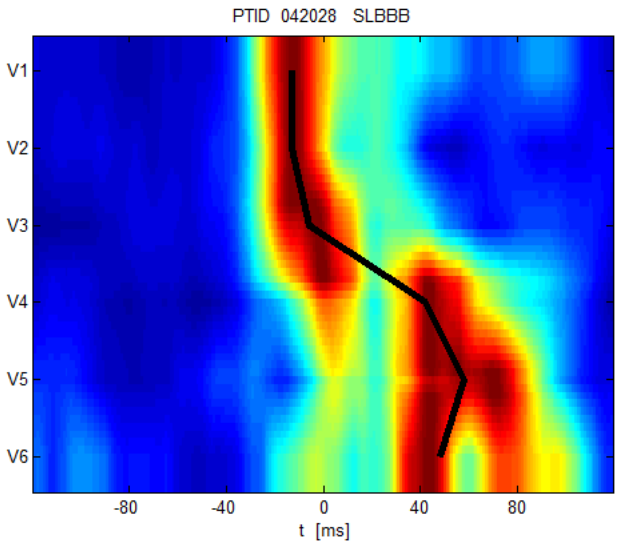
PTID 042028 SLBBB
V1
V2
V3
V4
V5
V6
-80
-40
0
40
80
t [ms]

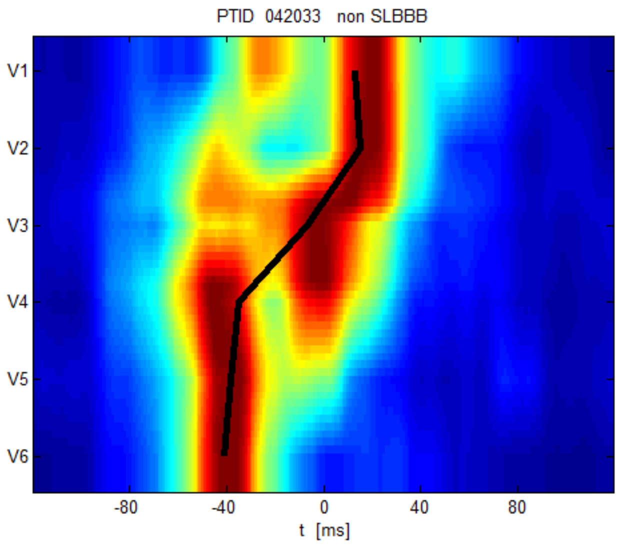
PTID 042033 non SLBBB
V1
V2
V3
V4
V5
V6
-80
-40
0
40
80
t [ms]

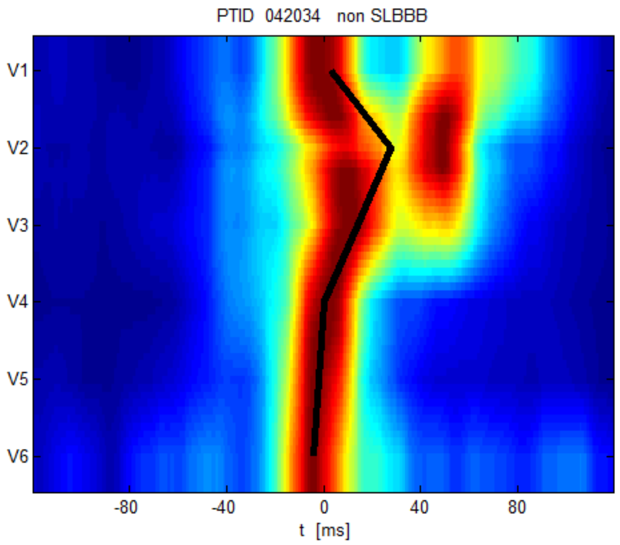
PTID 042034 non SLBBB
V1
V2
V3
V4
V5
V6
-80
-40
0
40
80
t [ms]

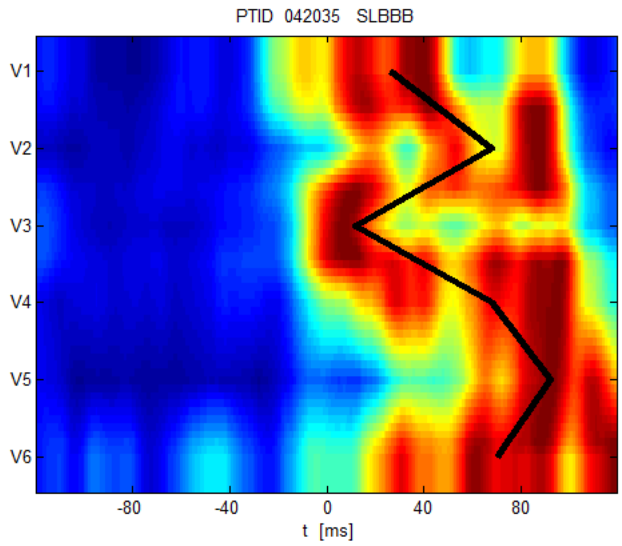

PTID 042035 SLBBB
V1
V2
V3
V4
V5
V6
-80
-40
0
40
80
t [ms]

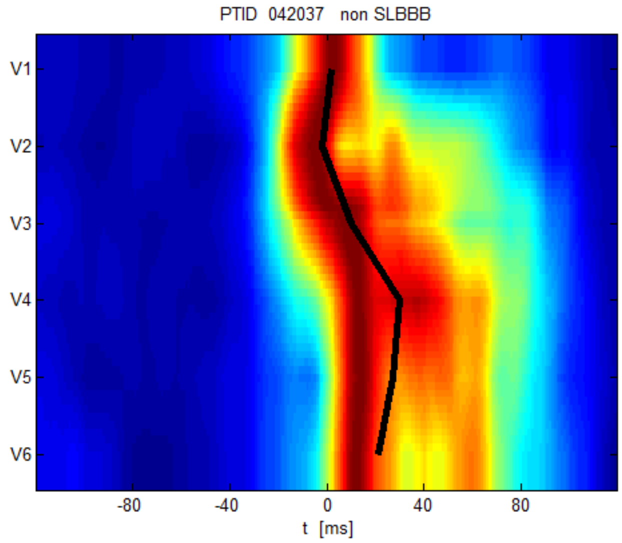

PTID 042037 non SLBBB
V1
V2
V3
V4
V5
V6
-80
-40
0
40
80
t [ms]

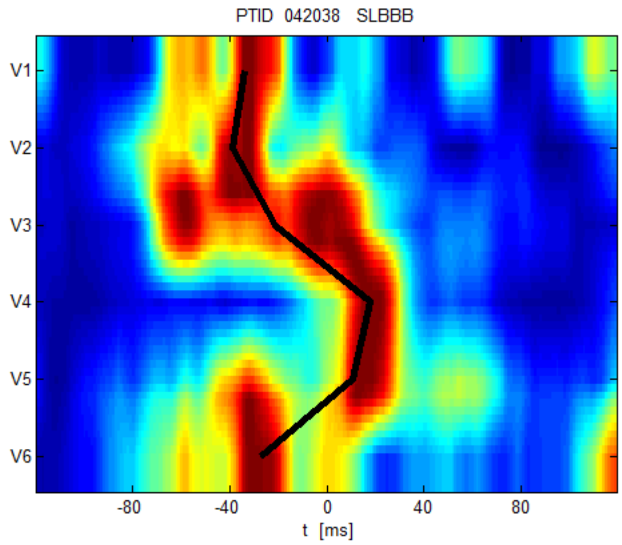

PTID 042038 SLBBB
V1
V2
V3
V4
V5
V6
-80
-40
0
40
80
t [ms]

PTID 042041 SLBBB

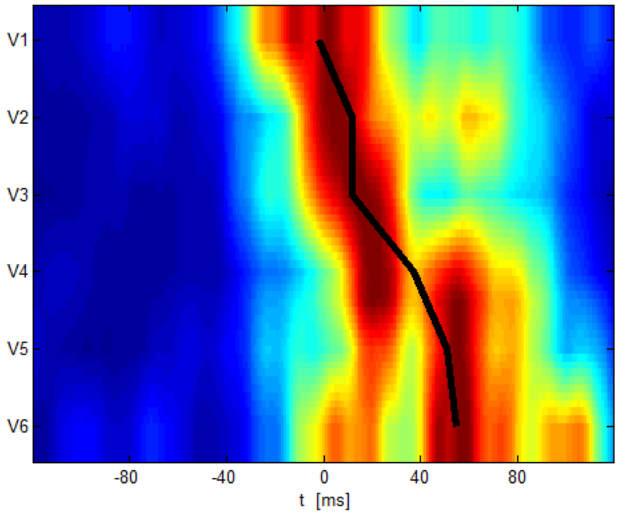

PTID 042044 SLBBB

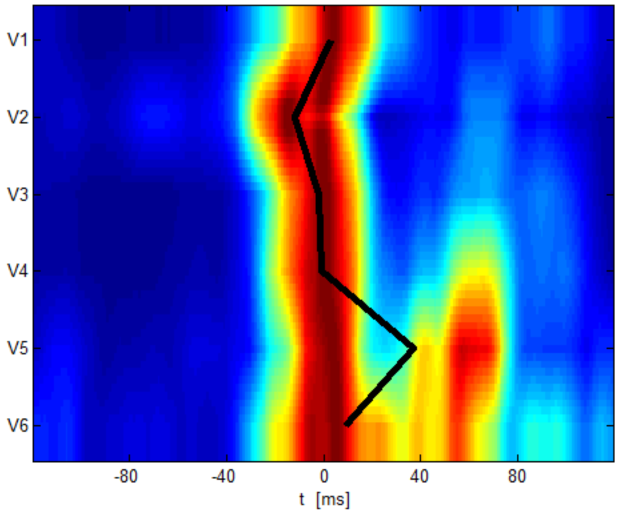

PTID 042046 SLBBB

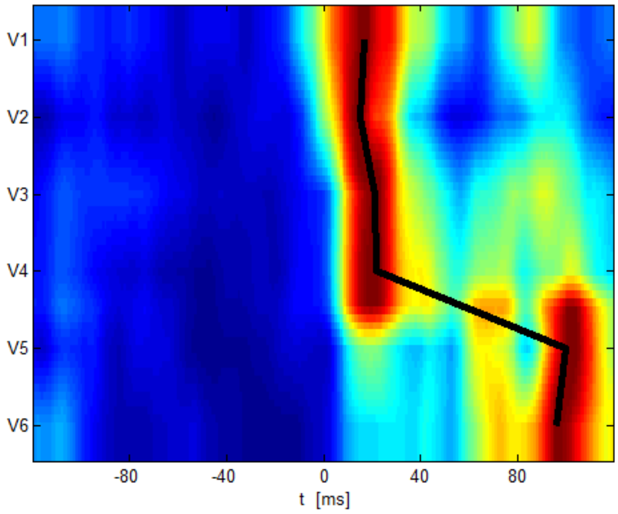

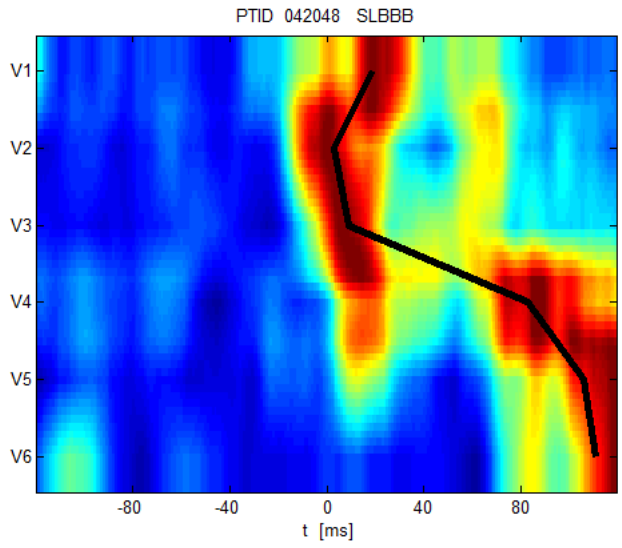

PTID 042048 SLBBB
V1
V2
V3
V4
V5
V6
-80
-40
0
40
80
t [ms]

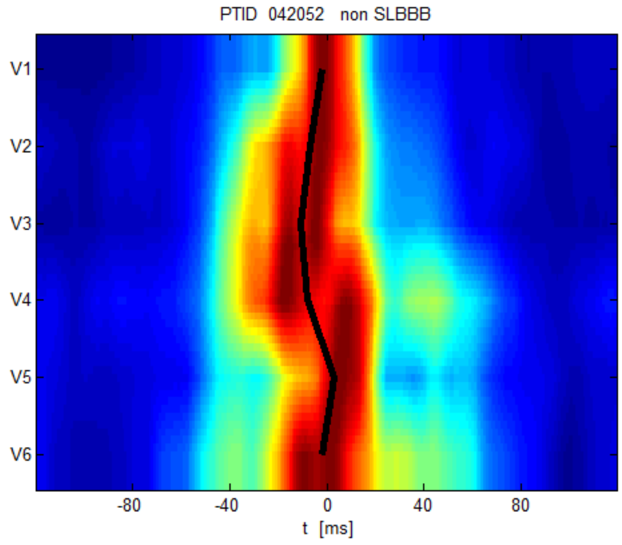

PTID 042052 non SLBBB
V1
V2
V3
V4
V5
V6
-80
-40
0
40
80
t [ms]

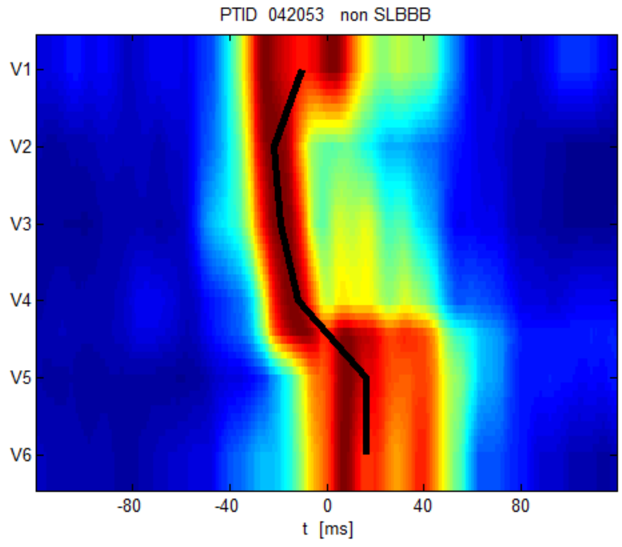

PTID 042053 non SLBBB
V1
V2
V3
V4
V5
V6
-80
-40
0
40
80
t [ms]

PTID 042055 SLBBB

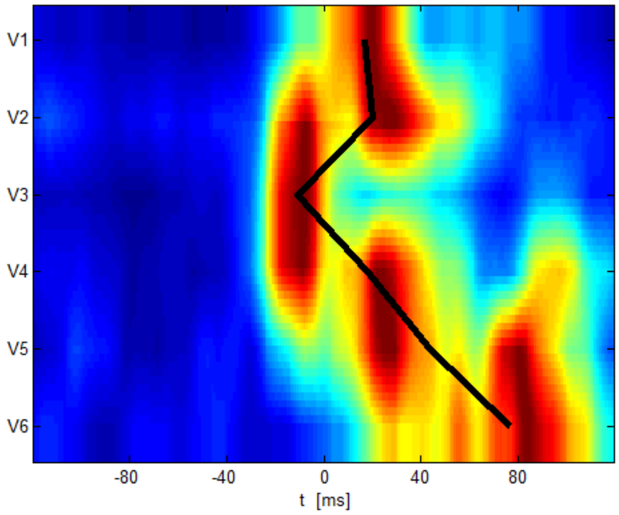

PTID 042056 SLBBB

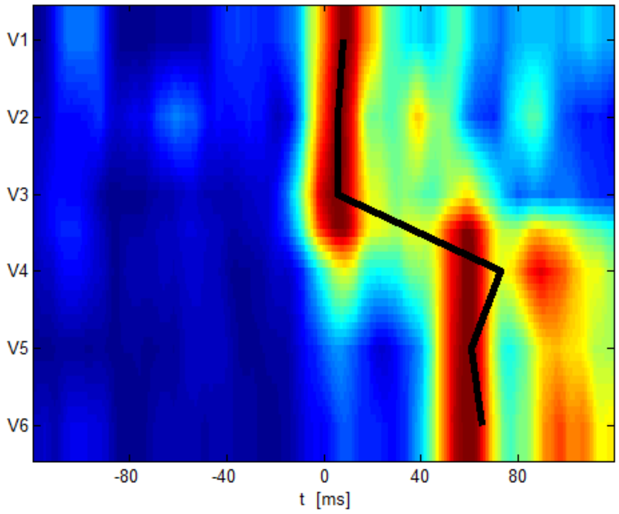

PTID 042057 non SLBBB

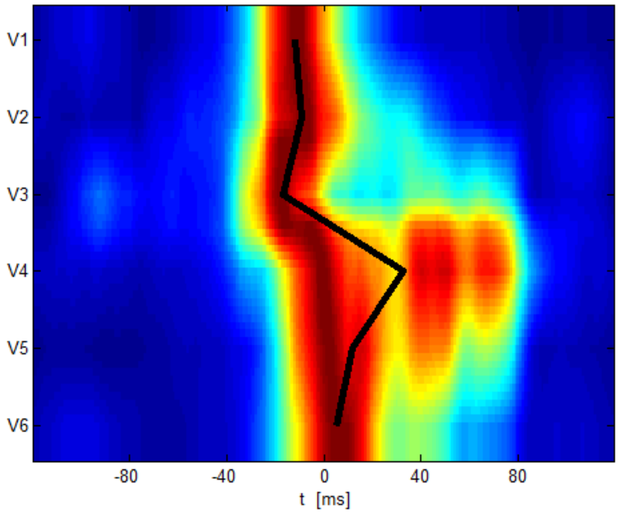

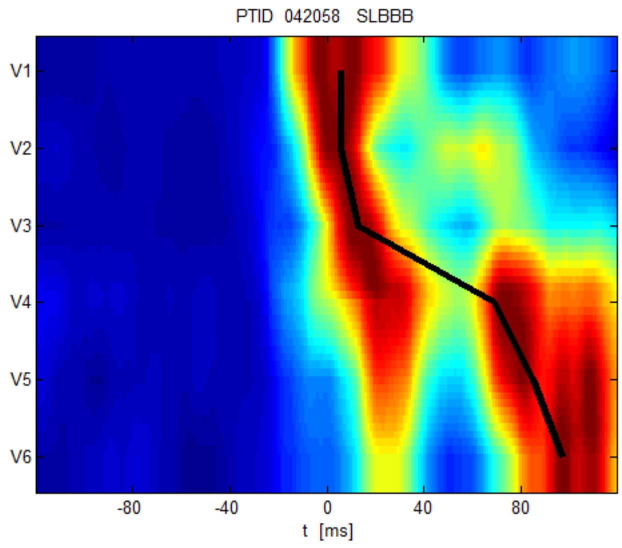
PTID 042058 SLBBB
V1
V2
V3
V4
V5
V6
-80
-40
0
40
80
t [ms]

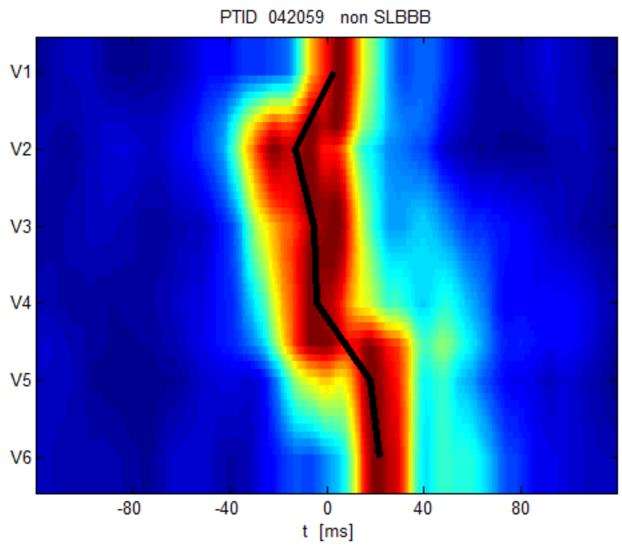
PTID 042059 non SLBBB
V1
V2
V3
V4
V5
V6
-80
-40
0
40
80
t [ms]

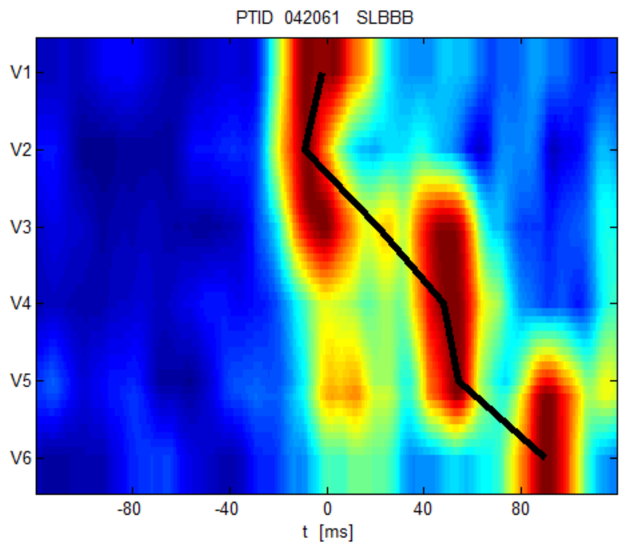
PTID 042061 SLBBB
V1
V2
V3
V4
V5
V6
-80
-40
0
40
80
t [ms]

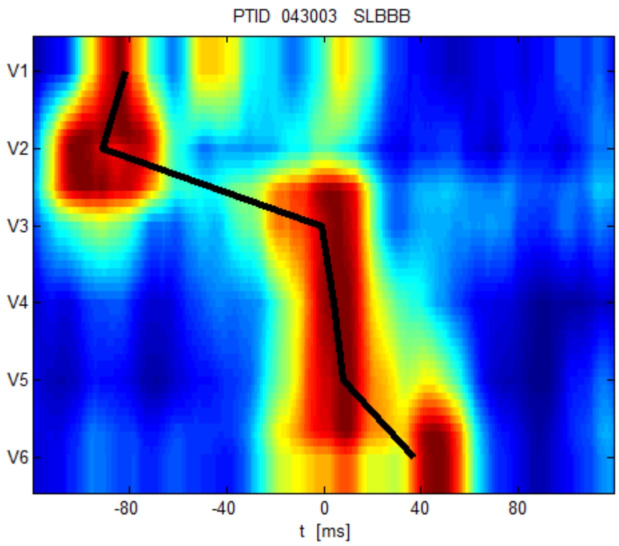
PTID 043003 SLBBB
V1
V2
V3
V4
V5
V6
-80
-40
0
40
80
t [ms]

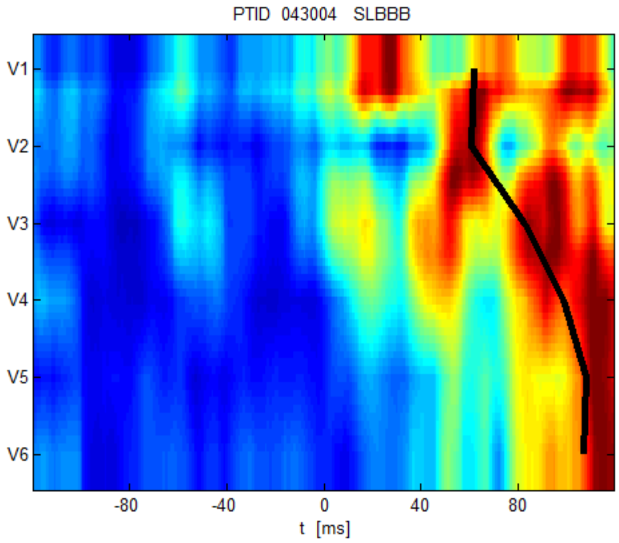
PTID 043004 SLBBB
V1
V2
V3
V4
V5
V6
-80
-40
0
40
80
t [ms]

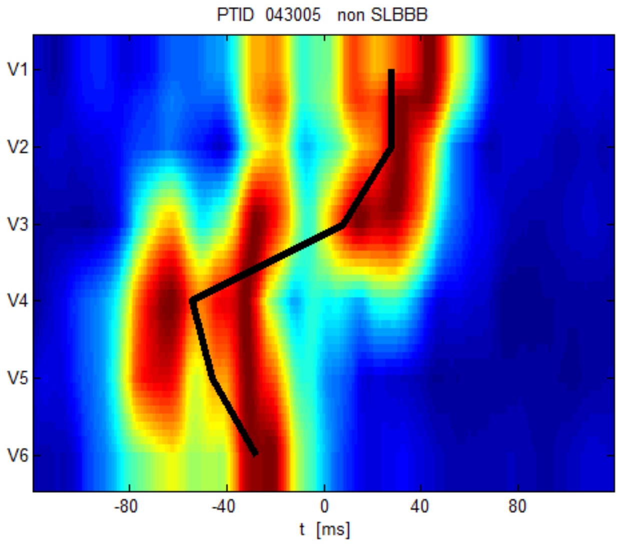
PTID 043005 non SLBBB
V1
V2
V3
V4
V5
V6
-80
-40
0
40
80
t [ms]

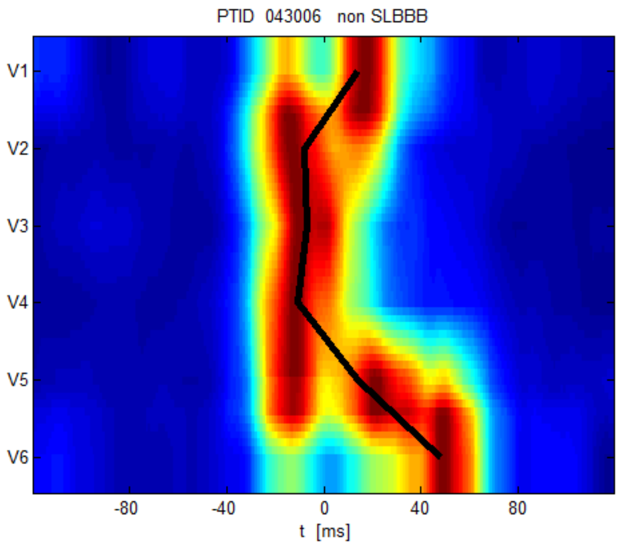
PTID 043006 non SLBBB
V1
V2
V3
V4
V5
V6
-80
-40
0
40
80
t [ms]

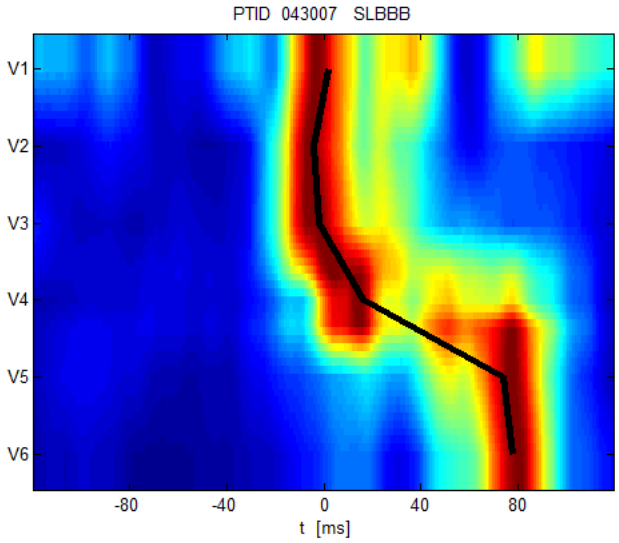
PTID 043007 SLBBB
V1
V2
V3
V4
V5
V6
-80
-40
0
40
80
t [ms]

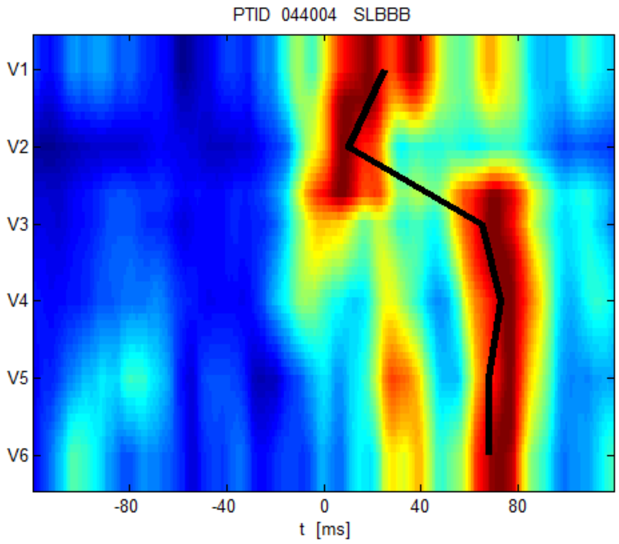
PTID 044004 SLBBB
V1
V2
V3
V4
V5
V6
-80
-40
0
40
80
t [ms]

PTID 045008 SLBBB

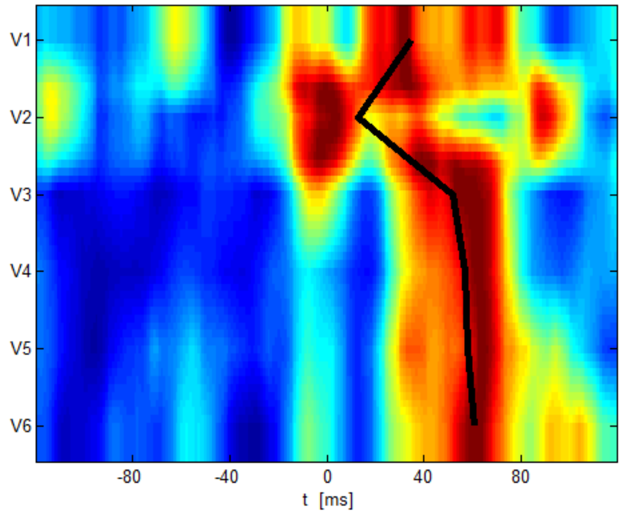

PTID 045010 SLBBB

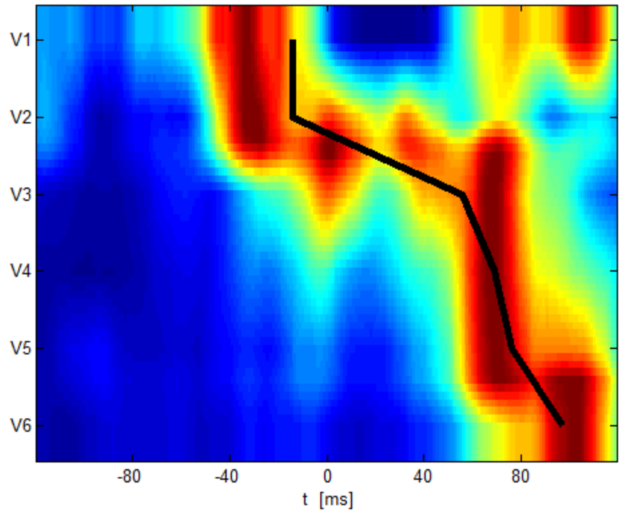

PTID 046002 non SLBBB

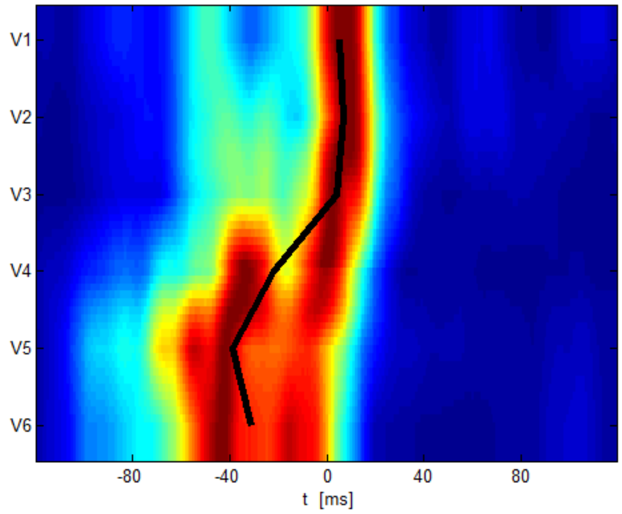

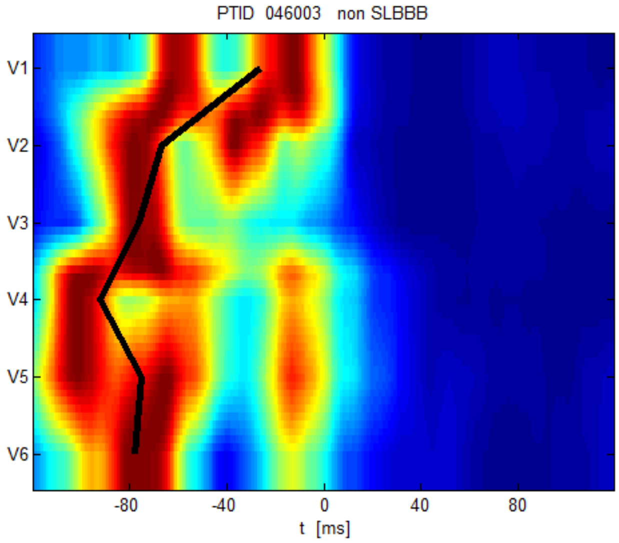
PTID 046003 non SLBBB
V1
V2
V3
V4
V5
V6
-80
-40
0
40
80
t [ms]

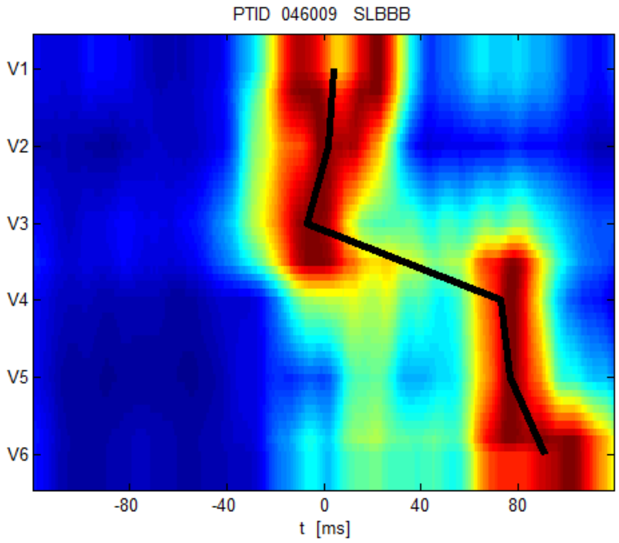
PTID 046009 SLBBB
V1
V2
V3
V4
V5
V6
-80
-40
0
40
80
t [ms]

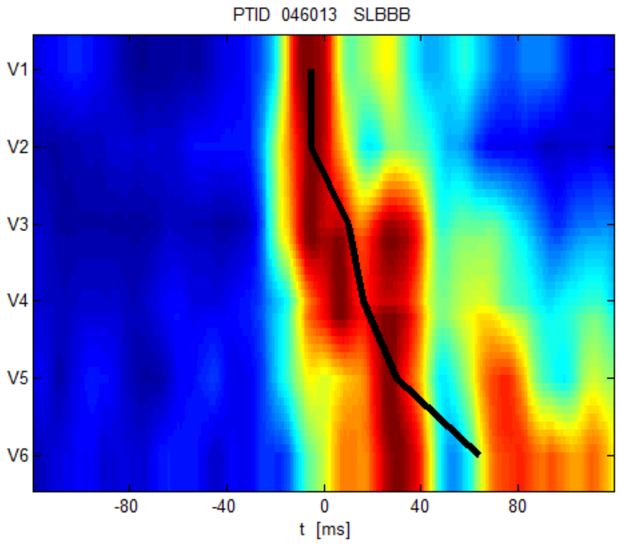
PTID 046013 SLBBB
V1
V2
V3
V4
V5
V6
-80
-40
0
40
80
t [ms]

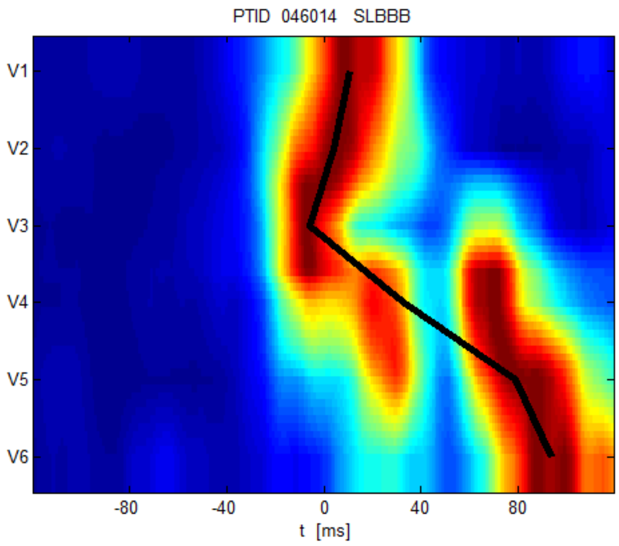
PTID 046014 SLBBB
V1
V2
V3
V4
V5
V6
-80
-40
0
40
80
t [ms]

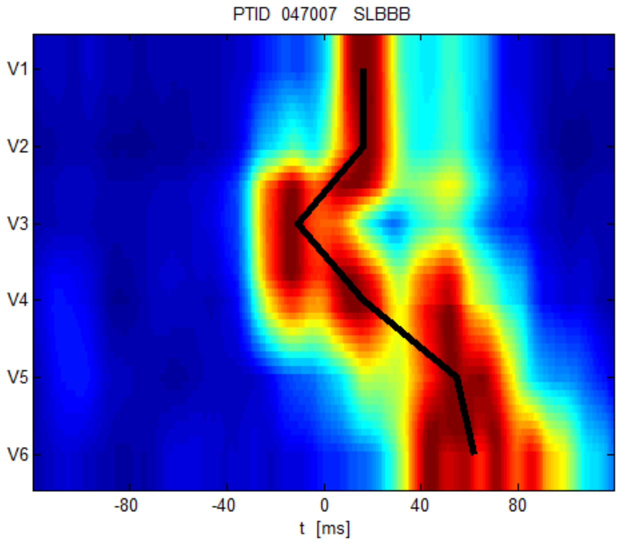
PTID 047007 SLBBB
V1
V2
V3
V4
V5
V6
-80
-40
0
40
80
t [ms]

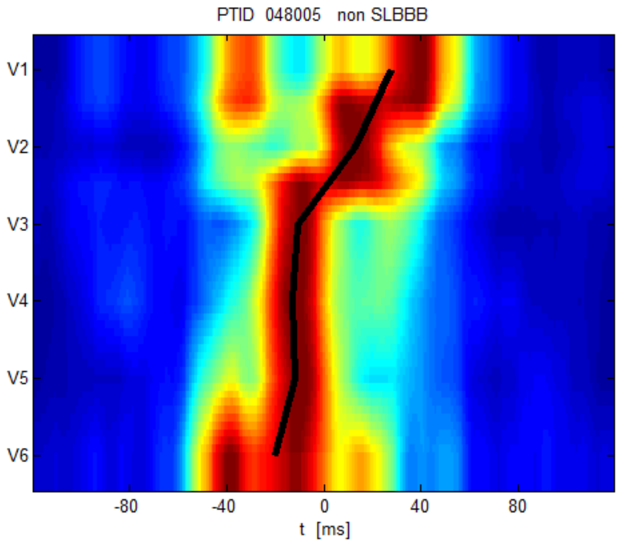
PTID 048005 non SLBBB
V1
V2
V3
V4
V5
V6
-80
-40
0
40
80
t [ms]

PTID 048008 SLBBB

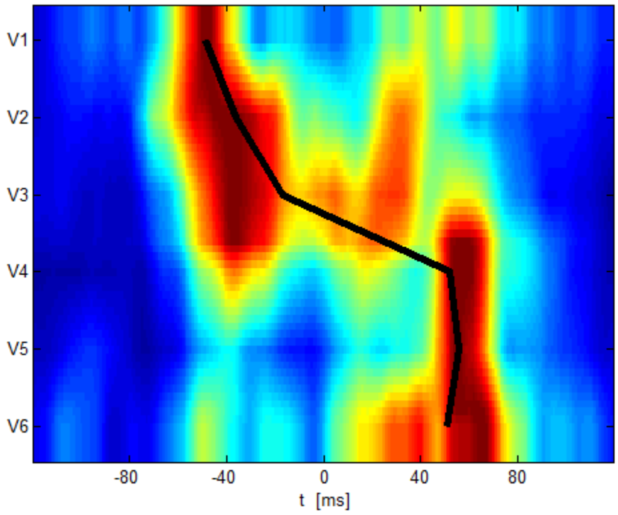

PTID 048015 non SLBBB

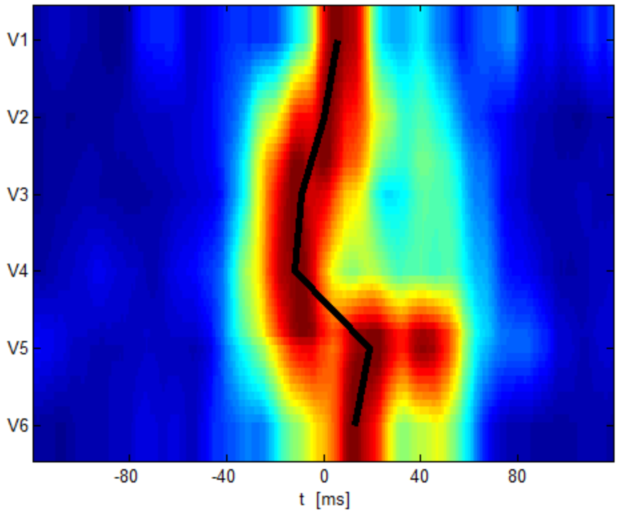

PTID 048016 SLBBB

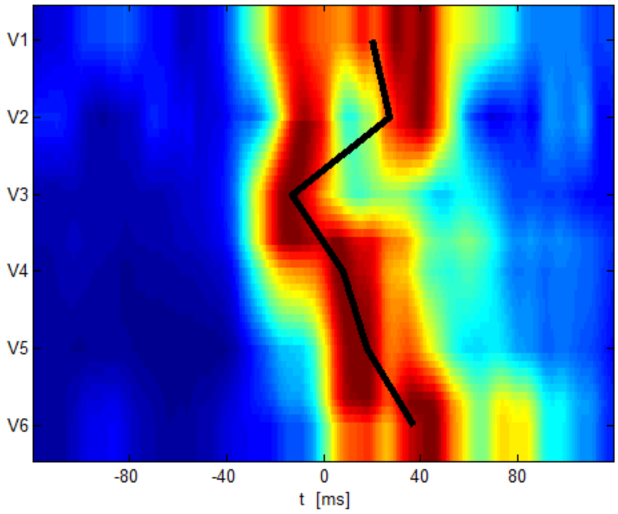

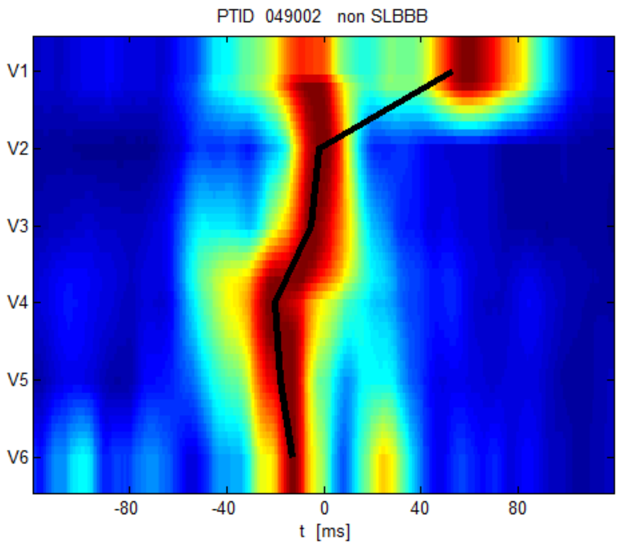
PTID 049002 non SLBBB
V1
V2
V3
V4
V5
V6
-80
-40
0
40
80
t [ms]

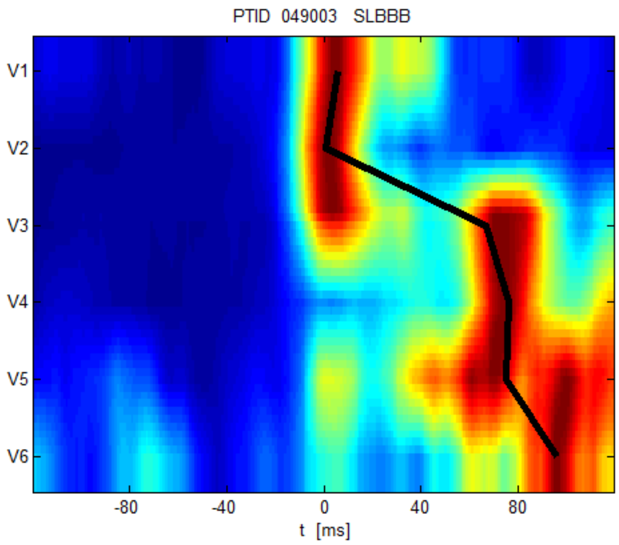
PTID 049003 SLBBB
V1
V2
V3
V4
V5
V6
-80
-40
0
40
80
t [ms]

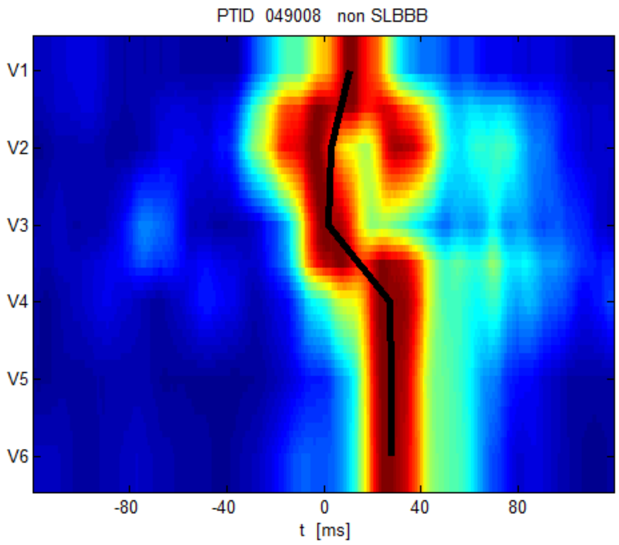
PTID 049008 non SLBBB
V1
V2
V3
V4
V5
V6
-80
-40
0
40
80
t [ms]

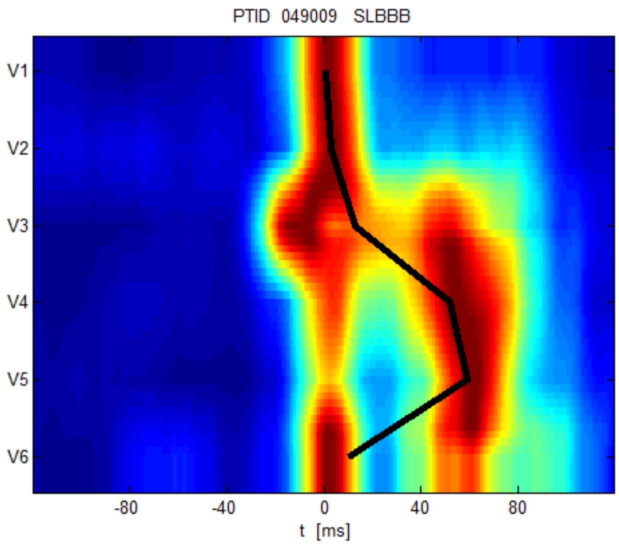
PTID 049009 SLBBB
V1
V2
V3
V4
V5
V6
-80
-40
0
40
80
t [ms]

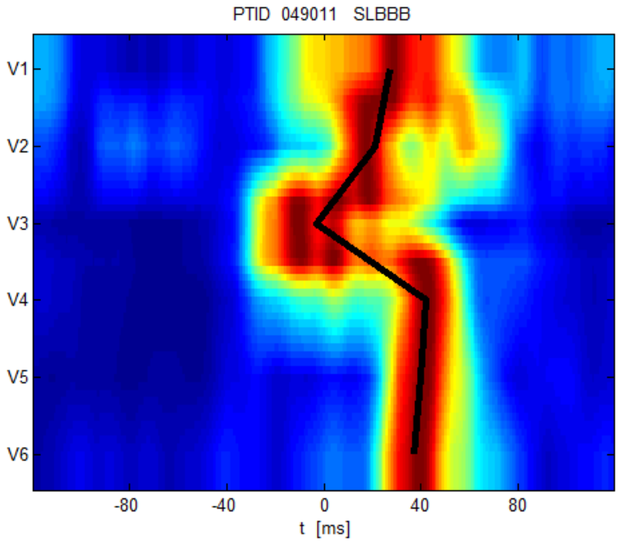
PTID 049011 SLBBB
V1
V2
V3
V4
V5
V6
-80
-40
0
40
80
t [ms]

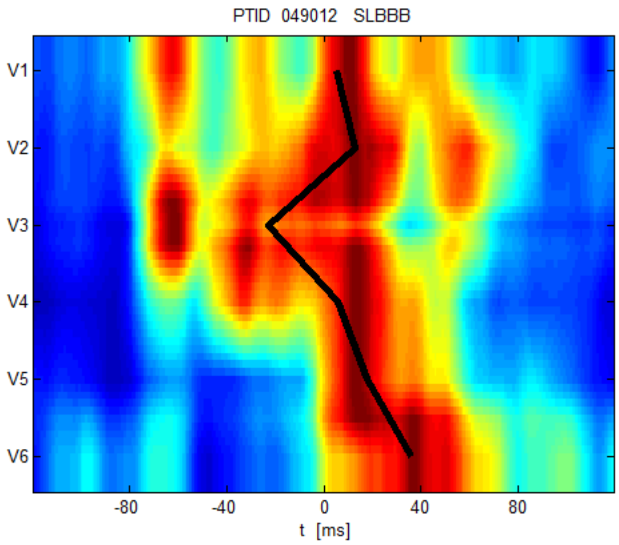
PTID 049012 SLBBB
V1
V2
V3
V4
V5
V6
-80
-40
0
40
80
t [ms]

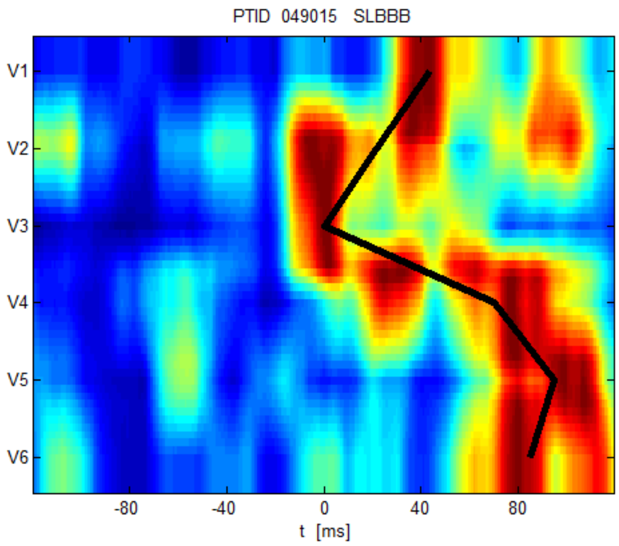
PTID 049015 SLBBB
V1
V2
V3
V4
V5
V6
-80
-40
0
40
80
t [ms]

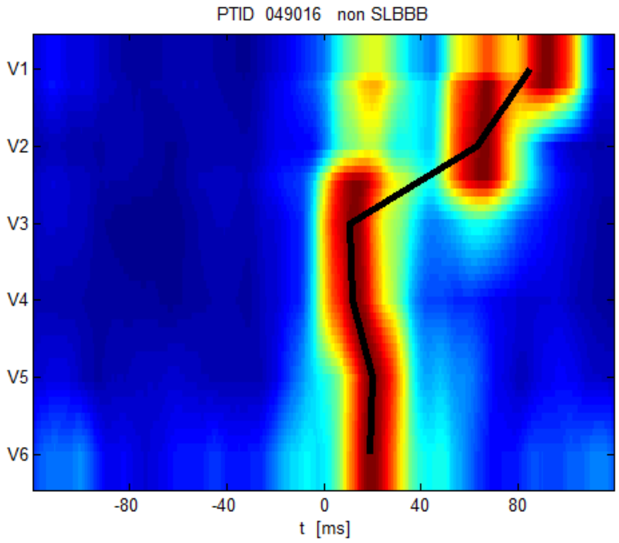
PTID 049016 non SLBBB
V1
V2
V3
V4
V5
V6
-80
-40
0
40
80
t [ms]

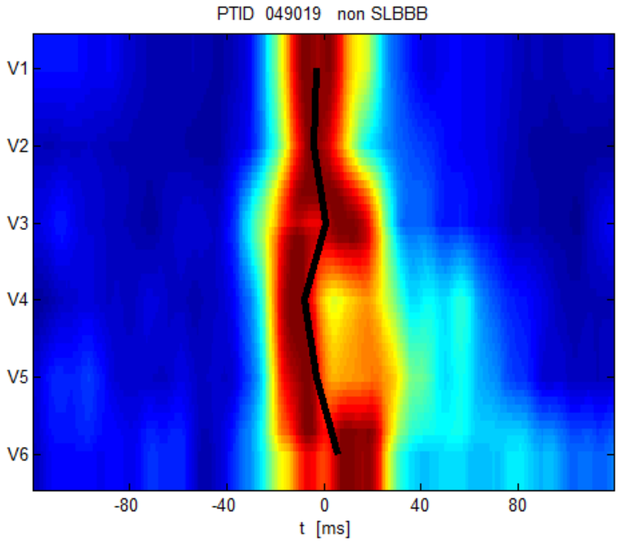
PTID 049019 non SLBBB
V1
V2
V3
V4
V5
V6
-80
-40
0
40
80
t [ms]

PTID 051007 SLBBB

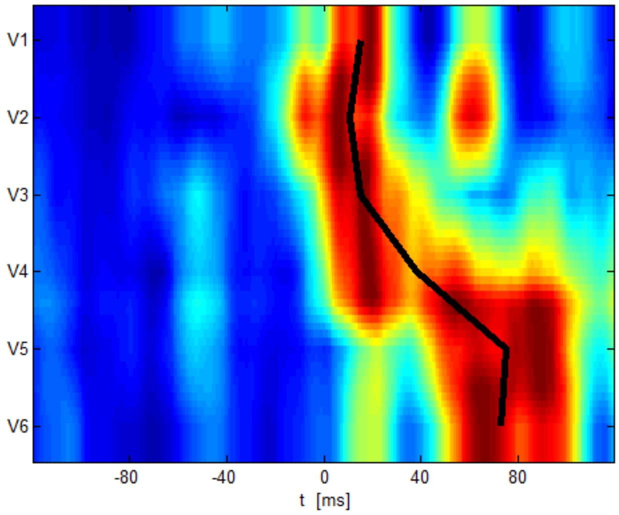

PTID 051011 SLBBB

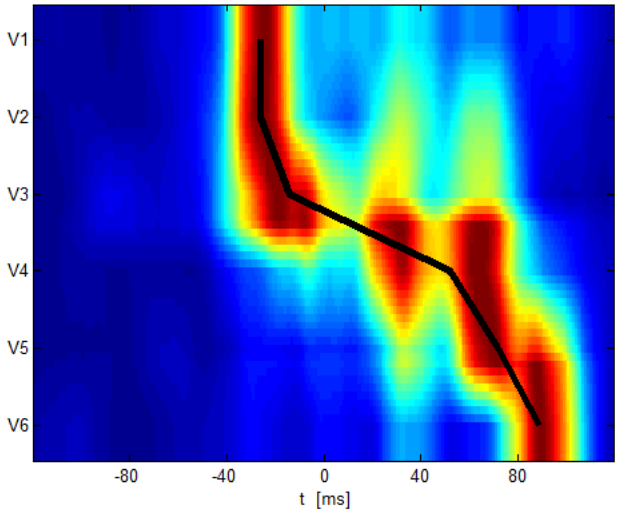

PTID 051013 SLBBB

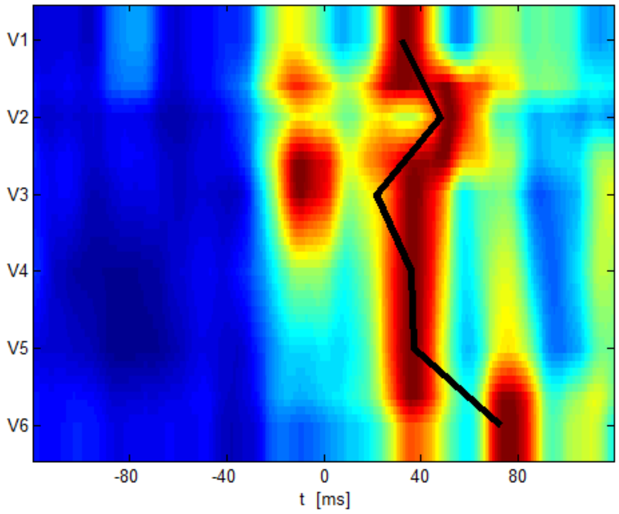

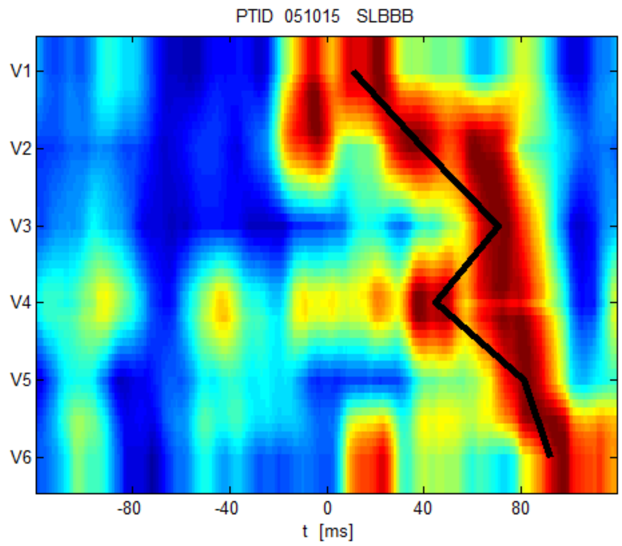
PTID 051015 SLBBB
V1
V2
V3
V4
V5
V6
-80
-40
0
40
80
t [ms]

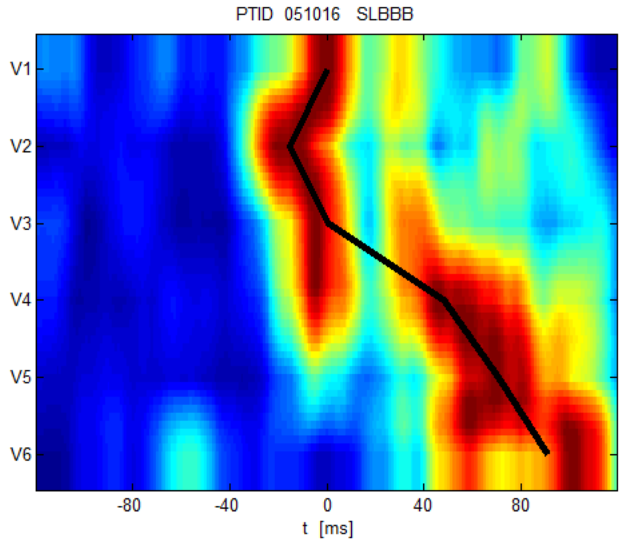
PTID 051016 SLBBB
V1
V2
V3
V4
V5
V6
-80
-40
0
40
80
t [ms]

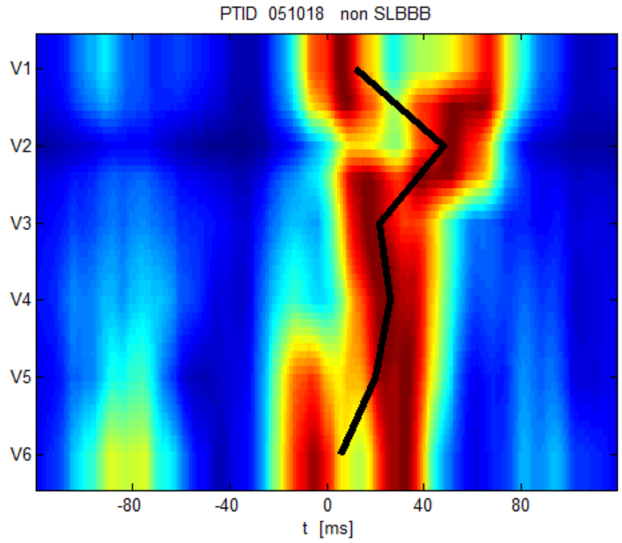
PTID 051018 non SLBBB
V1
V2
V3
V4
V5
V6
-80
-40
0
40
80
t [ms]

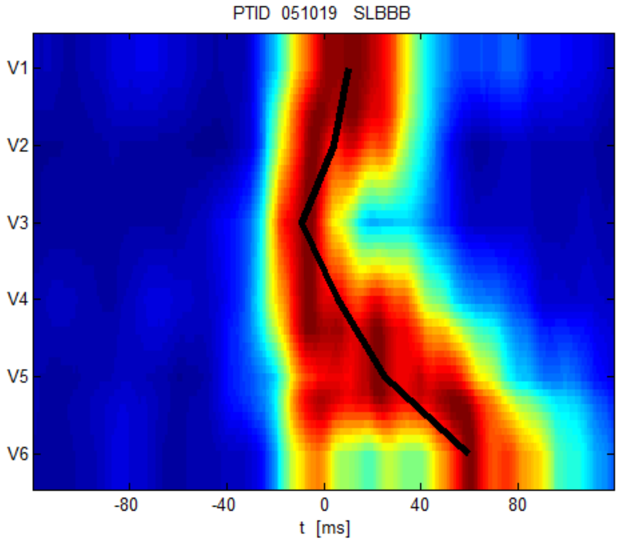
PTID 051019 SLBBB
V1
V2
V3
V4
V5
V6
-80
-40
0
40
80
t [ms]

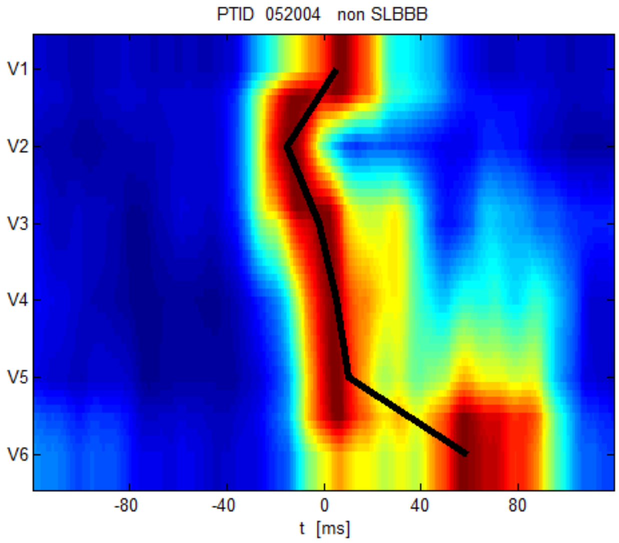
PTID 052004 non SLBBB
V1
V2
V3
V4
V5
V6
-80
-40
0
40
80
t [ms]

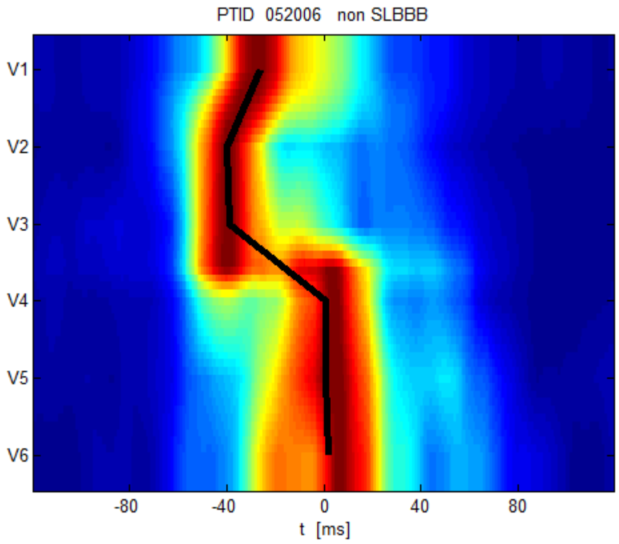
PTID 052006 non SLBBB
V1
V2
V3
V4
V5
V6
-80
-40
0
40
80
t [ms]

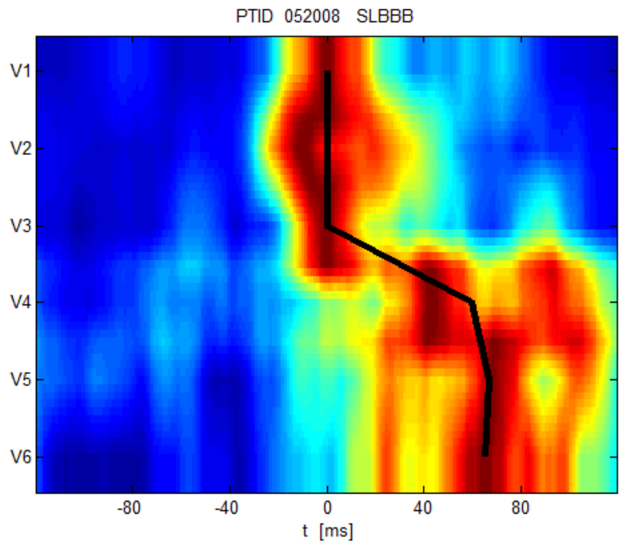
PTID 052008 SLBBB
V1
V2
V3
V4
V5
V6
-80
-40
0
40
80
t [ms]

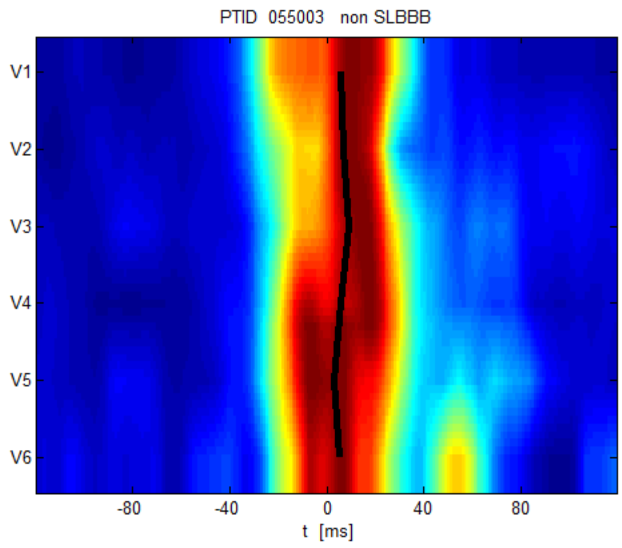
PTID 055003 non SLBBB
V1
V2
V3
V4
V5
V6
-80
-40
0
40
80
t [ms]

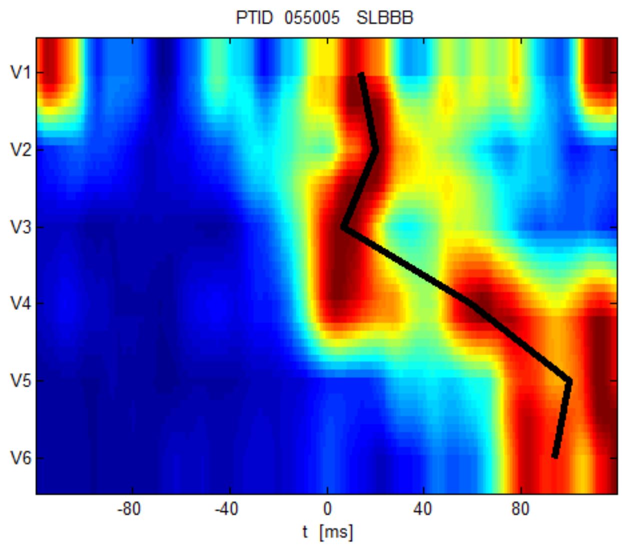
PTID 055005 SLBBB
V1
V2
V3
V4
V5
V6
-80
-40
0
40
80
t [ms]

PTID 055007 non SLBBB

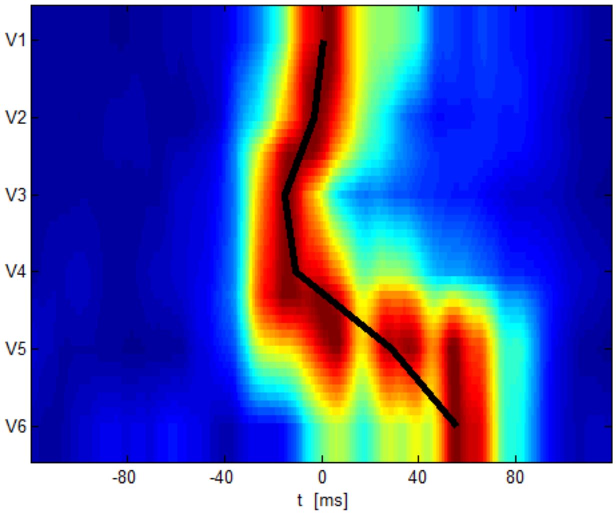

PTID 055011 non SLBBB

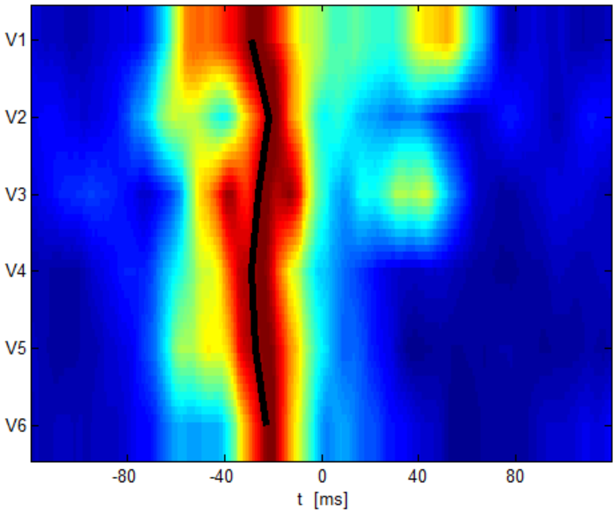

PTID 055013 SLBBB

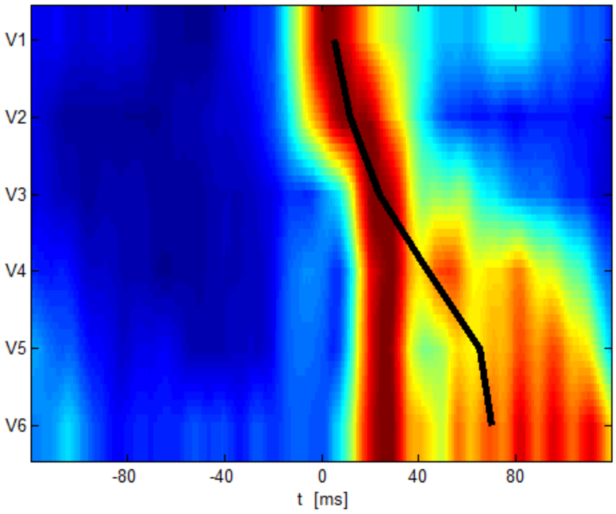

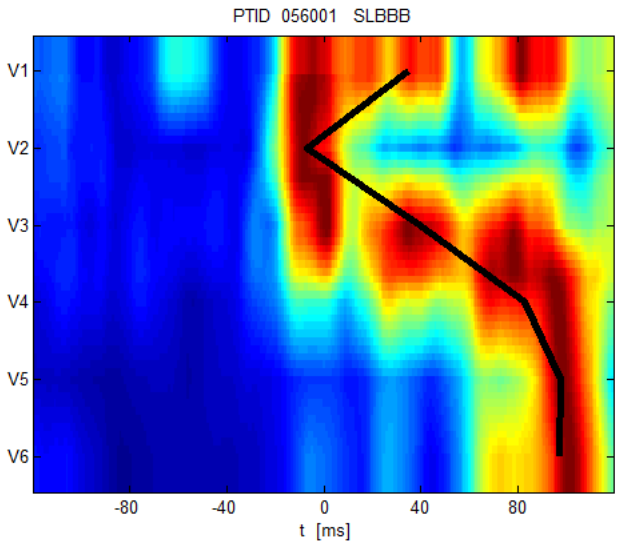

PTID 056001 SLBBB
V1
V2
V3
V4
V5
V6
-80
-40
0
40
80
t [ms]

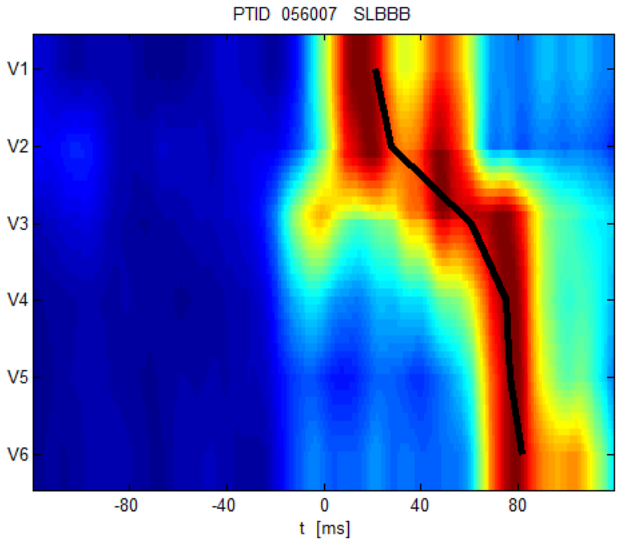

PTID 056007 SLBBB
V1
V2
V3
V4
V5
V6
-80
-40
0
40
80
t [ms]

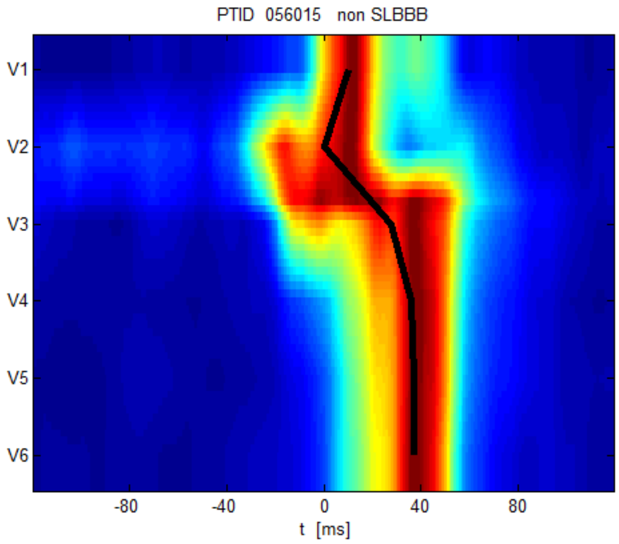

PTID 056015 non SLBBB
V1
V2
V3
V4
V5
V6
-80
-40
0
40
80
t [ms]

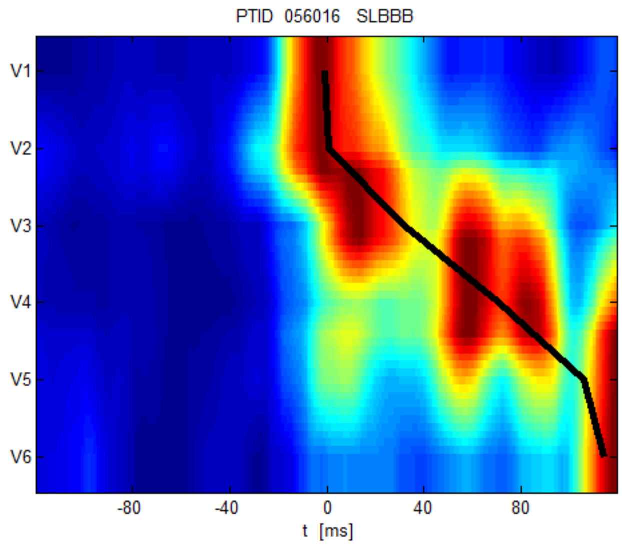
PTID 056016 SLBBB
V1
V2
V3
V4
V5
V6
-80
-40
0
40
80
t [ms]

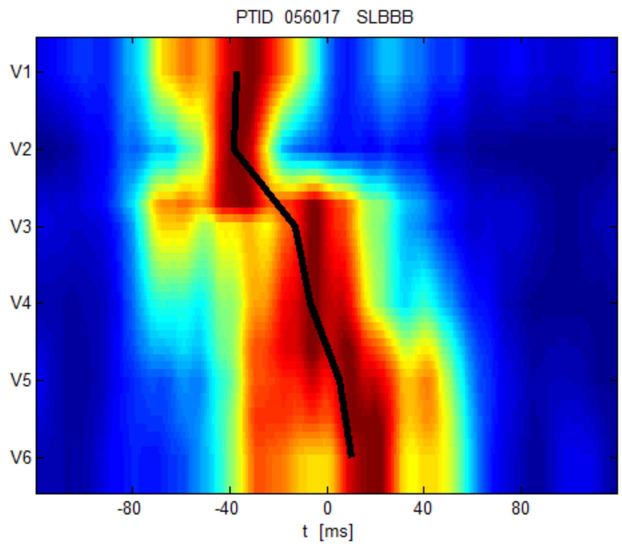
PTID 056017 SLBBB
V1
V2
V3
V4
V5
V6
-80
-40
0
40
80
t [ms]

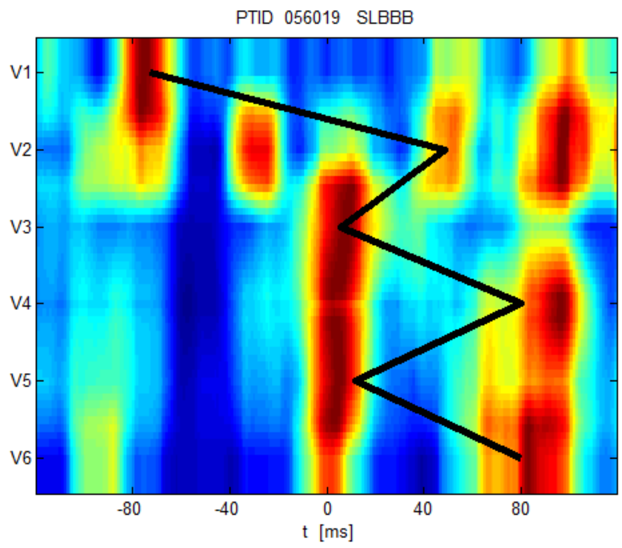
PTID 056019 SLBBB
V1
V2
V3
V4
V5
V6
-80
-40
0
40
80
t [ms]

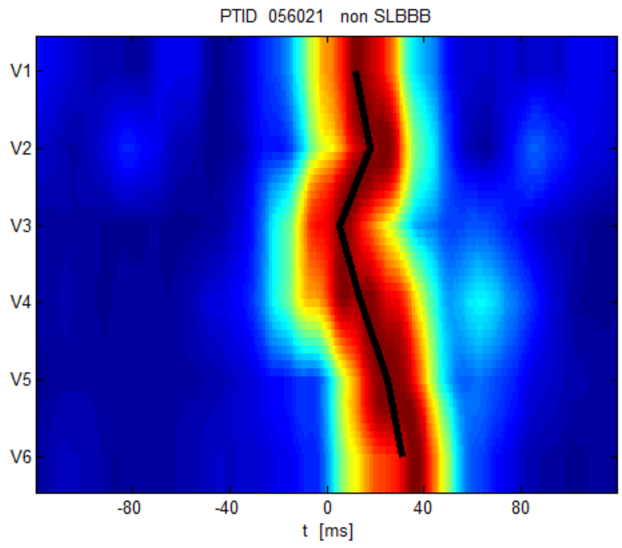
PTID 056021 non SLBBB
V1
V2
V3
V4
V5
V6
-80
-40
0
40
80
t [ms]

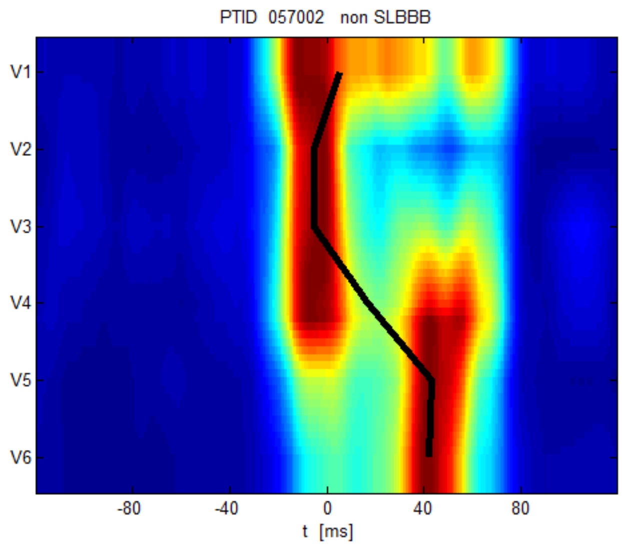
PTID 057002 non SLBBB
V1
V2
V3
V4
V5
V6
-80
-40
0
40
80
t [ms]

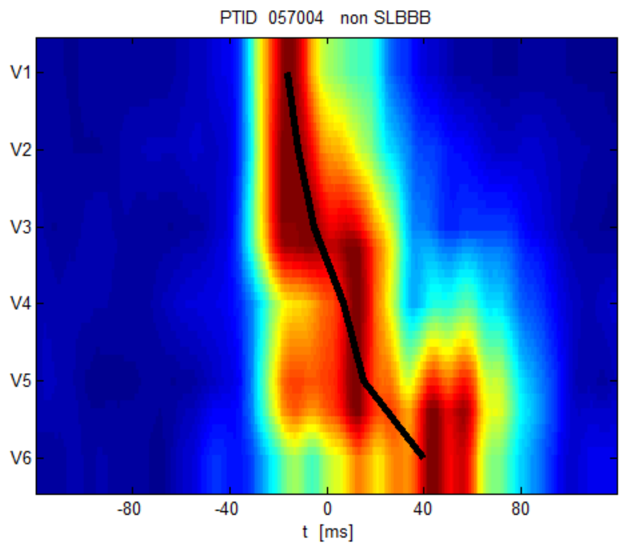
PTID 057004 non SLBBB
V1
V2
V3
V4
V5
V6
-80
-40
0
40
80
t [ms]

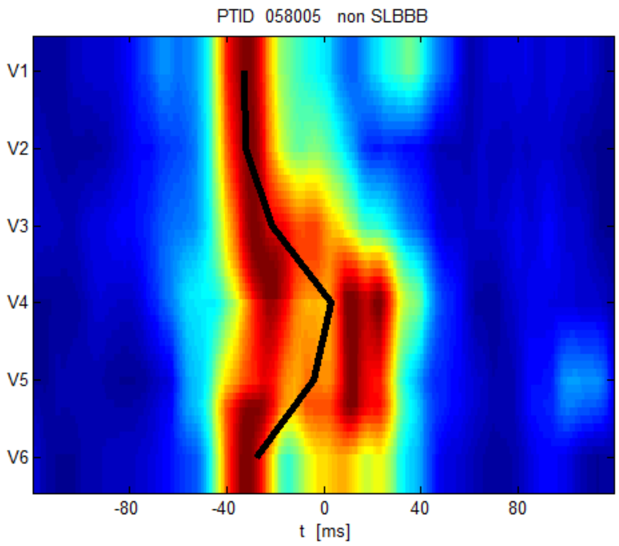
PTID 058005 non SLBBB
V1
V2
V3
V4
V5
V6
-80
-40
0
40
80
t [ms]

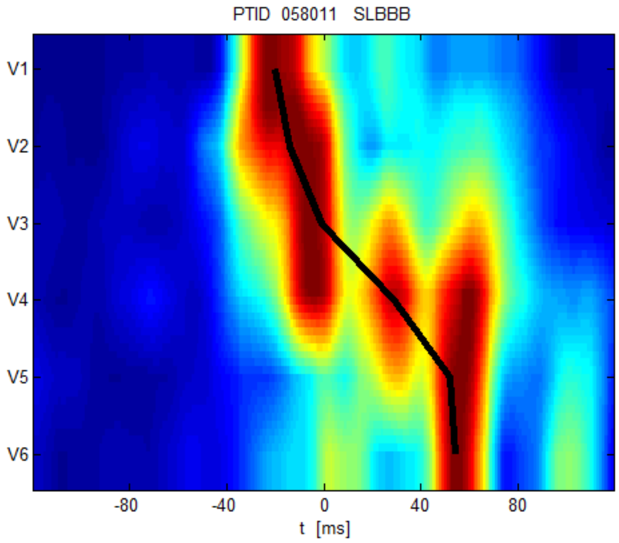
PTID 058011 SLBBB
V1
V2
V3
V4
V5
V6
-80
-40
0
40
80
t [ms]

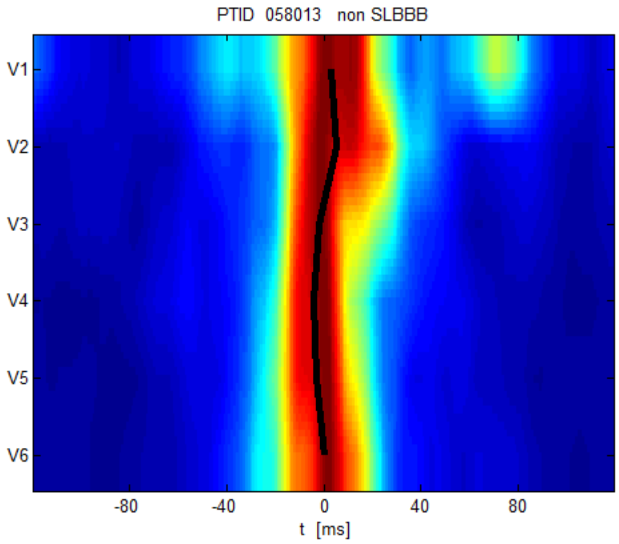
PTID 058013 non SLBBB
V1
V2
V3
V4
V5
V6
-80
-40
0
40
80
t [ms]

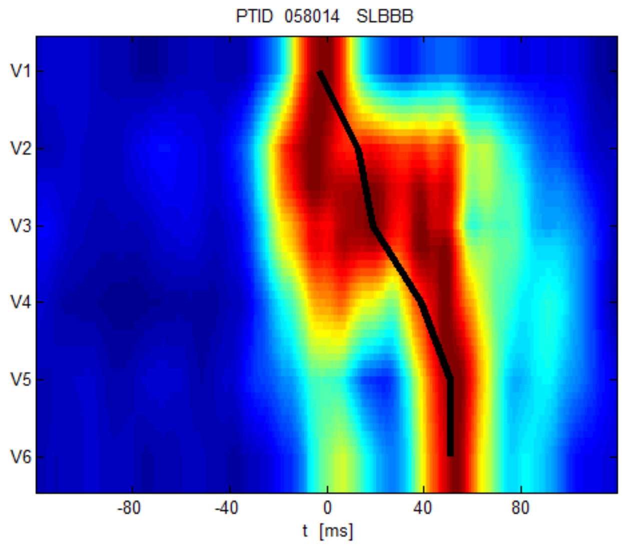
PTID 058014 SLBBB
V1
V2
V3
V4
V5
V6
-80
-40
0
40
80
t [ms]

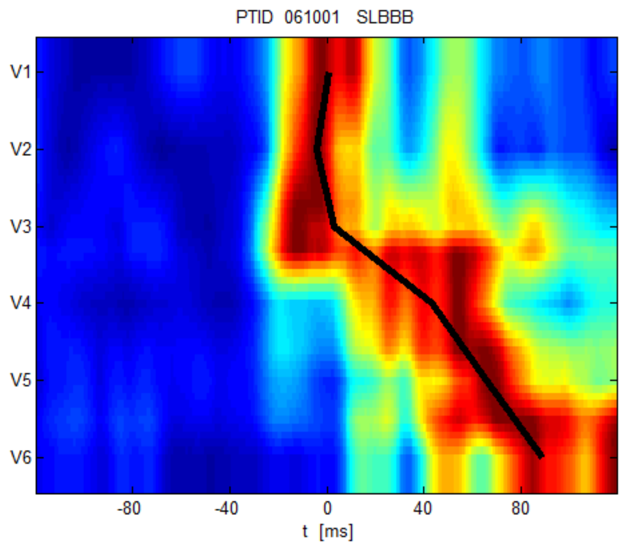
PTID 061001 SLBBB
V1
V2
V3
V4
V5
V6
-80
-40
0
40
80
t [ms]

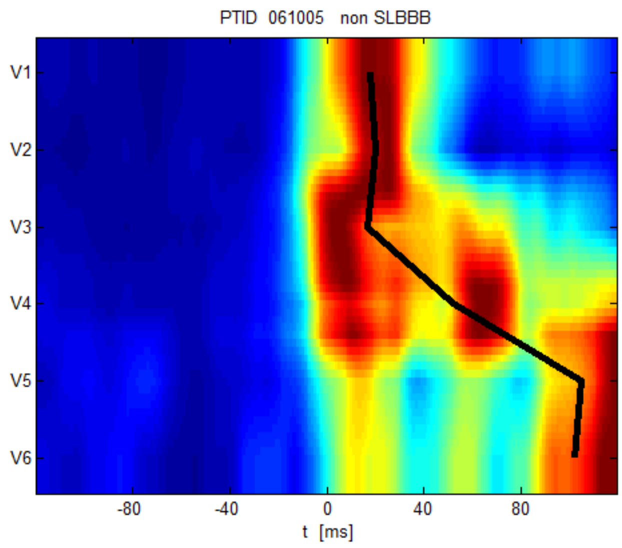
PTID 061005 non SLBBB
V1
V2
V3
V4
V5
V6
-80
-40
0
40
80
t [ms]

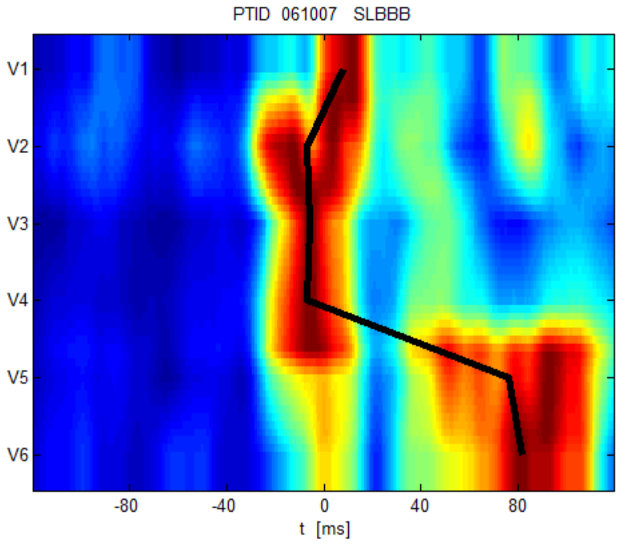
PTID 061007 SLBBB
V1
V2
V3
V4
V5
V6
-80
-40
0
40
80
t [ms]

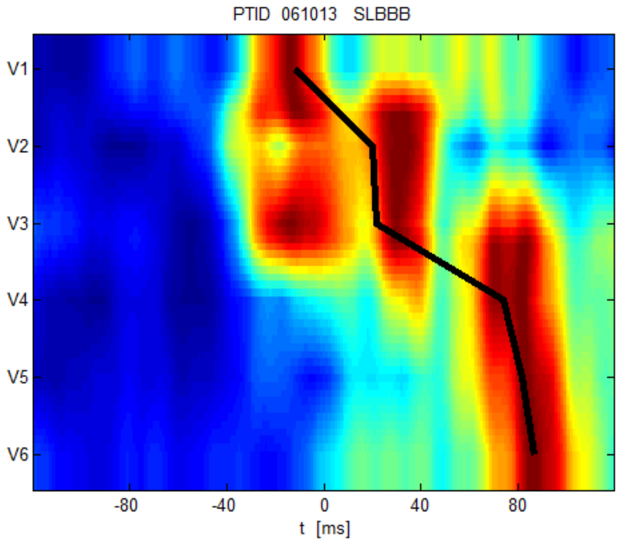
PTID 061013 SLBBB
V1
V2
V3
V4
V5
V6
-80
-40
0
40
80
t [ms]

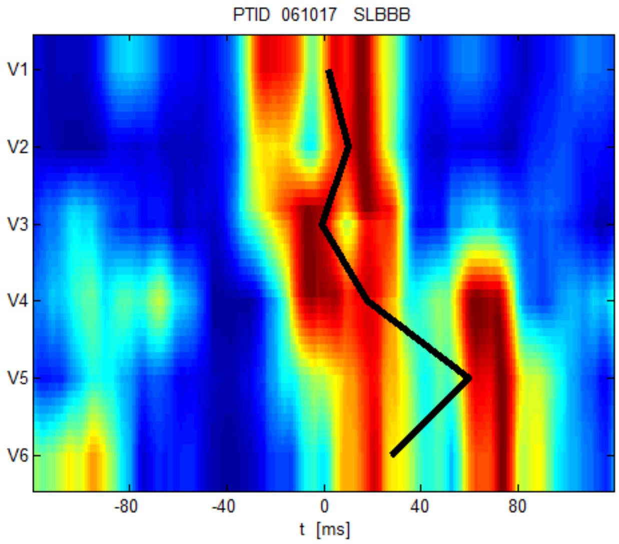
PTID 061017 SLBBB
V1
V2
V3
V4
V5
V6
-80
-40
0
40
80
t [ms]

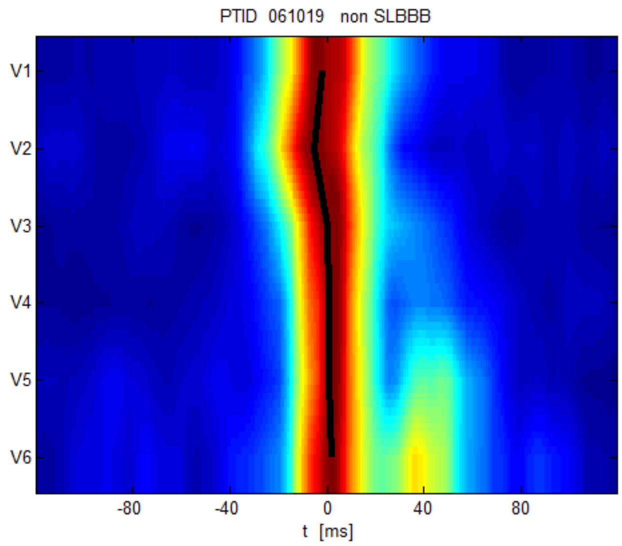
PTID 061019 non SLBBB
V1
V2
V3
V4
V5
V6
-80
-40
0
40
80
t [ms]

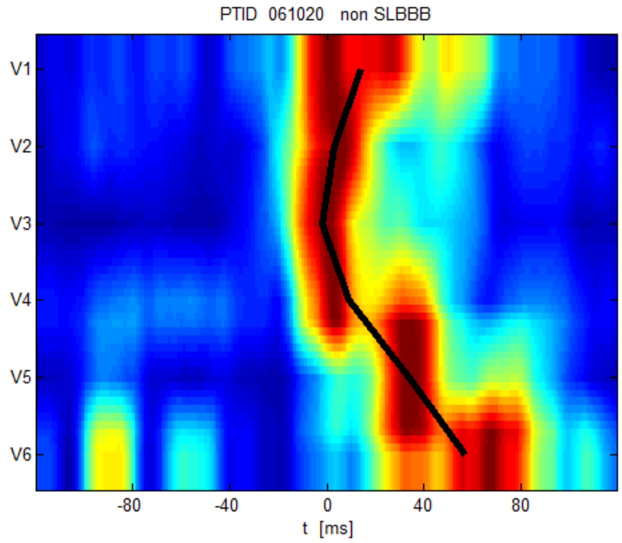
PTID 061020 non SLBBB
V1
V2
V3
V4
V5
V6
-80
-40
0
40
80
t [ms]

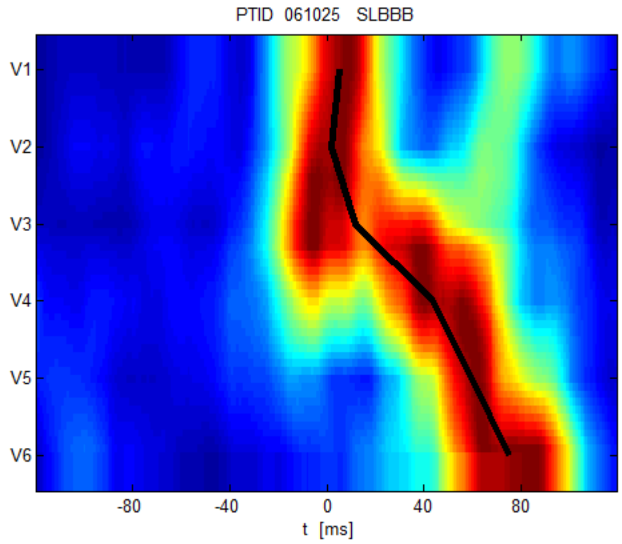
PTID 061025 SLBBB
V1
V2
V3
V4
V5
V6
-80
-40
0
40
80
t [ms]

PTID 062003 SLBBB

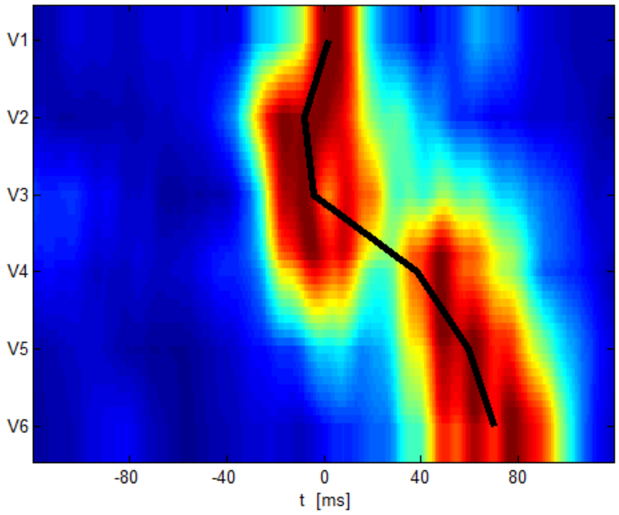

PTID 064002 SLBBB

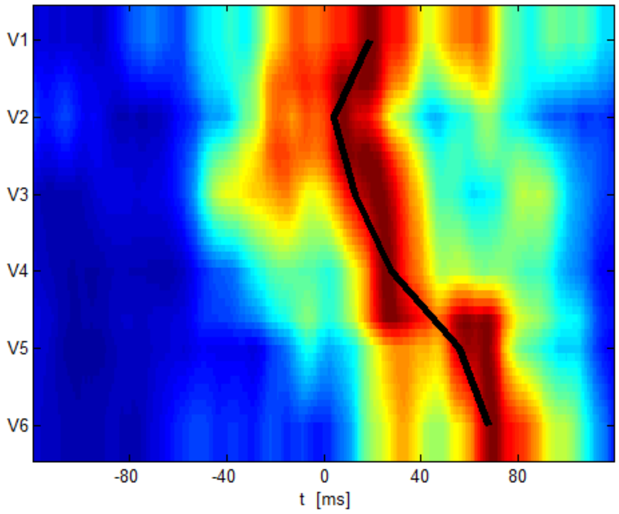

PTID 064003 SLBBB

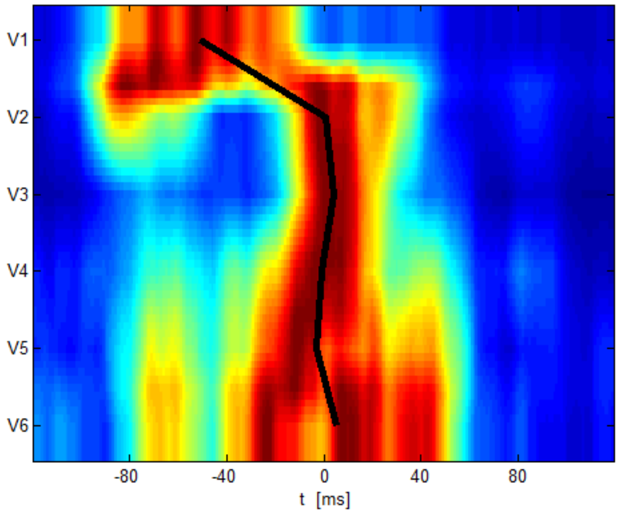

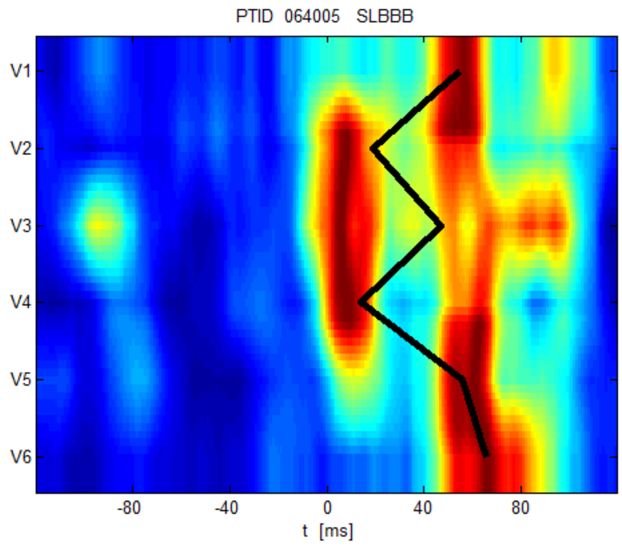
PTID 064005 SLBBB
V1
V2
V3
V4
V5
V6
-80
-40
0
40
80
t [ms]

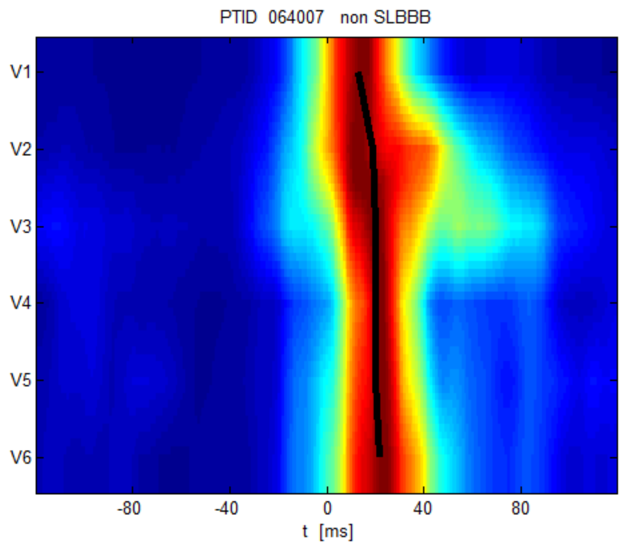
PTID 064007 non SLBBB
V1
V2
V3
V4
V5
V6
-80
-40
0
40
80
t [ms]

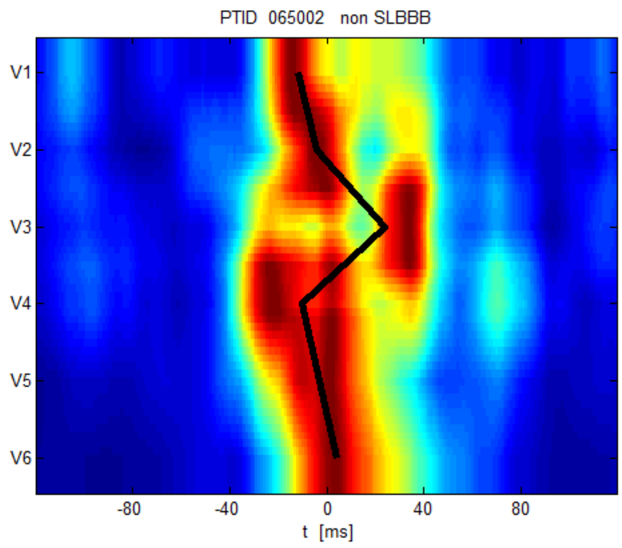
PTID 065002 non SLBBB
V1
V2
V3
V4
V5
V6
-80
-40
0
40
80
t [ms]

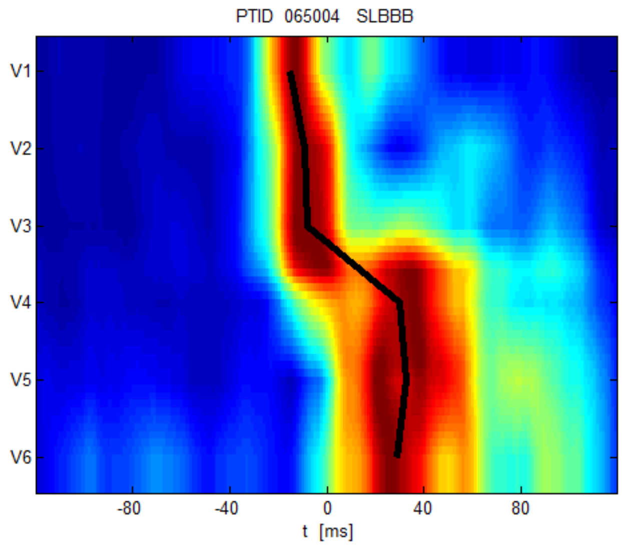
PTID 065004 SLBBB
V1
V2
V3
V4
V5
V6
-80
-40
0
40
80
t [ms]

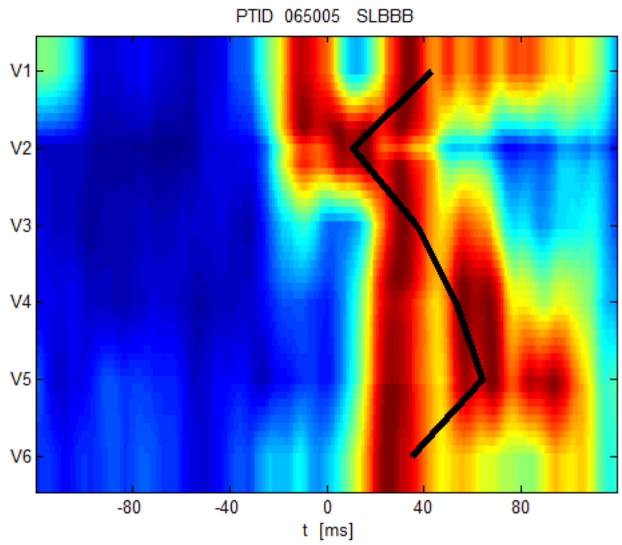
PTID 065005 SLBBB
V1
V2
V3
V4
V5
V6
-80
-40
0
40
80
t [ms]

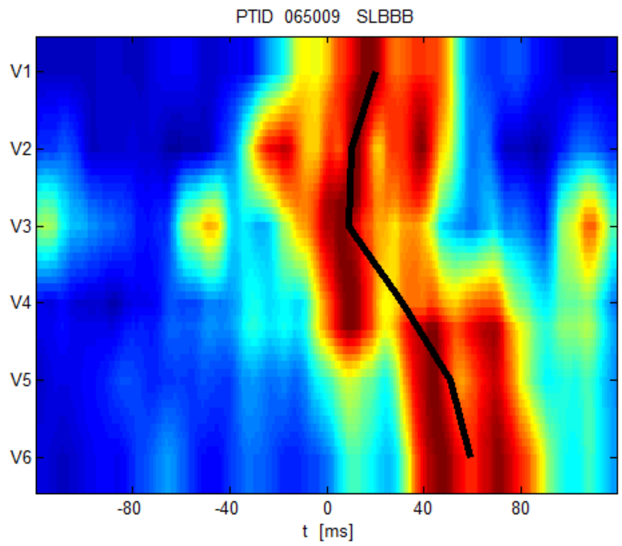
PTID 065009 SLBBB
V1
V2
V3
V4
V5
V6
-80
-40
0
40
80
t [ms]

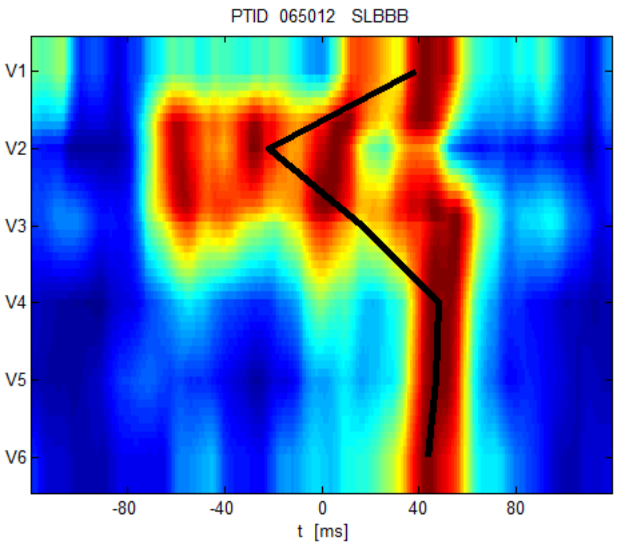
PTID 065012 SLBBB
V1
V2
V3
V4
V5
V6
-80
-40
0
40
80
t [ms]

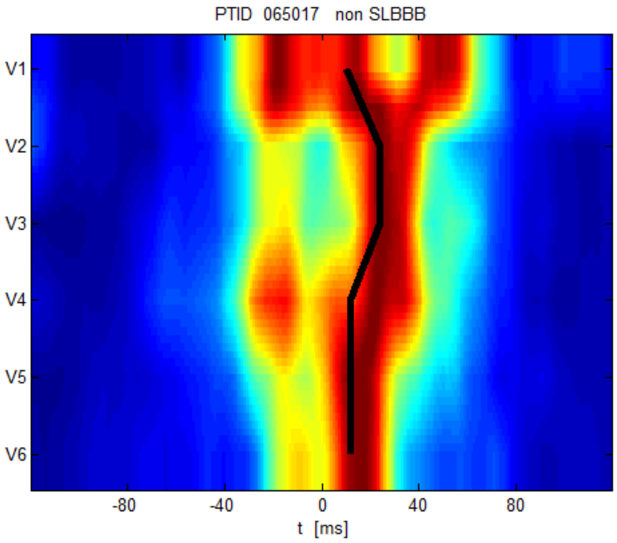
PTID 065017 non SLBBB
V1
V2
V3
V4
V5
V6
-80
-40
0
40
80
t [ms]

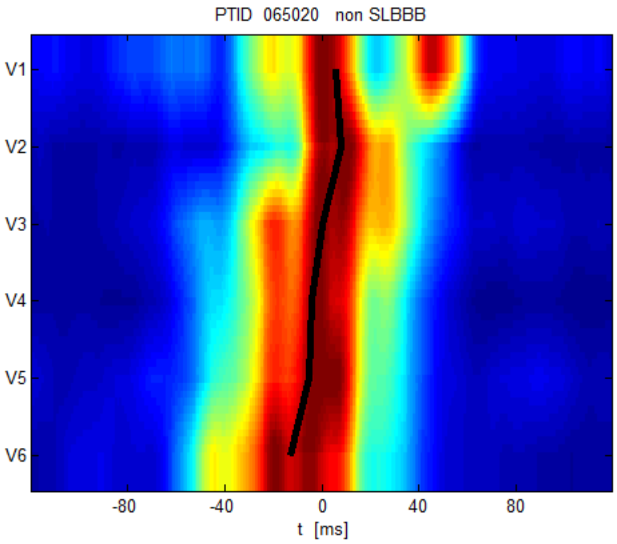
PTID 065020 non SLBBB
V1
V2
V3
V4
V5
V6
-80
-40
0
40
80
t [ms]

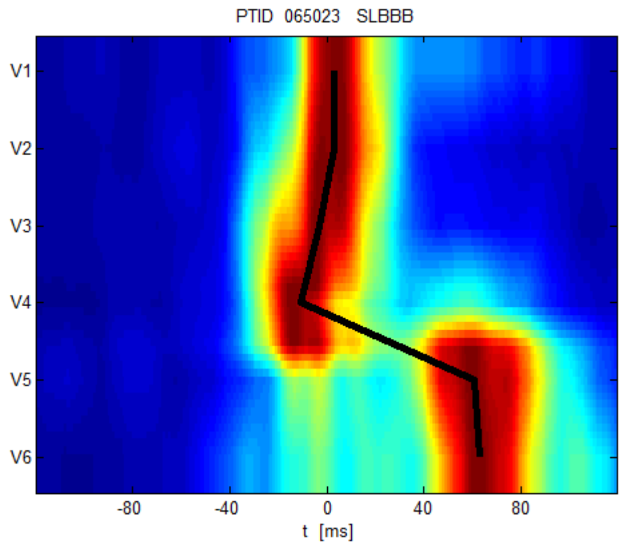
PTID 065023 SLBBB
V1
V2
V3
V4
V5
V6
-80
-40
0
40
80
t [ms]

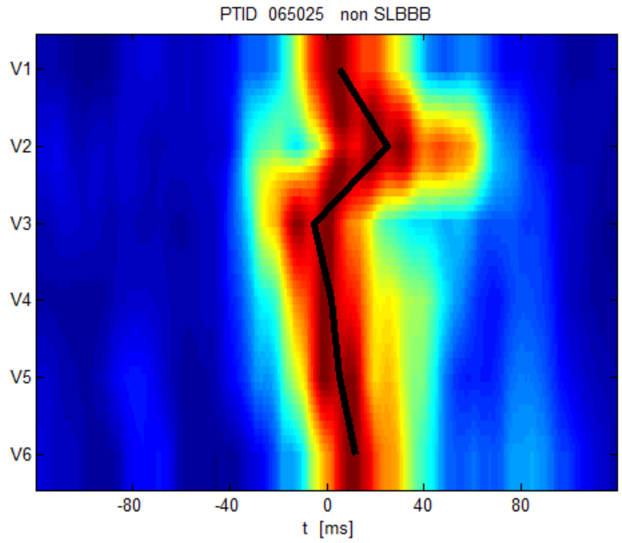
PTID 065025 non SLBBB
V1
V2
V3
V4
V5
V6
-80
-40
0
40
80
t [ms]

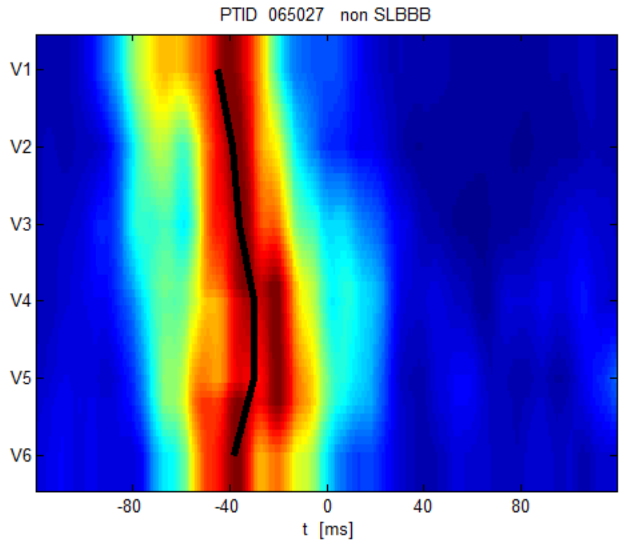
PTID 065027 non SLBBB
V1
V2
V3
V4
V5
V6
-80
-40
0
40
80
t [ms]

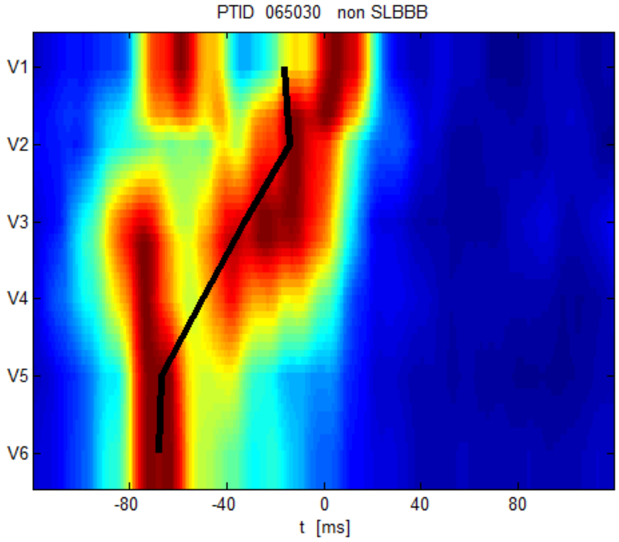
PTID 065030 non SLBBB
V1
V2
V3
V4
V5
V6
-80
-40
0
40
80
t [ms]

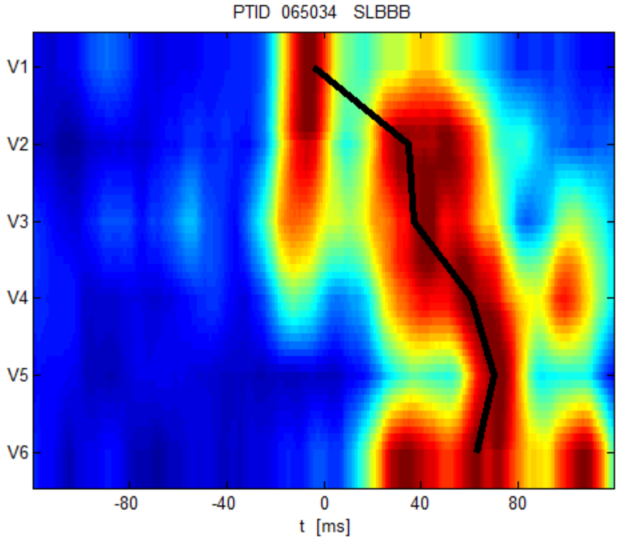
PTID 065034 SLBBB
V1
V2
V3
V4
V5
V6
-80
-40
0
40
80
t [ms]

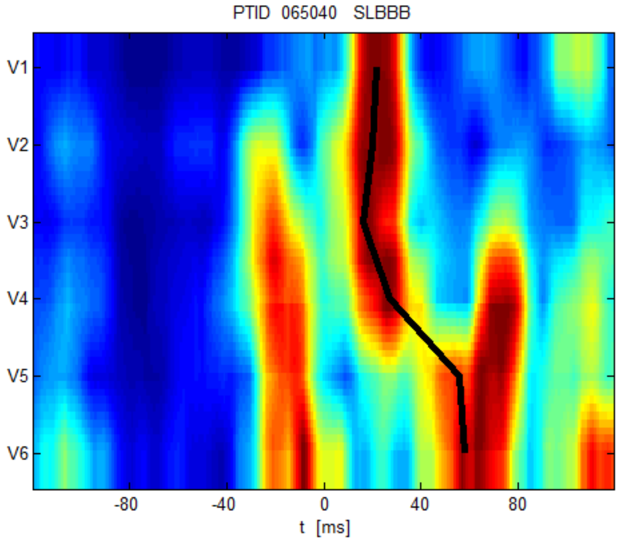
PTID 065040 SLBBB
V1
V2
V3
V4
V5
V6
-80
-40
0
40
80
t [ms]

PTID 065044 SLBBB

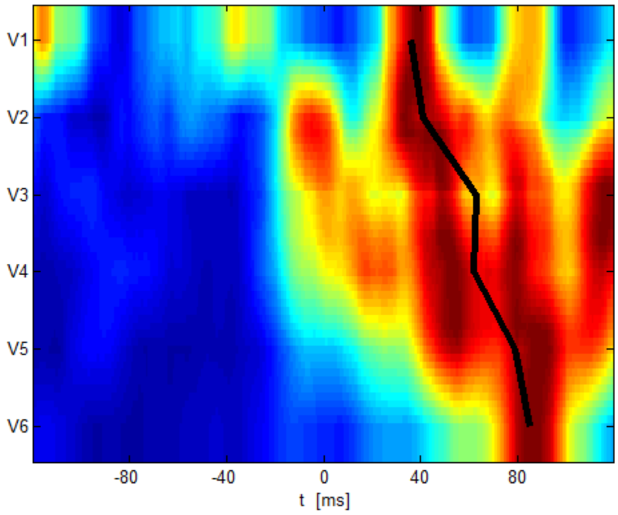

PTID 065045 non SLBBB

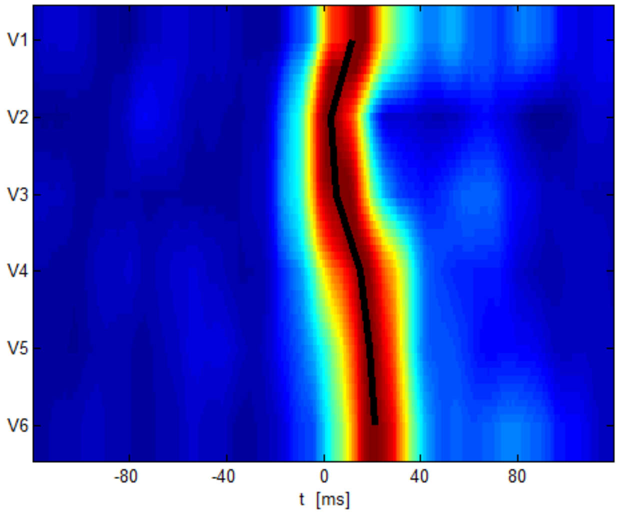

PTID 065049 non SLBBB

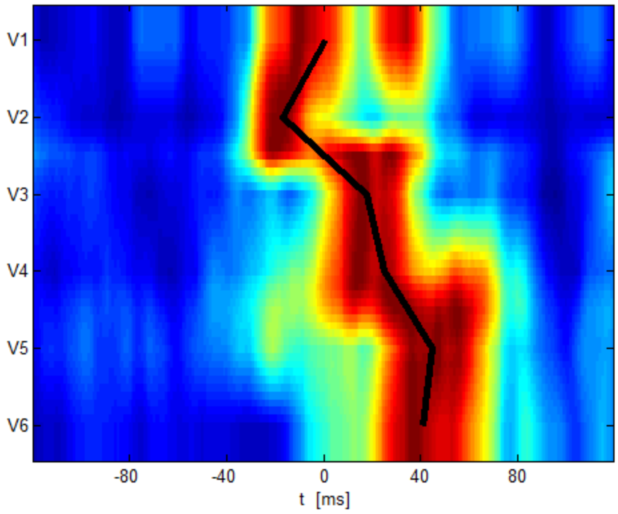

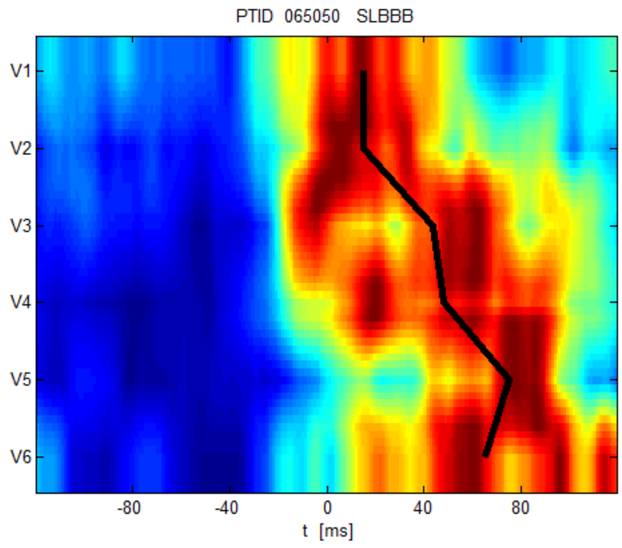
PTID 065050 SLBBB
V1
V2
V3
V4
V5
V6
-80
-40
0
40
80
t [ms]

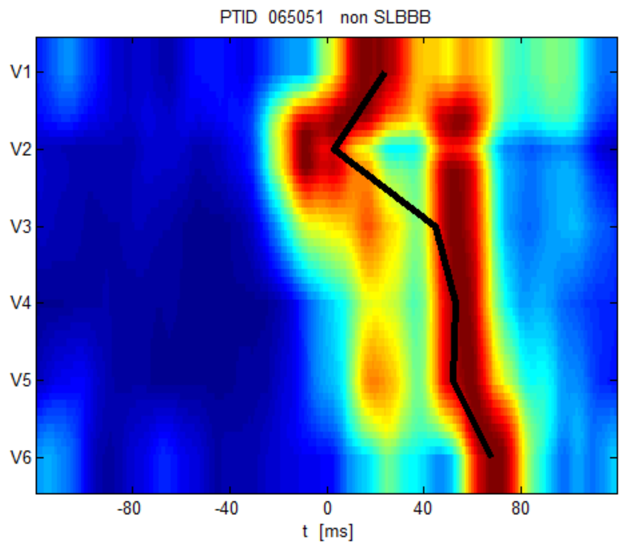
PTID 065051 non SLBBB
V1
V2
V3
V4
V5
V6
-80
-40
0
40
80
t [ms]

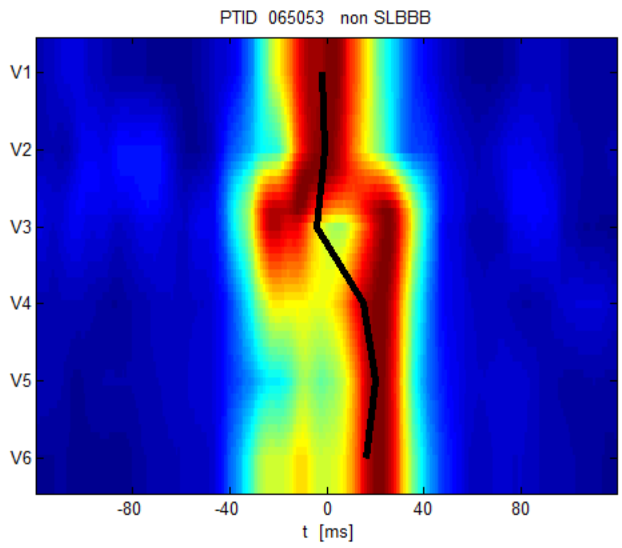
PTID 065053 non SLBBB
V1
V2
V3
V4
V5
V6
-80
-40
0
40
80
t [ms]

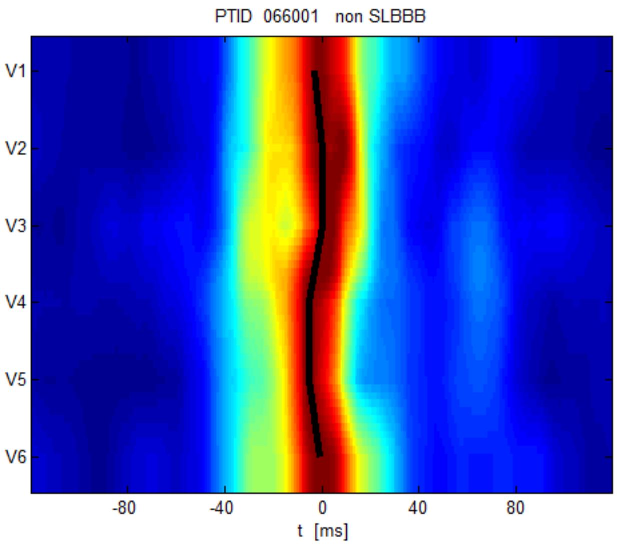
PTID 066001 non SLBBB
V1
V2
V3
V4
V5
V6
-80
-40
0
40
80
t [ms]

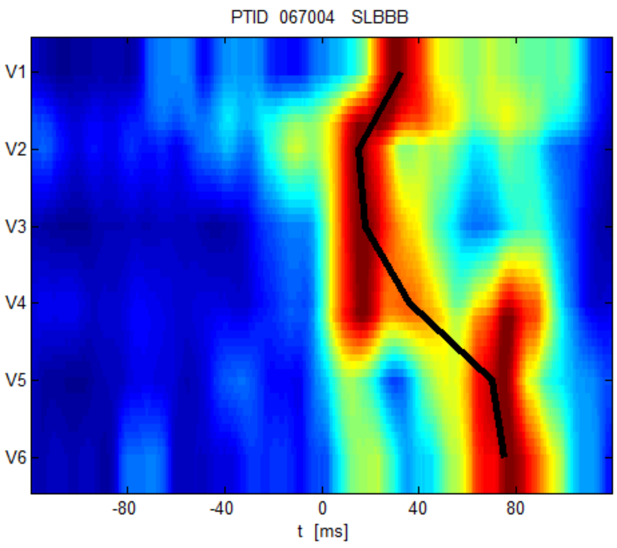
PTID 067004 SLBBB
V1
V2
V3
V4
V5
V6
-80
-40
0
40
80
t [ms]

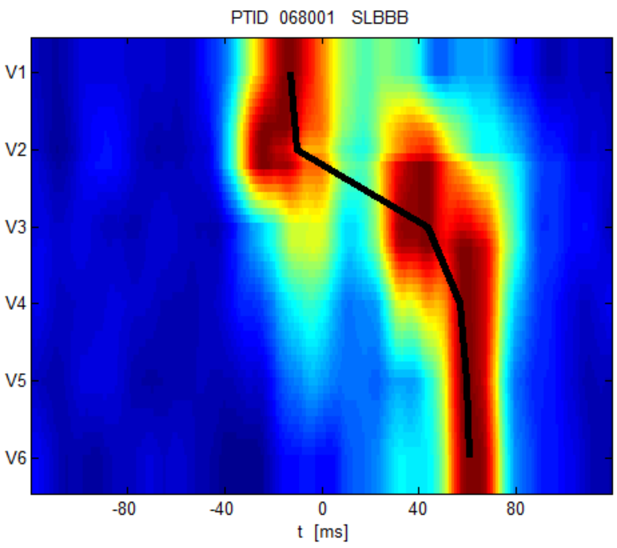
PTID 068001 SLBBB
V1
V2
V3
V4
V5
V6
-80
-40
0
40
80
t [ms]

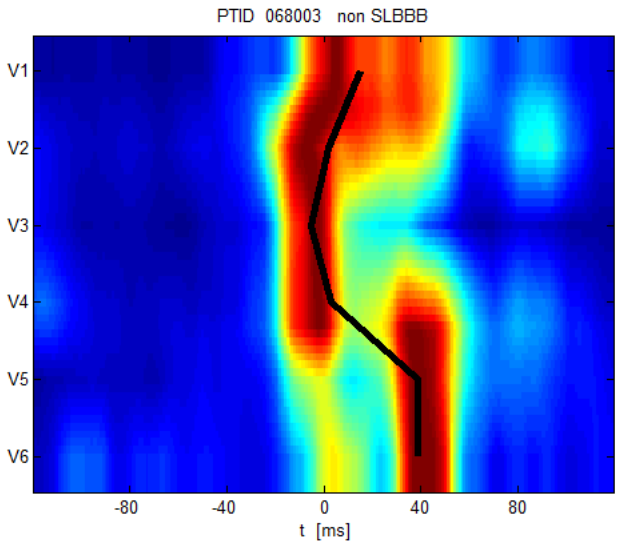
PTID 068003 non SLBBB
V1
V2
V3
V4
V5
V6
-80
-40
0
40
80
t [ms]

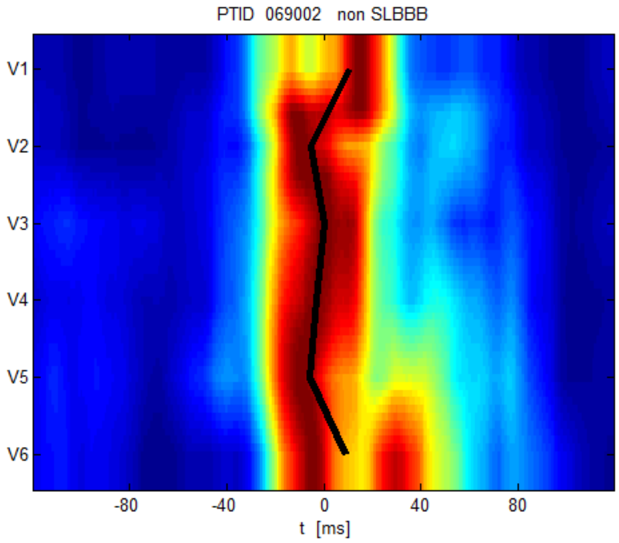
PTID 069002 non SLBBB
V1
V2
V3
V4
V5
V6
-80
-40
0
40
80
t [ms]

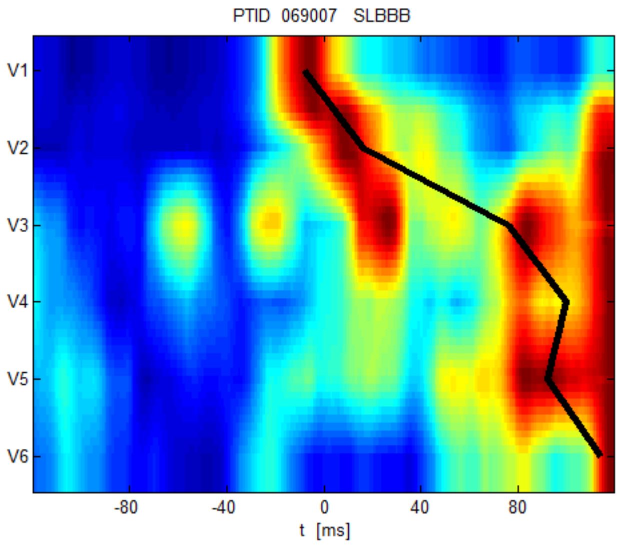
PTID 069007 SLBBB
V1
V2
V3
V4
V5
V6
-80
-40
0
40
80
t [ms]

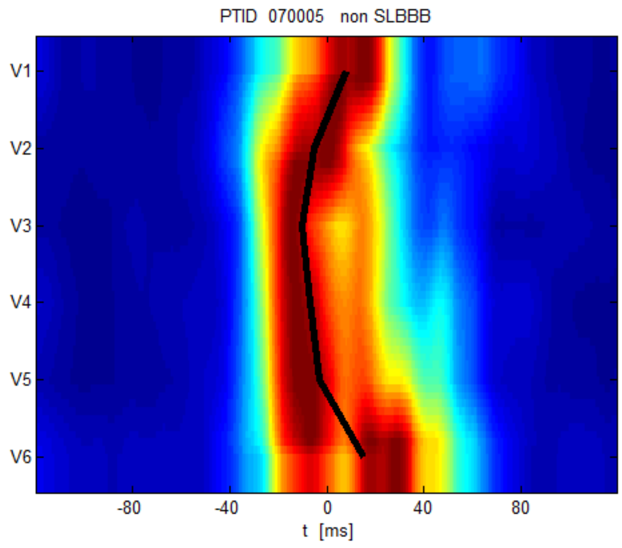
PTID 070005 non SLBBB
V1
V2
V3
V4
V5
V6
-80
-40
0
40
80
t [ms]

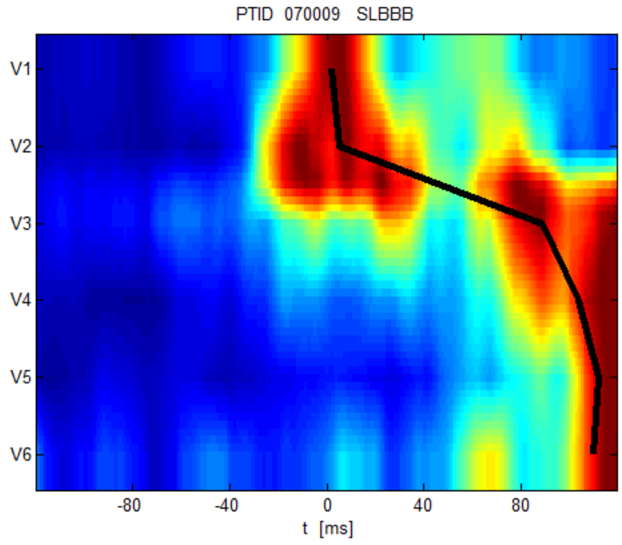
PTID 070009 SLBBB
V1
V2
V3
V4
V5
V6
-80
-40
0
40
80
t [ms]

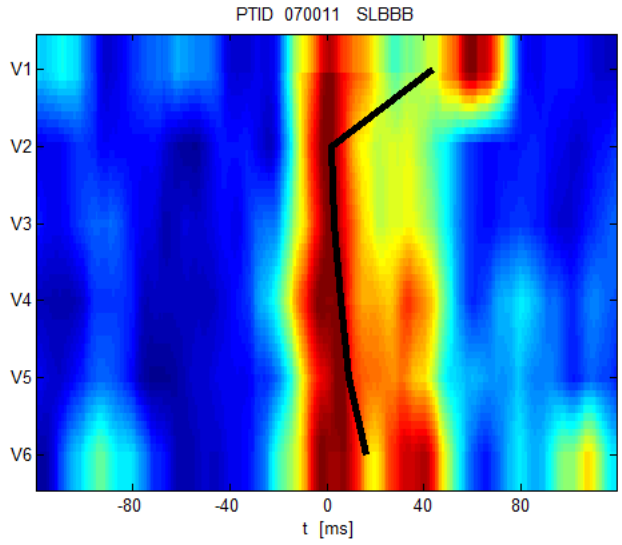
PTID 070011 SLBBB
V1
V2
V3
V4
V5
V6
-80
-40
0
40
80
t [ms]

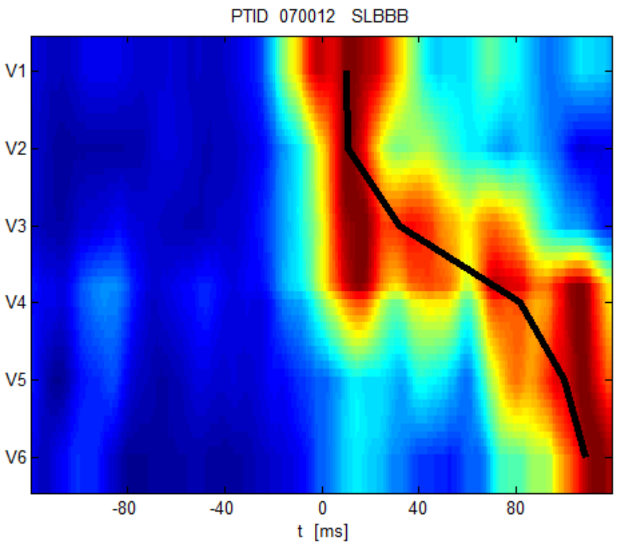
PTID 070012 SLBBB
V1
V2
V3
V4
V5
V6
-80
-40
0
40
80
t [ms]

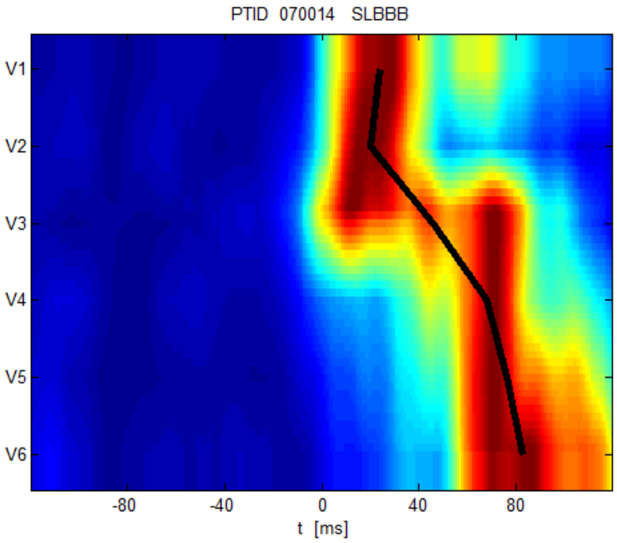
PTID 070014 SLBBB
V1
V2
V3
V4
V5
V6
-80
-40
0
40
80
t [ms]

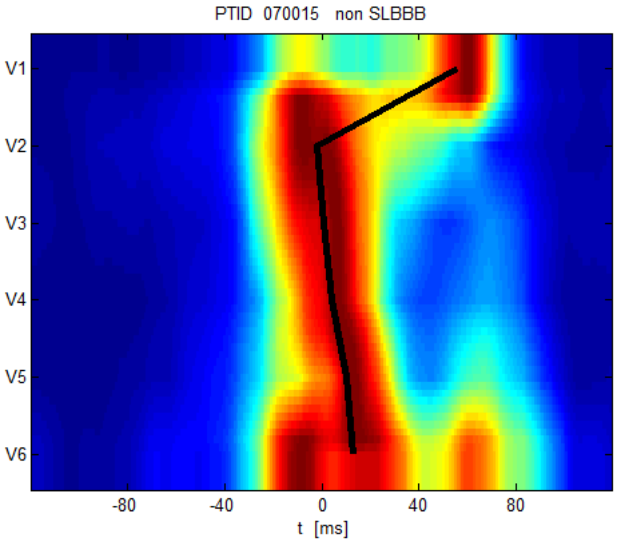
PTID 070015 non SLBBB
V1
V2
V3
V4
V5
V6
-80
-40
0
40
80
t [ms]

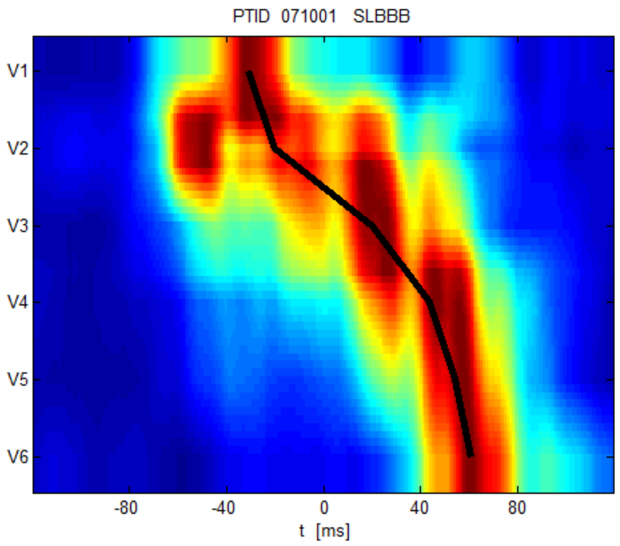
PTID 071001 SLBBB
V1
V2
V3
V4
V5
V6
-80
-40
0
40
80
t [ms]

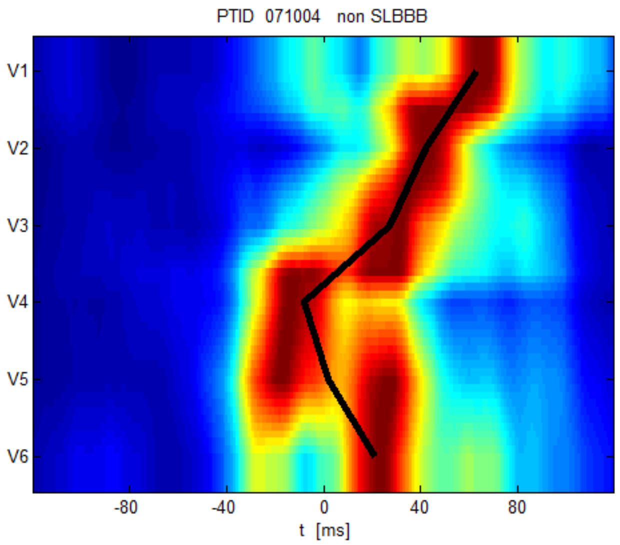
PTID 071004 non SLBBB
V1
V2
V3
V4
V5
V6
-80
-40
0
40
80
t [ms]

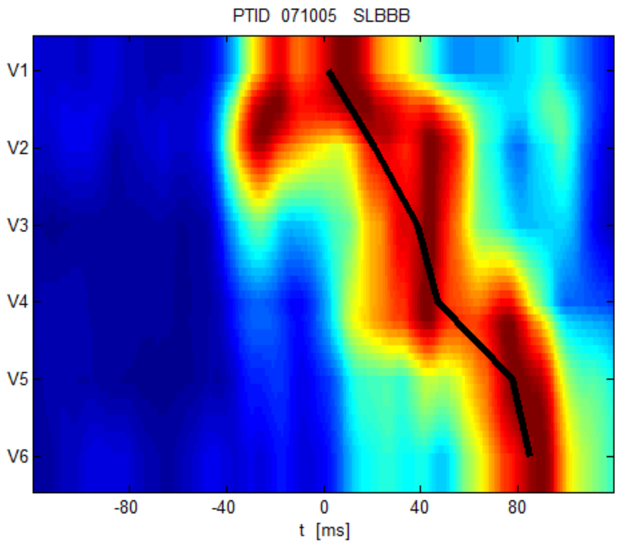
PTID 071005 SLBBB
V1
V2
V3
V4
V5
V6
-80
-40
0
40
80
t [ms]

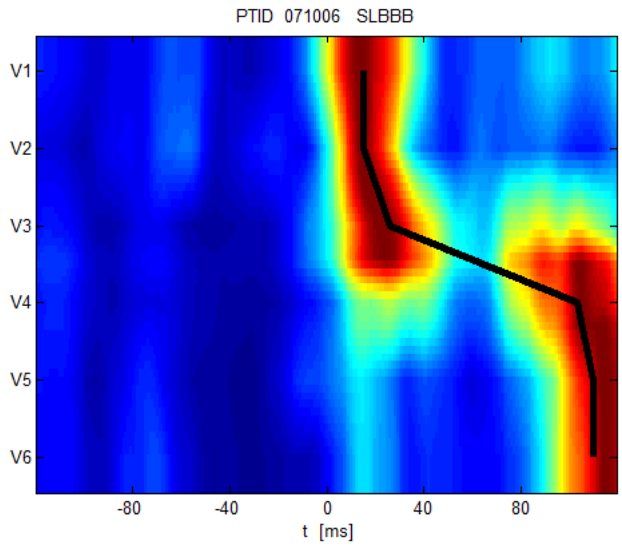
PTID 071006 SLBBB
V1
V2
V3
V4
V5
V6
-80
-40
0
40
80
t [ms]

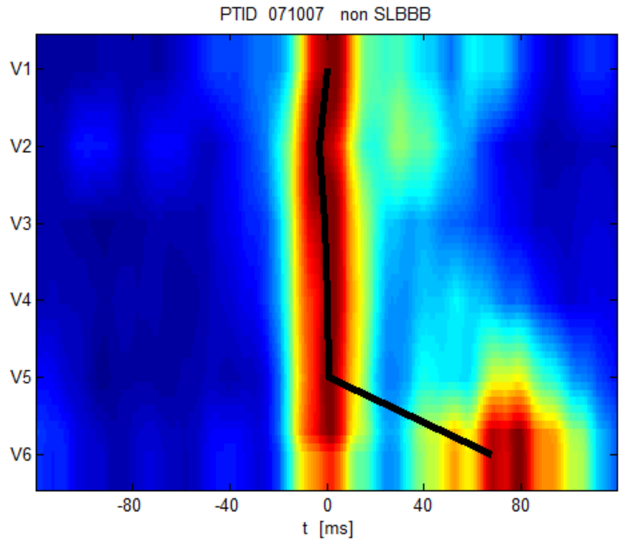
PTID 071007 non SLBBB
V1
V2
V3
V4
V5
V6
-80
-40
0
40
80
t [ms]

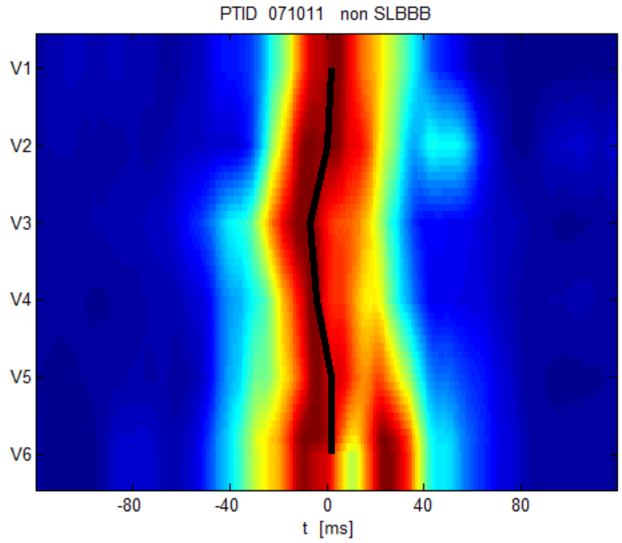
PTID 071011 non SLBBB
V1
V2
V3
V4
V5
V6
-80
-40
0
40
80
t [ms]

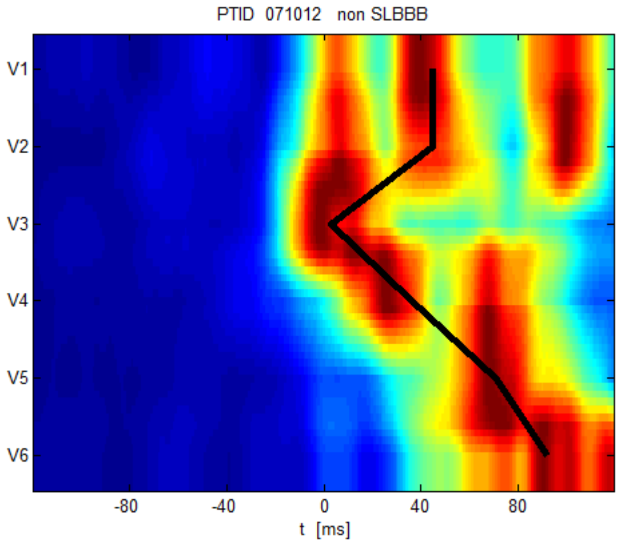
PTID 071012 non SLBBB
V1
V2
V3
V4
V5
V6
-80
-40
0
40
80
t [ms]

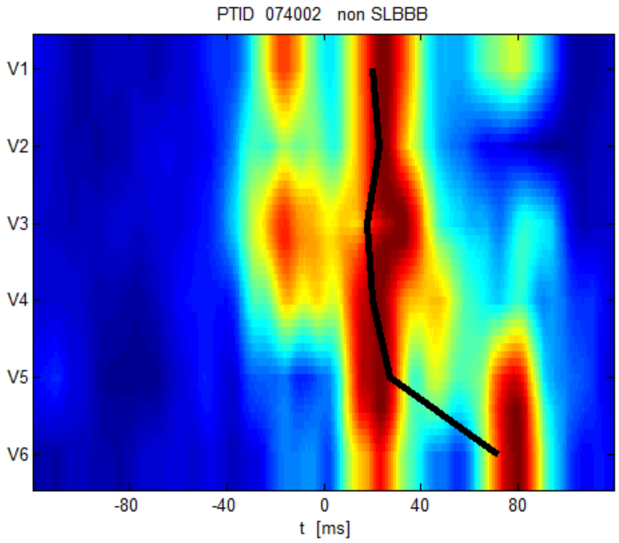
PTID 074002 non SLBBB
V1
V2
V3
V4
V5
V6
-80
-40
0
40
80
t [ms]

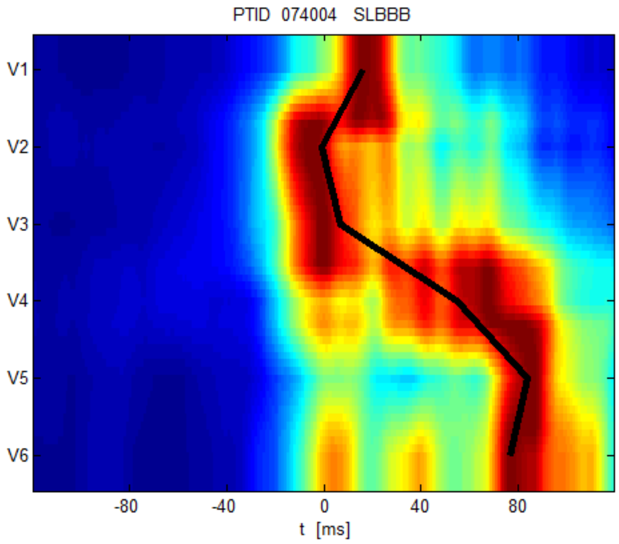
PTID 074004 SLBBB
V1
V2
V3
V4
V5
V6
-80
-40
0
40
80
t [ms]

PTID 074005 SLBBB

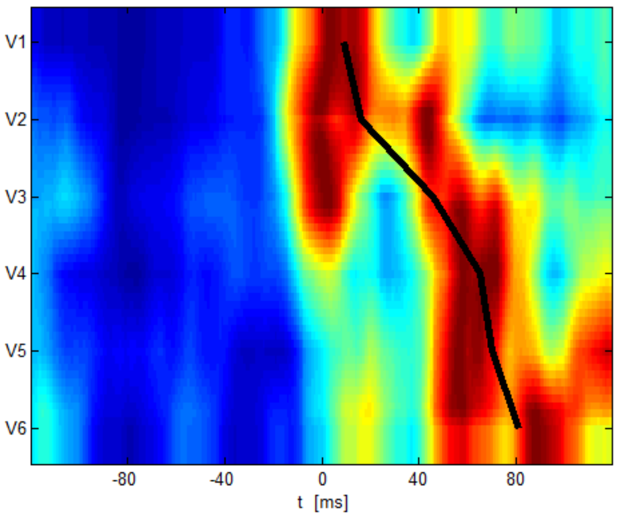

PTID 074014 non SLBBB

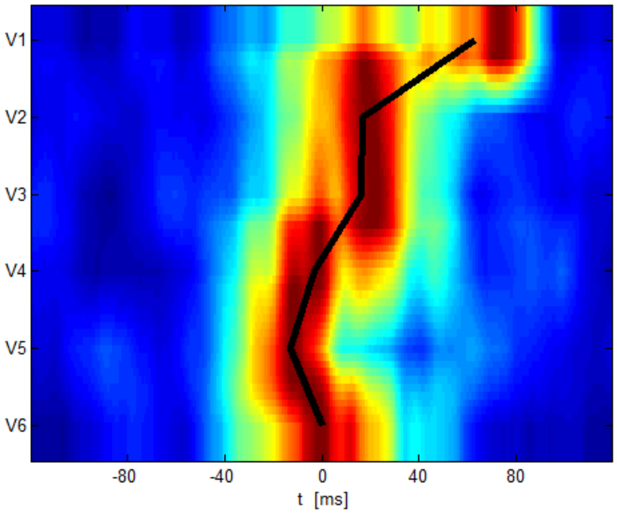

PTID 074015 SLBBB

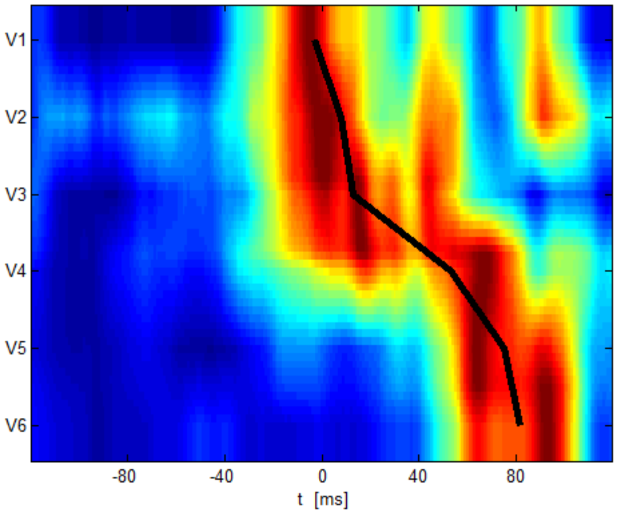

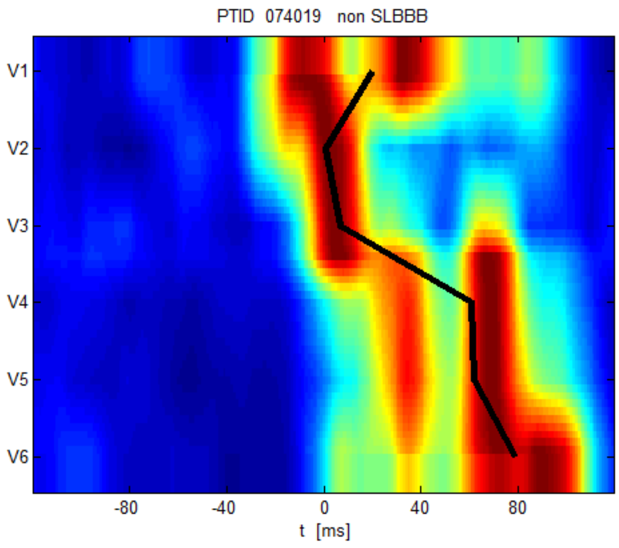
PTID 074019 non SLBBB
V1
V2
V3
V4
V5
V6
-80
-40
0
40
80
t [ms]

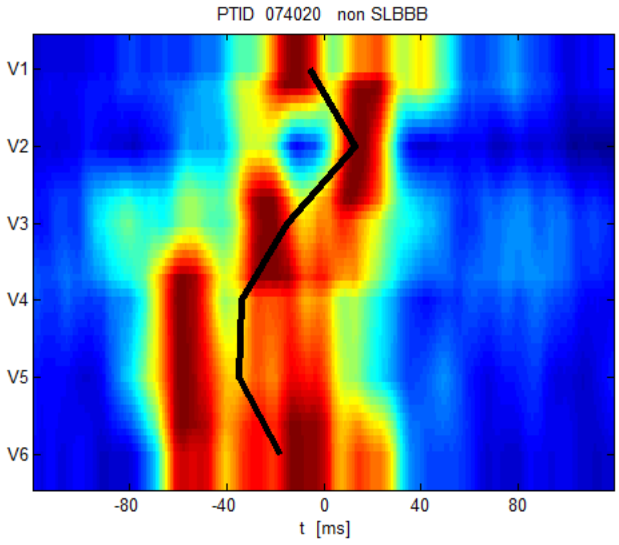
PTID 074020 non SLBBB
V1
V2
V3
V4
V5
V6
-80
-40
0
40
80
t [ms]

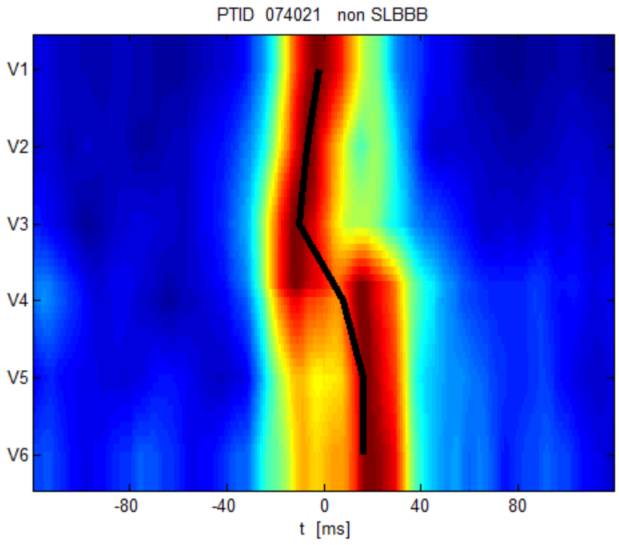
PTID 074021 non SLBBB
V1
V2
V3
V4
V5
V6
-80
-40
0
40
80
t [ms]

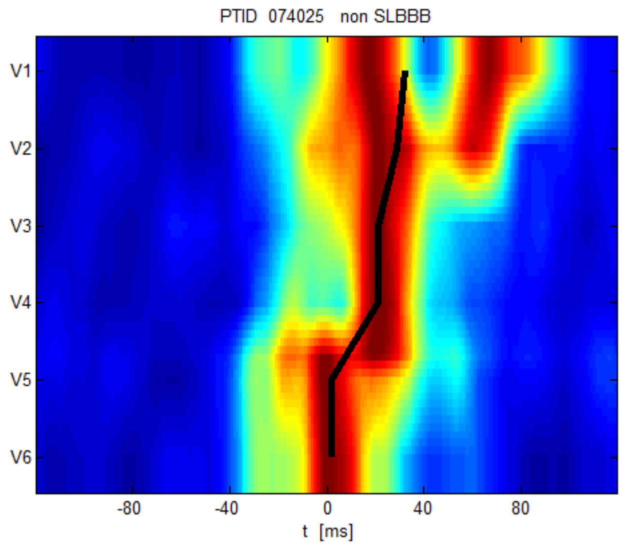
PTID 074025 non SLBBB
V1
V2
V3
V4
V5
V6
-80
-40
0
40
80
t [ms]

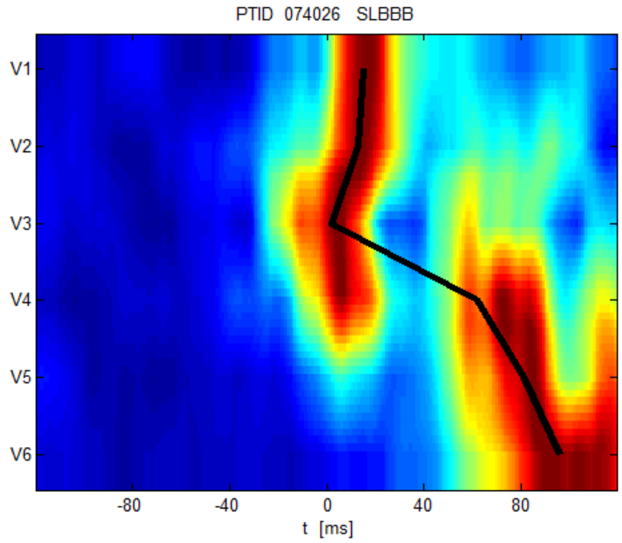
PTID 074026 SLBBB
V1
V2
V3
V4
V5
V6
-80
-40
0
40
80
t [ms]

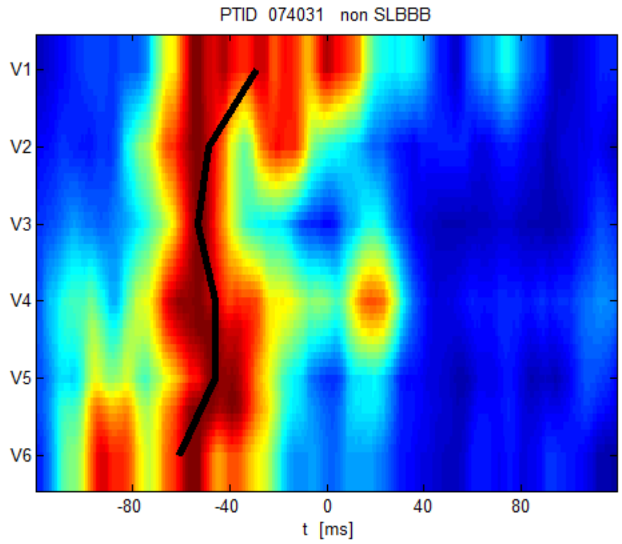
PTID 074031 non SLBBB
V1
V2
V3
V4
V5
V6
-80
-40
0
40
80
t [ms]

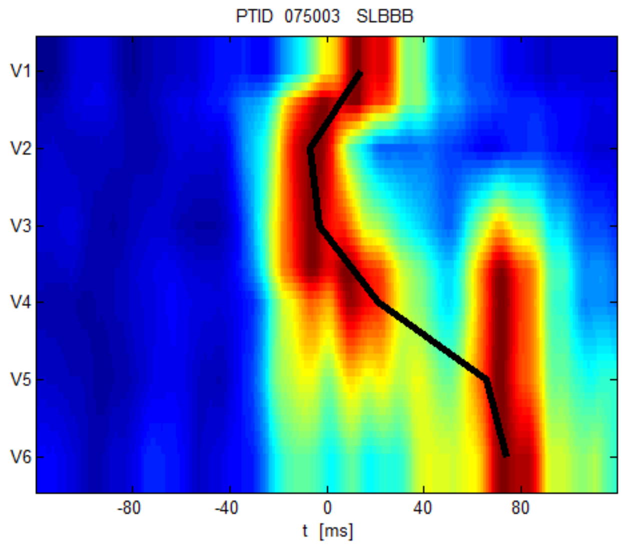
PTID 075003 SLBBB
V1
V2
V3
V4
V5
V6
-80
-40
0
40
80
t [ms]

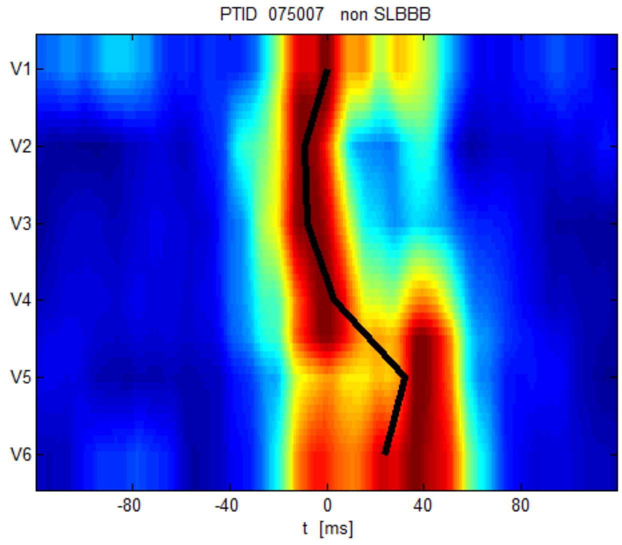
PTID 075007 non SLBBB
V1
V2
V3
V4
V5
V6
-80
-40
0
40
80
t [ms]

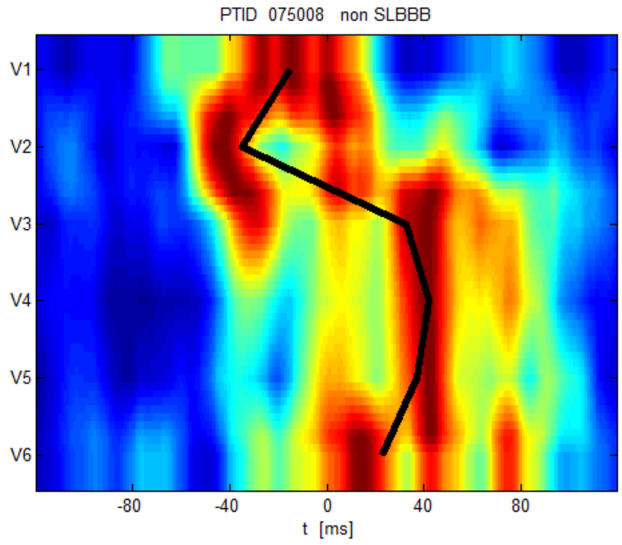
PTID 075008 non SLBBB
V1
V2
V3
V4
V5
V6
-80
-40
0
40
80
t [ms]

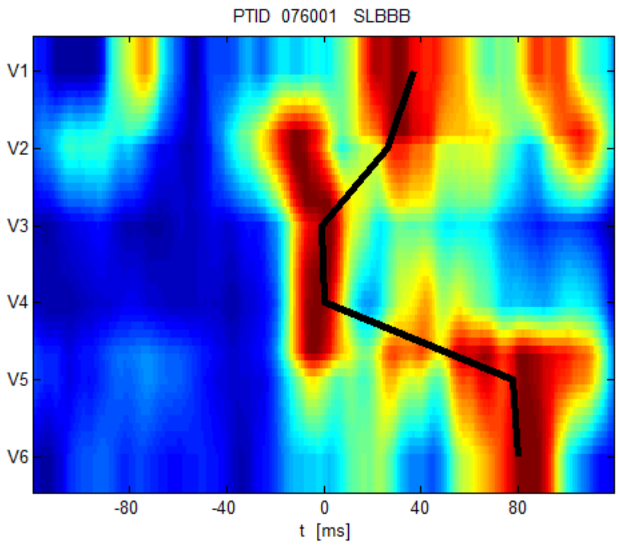
PTID 076001 SLBBB
V1
V2
V3
V4
V5
V6
-80
-40
0
40
80
t [ms]

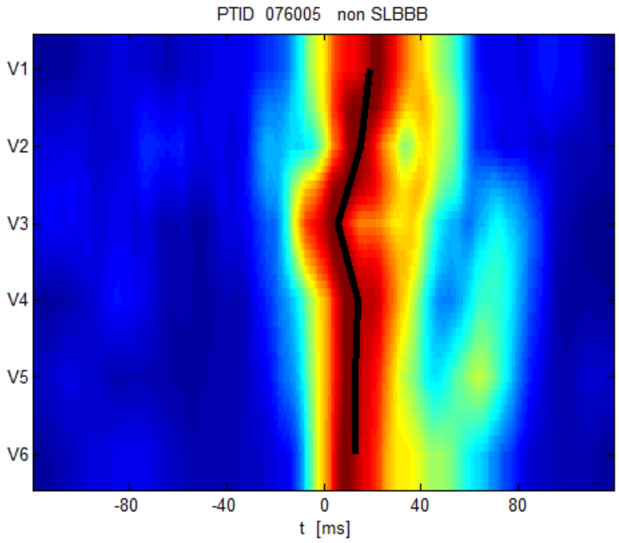
PTID 076005 non SLBBB
V1
V2
V3
V4
V5
V6
-80
-40
0
40
80
t [ms]

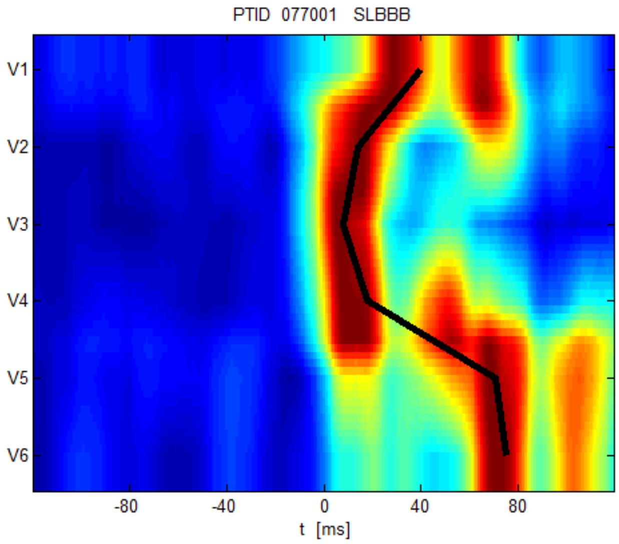
PTID 077001 SLBBB
V1
V2
V3
V4
V5
V6
-80
-40
0
40
80
t [ms]

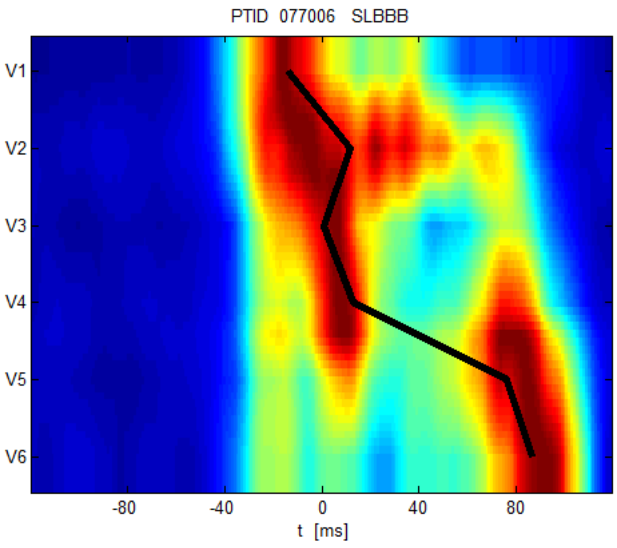
PTID 077006 SLBBB
V1
V2
V3
V4
V5
V6
-80
-40
0
40
80
t [ms]

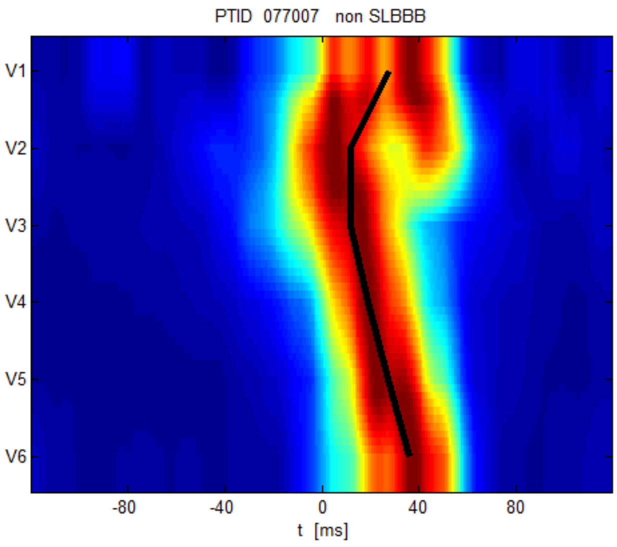
PTID 077007 non SLBBB
V1
V2
V3
V4
V5
V6
-80
-40
0
40
80
t [ms]

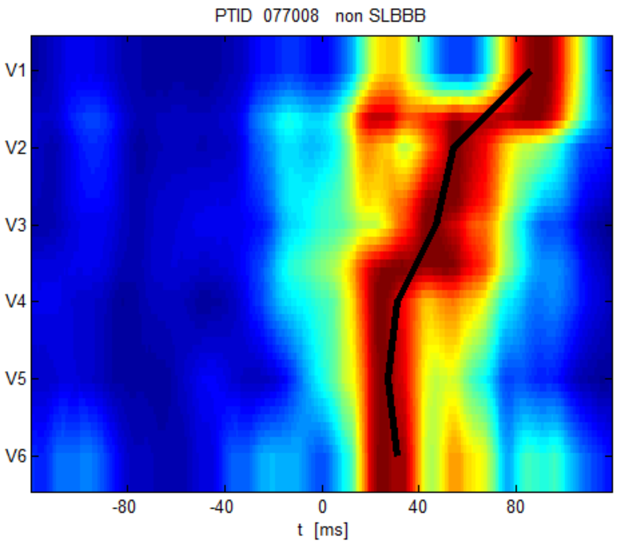
PTID 077008 non SLBBB
V1
V2
V3
V4
V5
V6
-80
-40
0
40
80
t [ms]

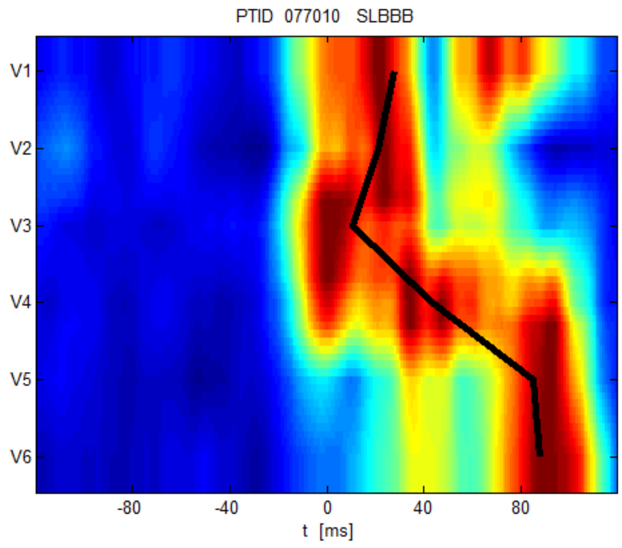
PTID 077010 SLBBB
V1
V2
V3
V4
V5
V6
-80
-40
0
40
80
t [ms]

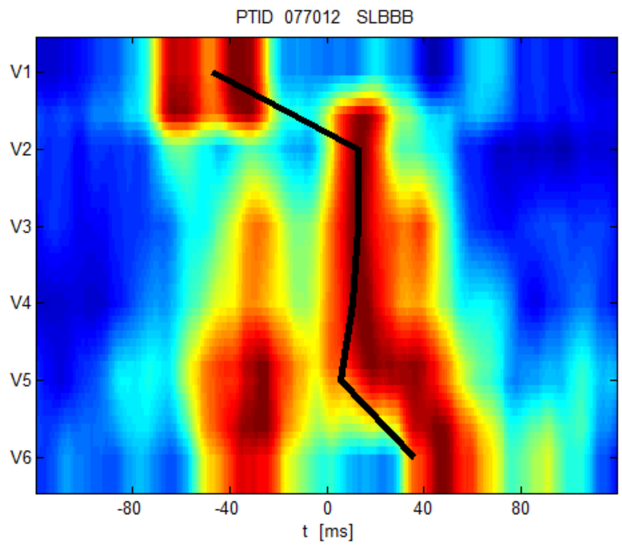
PTID 077012 SLBBB
V1
V2
V3
V4
V5
V6
-80
-40
0
40
80
t [ms]

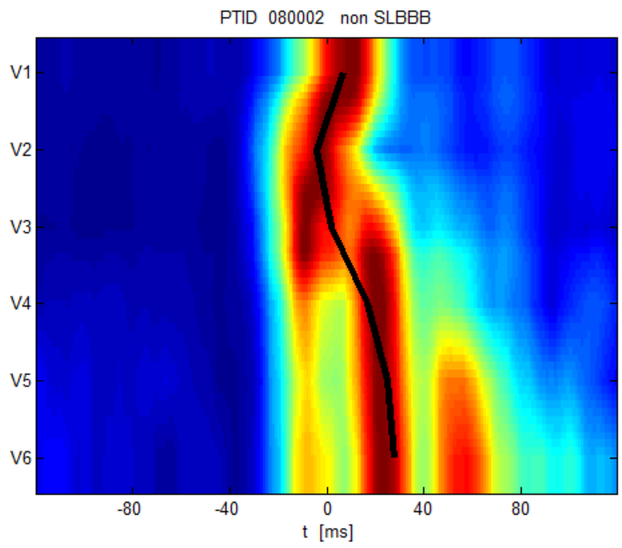
PTID 080002 non SLBBB
V1
V2
V3
V4
V5
V6
-80
-40
0
40
80
t [ms]

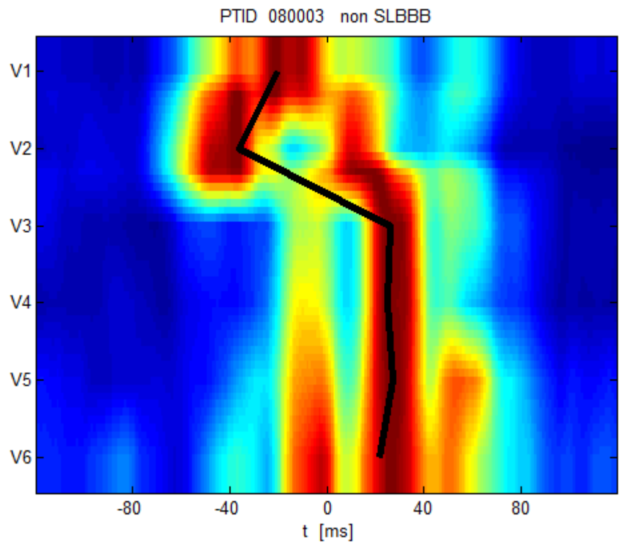
PTID 080003 non SLBBB
V1
V2
V3
V4
V5
V6
-80
-40
0
40
80
t [ms]

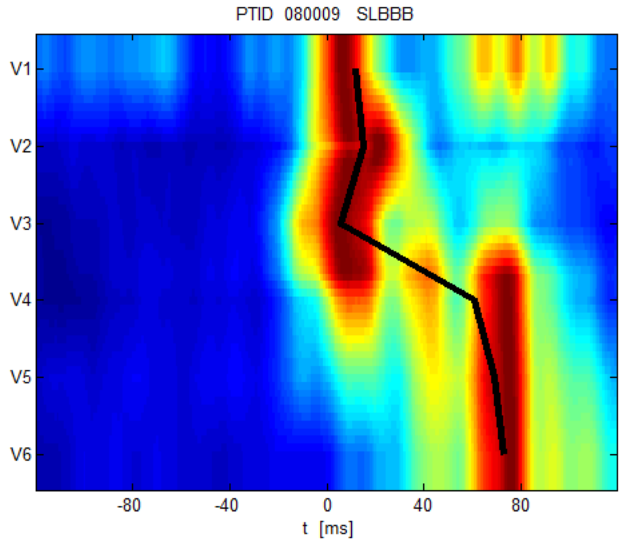
PTID 080009 SLBBB
V1
V2
V3
V4
V5
V6
-80
-40
0
40
80
t [ms]

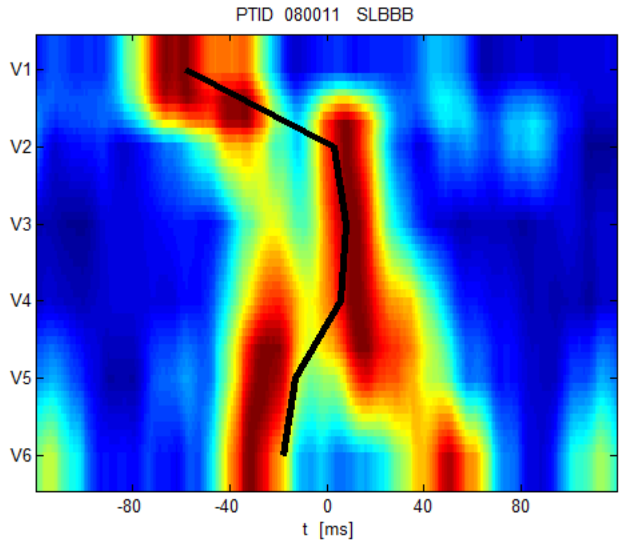
PTID 080011 SLBBB
V1
V2
V3
V4
V5
V6
-80
-40
0
40
80
t [ms]

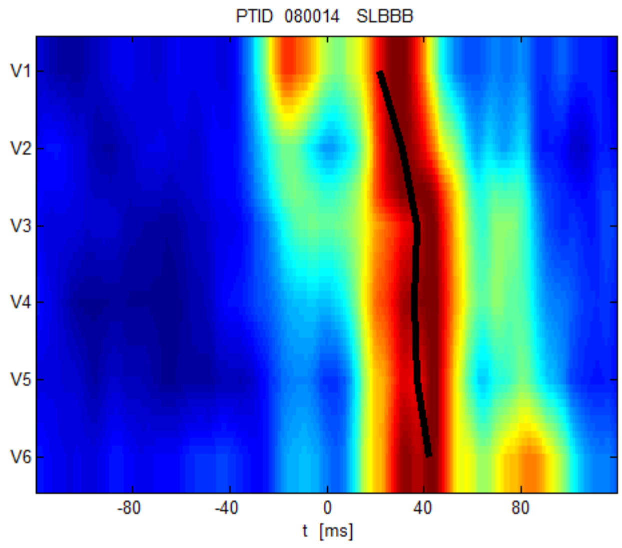
PTID 080014 SLBBB
V1
V2
V3
V4
V5
V6
-80
-40
0
40
80
t [ms]

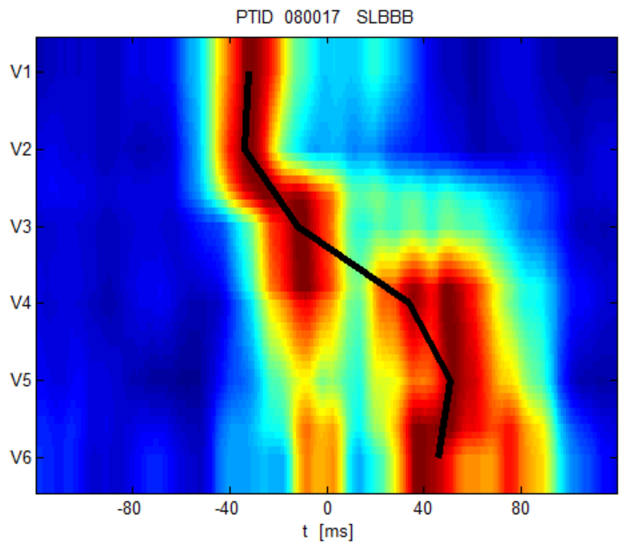
PTID 080017 SLBBB
V1
V2
V3
V4
V5
V6
-80
-40
0
40
80
t [ms]

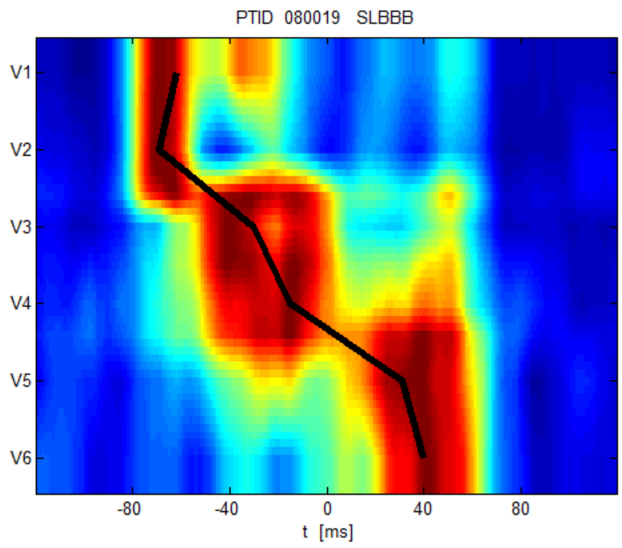
PTID 080019 SLBBB
V1
V2
V3
V4
V5
V6
-80
-40
0
40
80
t [ms]

PTID 080020 non SLBBB

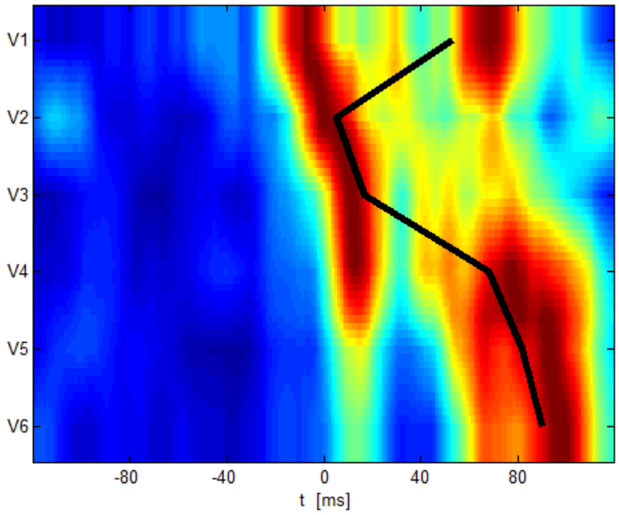

PTID 080031 non SLBBB

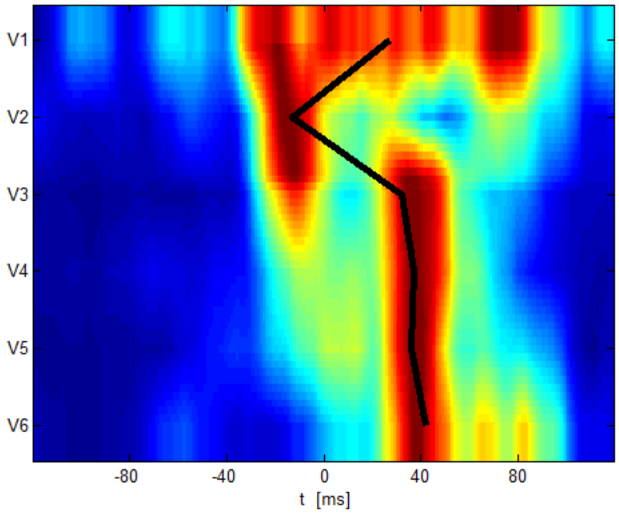

PTID 080033 SLBBB

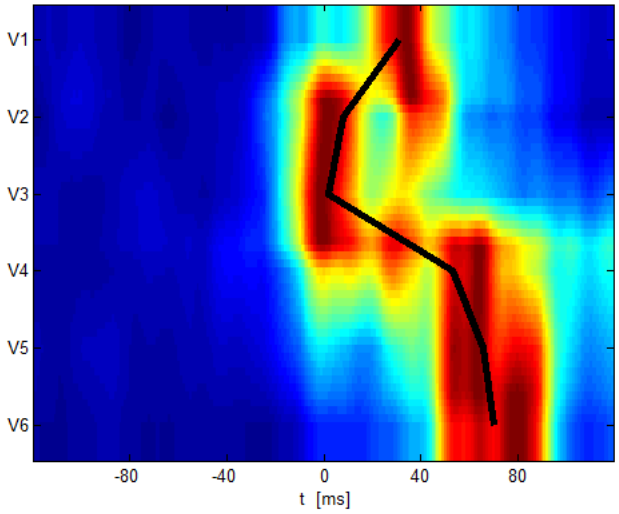

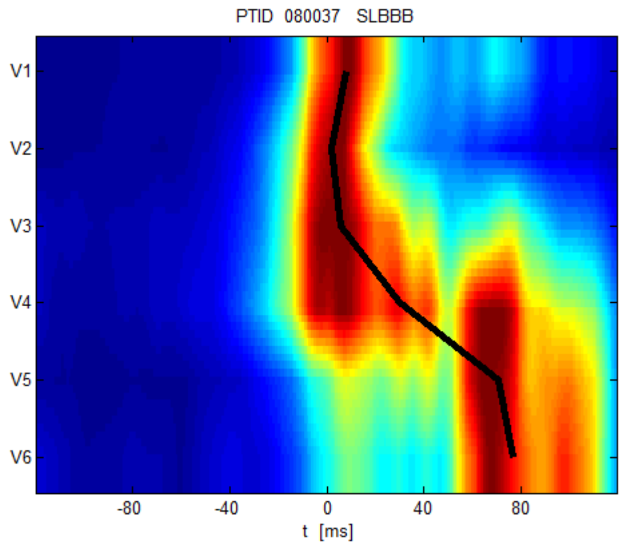
PTID 080037 SLBBB
V1
V2
V3
V4
V5
V6
-80
-40
0
40
80
t [ms]

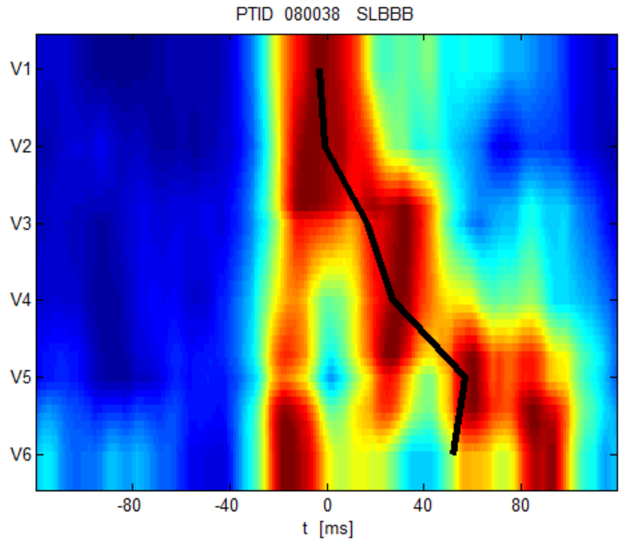
PTID 080038 SLBBB
V1
V2
V3
V4
V5
V6
-80
-40
0
40
80
t [ms]

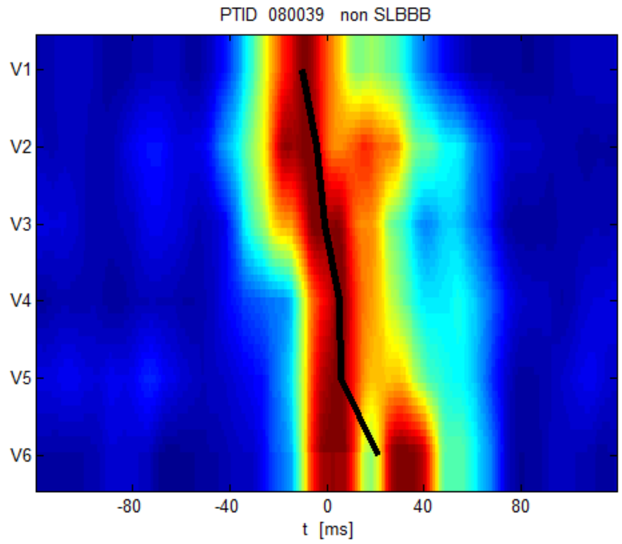
PTID 080039 non SLBBB
V1
V2
V3
V4
V5
V6
-80
-40
0
40
80
t [ms]

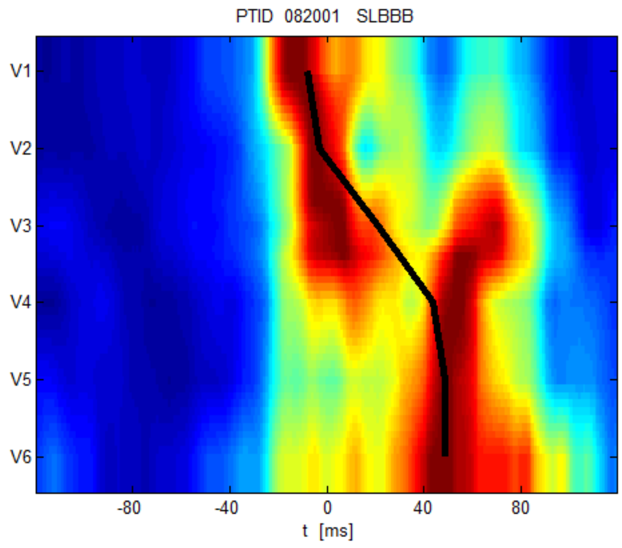
PTID 082001 SLBBB
V1
V2
V3
V4
V5
V6
-80
-40
0
40
80
t [ms]

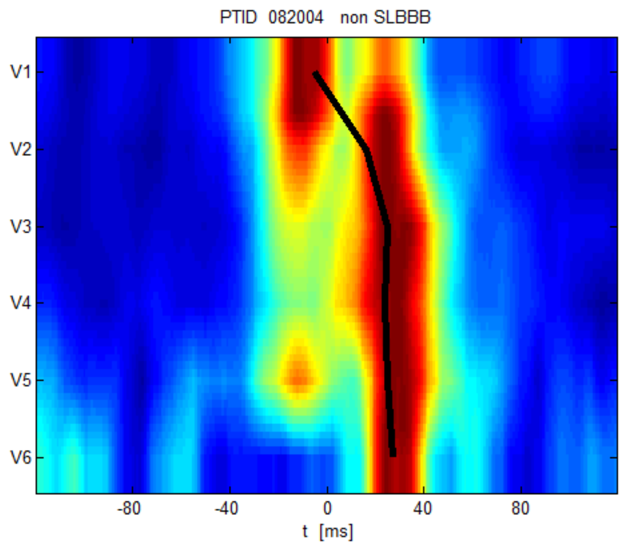
PTID 082004 non SLBBB
V1
V2
V3
V4
V5
V6
-80
-40
0
40
80
t [ms]

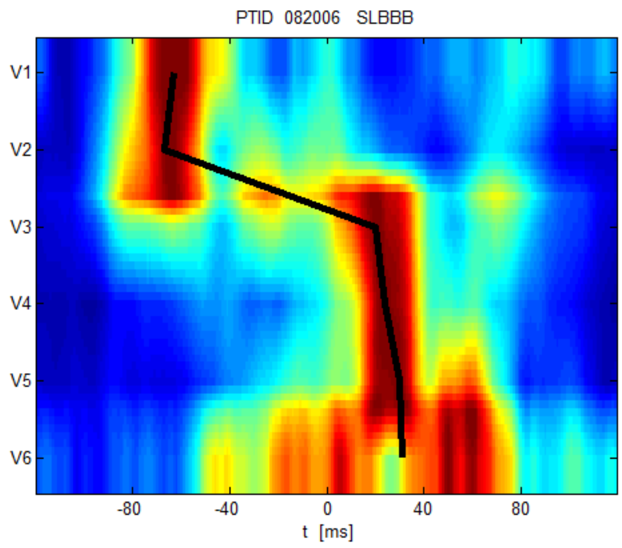
PTID 082006 SLBBB
V1
V2
V3
V4
V5
V6
-80
-40
0
40
80
t [ms]

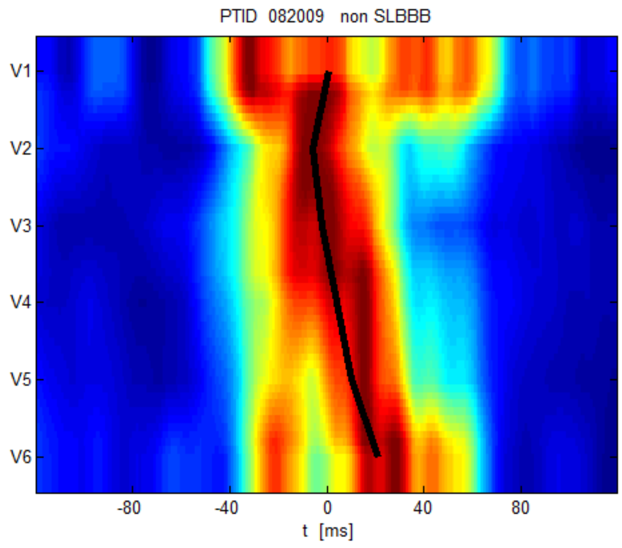
PTID 082009 non SLBBB
V1
V2
V3
V4
V5
V6
-80
-40
0
40
80
t [ms]

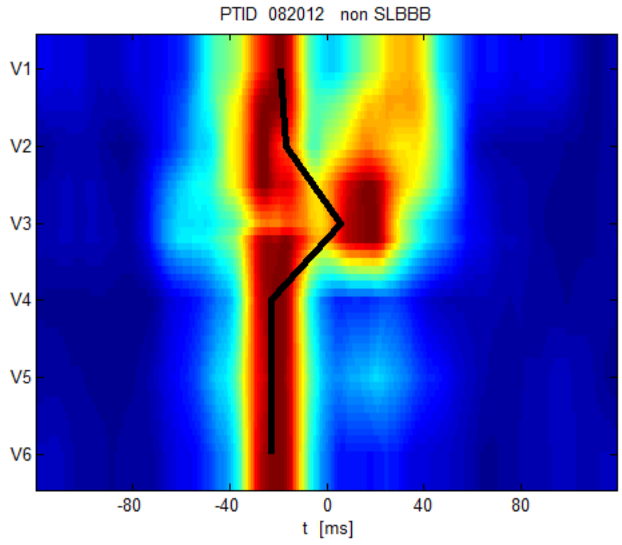
PTID 082012 non SLBBB
V1
V2
V3
V4
V5
V6
-80
-40
0
40
80
t [ms]

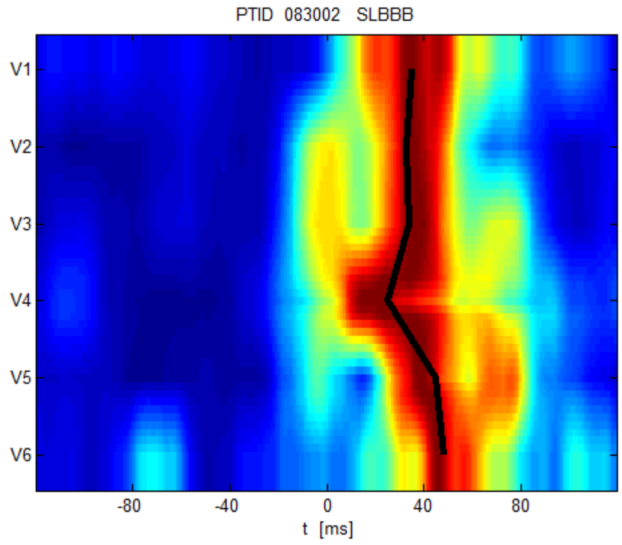
PTID 083002 SLBBB
V1
V2
V3
V4
V5
V6
-80
-40
0
40
80
t [ms]

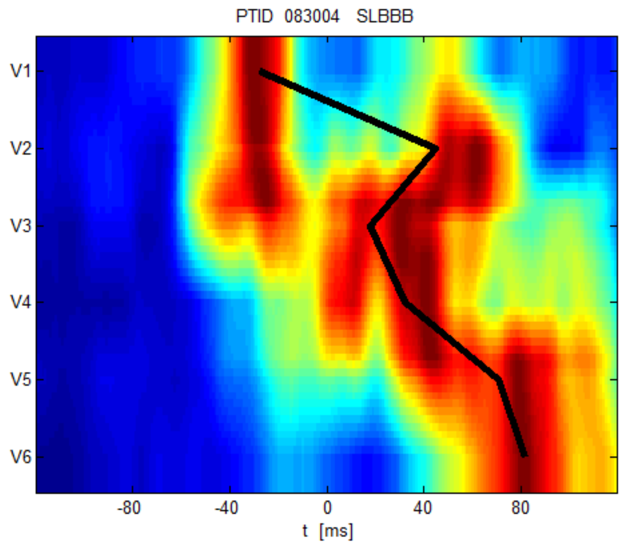
PTID 083004 SLBBB
V1
V2
V3
V4
V5
V6
-80
-40
0
40
80
t [ms]

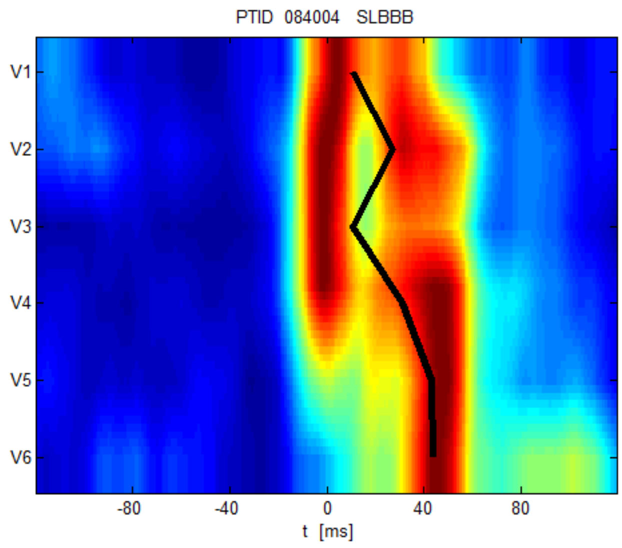
PTID 084004 SLBBB
V1
V2
V3
V4
V5
V6
-80
-40
0
40
80
t [ms]

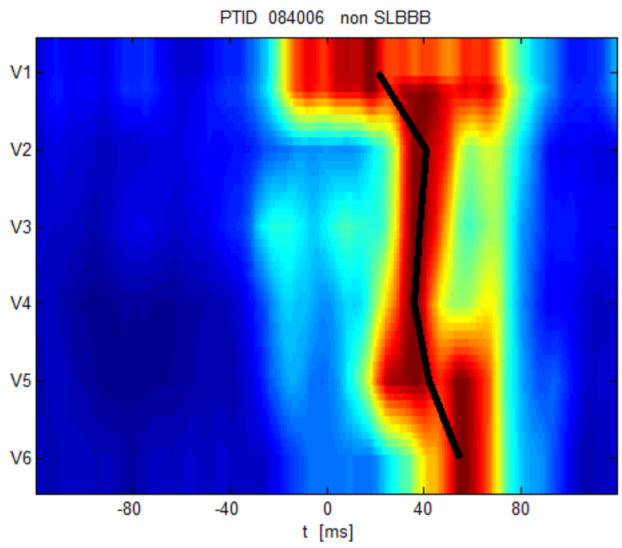
PTID 084006 non SLBBB
V1
V2
V3
V4
V5
V6
-80
-40
0
40
80
t [ms]

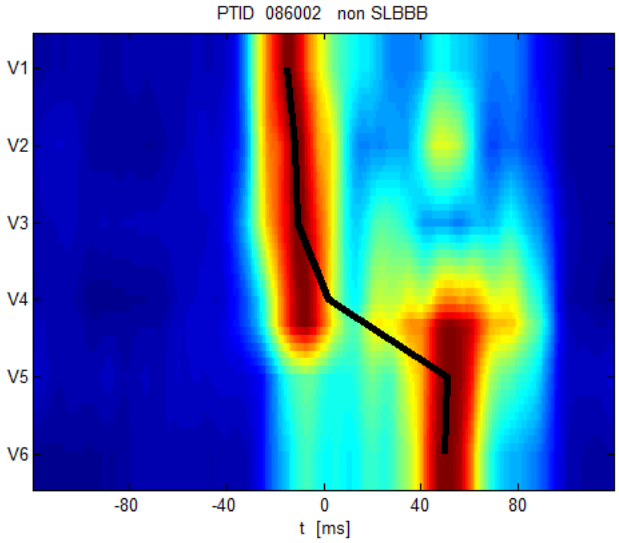
PTID 086002 non SLBBB
V1
V2
V3
V4
V5
V6
-80
-40
0
40
80
t [ms]

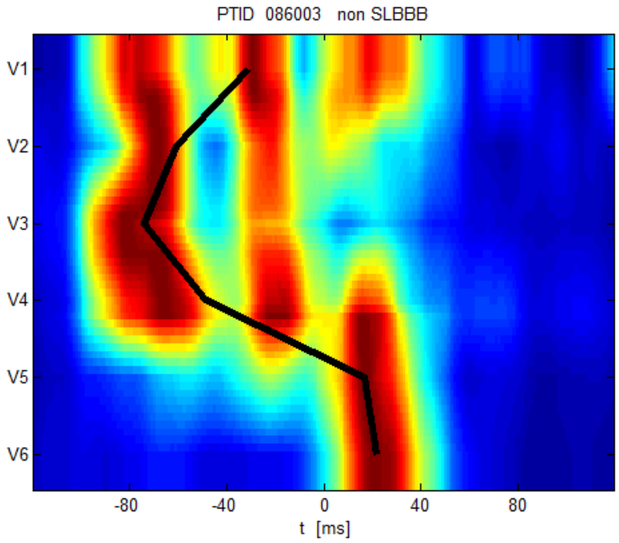
PTID 086003 non SLBBB
V1
V2
V3
V4
V5
V6
-80
-40
0
40
80
t [ms]

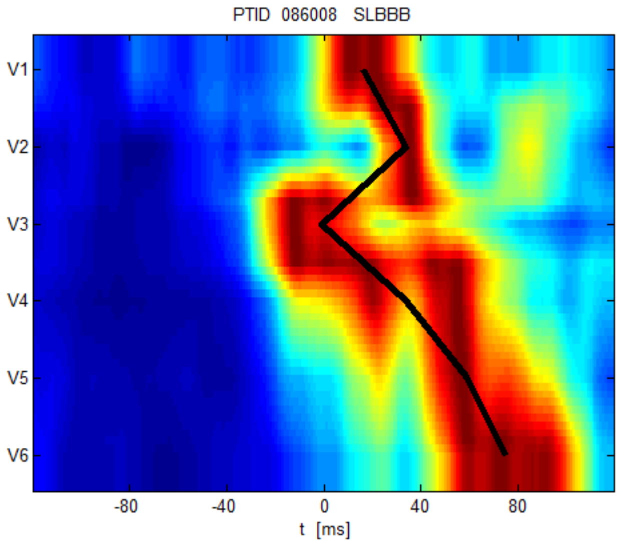
PTID 086008 SLBBB
V1
V2
V3
V4
V5
V6
-80
-40
0
40
80
t [ms]

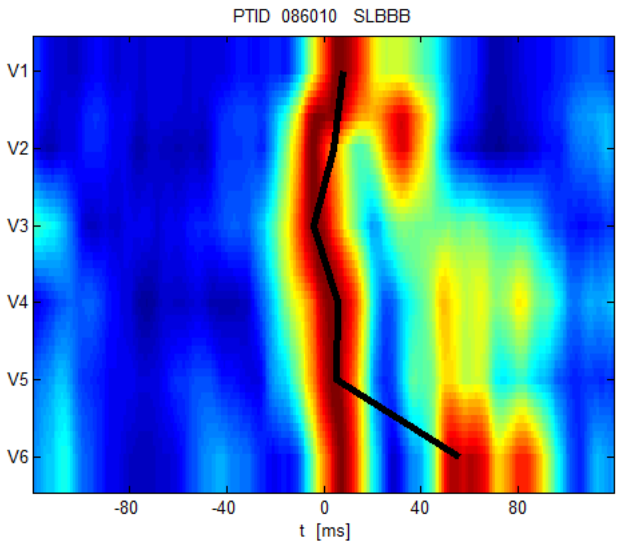
PTID 086010 SLBBB
V1
V2
V3
V4
V5
V6
-80
-40
0
40
80
t [ms]

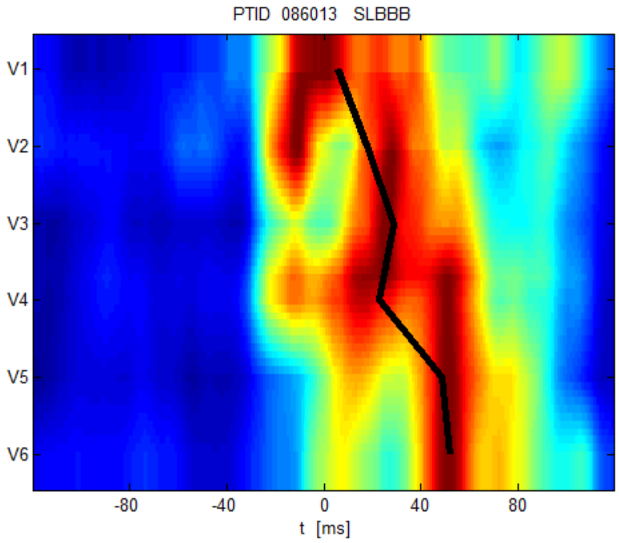
PTID 086013 SLBBB
V1
V2
V3
V4
V5
V6
-80
-40
0
40
80
t [ms]

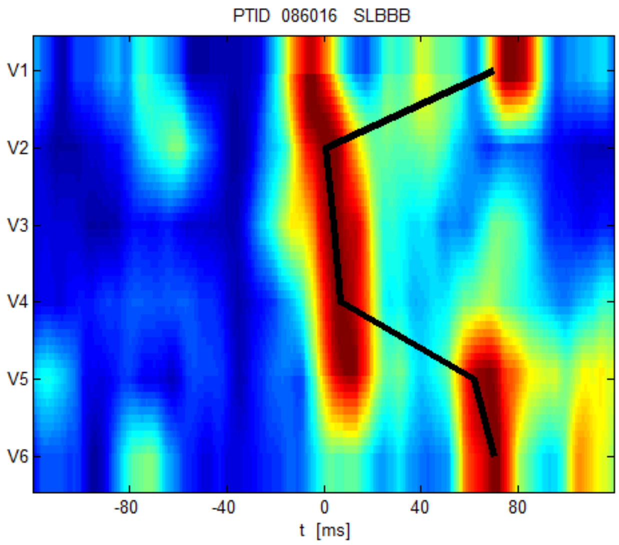
PTID 086016 SLBBB
V1
V2
V3
V4
V5
V6
-80
-40
0
40
80
t [ms]

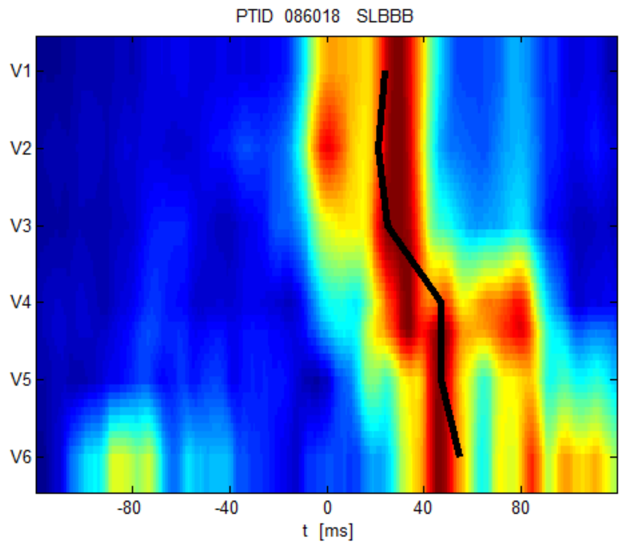
PTID 086018 SLBBB
V1
V2
V3
V4
V5
V6
-80
-40
0
40
80
t [ms]

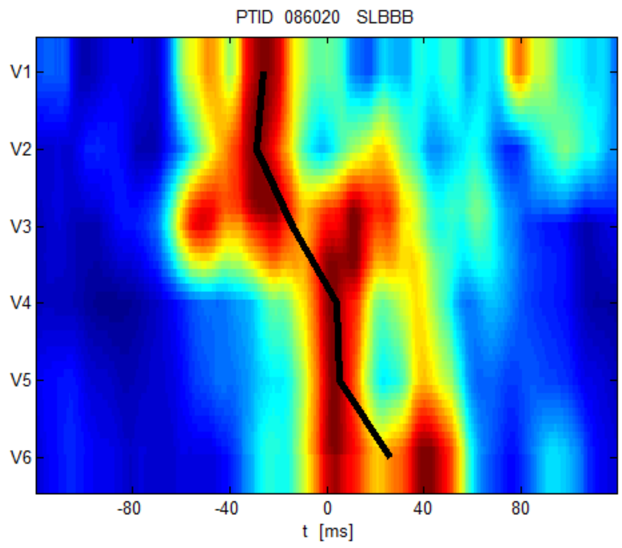
PTID 086020 SLBBB
V1
V2
V3
V4
V5
V6
-80
-40
0
40
80
t [ms]

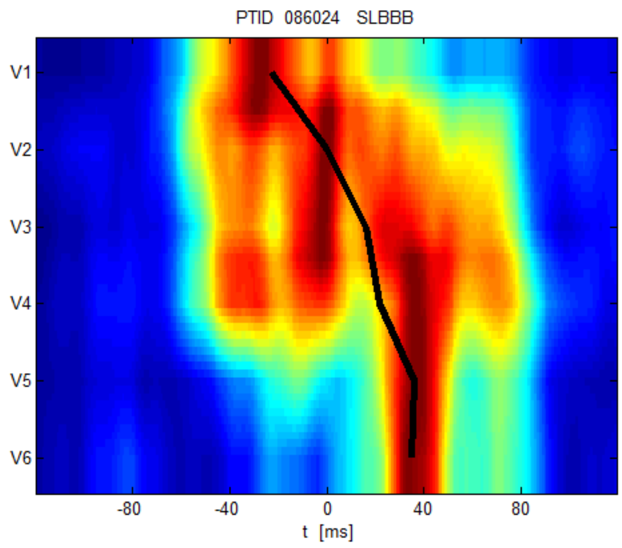
PTID 086024 SLBBB
V1
V2
V3
V4
V5
V6
-80
-40
0
40
80
t [ms]

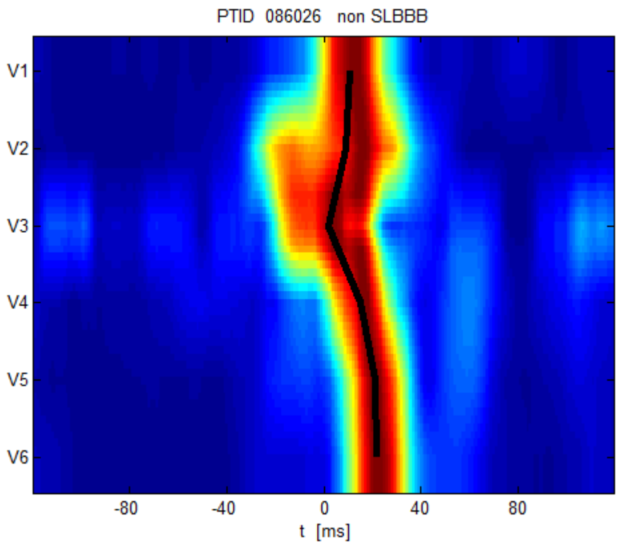
PTID 086026 non SLBBB
V1
V2
V3
V4
V5
V6
-80
-40
0
40
80
t [ms]

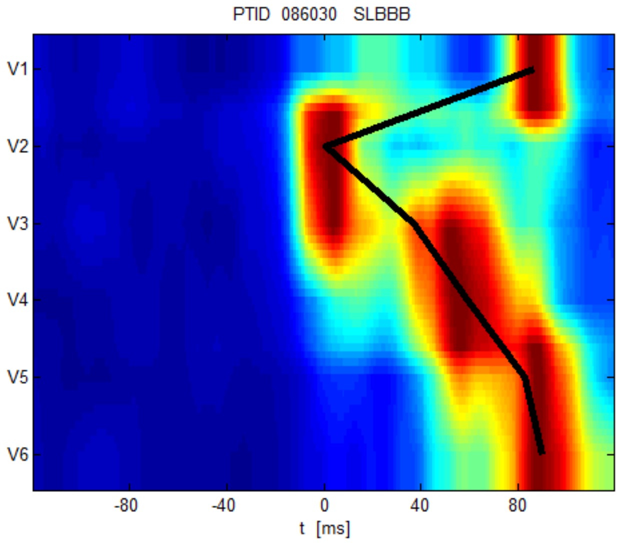
PTID 086030 SLBBB
V1
V2
V3
V4
V5
V6
-80
-40
0
40
80
t [ms]

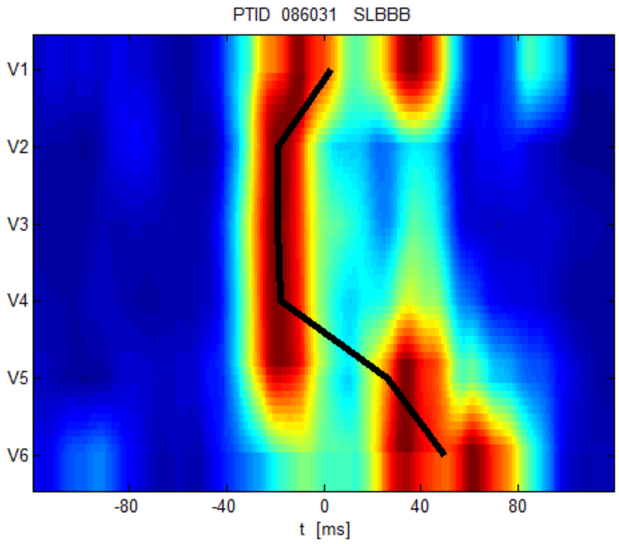
PTID 086031 SLBBB
V1
V2
V3
V4
V5
V6
-80
-40
0
40
80
t [ms]

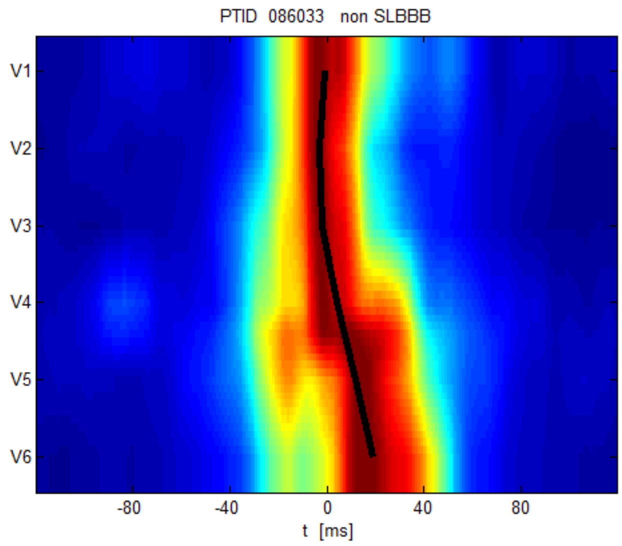
PTID 086033 non SLBBB
V1
V2
V3
V4
V5
V6
-80
-40
0
40
80
t [ms]

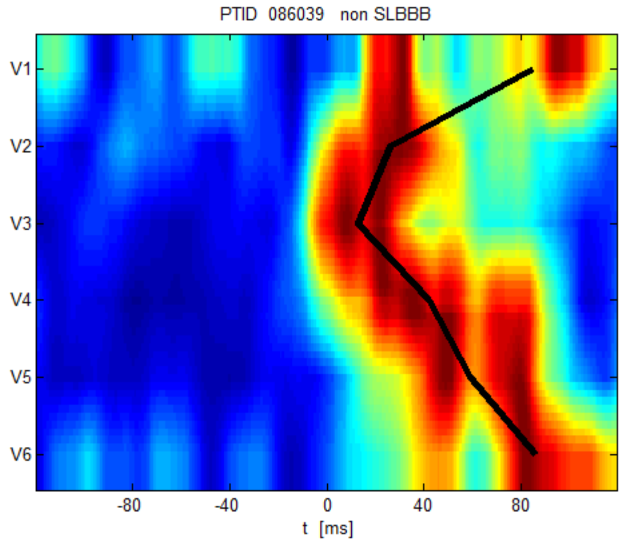
PTID 086039 non SLBBB
V1
V2
V3
V4
V5
V6
-80
-40
0
40
80
t [ms]

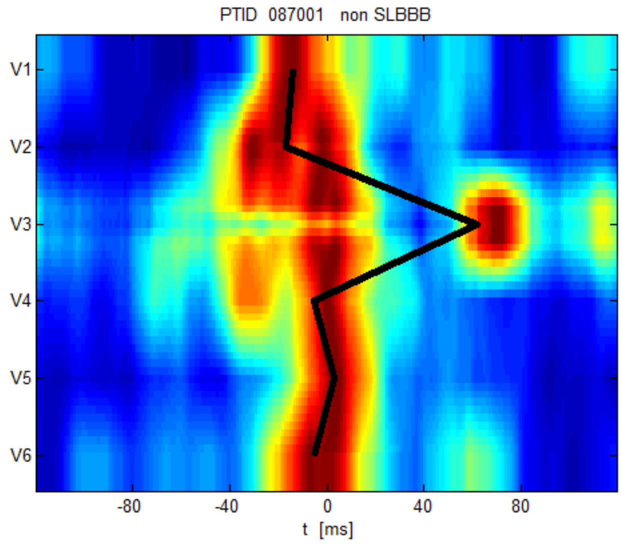
PTID 087001 non SLBBB
V1
V2
V3
V4
V5
V6
-80
-40
0
40
80
t [ms]

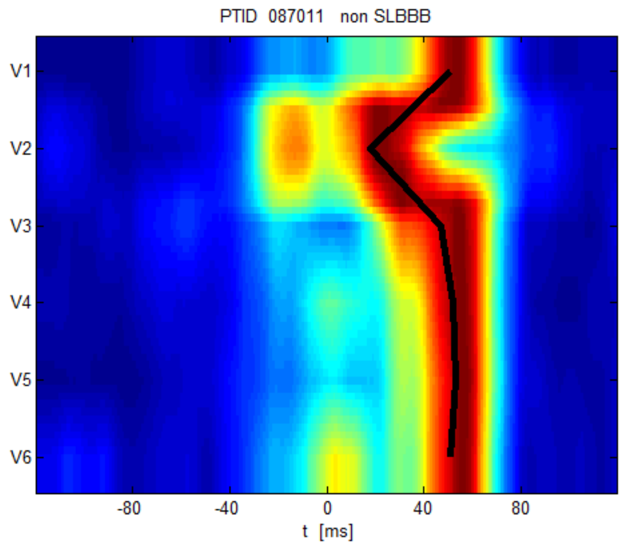

PTID 087011 non SLBBB
V1
V2
V3
V4
V5
V6
-80
-40
0
40
80
t [ms]

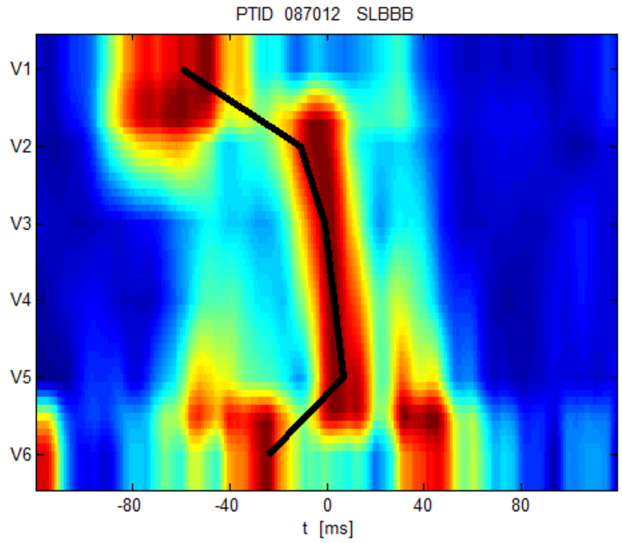

PTID 087012 SLBBB
V1
V2
V3
V4
V5
V6
-80
-40
0
40
80
t [ms]

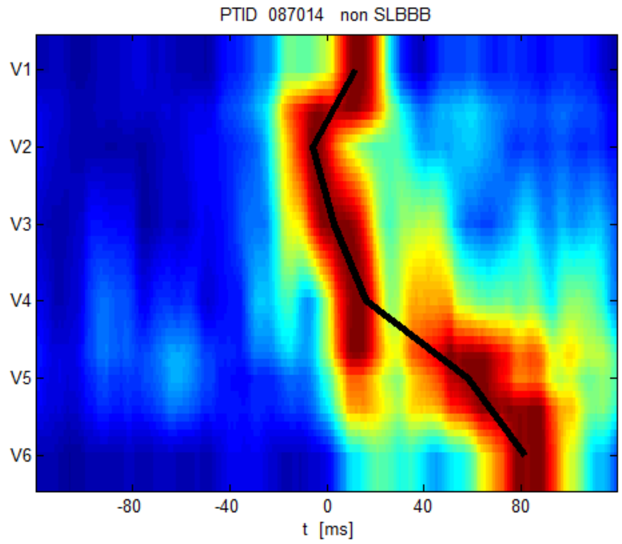

PTID 087014 non SLBBB
V1
V2
V3
V4
V5
V6
-80
-40
0
40
80
t [ms]

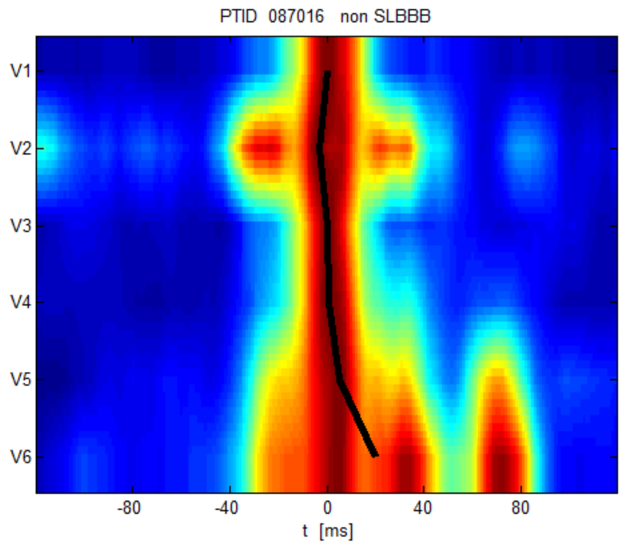
PTID 087016 non SLBBB
V1
V2
V3
V4
V5
V6
-80
-40
0
40
80
t [ms]

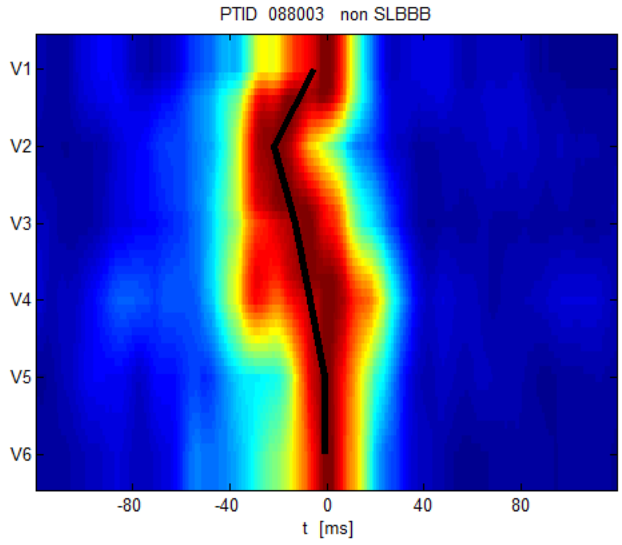
PTID 088003 non SLBBB
V1
V2
V3
V4
V5
V6
-80
-40
0
40
80
t [ms]

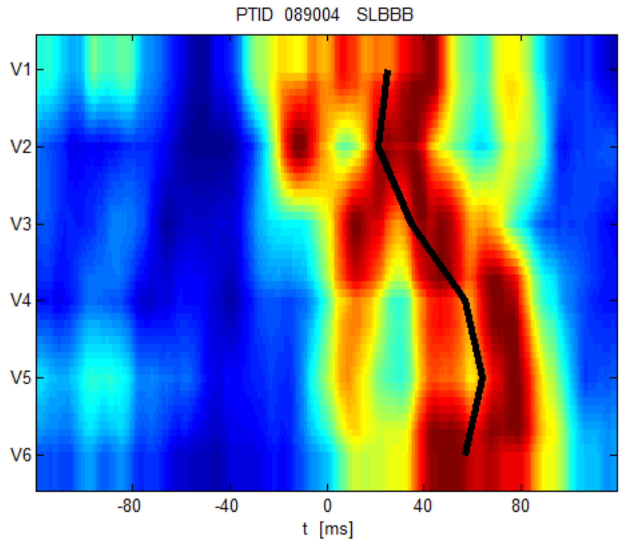
PTID 089004 SLBBB
V1
V2
V3
V4
V5
V6
-80
-40
0
40
80
t [ms]

PTID 089006 non SLBBB

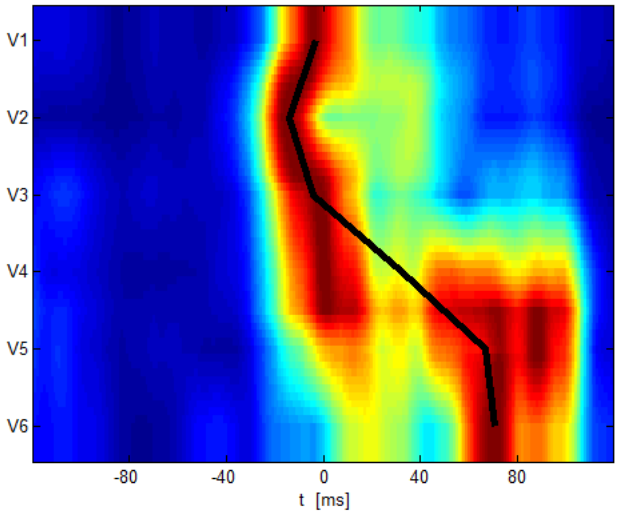

PTID 089008 non SLBBB

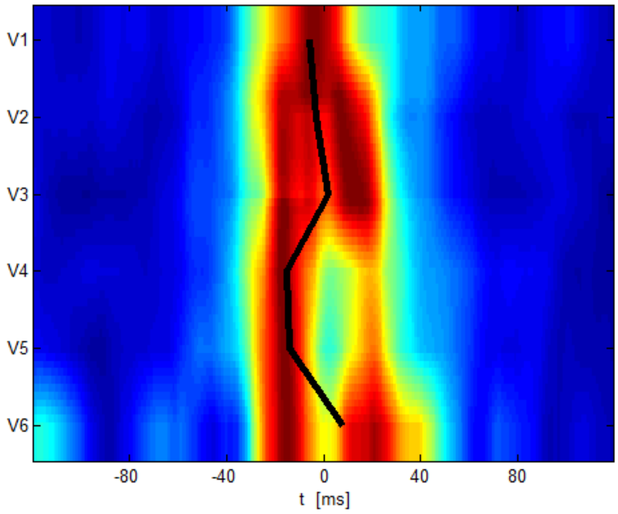

PTID 089013 SLBBB

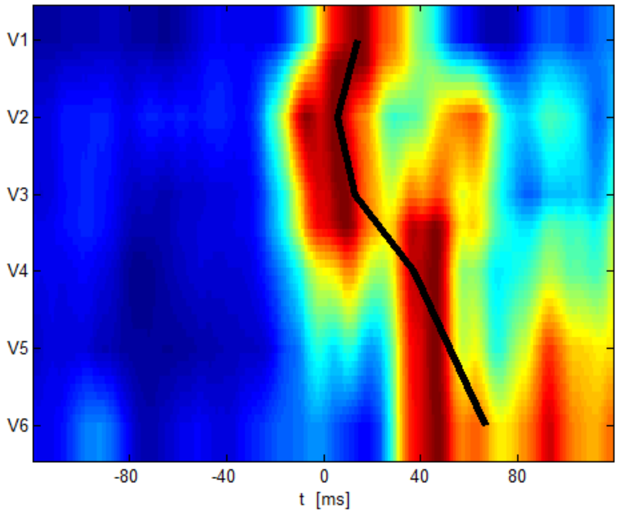

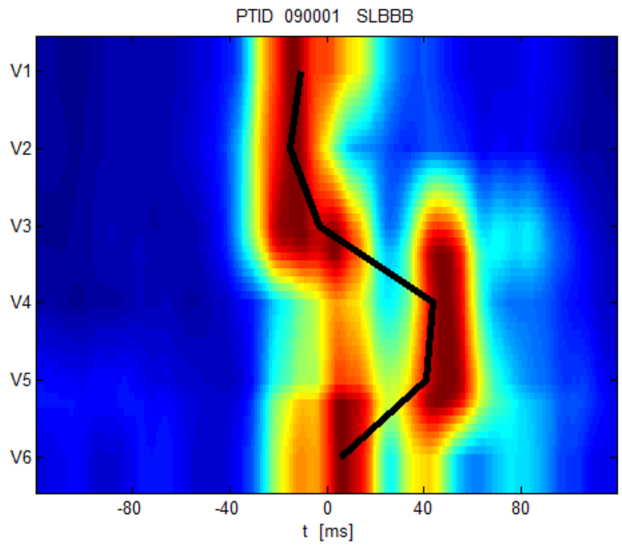
PTID 090001 SLBBB
V1
V2
V3
V4
V5
V6
-80
-40
0
40
80
t [ms]

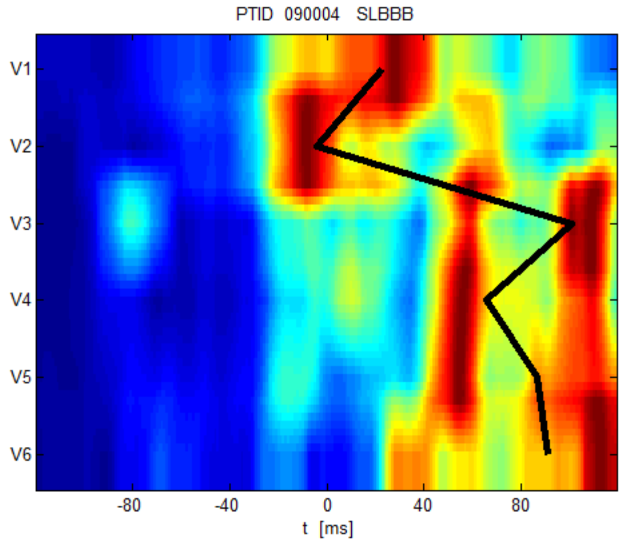
PTID 090004 SLBBB
V1
V2
V3
V4
V5
V6
-80
-40
0
40
80
t [ms]

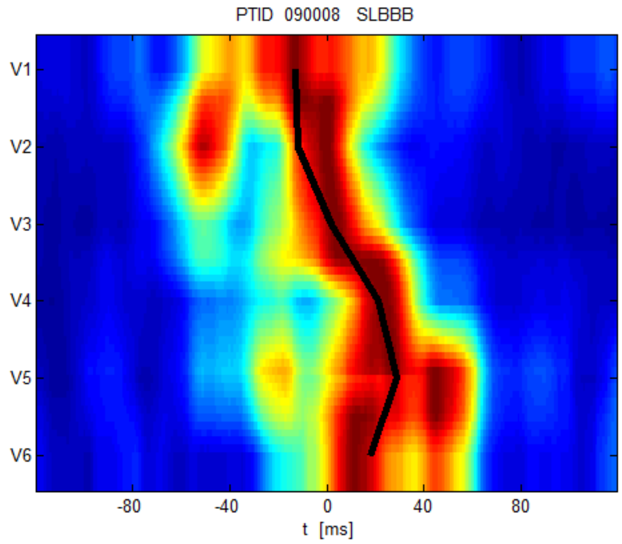
PTID 090008 SLBBB
V1
V2
V3
V4
V5
V6
-80
-40
0
40
80
t [ms]

PTID 091006 SLBBB

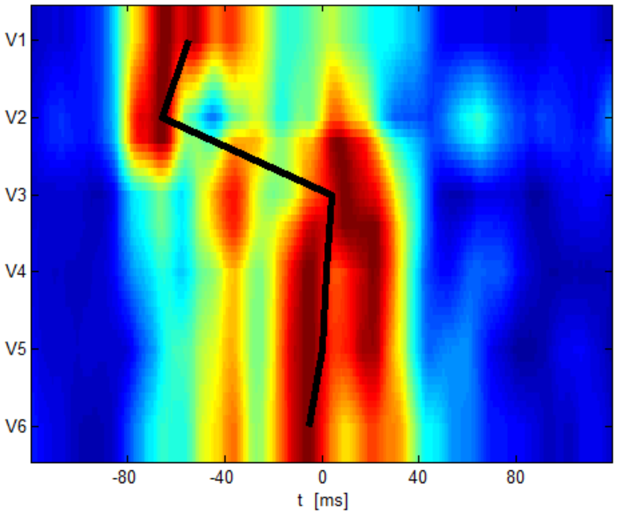

PTID 093001 SLBBB

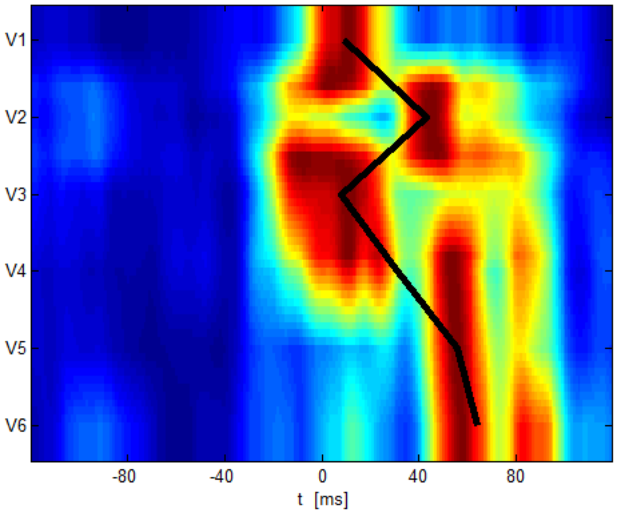

PTID 094003 SLBBB

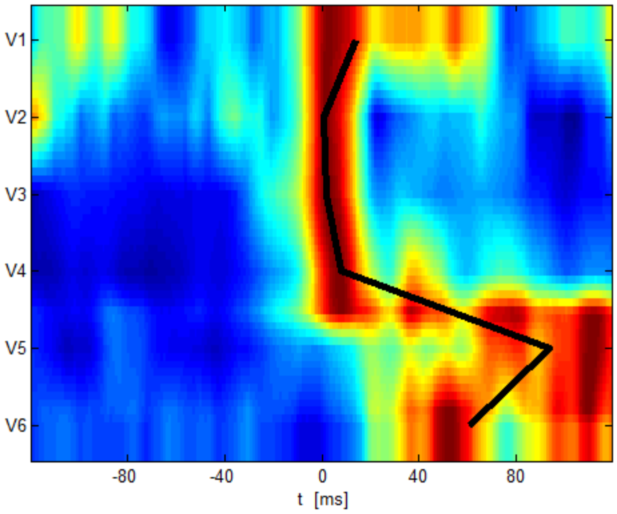

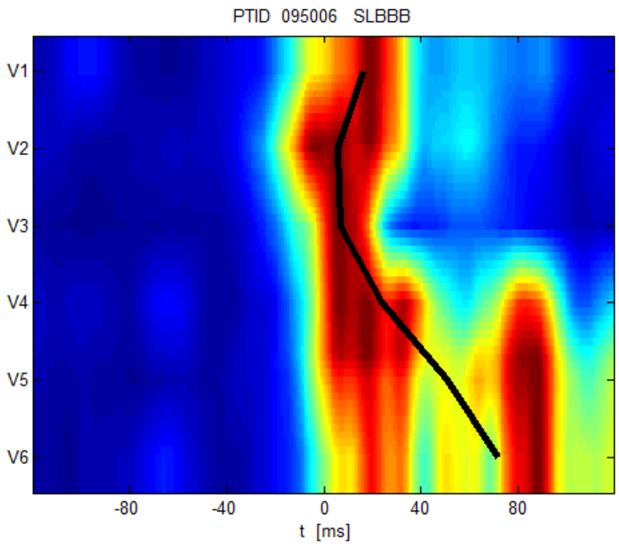
PTID 095006 SLBBB
V1
V2
V3
V4
V5
V6
-80
-40
0
40
80
t [ms]

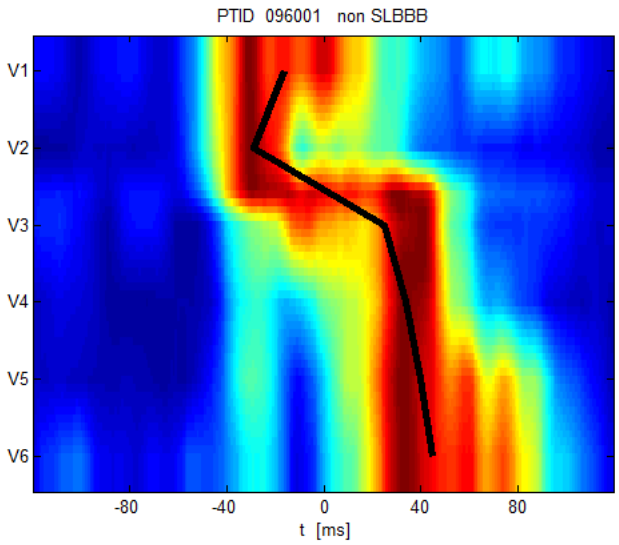
PTID 096001 non SLBBB
V1
V2
V3
V4
V5
V6
-80
-40
0
40
80
t [ms]

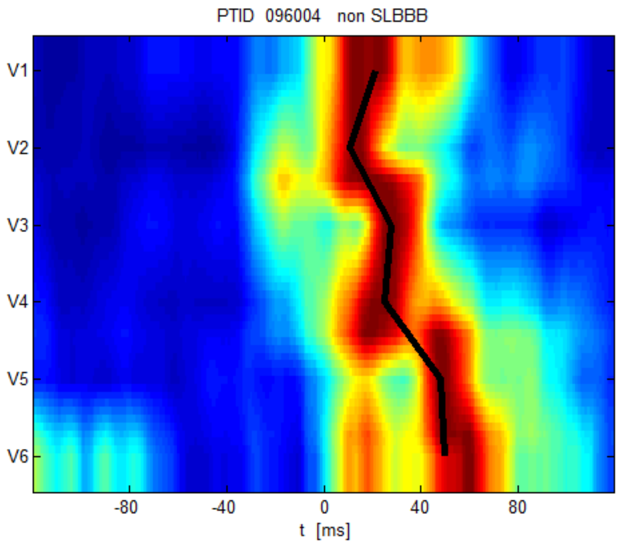
PTID 096004 non SLBBB
V1
V2
V3
V4
V5
V6
-80
-40
0
40
80
t [ms]

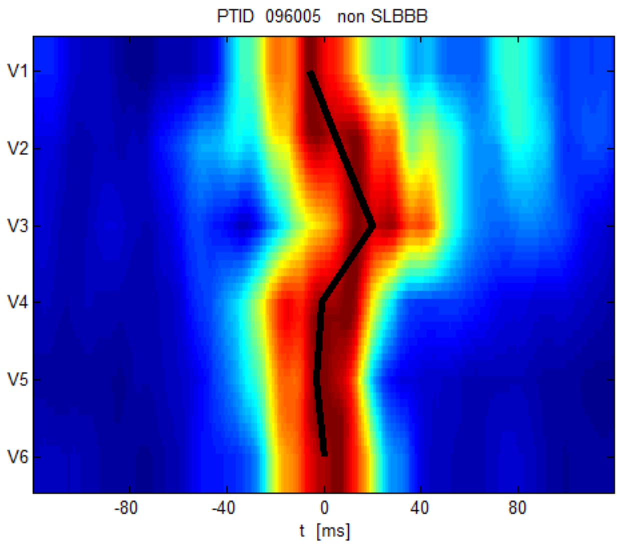
PTID 096005 non SLBBB
V1
V2
V3
V4
V5
V6
-80
-40
0
40
80
t [ms]

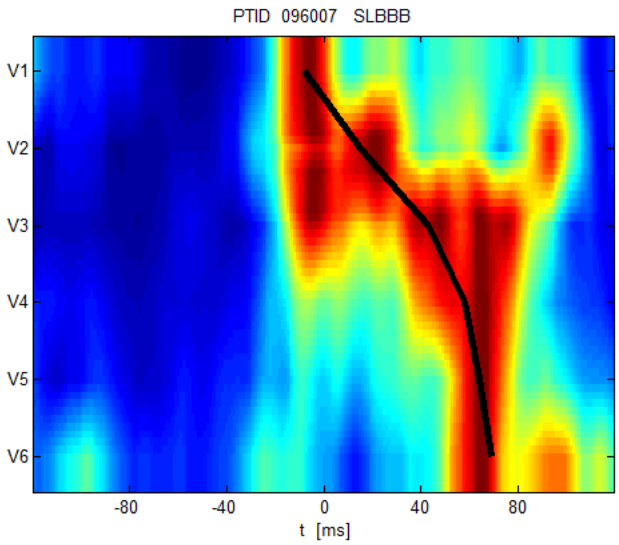
PTID 096007 SLBBB
V1
V2
V3
V4
V5
V6
-80
-40
0
40
80
t [ms]

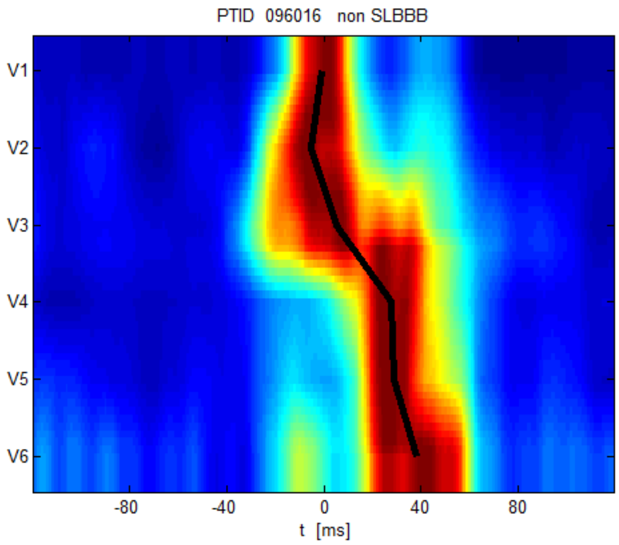
PTID 096016 non SLBBB
V1
V2
V3
V4
V5
V6
-80
-40
0
40
80
t [ms]

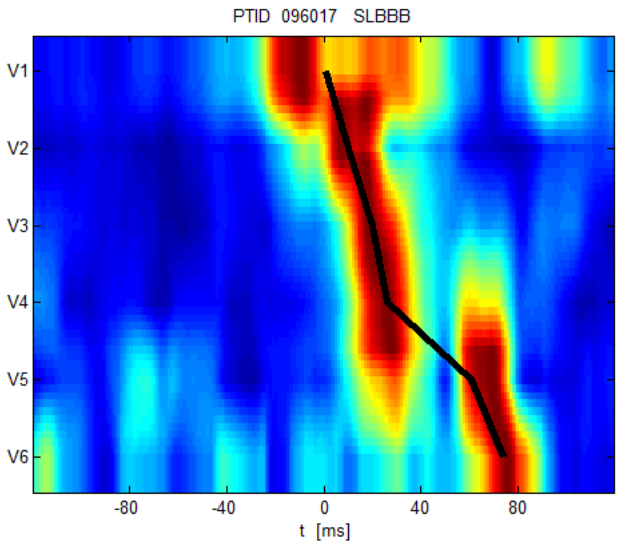
PTID 096017 SLBBB
V1
V2
V3
V4
V5
V6
-80
-40
0
40
80
t [ms]

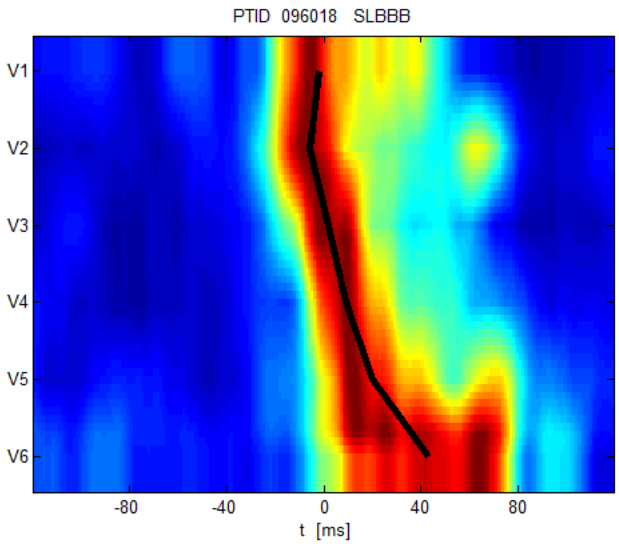
PTID 096018 SLBBB
V1
V2
V3
V4
V5
V6
-80
-40
0
40
80
t [ms]

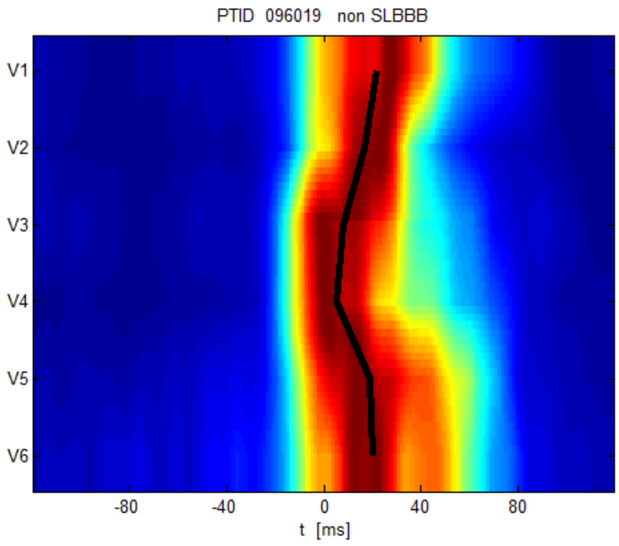
PTID 096019 non SLBBB
V1
V2
V3
V4
V5
V6
-80
-40
0
40
80
t [ms]

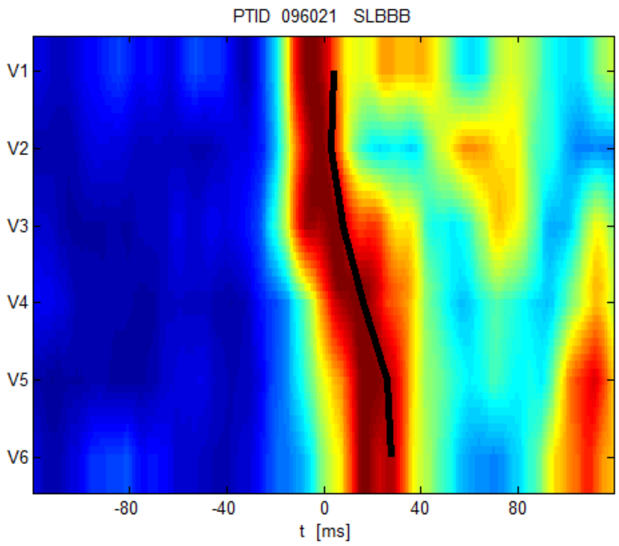
PTID 096021 SLBBB
V1
V2
V3
V4
V5
V6
-80
-40
0
40
80
t [ms]

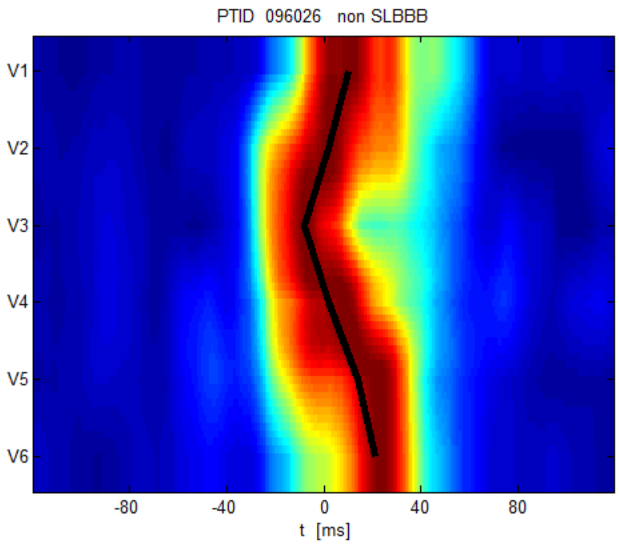
PTID 096026 non SLBBB
V1
V2
V3
V4
V5
V6
-80
-40
0
40
80
t [ms]

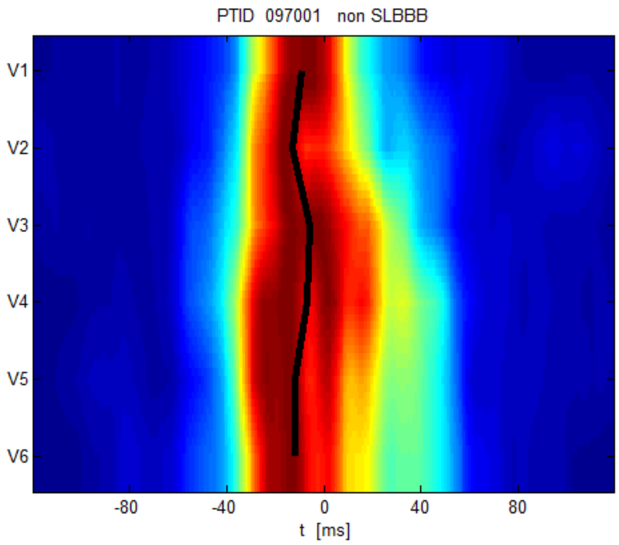
PTID 097001 non SLBBB
V1
V2
V3
V4
V5
V6
-80
-40
0
40
80
t [ms]

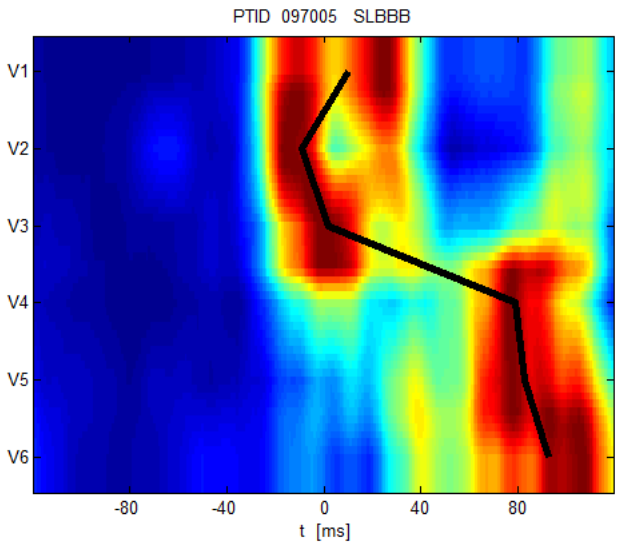
PTID 097005 SLBBB
V1
V2
V3
V4
V5
V6
-80
-40
0
40
80
t [ms]

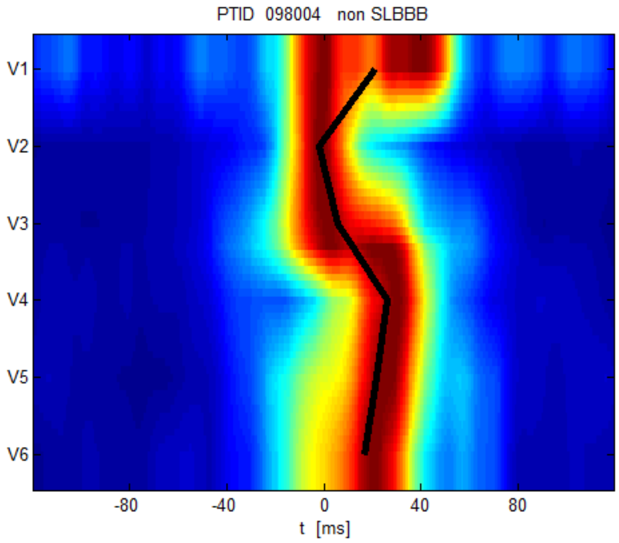
PTID 098004 non SLBBB
V1
V2
V3
V4
V5
V6
-80
-40
0
40
80
t [ms]

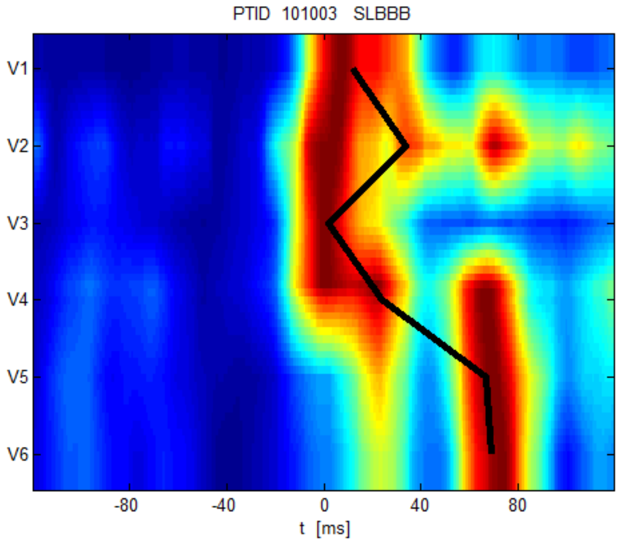
PTID 101003 SLBBB
V1
V2
V3
V4
V5
V6
-80
-40
0
40
80
t [ms]

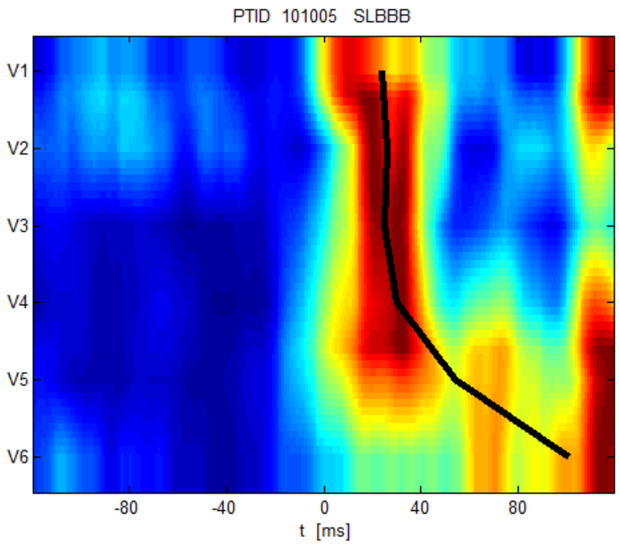
PTID 101005 SLBBB
V1
V2
V3
V4
V5
V6
-80
-40
0
40
80
t [ms]

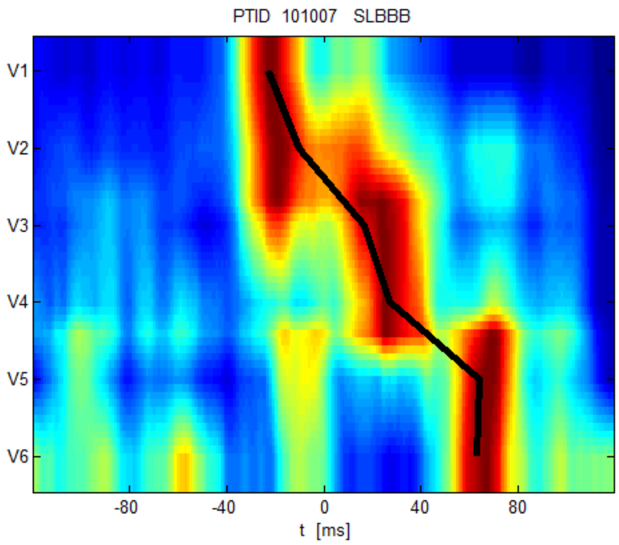
PTID 101007 SLBBB
V1
V2
V3
V4
V5
V6
-80
-40
0
40
80
t [ms]

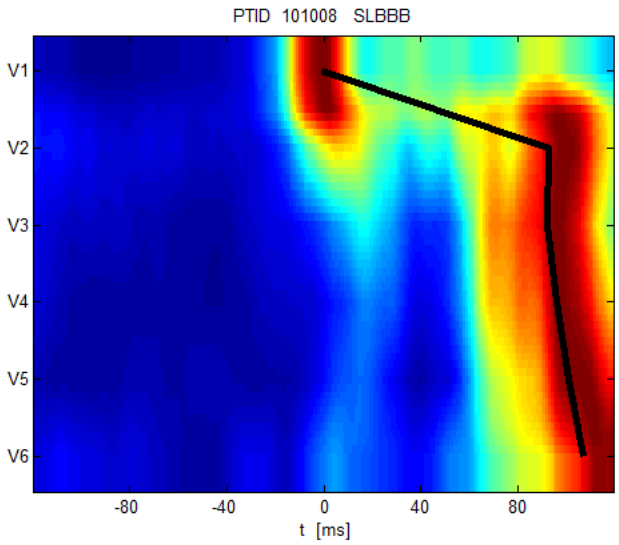
PTID 101008 SLBBB
V1
V2
V3
V4
V5
V6
-80
-40
0
40
80
t [ms]

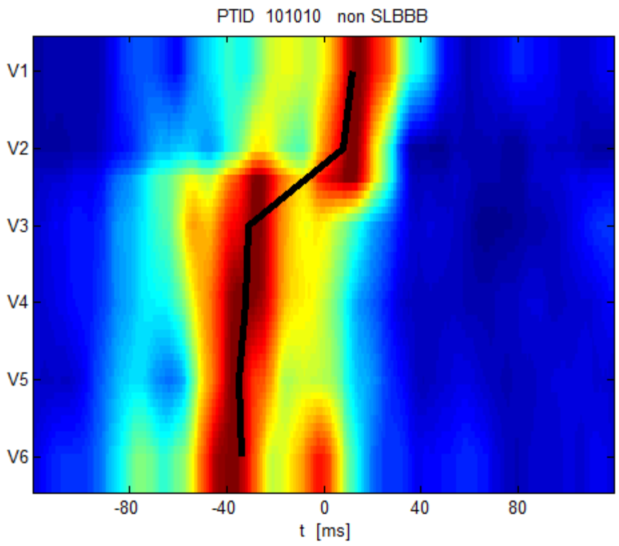
PTID 101010 non SLBBB
V1
V2
V3
V4
V5
V6
-80
-40
0
40
80
t [ms]

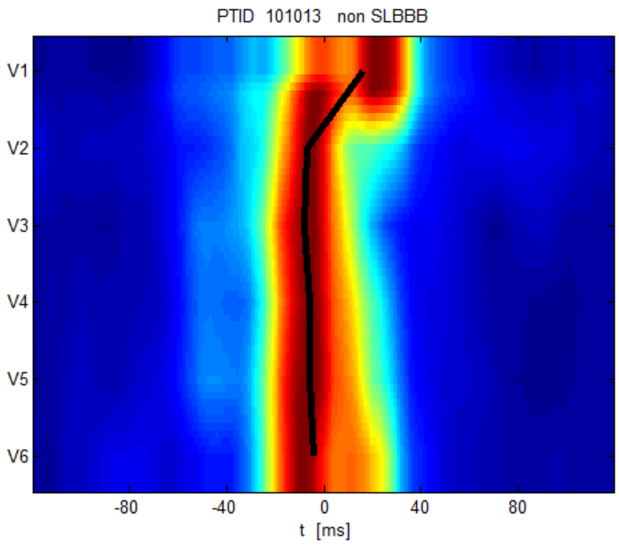
PTID 101013 non SLBBB
V1
V2
V3
V4
V5
V6
-80
-40
0
40
80
t [ms]

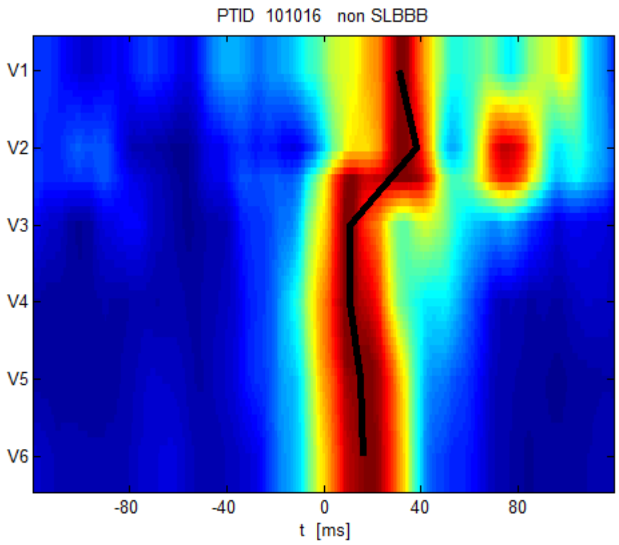
PTID 101016 non SLBBB
V1
V2
V3
V4
V5
V6
-80
-40
0
40
80
t [ms]

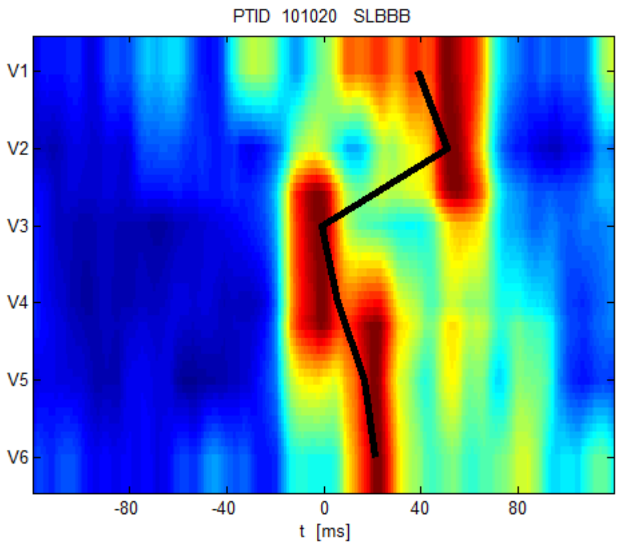
PTID 101020 SLBBB
V1
V2
V3
V4
V5
V6
-80
-40
0
40
80
t [ms]

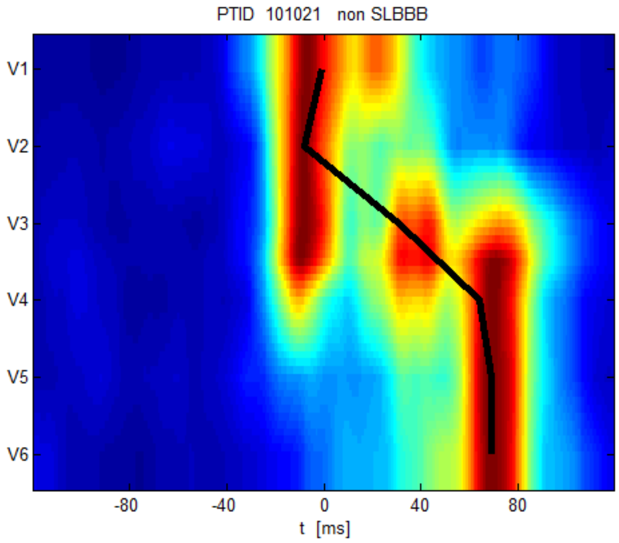
PTID 101021 non SLBBB
V1
V2
V3
V4
V5
V6
-80
-40
0
40
80
t [ms]

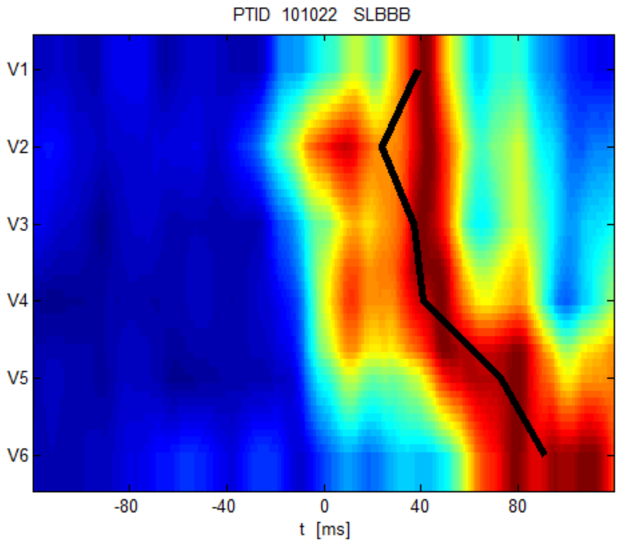
PTID 101022 SLBBB
V1
V2
V3
V4
V5
V6
-80
-40
0
40
80
t [ms]

PTID 101028 SLBBB

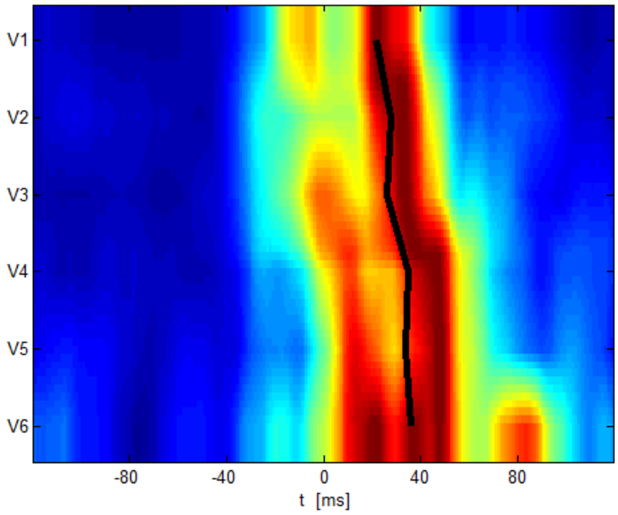

PTID 102001 SLBBB

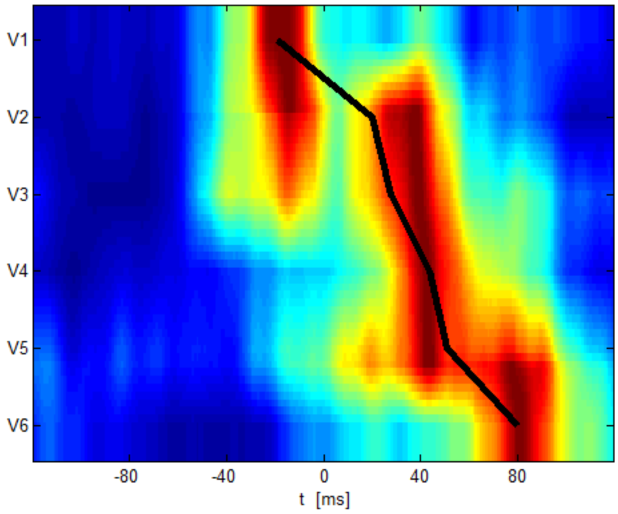

PTID 102007 non SLBBB

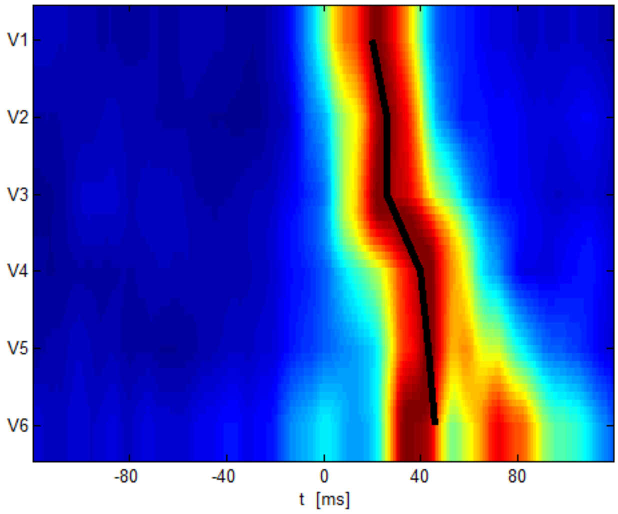

PTID 102011 SLBBB

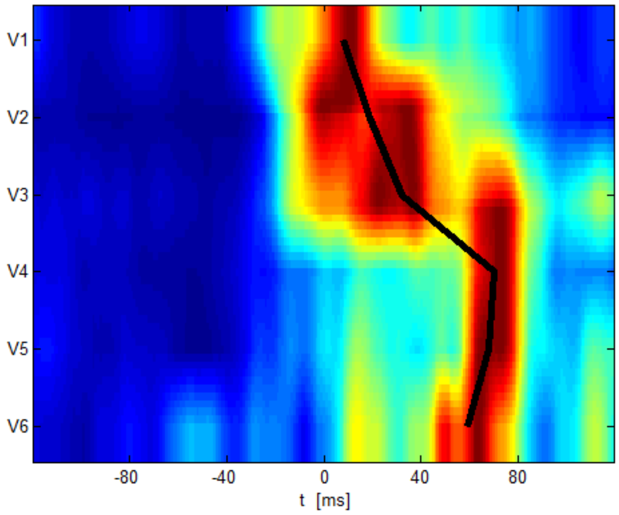

PTID 102013 SLBBB

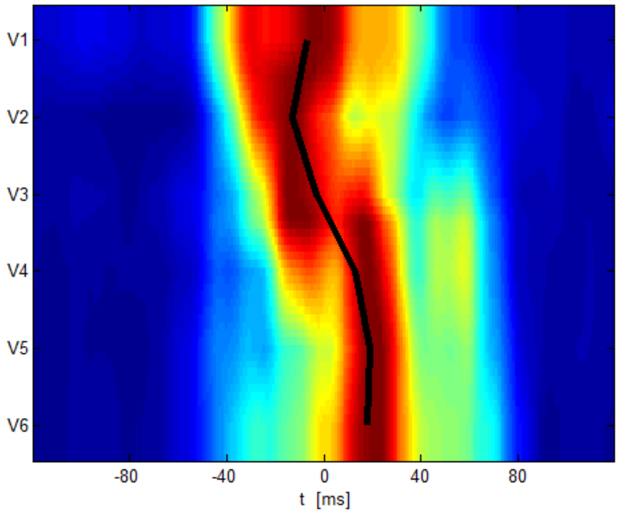

PTID 102014 SLBBB

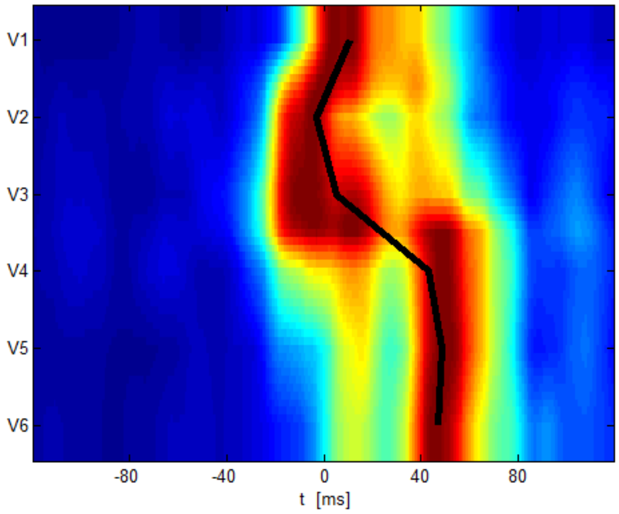

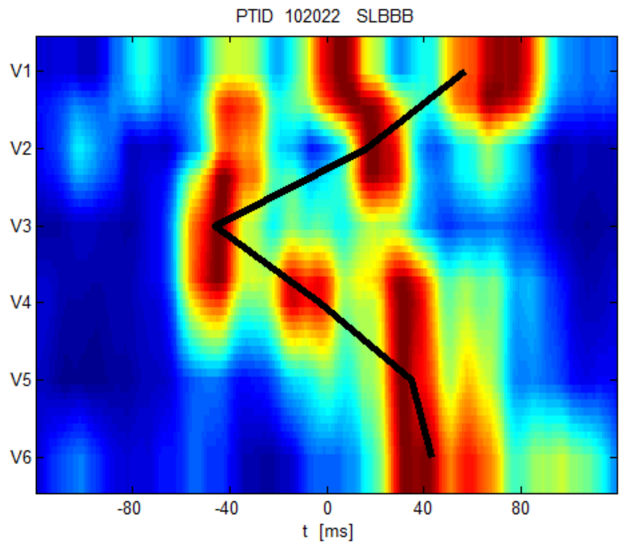
PTID 102022 SLBBB
V1
V2
V3
V4
V5
V6
-80
-40
0
40
80
t [ms]

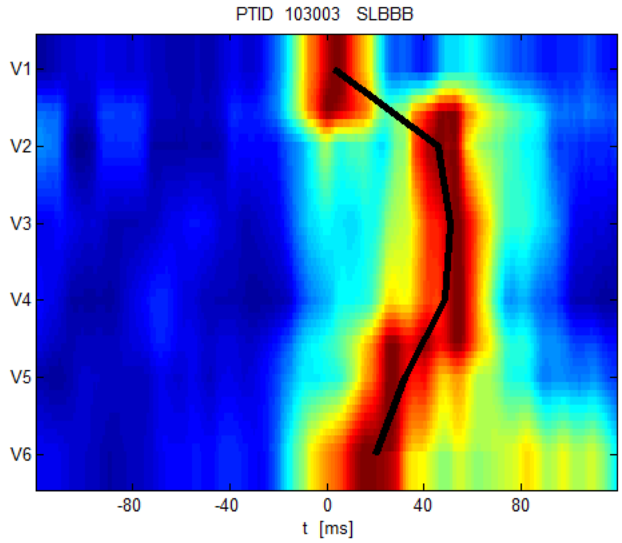
PTID 103003 SLBBB
V1
V2
V3
V4
V5
V6
-80
-40
0
40
80
t [ms]

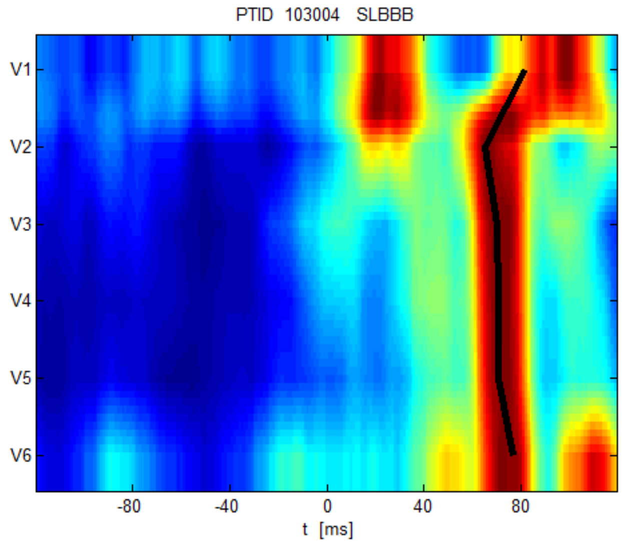
PTID 103004 SLBBB
V1
V2
V3
V4
V5
V6
-80
-40
0
40
80
t [ms]

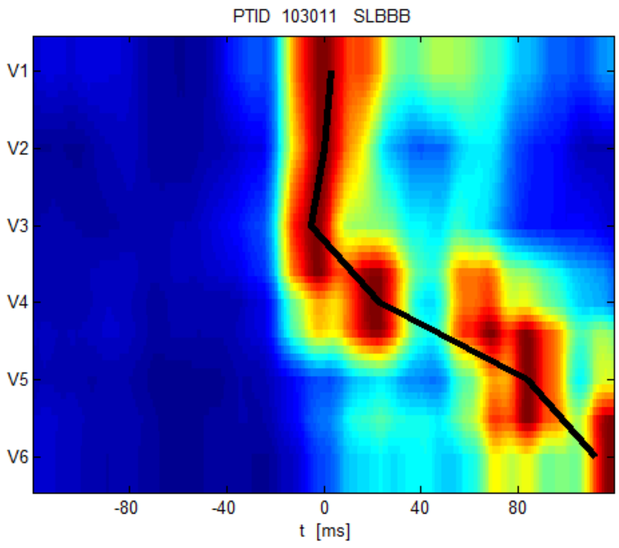
PTID 103011 SLBBB
V1
V2
V3
V4
V5
V6
-80
-40
0
40
80
t [ms]

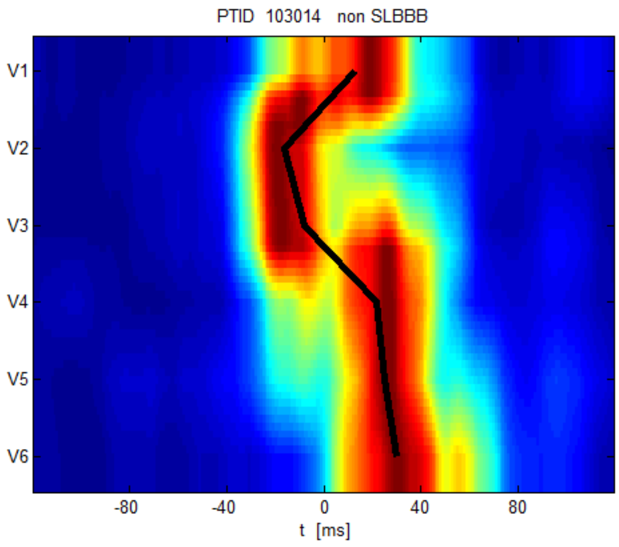
PTID 103014 non SLBBB
V1
V2
V3
V4
V5
V6
-80
-40
0
40
80
t [ms]

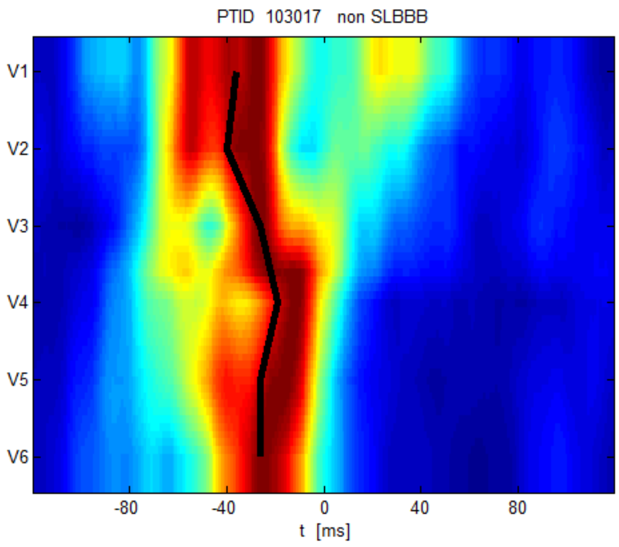
PTID 103017 non SLBBB
V1
V2
V3
V4
V5
V6
-80
-40
0
40
80
t [ms]

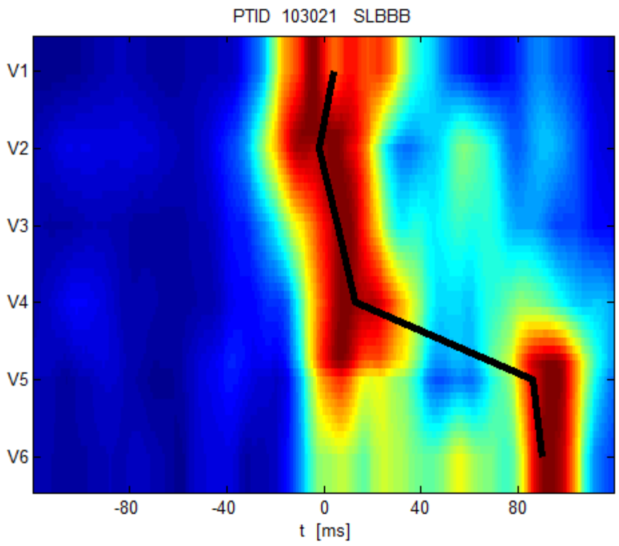
PTID 103021 SLBBB
V1
V2
V3
V4
V5
V6
-80
-40
0
40
80
t [ms]

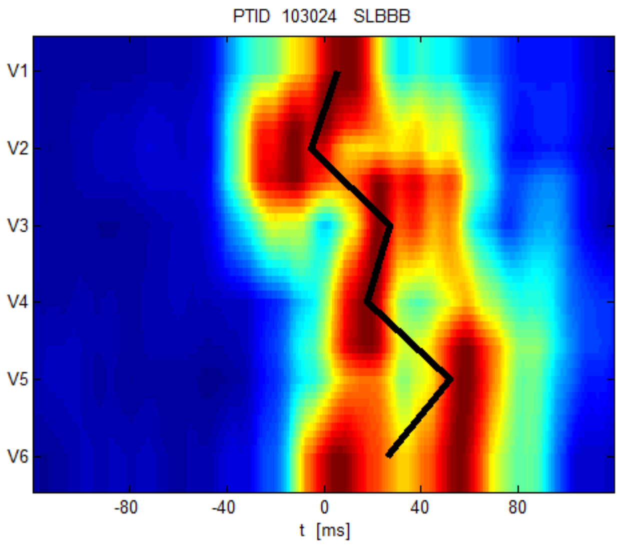
PTID 103024 SLBBB
V1
V2
V3
V4
V5
V6
-80
-40
0
40
80
t [ms]

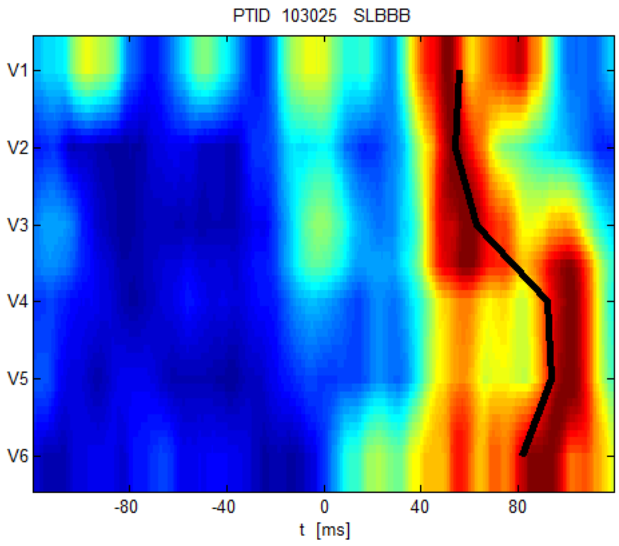
PTID 103025 SLBBB
V1
V2
V3
V4
V5
V6
-80
-40
0
40
80
t [ms]

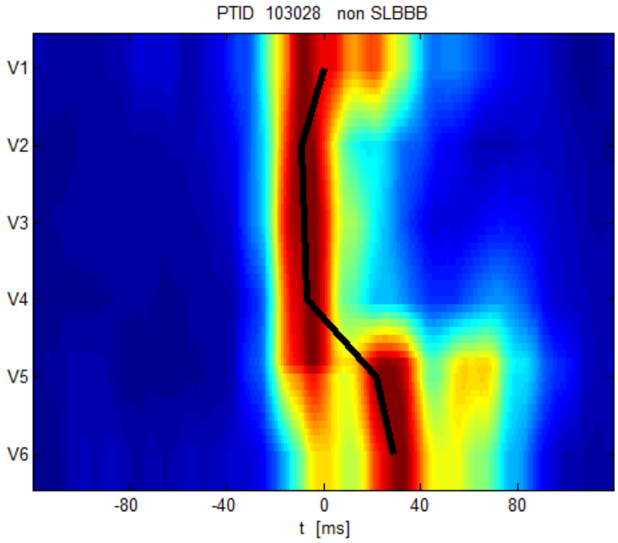
PTID 103028 non SLBBB
V1
V2
V3
V4
V5
V6
-80
-40
0
40
80
t [ms]

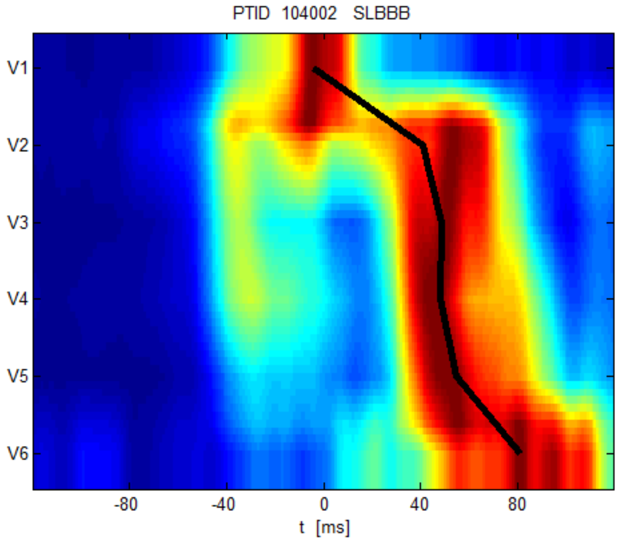
PTID 104002 SLBBB
V1
V2
V3
V4
V5
V6
-80
-40
0
40
80
t [ms]

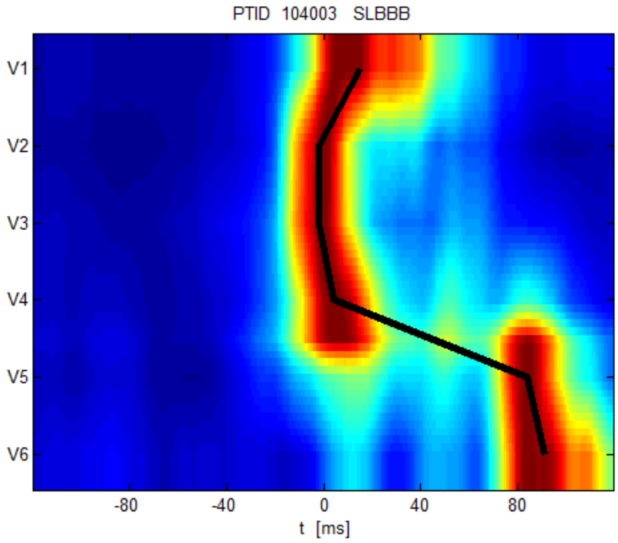
PTID 104003 SLBBB
V1
V2
V3
V4
V5
V6
-80
-40
0
40
80
t [ms]

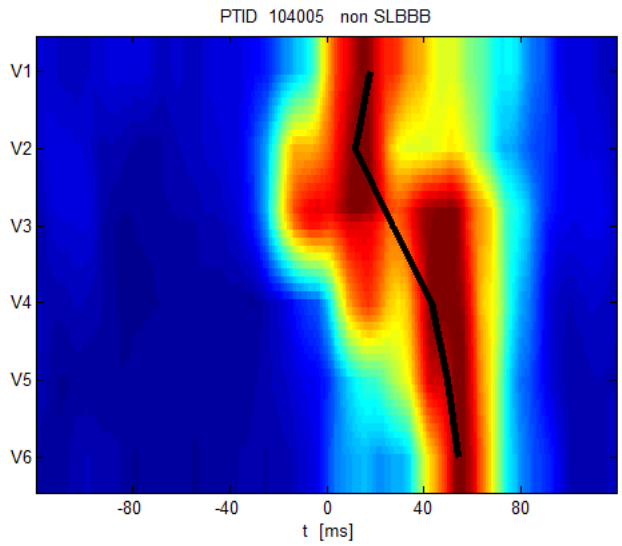
PTID 104005 non SLBBB
V1
V2
V3
V4
V5
V6
-80
-40
0
40
80
t [ms]

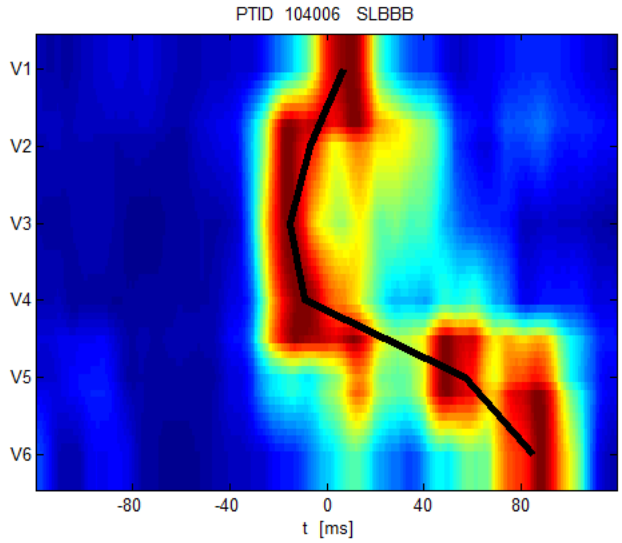
PTID 104006 SLBBB
V1
V2
V3
V4
V5
V6
-80
-40
0
40
80
t [ms]

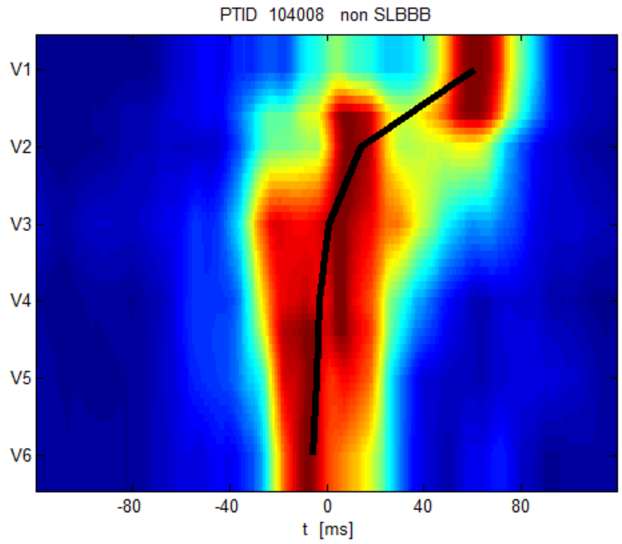
PTID 104008 non SLBBB
V1
V2
V3
V4
V5
V6
-80
-40
0
40
80
t [ms]

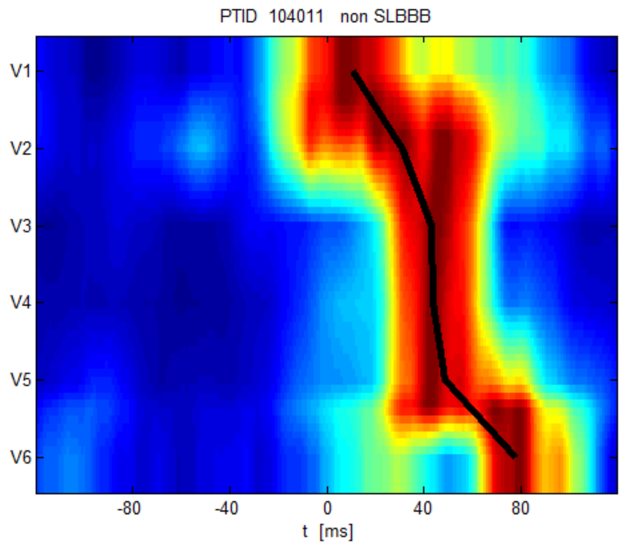
PTID 104011 non SLBBB
V1
V2
V3
V4
V5
V6
-80
-40
0
40
80
t [ms]

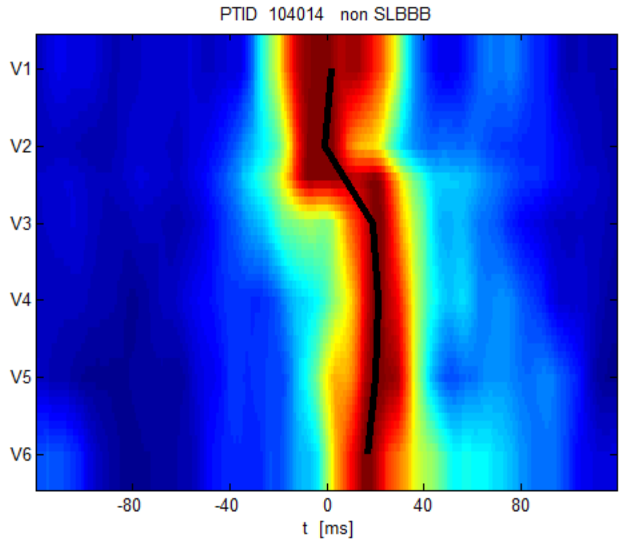
PTID 104014 non SLBBB
V1
V2
V3
V4
V5
V6
-80
-40
0
40
80
t [ms]

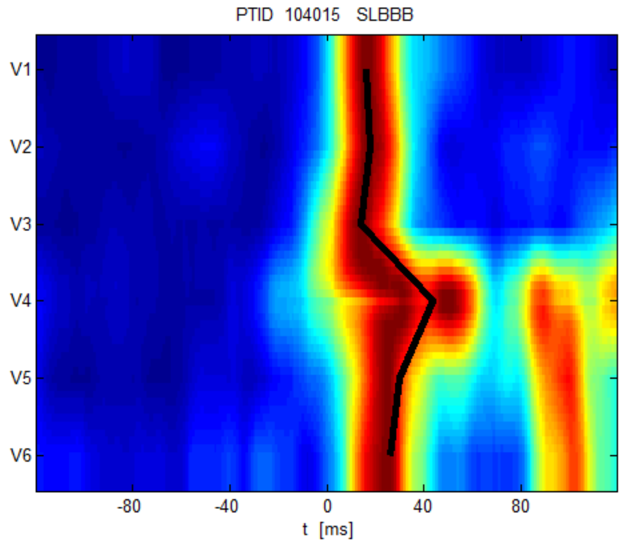
PTID 104015 SLBBB
V1
V2
V3
V4
V5
V6
-80
-40
0
40
80
t [ms]

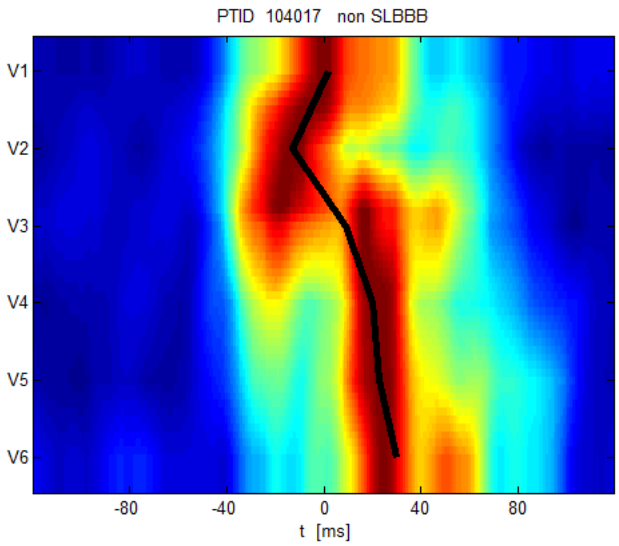
PTID 104017 non SLBBB
V1
V2
V3
V4
V5
V6
-80
-40
0
40
80
t [ms]

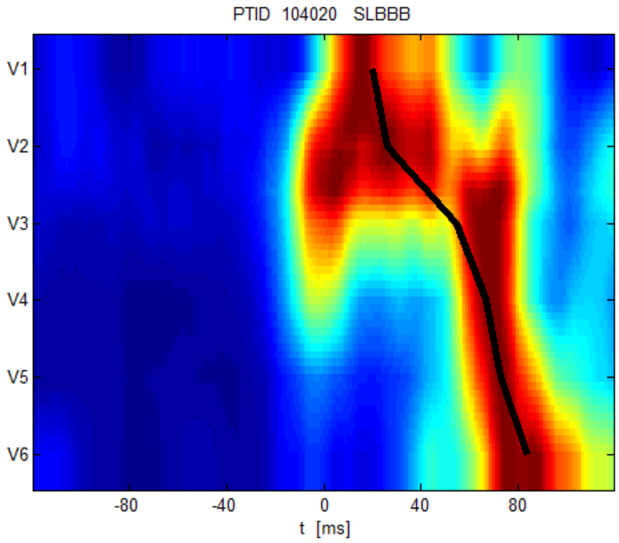
PTID 104020 SLBBB
V1
V2
V3
V4
V5
V6
-80
-40
0
40
80
t [ms]

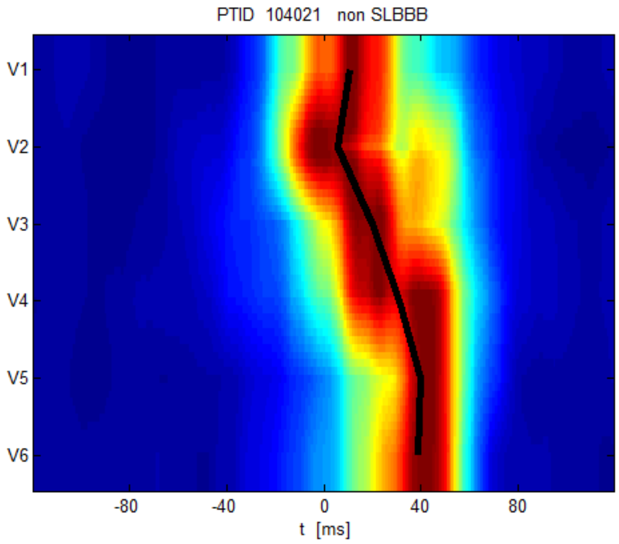
PTID 104021 non SLBBB
V1
V2
V3
V4
V5
V6
-80
-40
0
40
80
t [ms]

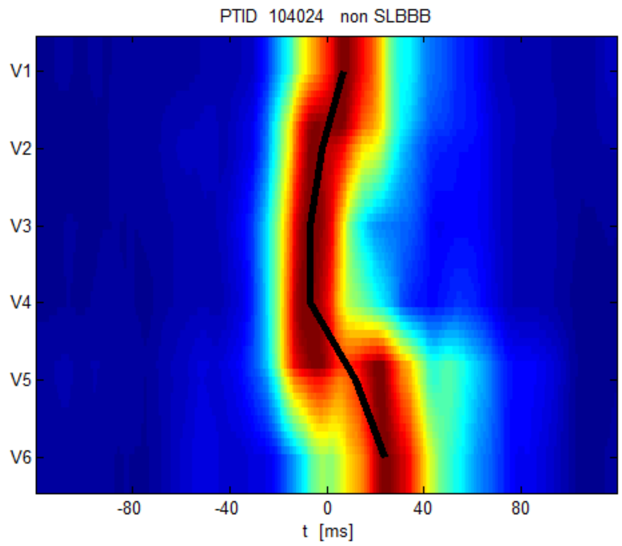
PTID 104024 non SLBBB
V1
V2
V3
V4
V5
V6
-80
-40
0
40
80
t [ms]

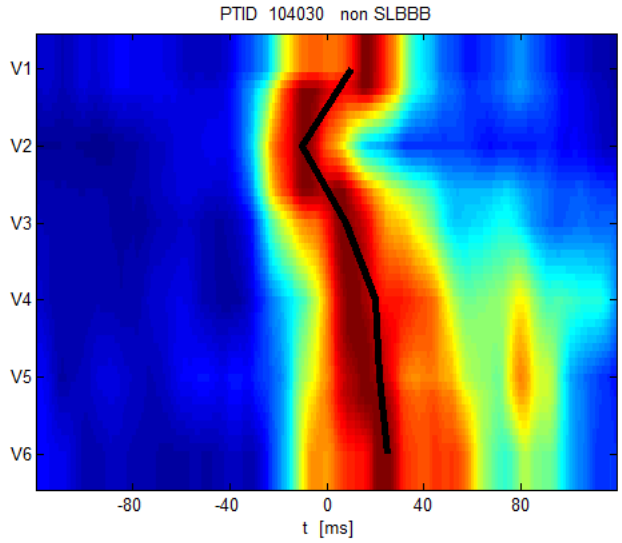
PTID 104030 non SLBBB
V1
V2
V3
V4
V5
V6
-80
-40
0
40
80
t [ms]

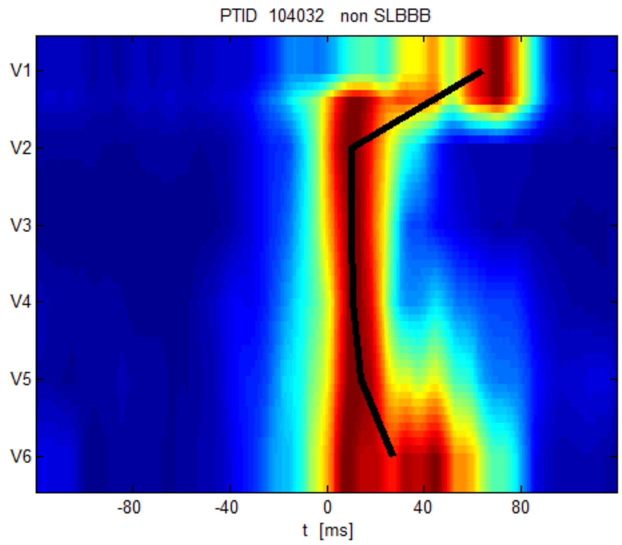
PTID 104032 non SLBBB
V1
V2
V3
V4
V5
V6
-80
-40
0
40
80
t [ms]

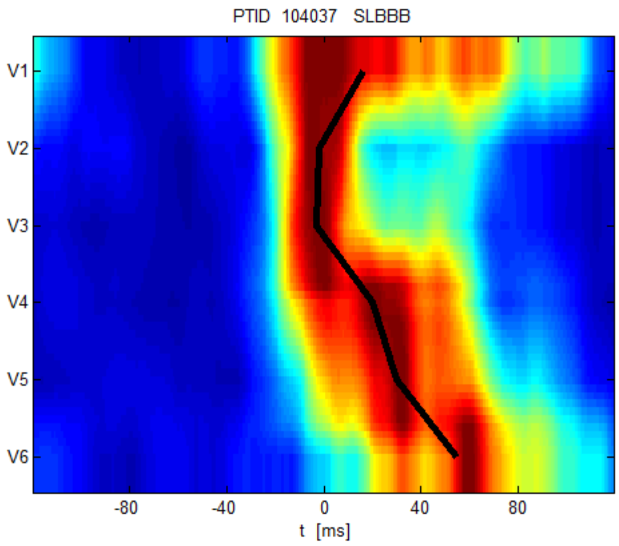
PTID 104037 SLBBB
V1
V2
V3
V4
V5
V6
-80
-40
0
40
80
t [ms]

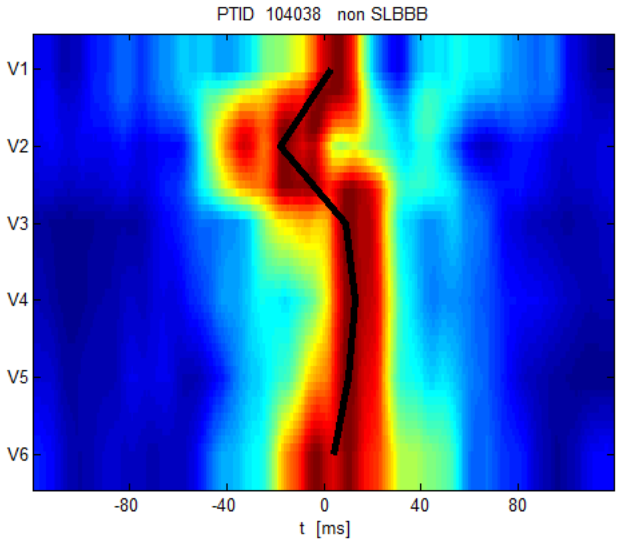
PTID 104038 non SLBBB
V1
V2
V3
V4
V5
V6
-80
-40
0
40
80
t [ms]

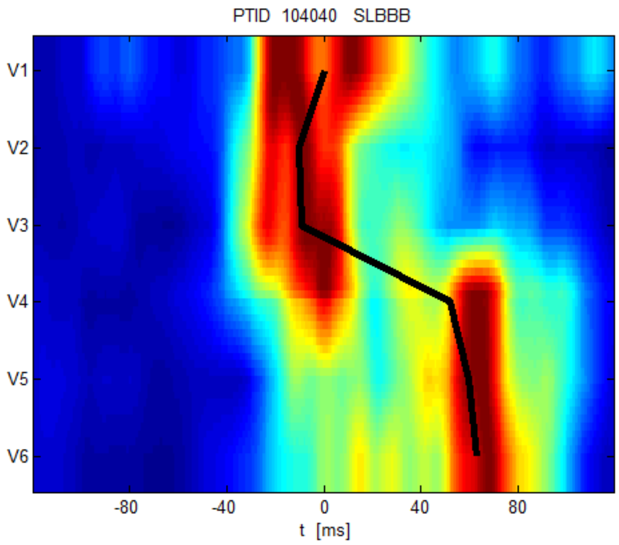
PTID 104040 SLBBB
V1
V2
V3
V4
V5
V6
-80
-40
0
40
80
t [ms]

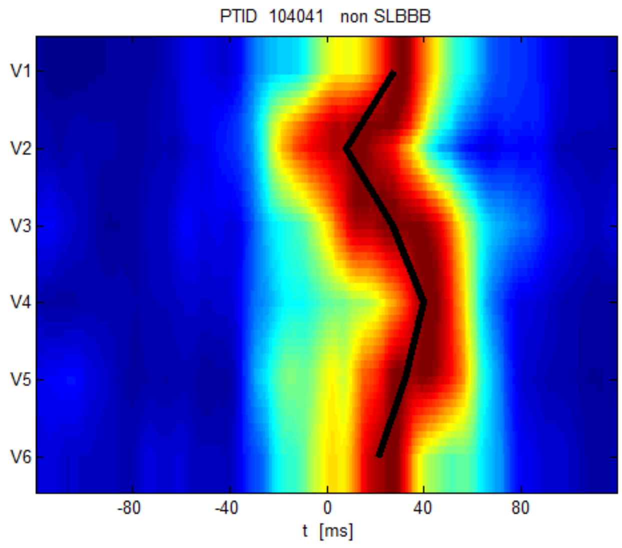
PTID 104041 non SLBBB
V1
V2
V3
V4
V5
V6
-80
-40
0
40
80
t [ms]

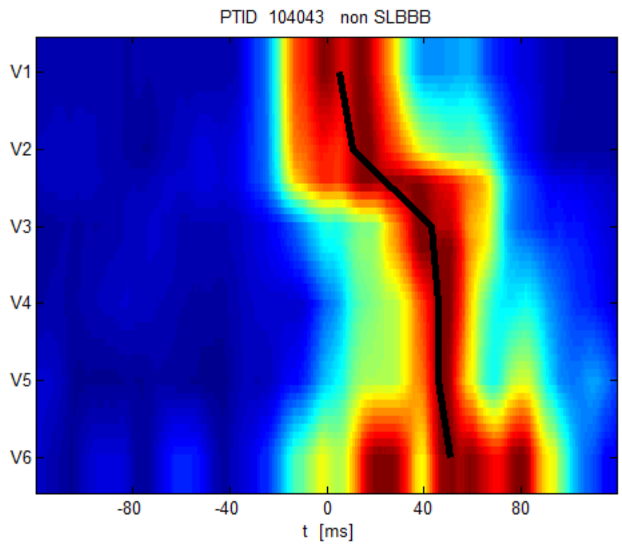
PTID 104043 non SLBBB
V1
V2
V3
V4
V5
V6
-80
-40
0
40
80
t [ms]

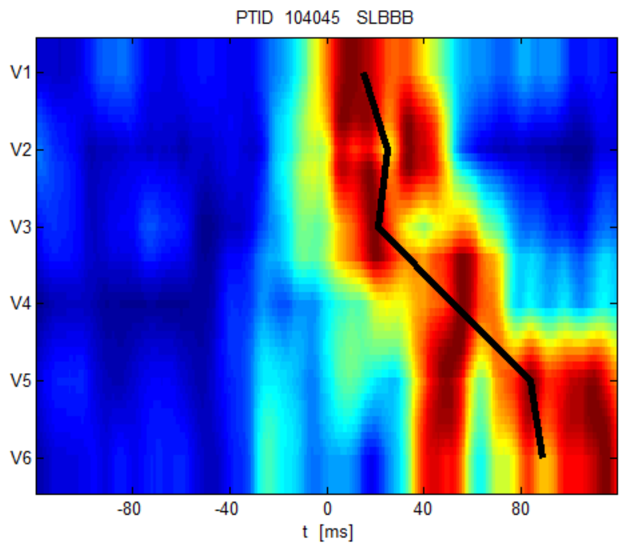
PTID 104045 SLBBB
V1
V2
V3
V4
V5
V6
-80
-40
0
40
80
t [ms]

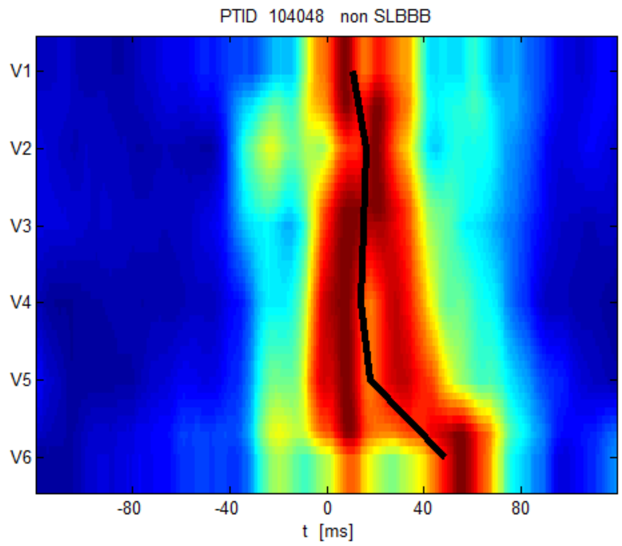

PTID 104048 non SLBBB
V1
V2
V3
V4
V5
V6
-80
-40
0
40
80
t [ms]

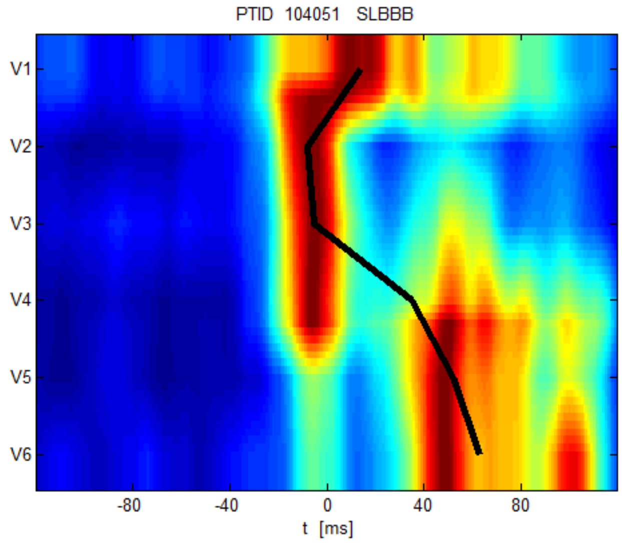

PTID 104051 SLBBB
V1
V2
V3
V4
V5
V6
-80
-40
0
40
80
t [ms]

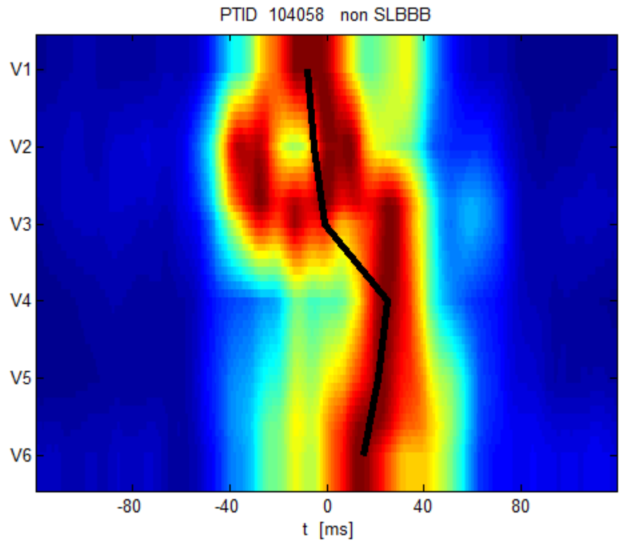

PTID 104058 non SLBBB
V1
V2
V3
V4
V5
V6
-80
-40
0
40
80
t [ms]

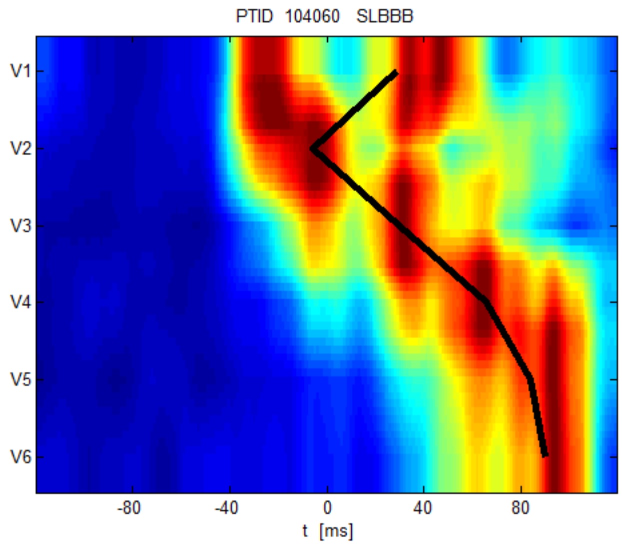
PTID 104060 SLBBB
V1
V2
V3
V4
V5
V6
-80
-40
0
40
80
t [ms]

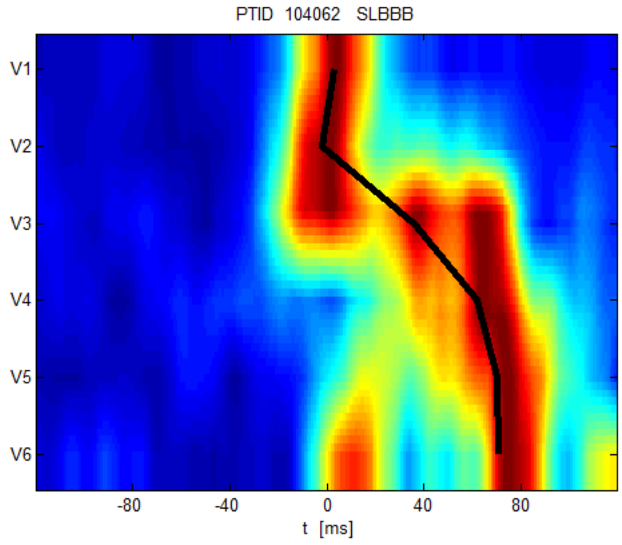
PTID 104062 SLBBB
V1
V2
V3
V4
V5
V6
-80
-40
0
40
80
t [ms]

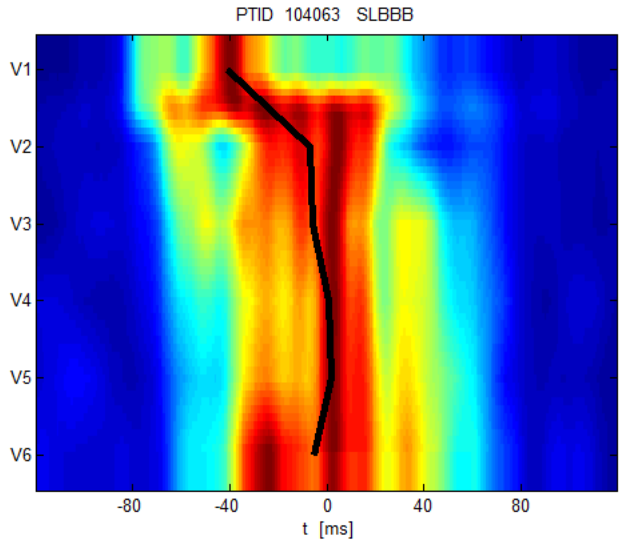
PTID 104063 SLBBB
V1
V2
V3
V4
V5
V6
-80
-40
0
40
80
t [ms]

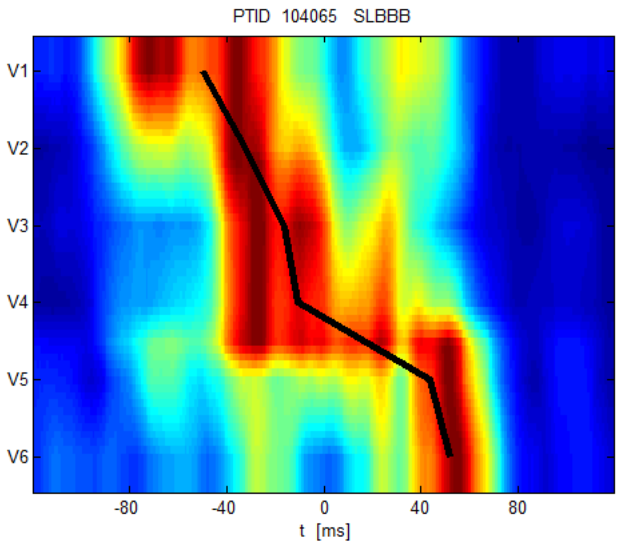
PTID 104065 SLBBB
V1
V2
V3
V4
V5
V6
-80
-40
0
40
80
t [ms]

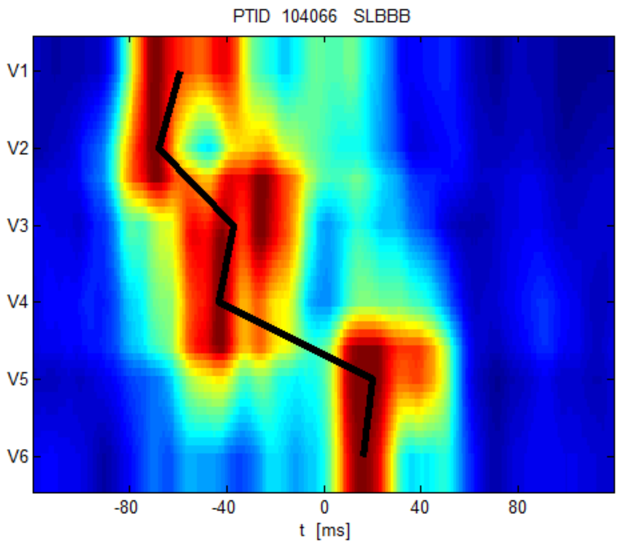
PTID 104066 SLBBB
V1
V2
V3
V4
V5
V6
-80
-40
0
40
80
t [ms]

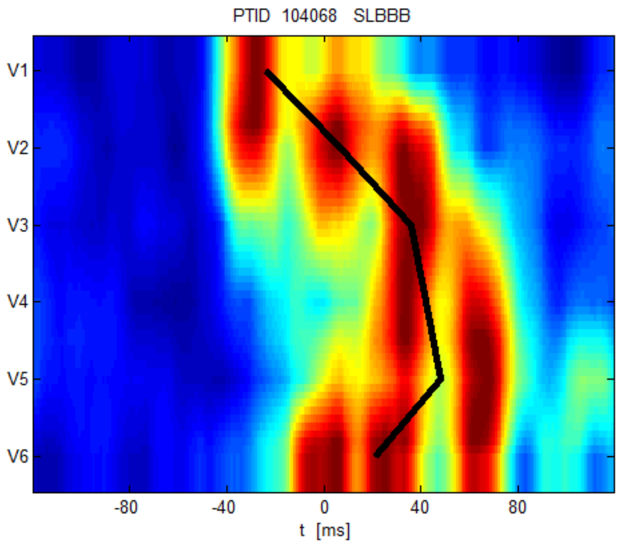
PTID 104068 SLBBB
V1
V2
V3
V4
V5
V6
-80
-40
0
40
80
t [ms]

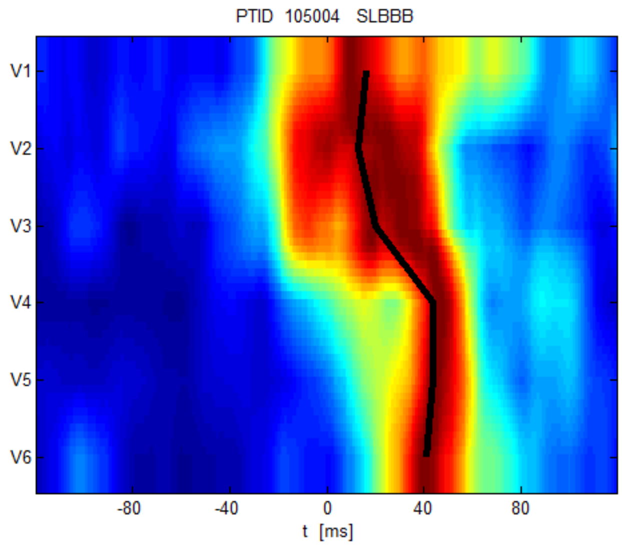
PTID 105004 SLBBB
V1
V2
V3
V4
V5
V6
-80
-40
0
40
80
t [ms]

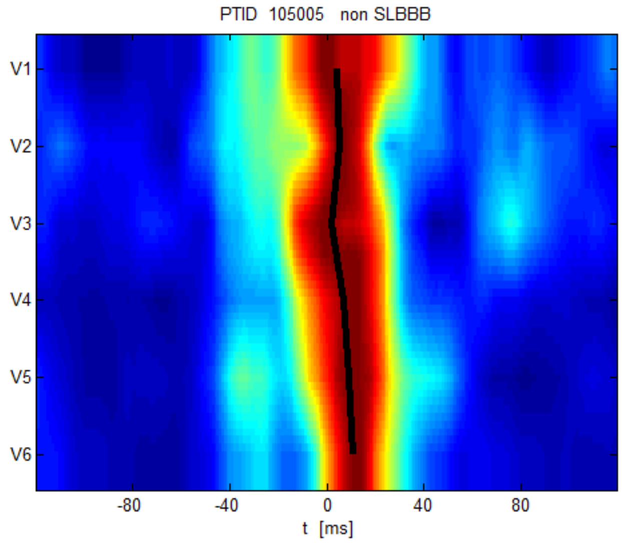
PTID 105005 non SLBBB
V1
V2
V3
V4
V5
V6
-80
-40
0
40
80
t [ms]

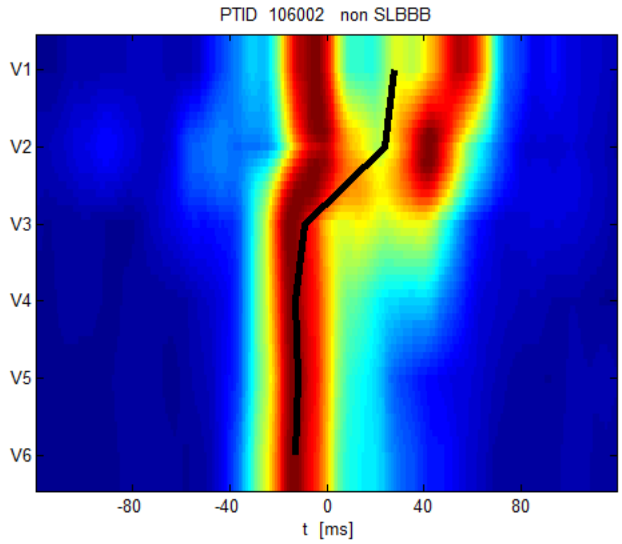
PTID 106002 non SLBBB
V1
V2
V3
V4
V5
V6
-80
-40
0
40
80
t [ms]

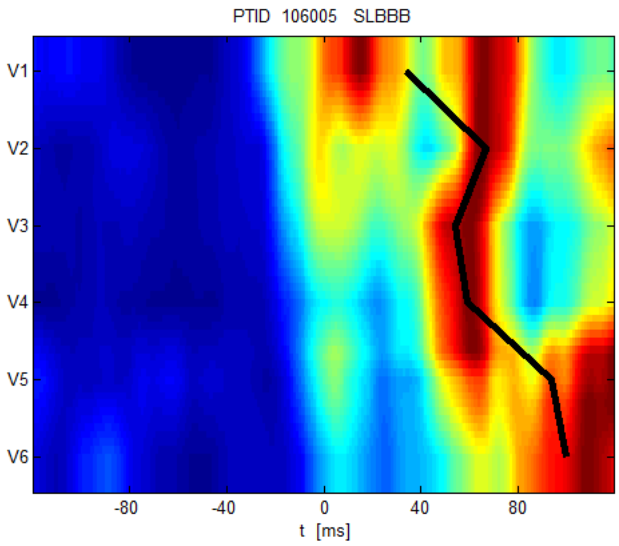
PTID 106005 SLBBB
V1
V2
V3
V4
V5
V6
-80
-40
0
40
80
t [ms]

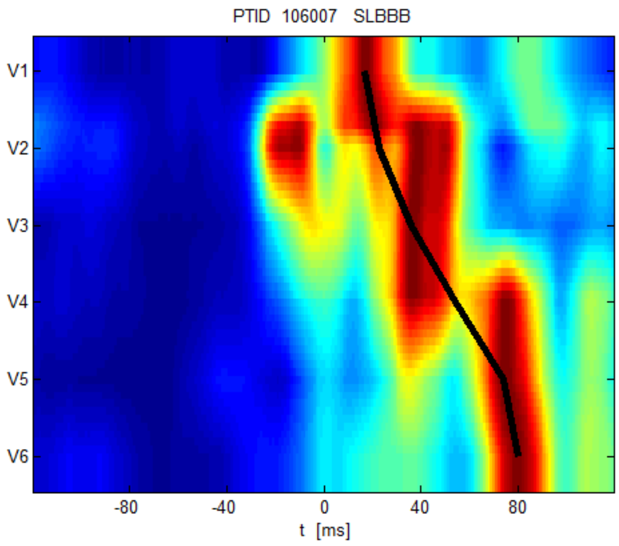
PTID 106007 SLBBB
V1
V2
V3
V4
V5
V6
-80
-40
0
40
80
t [ms]

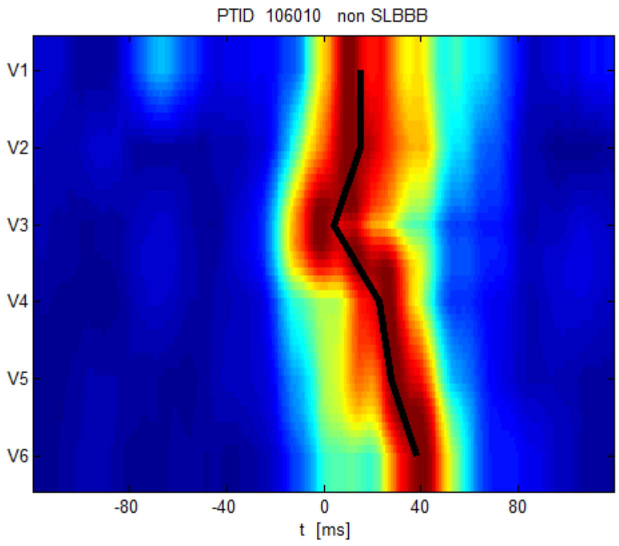
PTID 106010 non SLBBB
V1
V2
V3
V4
V5
V6
-80
-40
0
40
80
t [ms]

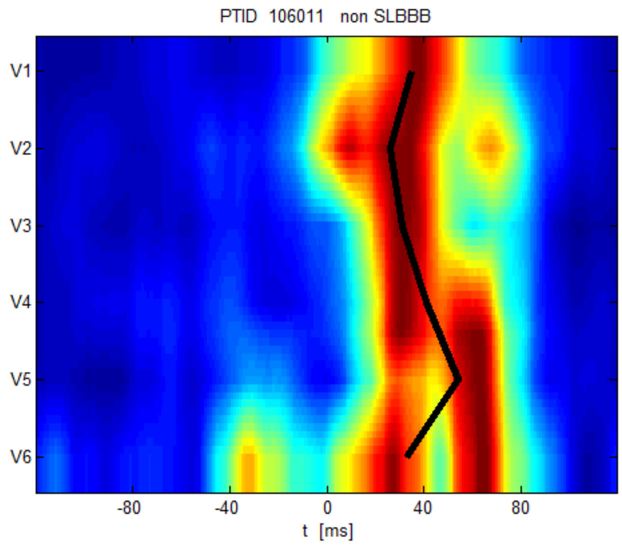
PTID 106011 non SLBBB
V1
V2
V3
V4
V5
V6
-80
-40
0
40
80
t [ms]

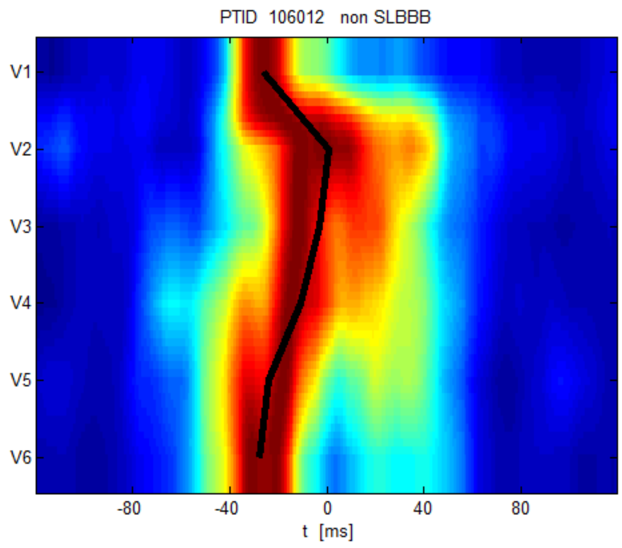
PTID 106012 non SLBBB
V1
V2
V3
V4
V5
V6
-80
-40
0
40
80
t [ms]

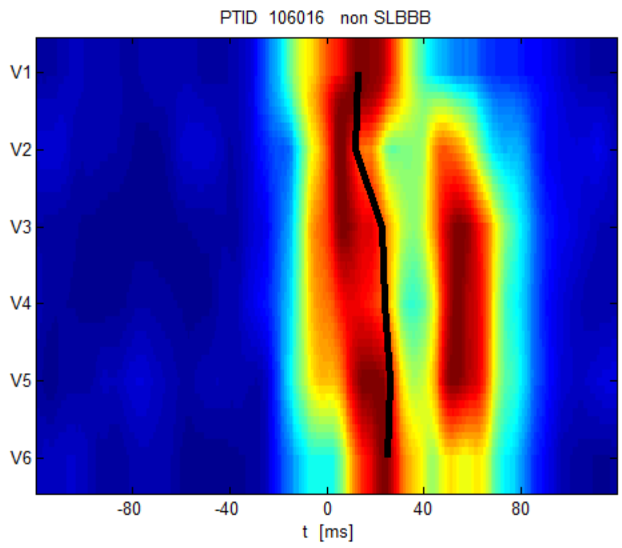
PTID 106016 non SLBBB
V1
V2
V3
V4
V5
V6
-80
-40
0
40
80
t [ms]

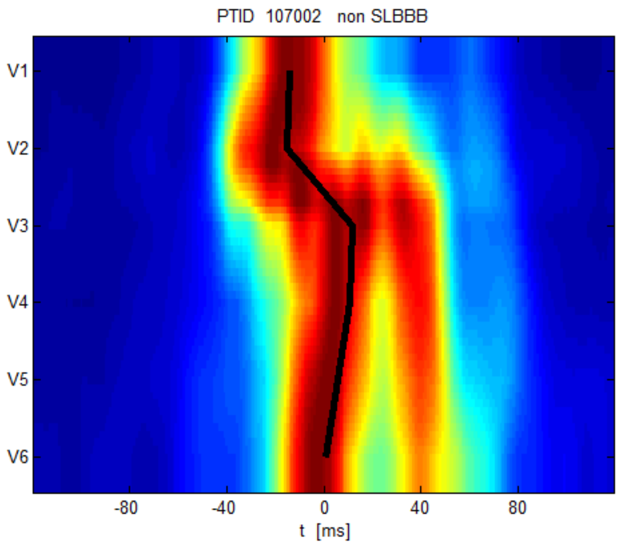
PTID 107002 non SLBBB
V1
V2
V3
V4
V5
V6
-80
-40
0
40
80
t [ms]

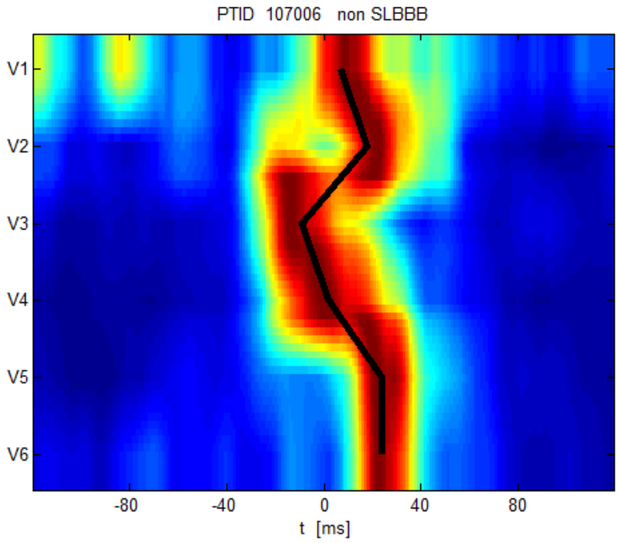
PTID 107006 non SLBBB
V1
V2
V3
V4
V5
V6
-80
-40
0
40
80
t [ms]

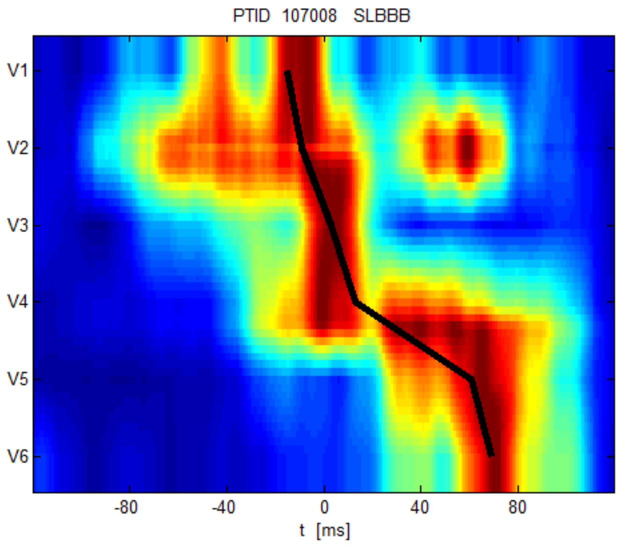
PTID 107008 SLBBB
V1
V2
V3
V4
V5
V6
-80
-40
0
40
80
t [ms]

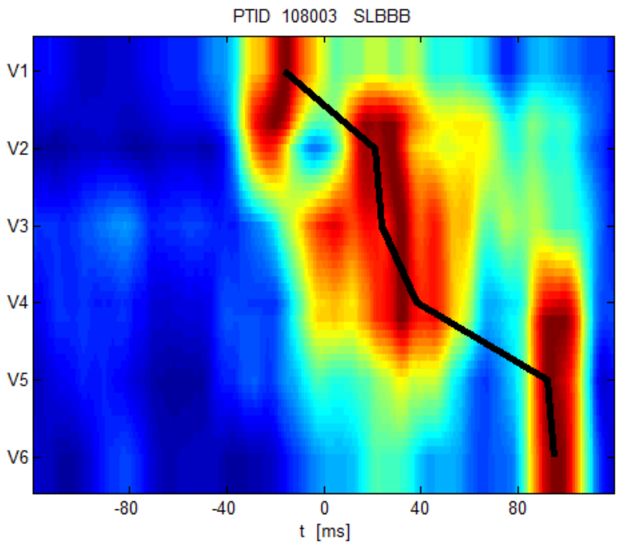
PTID 108003 SLBBB
V1
V2
V3
V4
V5
V6
-80
-40
0
40
80
t [ms]

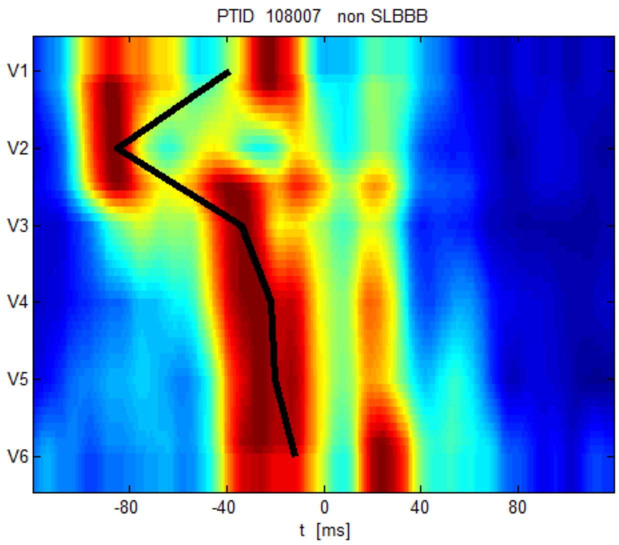
PTID 108007 non SLBBB
V1
V2
V3
V4
V5
V6
-80
-40
0
40
80
t [ms]

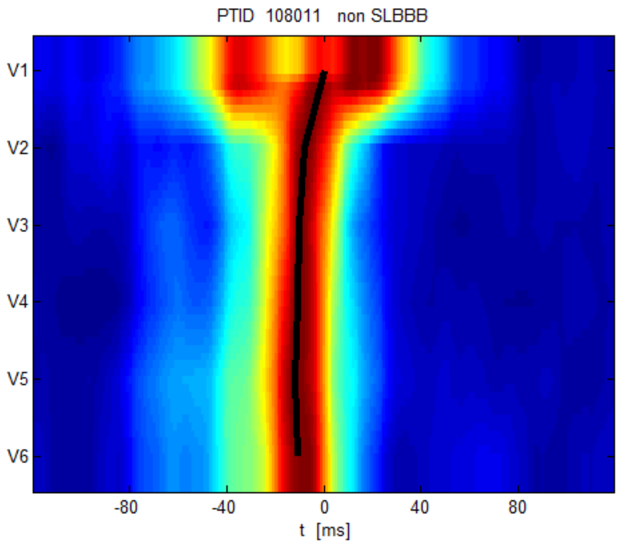
PTID 108011 non SLBBB
V1
V2
V3
V4
V5
V6
-80
-40
0
40
80
t [ms]

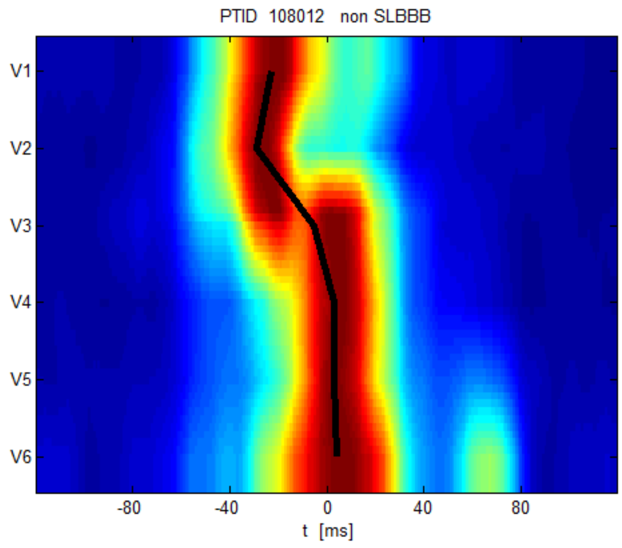
PTID 108012 non SLBBB
V1
V2
V3
V4
V5
V6
-80
-40
0
40
80
t [ms]

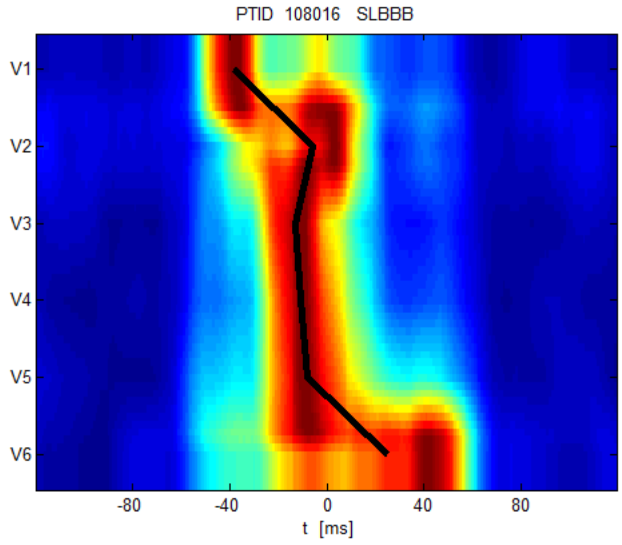
PTID 108016 SLBBB
V1
V2
V3
V4
V5
V6
-80
-40
0
40
80
t [ms]

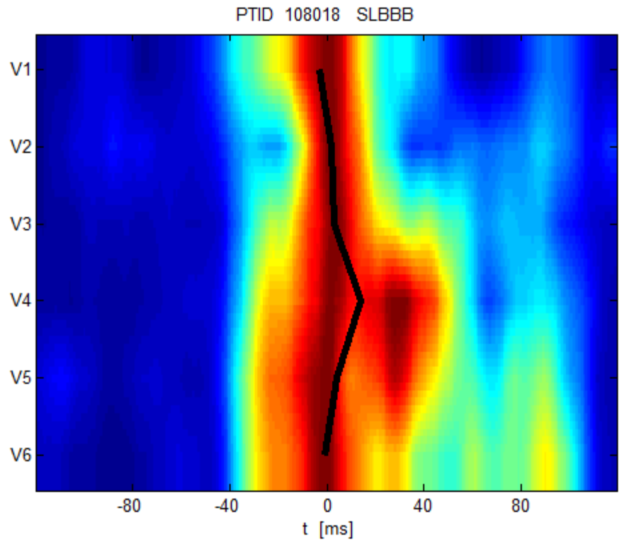
PTID 108018 SLBBB
V1
V2
V3
V4
V5
V6
-80
-40
0
40
80
t [ms]

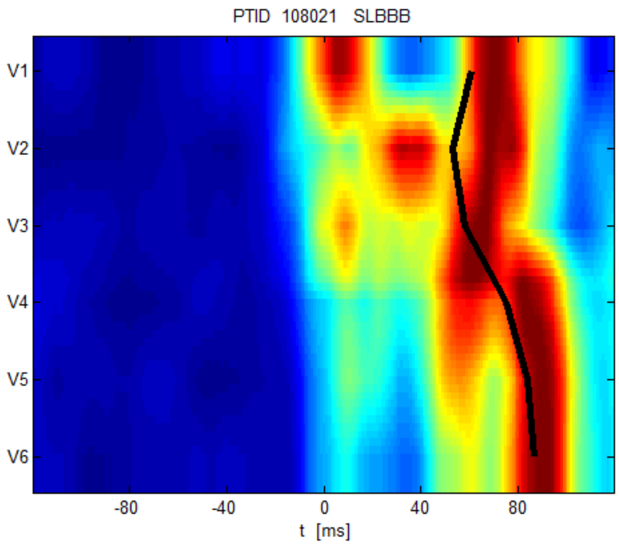
PTID 108021 SLBBB
V1
V2
V3
V4
V5
V6
-80
-40
0
40
80
t [ms]

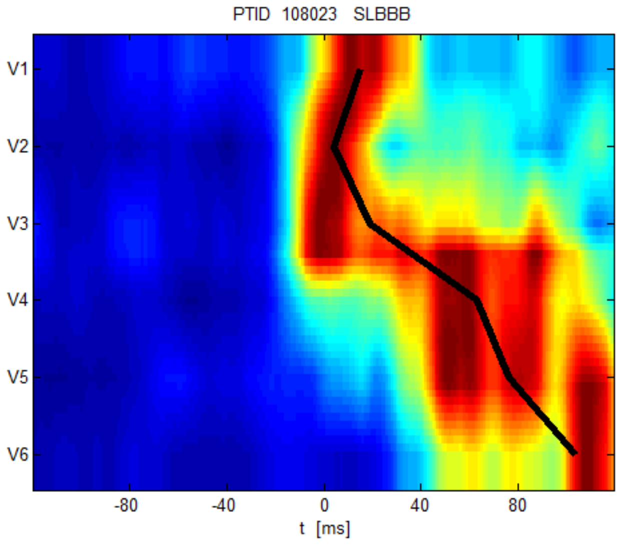
PTID 108023 SLBBB
V1
V2
V3
V4
V5
V6
-80
-40
0
40
80
t [ms]

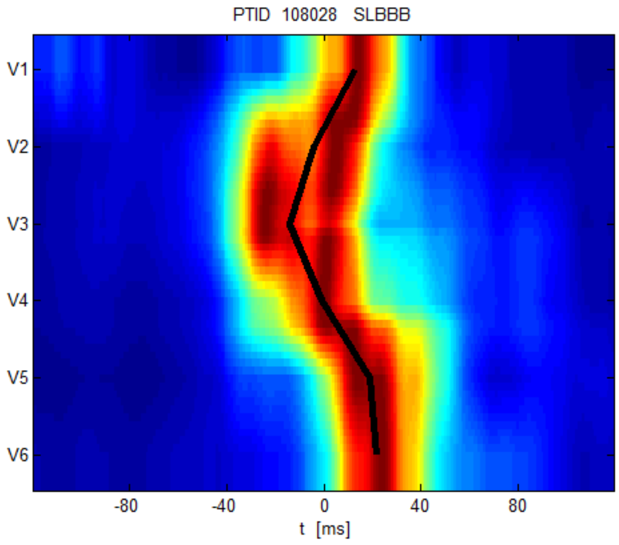
PTID 108028 SLBBB
V1
V2
V3
V4
V5
V6
-80
-40
0
40
80
t [ms]

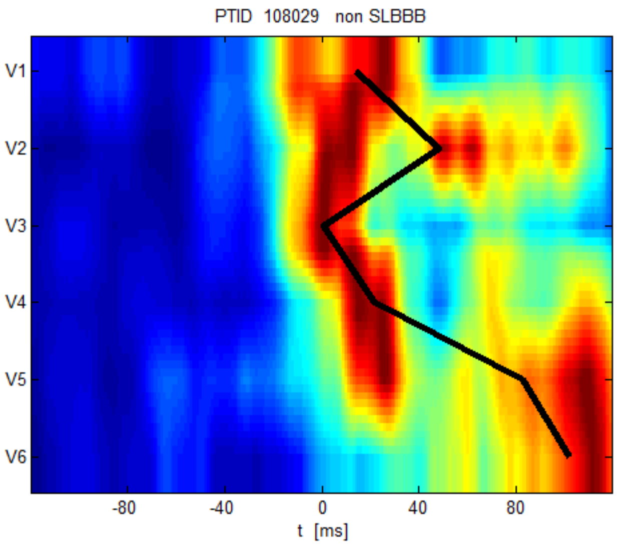
PTID 108029 non SLBBB
V1
V2
V3
V4
V5
V6
-80
-40
0
40
80
t [ms]

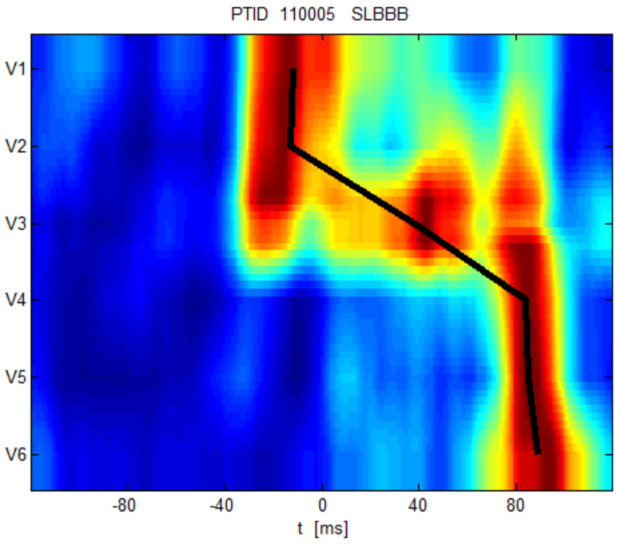
PTID 110005 SLBBB
V1
V2
V3
V4
V5
V6
-80
-40
0
40
80
t [ms]

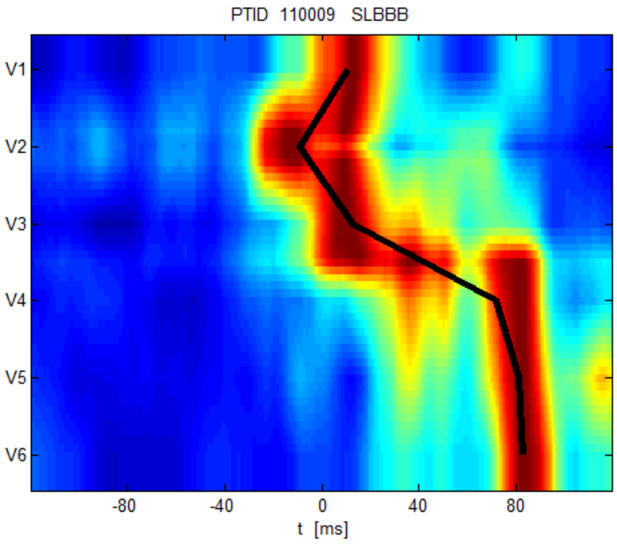
PTID 110009 SLBBB
V1
V2
V3
V4
V5
V6
-80
-40
0
40
80
t [ms]

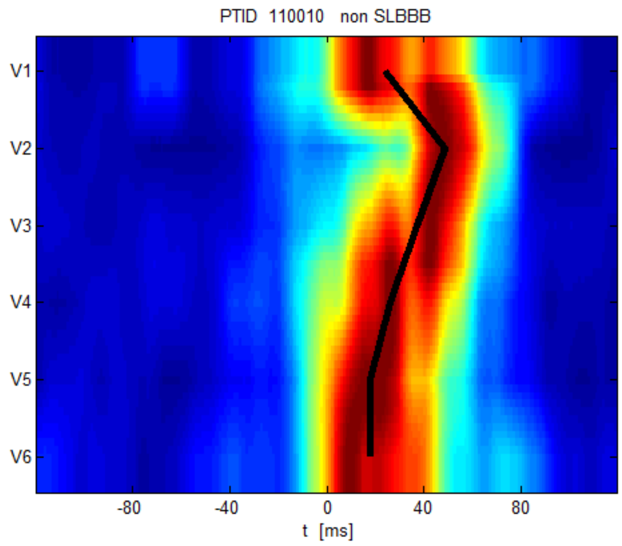
PTID 110010 non SLBBB
V1
V2
V3
V4
V5
V6
-80
-40
0
40
80
t [ms]

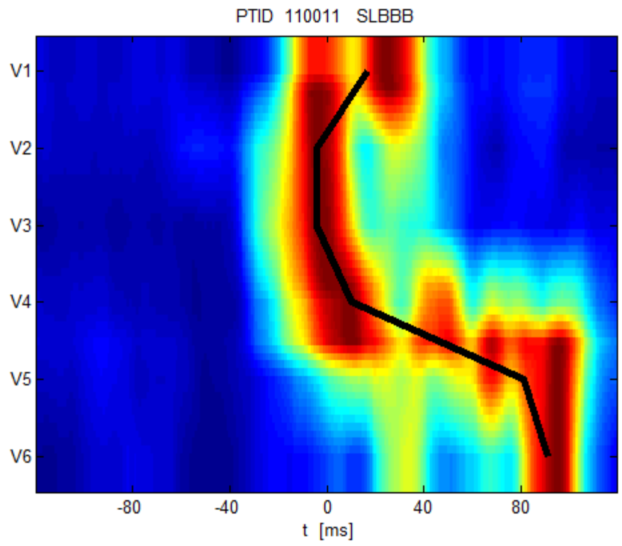
PTID 110011 SLBBB
V1
V2
V3
V4
V5
V6
-80
-40
0
40
80
t [ms]

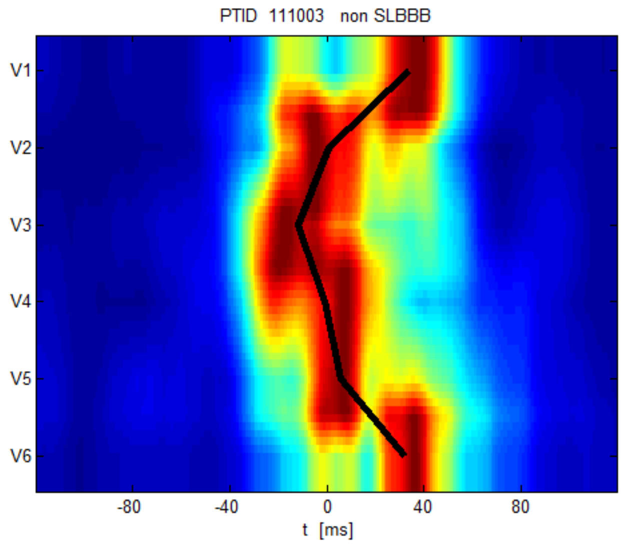
PTID 111003 non SLBBB
V1
V2
V3
V4
V5
V6
-80
-40
0
40
80
t [ms]

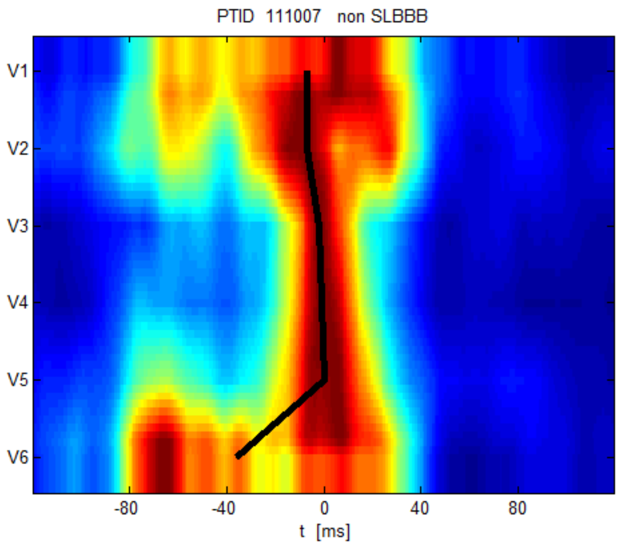
PTID 111007 non SLBBB
V1
V2
V3
V4
V5
V6
-80
-40
0
40
80
t [ms]

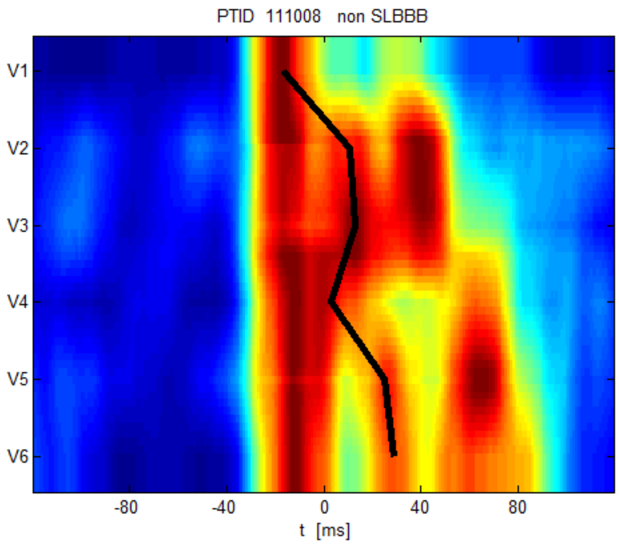
PTID 111008 non SLBBB
V1
V2
V3
V4
V5
V6
-80
-40
0
40
80
t [ms]

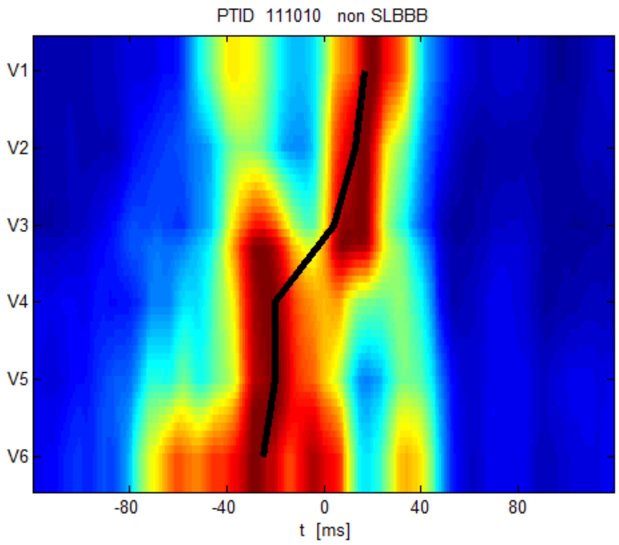
PTID 111010 non SLBBB
V1
V2
V3
V4
V5
V6
-80
-40
0
40
80
t [ms]

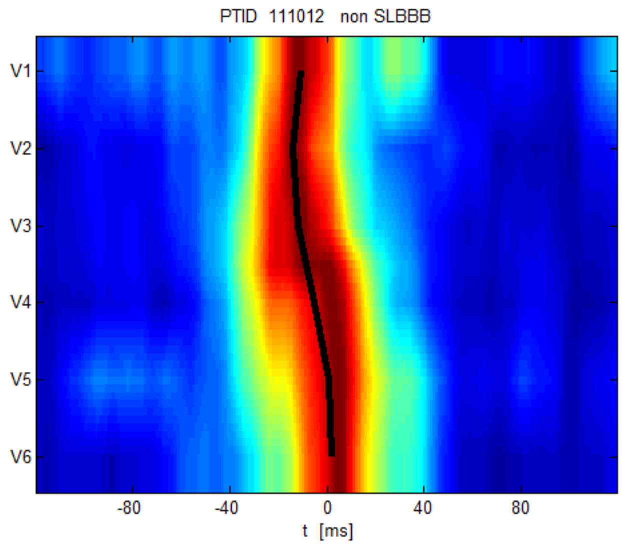
PTID 111012 non SLBBB
V1
V2
V3
V4
V5
V6
-80
-40
0
40
80
t [ms]

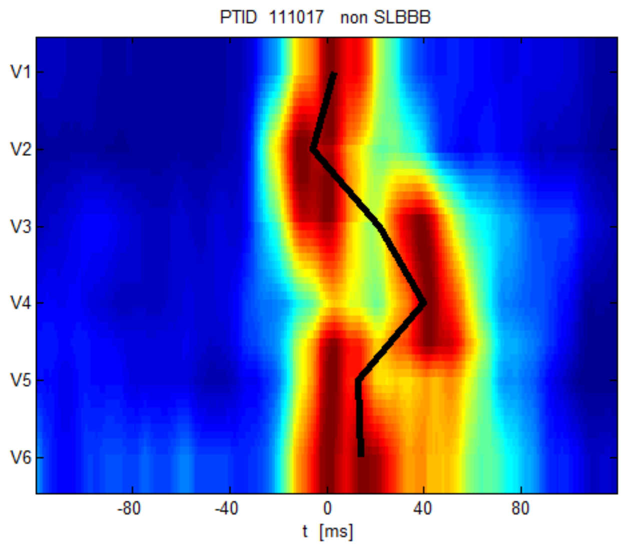
PTID 111017 non SLBBB
V1
V2
V3
V4
V5
V6
-80
-40
0
40
80
t [ms]

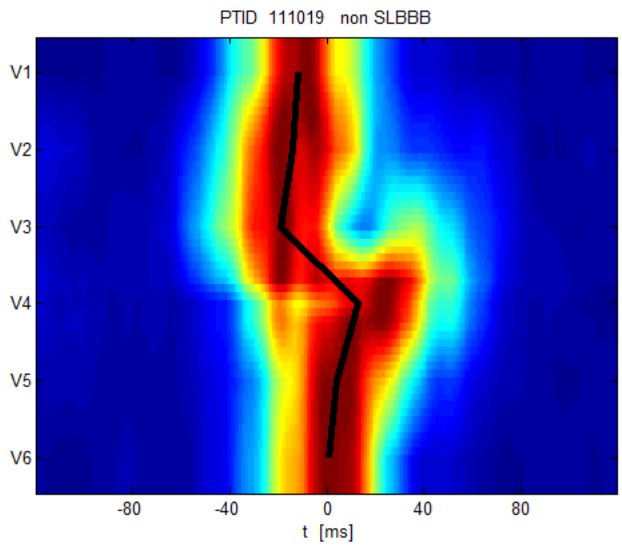
PTID 111019 non SLBBB
V1
V2
V3
V4
V5
V6
-80
-40
0
40
80
t [ms]

PTID 111021 SLBBB

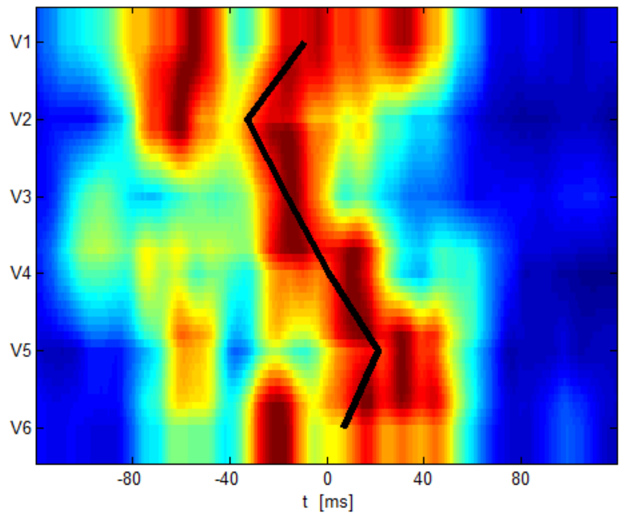

PTID 111022 non SLBBB

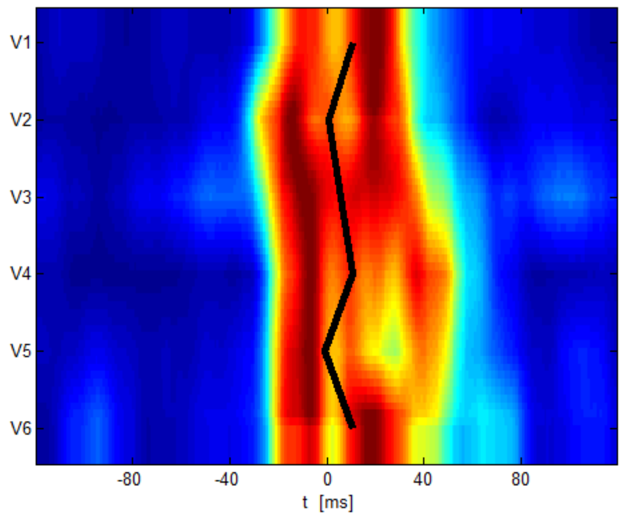

PTID 111024 SLBBB

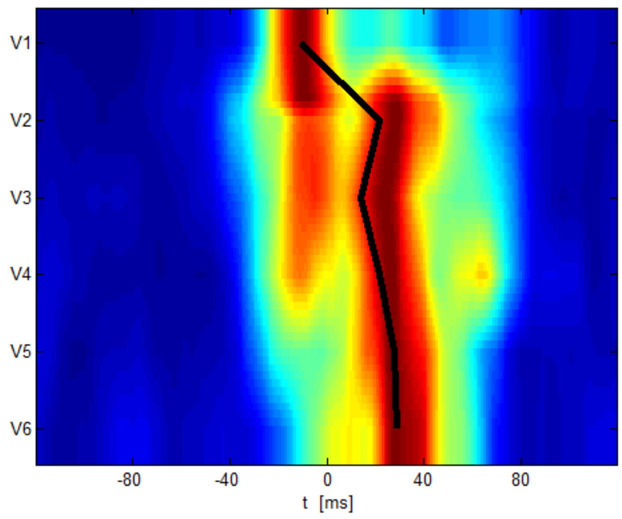

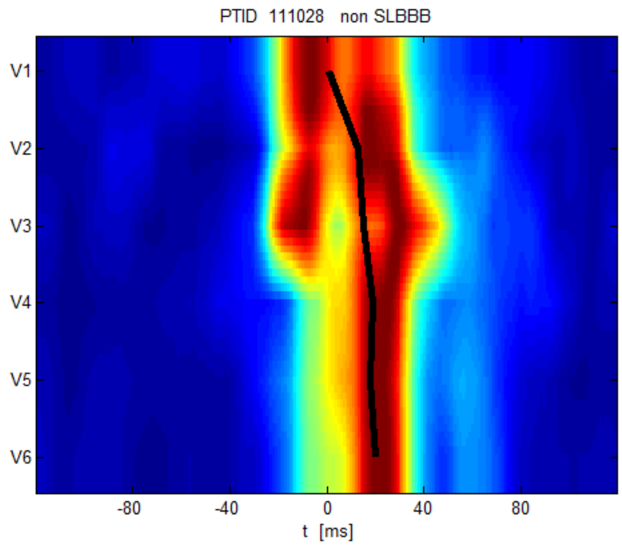
PTID 111028 non SLBBB
V1
V2
V3
V4
V5
V6
-80
-40
0
40
80
t [ms]

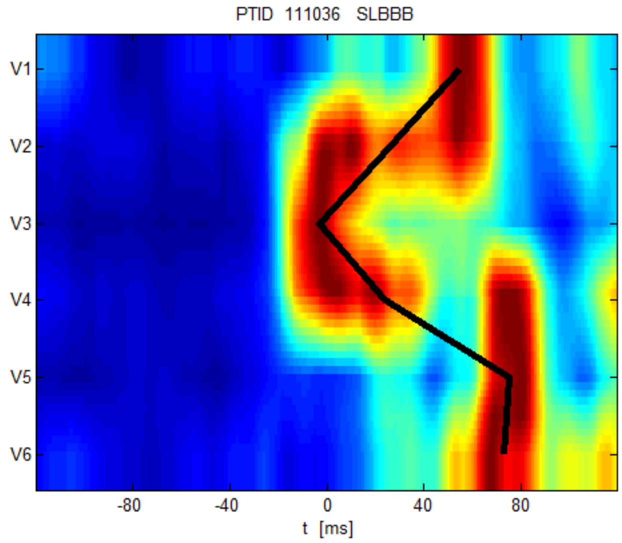
PTID 111036 SLBBB
V1
V2
V3
V4
V5
V6
-80
-40
0
40
80
t [ms]

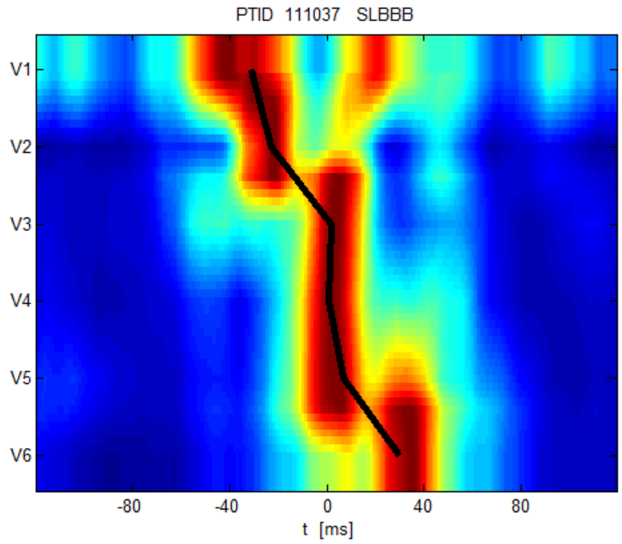
PTID 111037 SLBBB
V1
V2
V3
V4
V5
V6
-80
-40
0
40
80
t [ms]

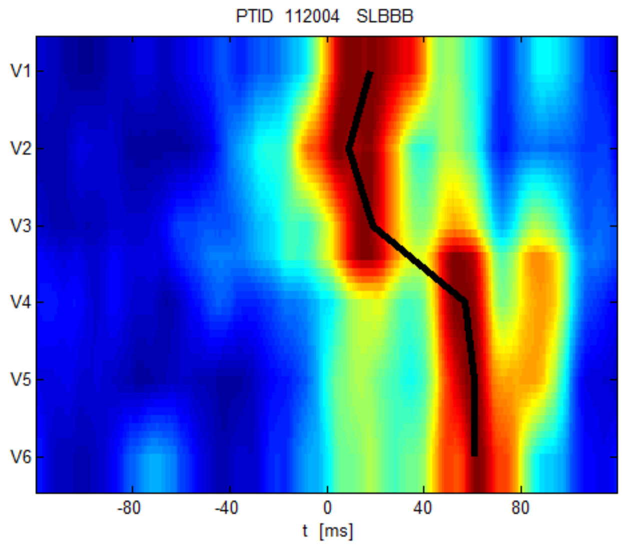
PTID 112004 SLBBB
V1
V2
V3
V4
V5
V6
-80
-40
0
40
80
t [ms]

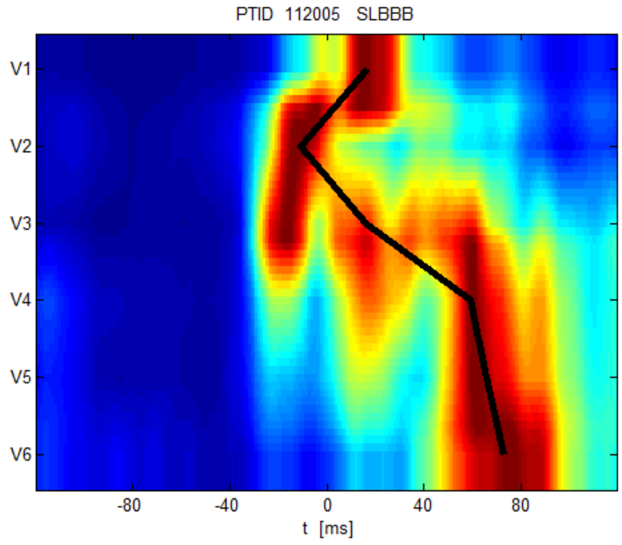
PTID 112005 SLBBB
V1
V2
V3
V4
V5
V6
-80
-40
0
40
80
t [ms]

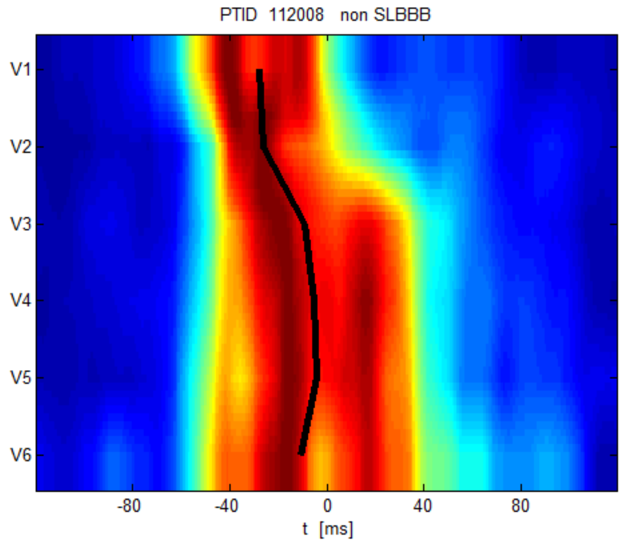
PTID 112008 non SLBBB
V1
V2
V3
V4
V5
V6
-80
-40
0
40
80
t [ms]

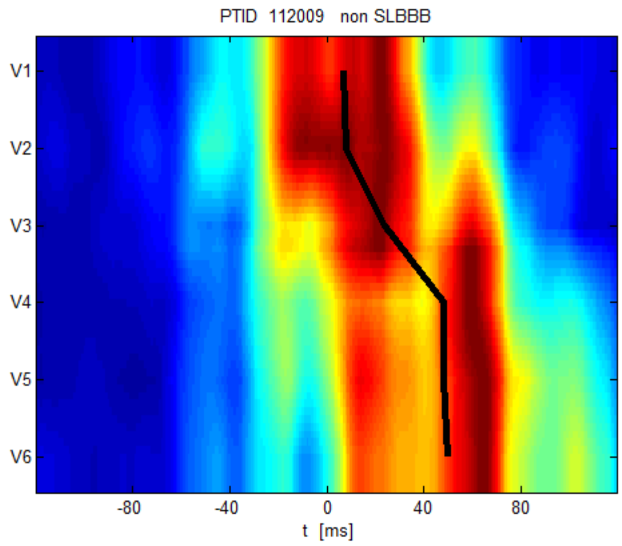
PTID 112009 non SLBBB
V1
V2
V3
V4
V5
V6
-80
-40
0
40
80
t [ms]

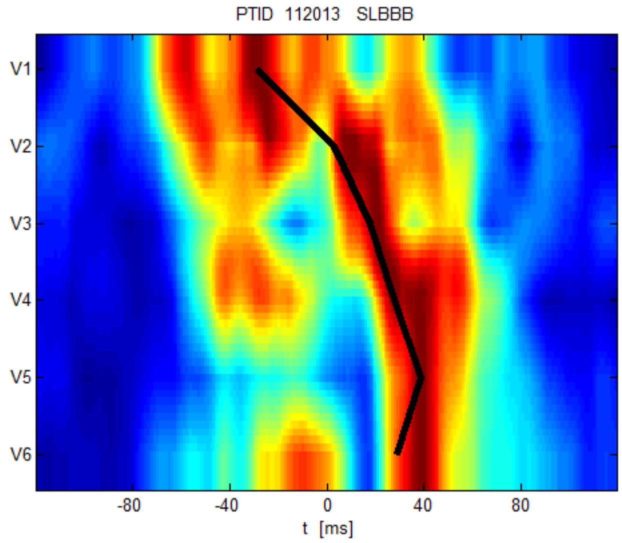
PTID 112013 SLBBB
V1
V2
V3
V4
V5
V6
-80
-40
0
40
80
t [ms]

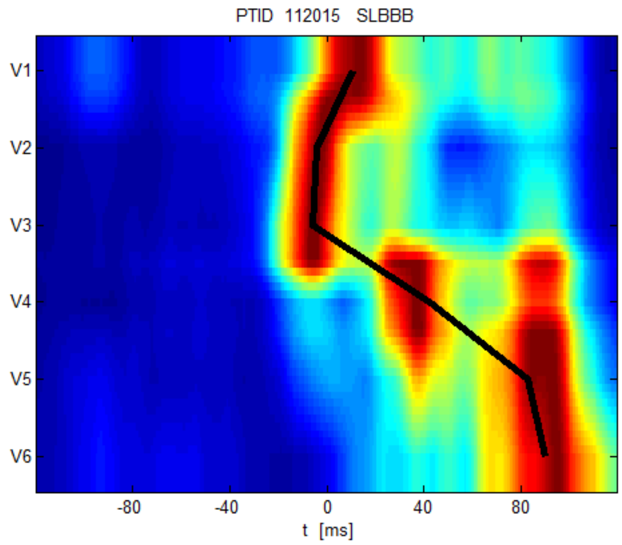
PTID 112015 SLBBB
V1
V2
V3
V4
V5
V6
-80
-40
0
40
80
t [ms]

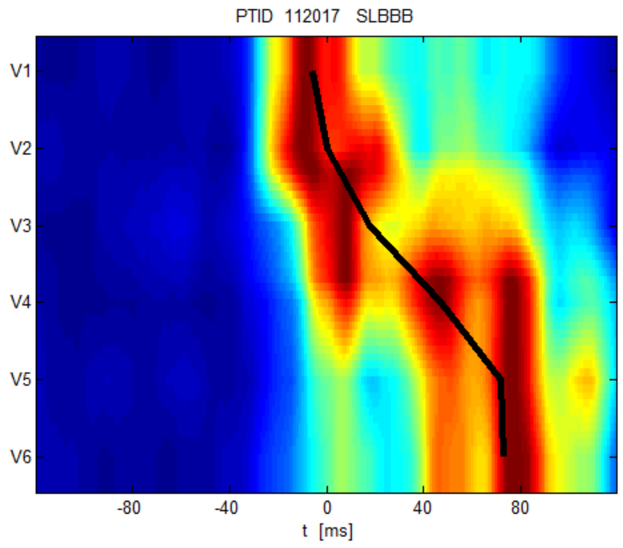
PTID 112017 SLBBB
V1
V2
V3
V4
V5
V6
-80
-40
0
40
80
t [ms]

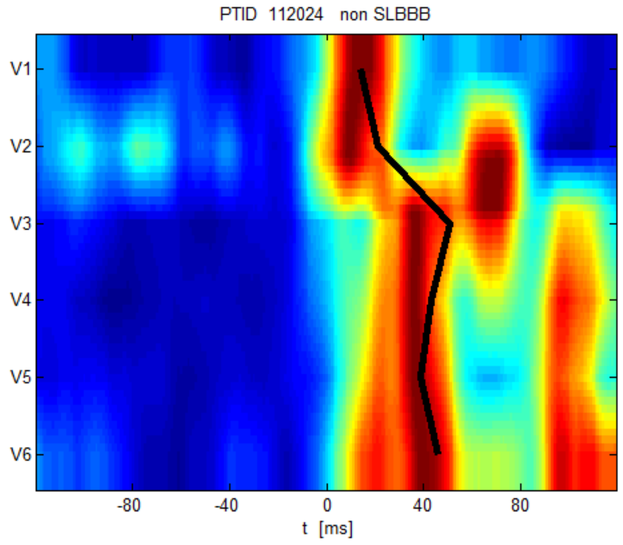
PTID 112024 non SLBBB
V1
V2
V3
V4
V5
V6
-80
-40
0
40
80
t [ms]

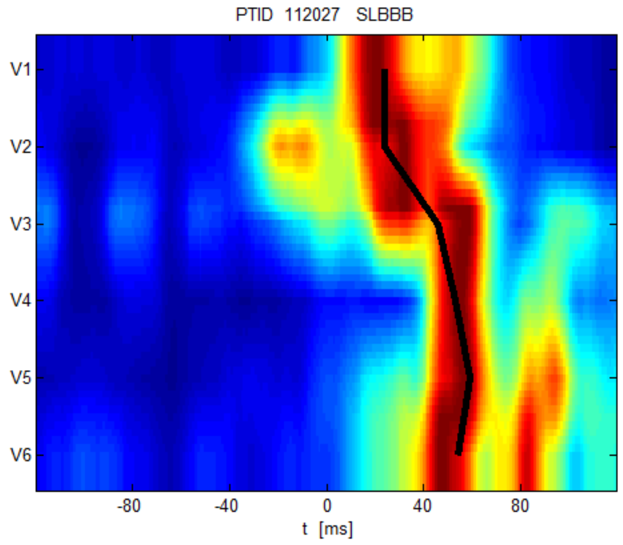
PTID 112027 SLBBB
V1
V2
V3
V4
V5
V6
-80
-40
0
40
80
t [ms]

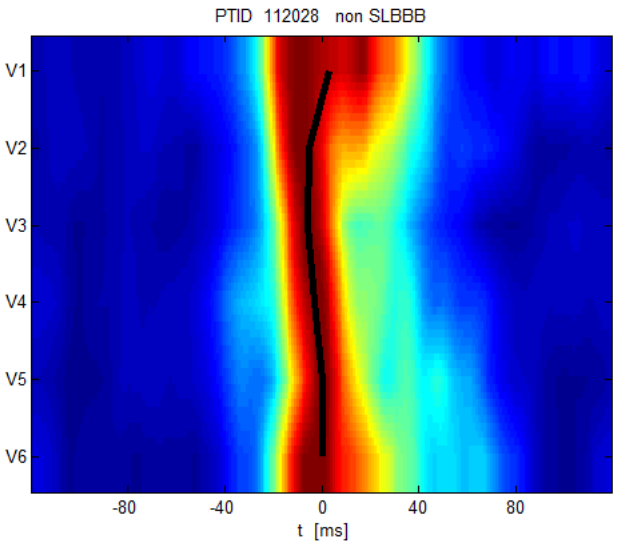
PTID 112028 non SLBBB
V1
V2
V3
V4
V5
V6
-80
-40
0
40
80
t [ms]

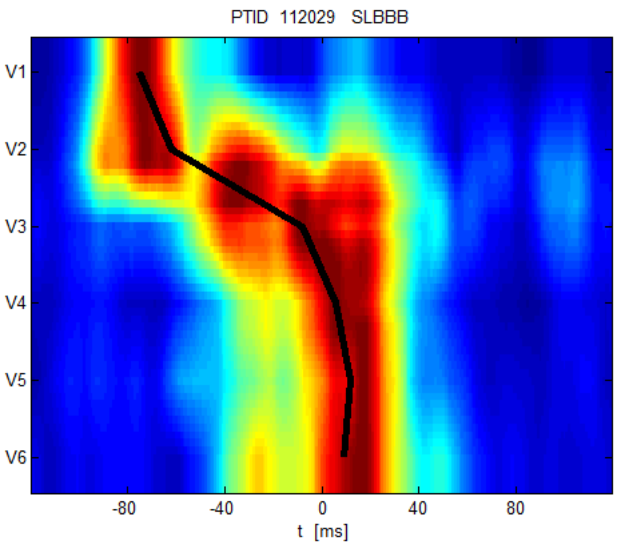
PTID 112029 SLBBB
V1
V2
V3
V4
V5
V6
-80
-40
0
40
80
t [ms]

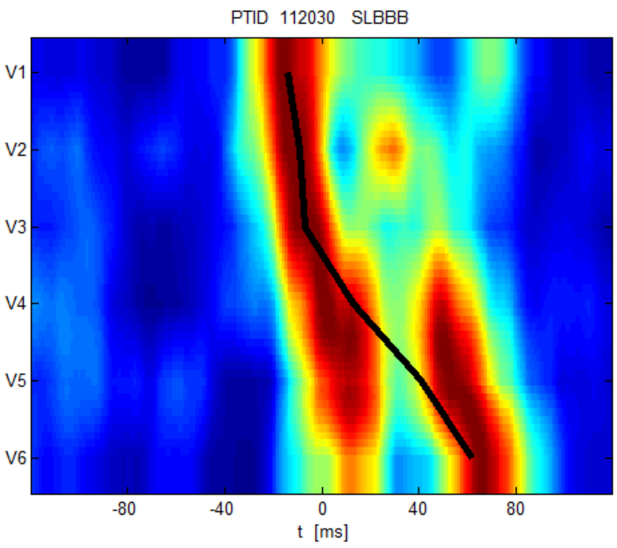
PTID 112030 SLBBB
V1
V2
V3
V4
V5
V6
-80
-40
0
40
80
t [ms]

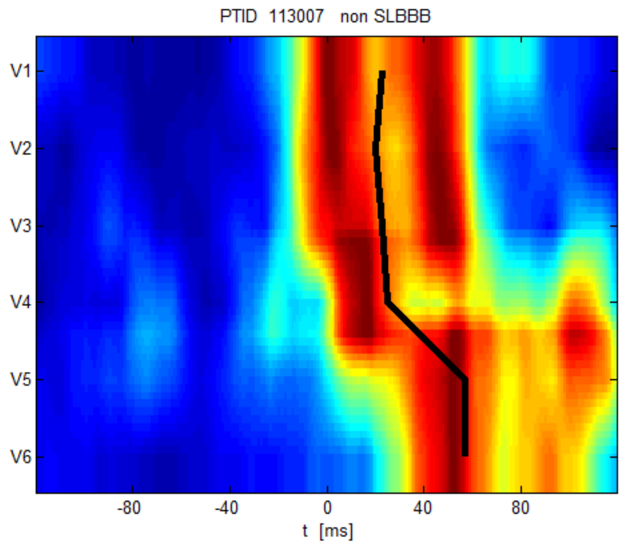
PTID 113007 non SLBBB
V1
V2
V3
V4
V5
V6
-80
-40
0
40
80
t [ms]

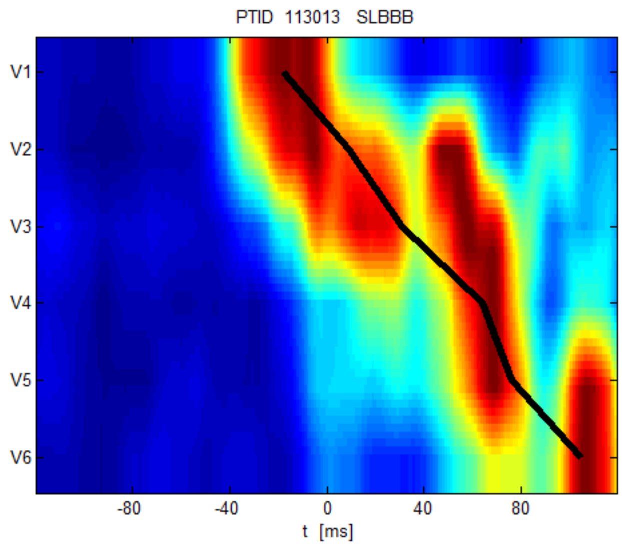
PTID 113013 SLBBB
V1
V2
V3
V4
V5
V6
-80
-40
0
40
80
t [ms]

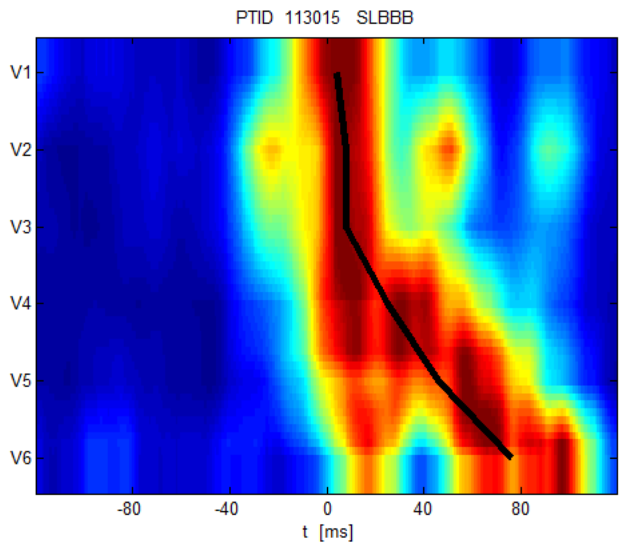
PTID 113015 SLBBB
V1
V2
V3
V4
V5
V6
-80
-40
0
40
80
t [ms]

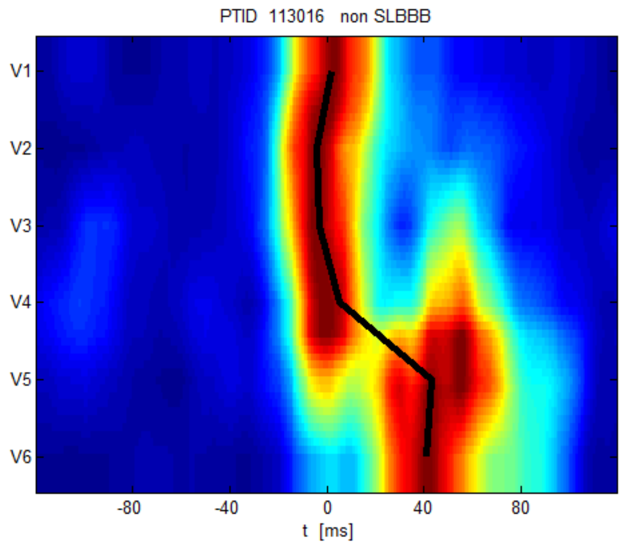
PTID 113016 non SLBBB
V1
V2
V3
V4
V5
V6
-80
-40
0
40
80
t [ms]

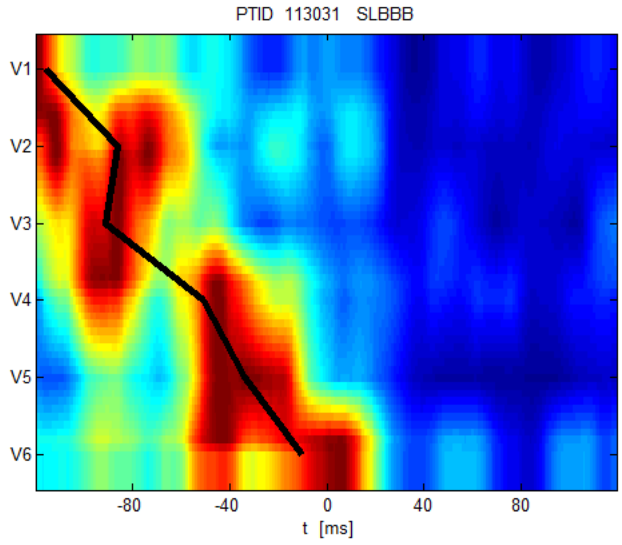
PTID 113031 SLBBB
V1
V2
V3
V4
V5
V6
-80
-40
0
40
80
t [ms]

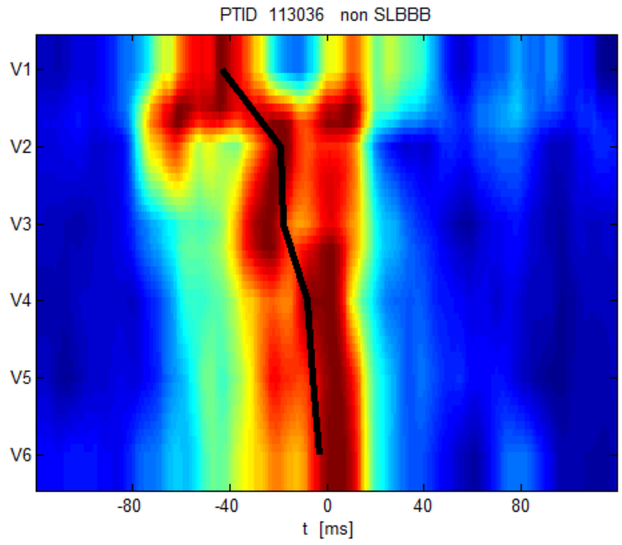
PTID 113036 non SLBBB
V1
V2
V3
V4
V5
V6
-80
-40
0
40
80
t [ms]

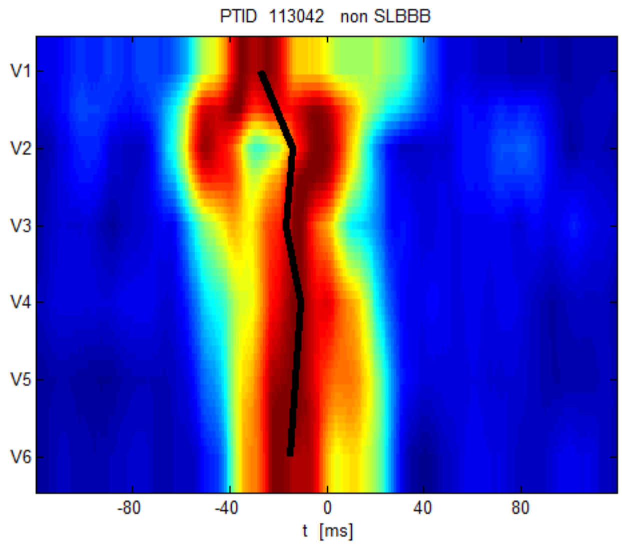
PTID 113042 non SLBBB
V1
V2
V3
V4
V5
V6
-80
-40
0
40
80
t [ms]

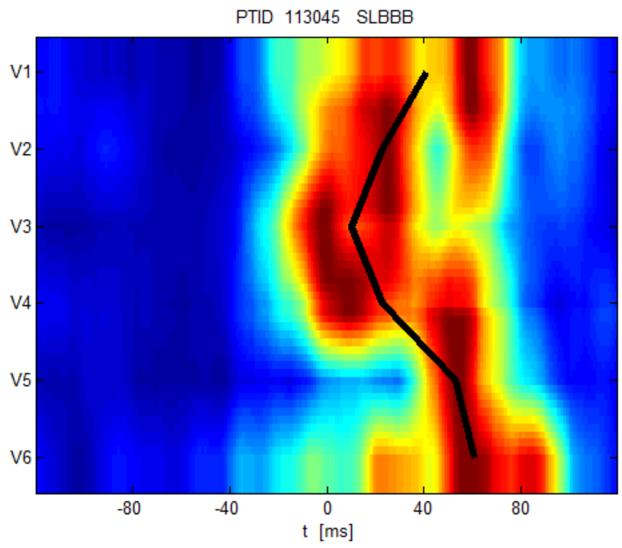
PTID 113045 SLBBB
V1
V2
V3
V4
V5
V6
-80
-40
0
40
80
t [ms]

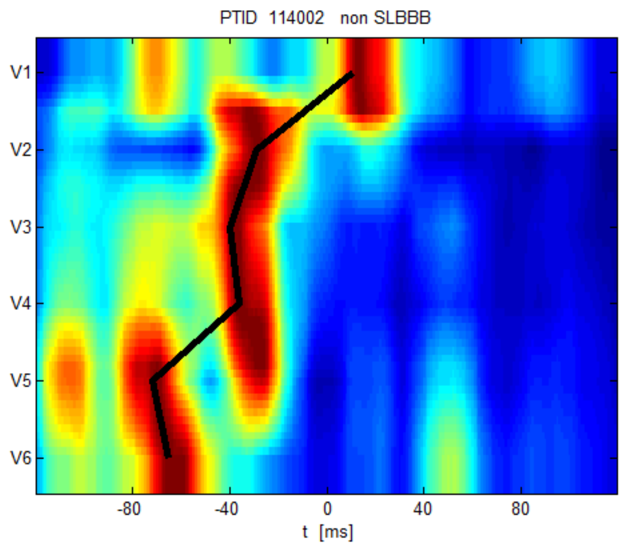
PTID 114002 non SLBBB
V1
V2
V3
V4
V5
V6
-80
-40
0
40
80
t [ms]

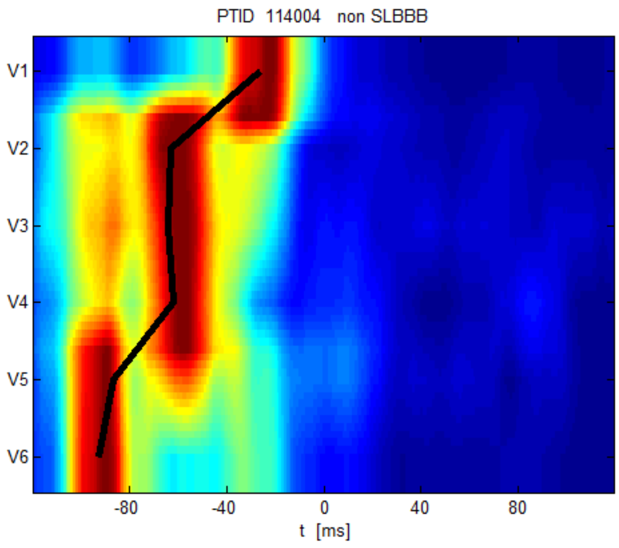
PTID 114004 non SLBBB
V1
V2
V3
V4
V5
V6
-80
-40
0
40
80
t [ms]

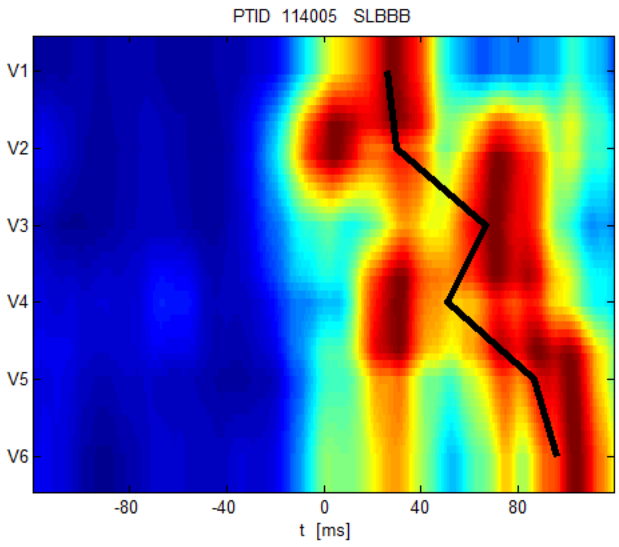
PTID 114005 SLBBB
V1
V2
V3
V4
V5
V6
-80
-40
0
40
80
t [ms]

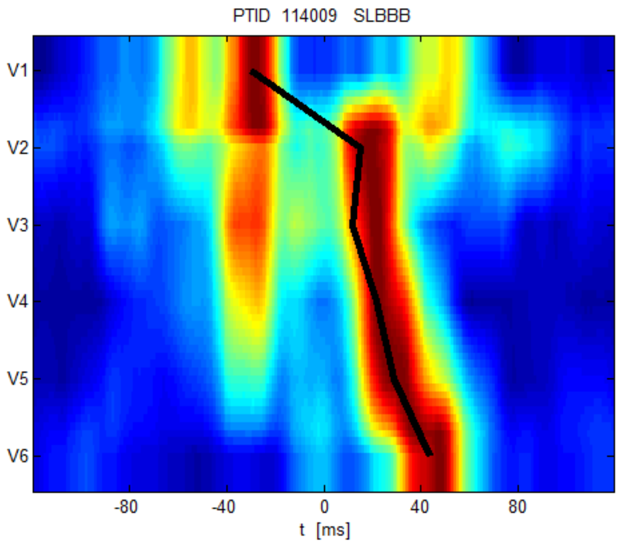
PTID 114009 SLBBB
V1
V2
V3
V4
V5
V6
-80
-40
0
40
80
t [ms]

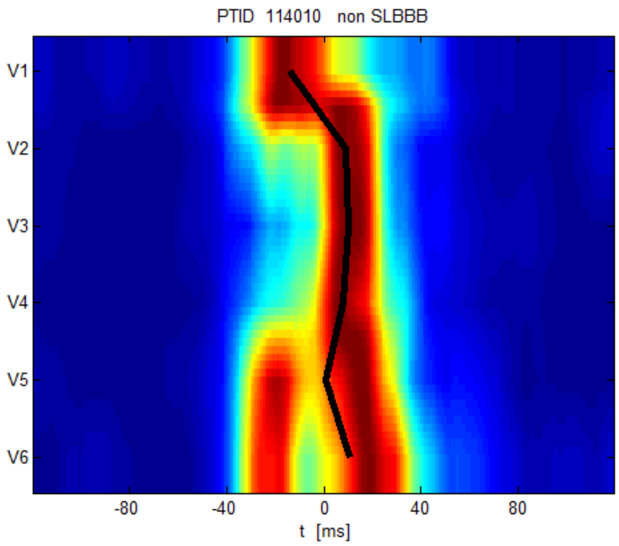
PTID 114010 non SLBBB
V1
V2
V3
V4
V5
V6
-80
-40
0
40
80
t [ms]

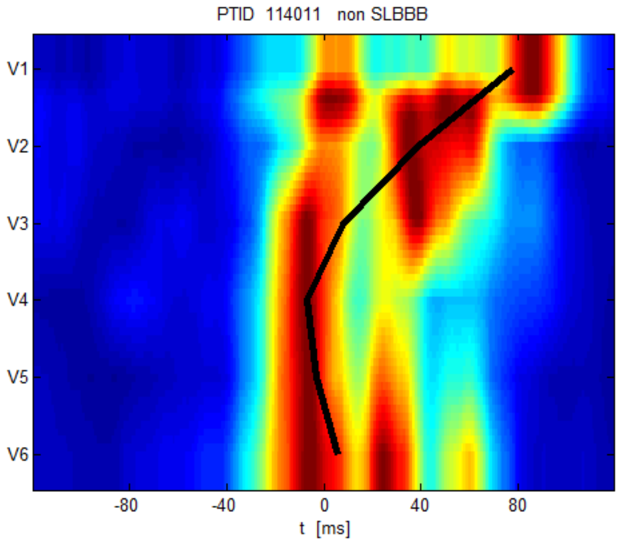
PTID 114011 non SLBBB
V1
V2
V3
V4
V5
V6
-80
-40
0
40
80
t [ms]

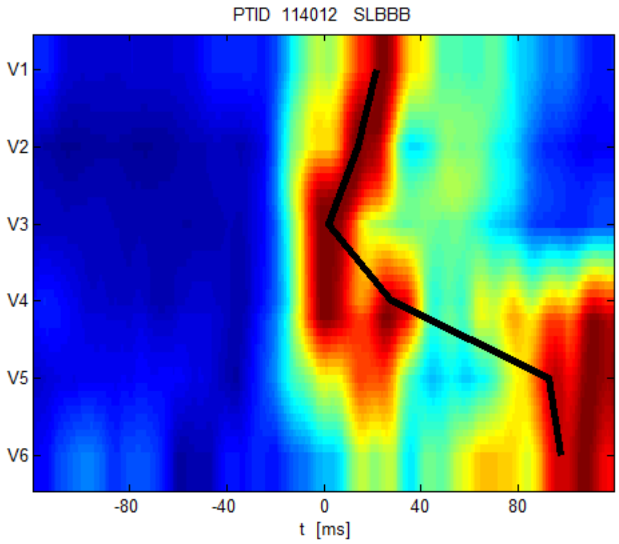
PTID 114012 SLBBB
V1
V2
V3
V4
V5
V6
-80
-40
0
40
80
t [ms]

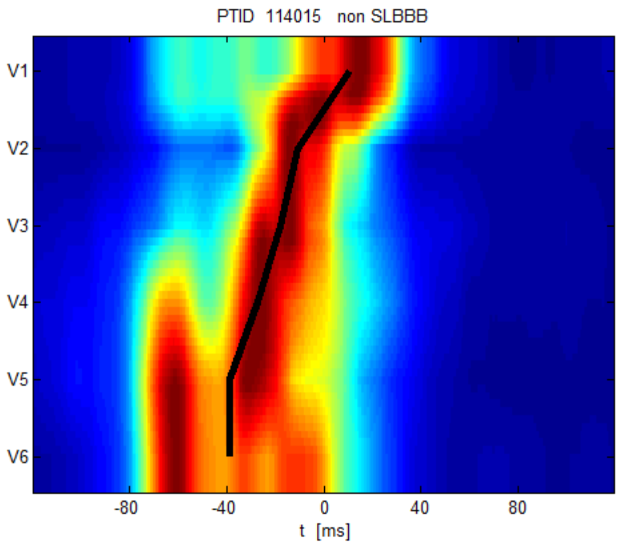

PTID 114015 non SLBBB
V1
V2
V3
V4
V5
V6
-80
-40
0
40
80
t [ms]

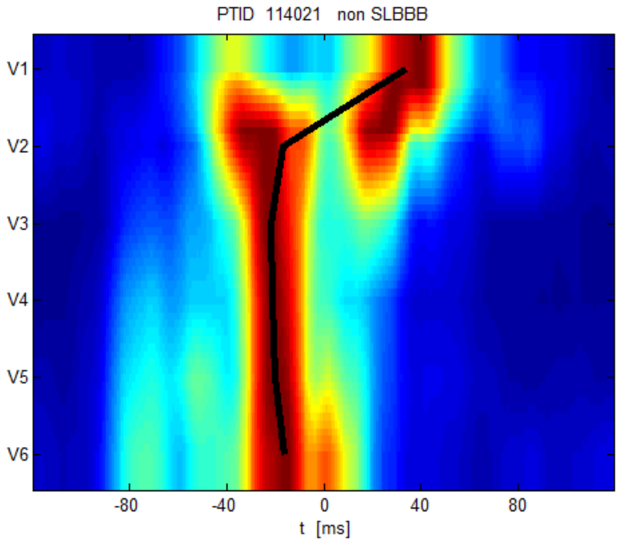

PTID 114021 non SLBBB
V1
V2
V3
V4
V5
V6
-80
-40
0
40
80
t [ms]

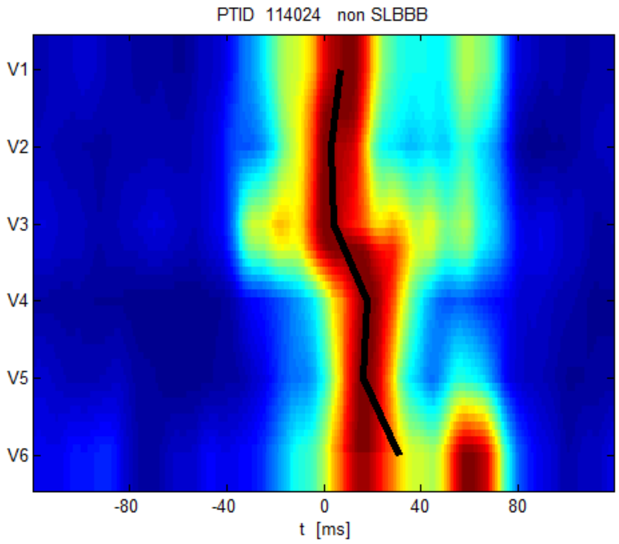

PTID 114024 non SLBBB
V1
V2
V3
V4
V5
V6
-80
-40
0
40
80
t [ms]

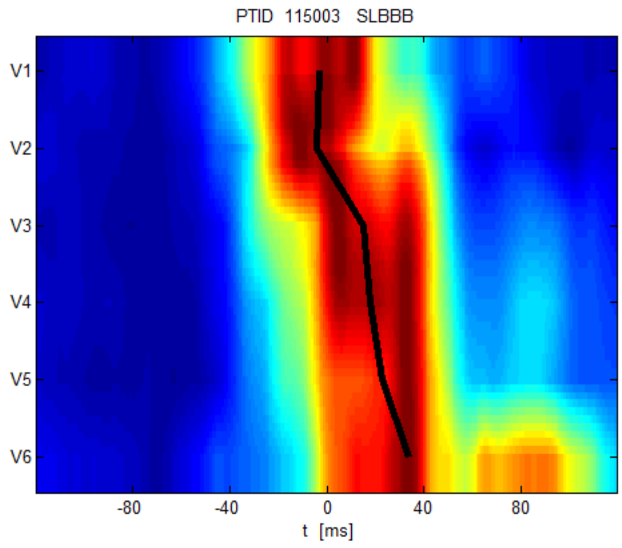
PTID 115003 SLBBB
V1
V2
V3
V4
V5
V6
-80
-40
0
40
80
t [ms]

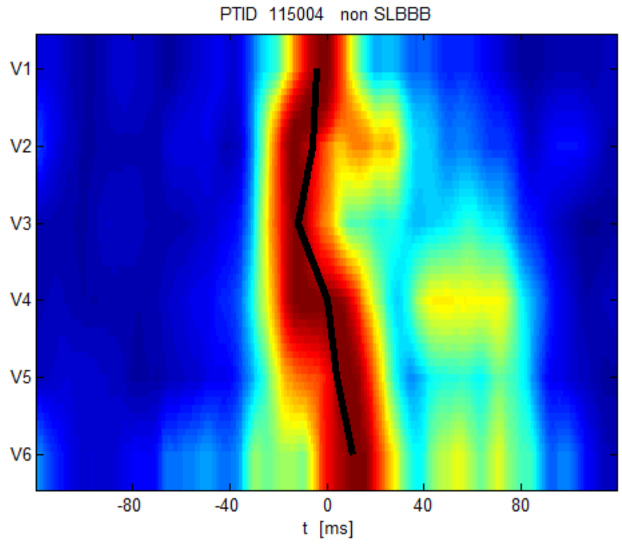
PTID 115004 non SLBBB
V1
V2
V3
V4
V5
V6
-80
-40
0
40
80
t [ms]

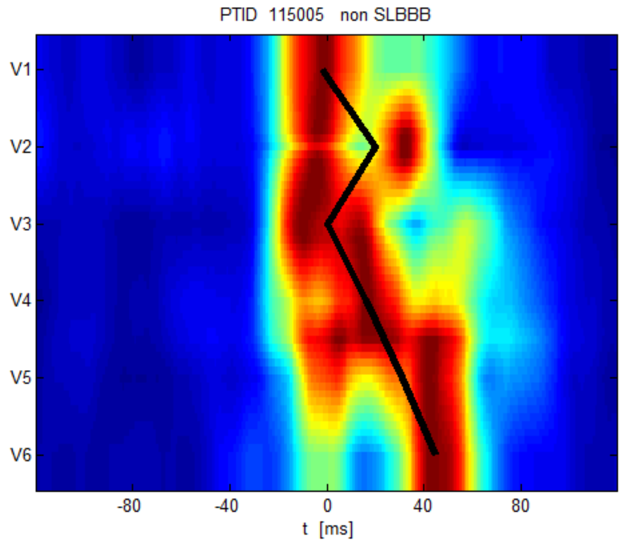
PTID 115005 non SLBBB
V1
V2
V3
V4
V5
V6
-80
-40
0
40
80
t [ms]

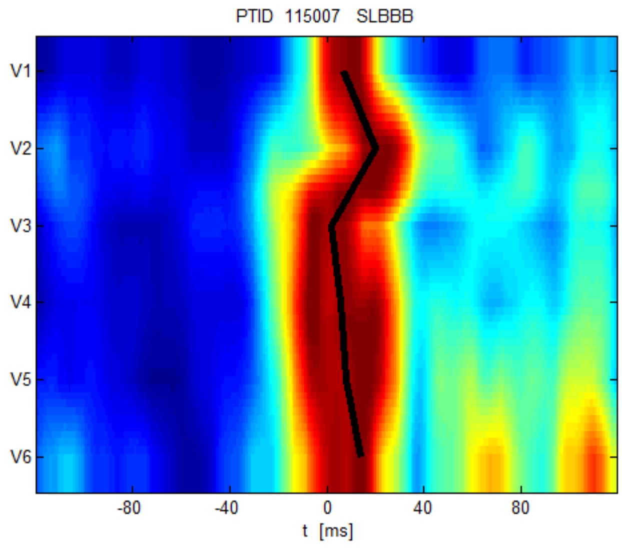
PTID 115007 SLBBB
V1
V2
V3
V4
V5
V6
-80
-40
0
40
80
t [ms]

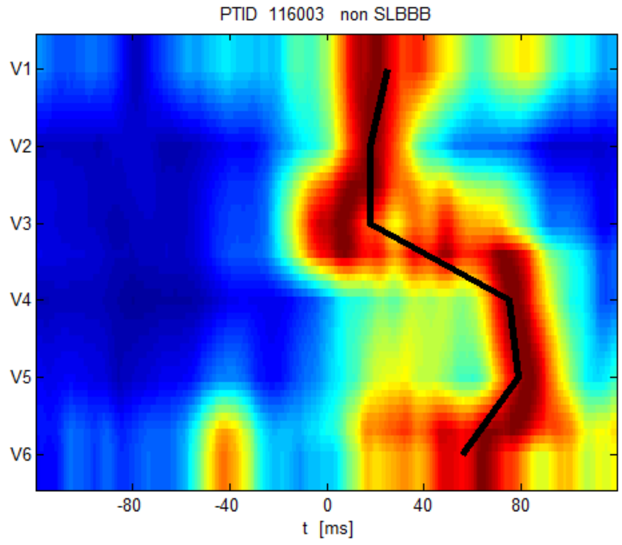
PTID 116003 non SLBBB
V1
V2
V3
V4
V5
V6
-80
-40
0
40
80
t [ms]

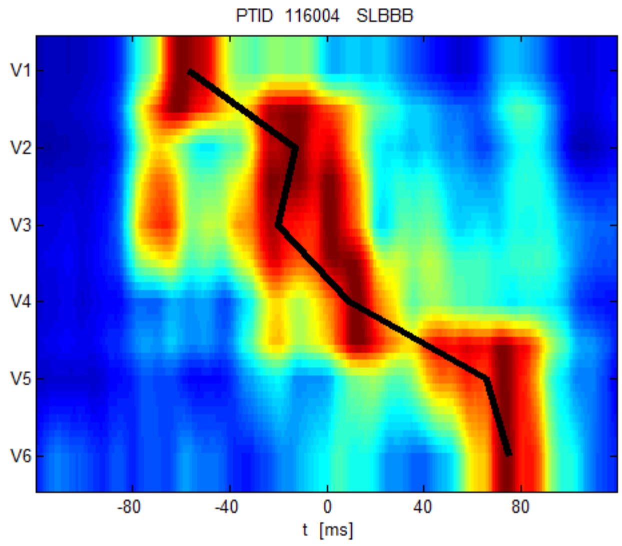
PTID 116004 SLBBB
V1
V2
V3
V4
V5
V6
-80
-40
0
40
80
t [ms]

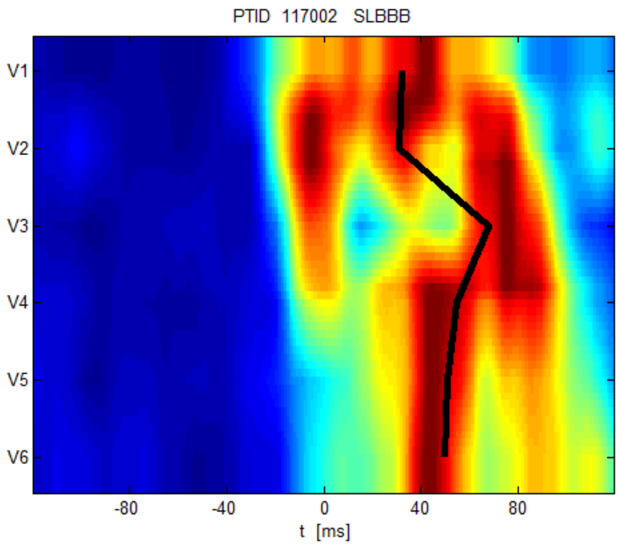
PTID 117002 SLBBB
V1
V2
V3
V4
V5
V6
-80
-40
0
40
80
t [ms]

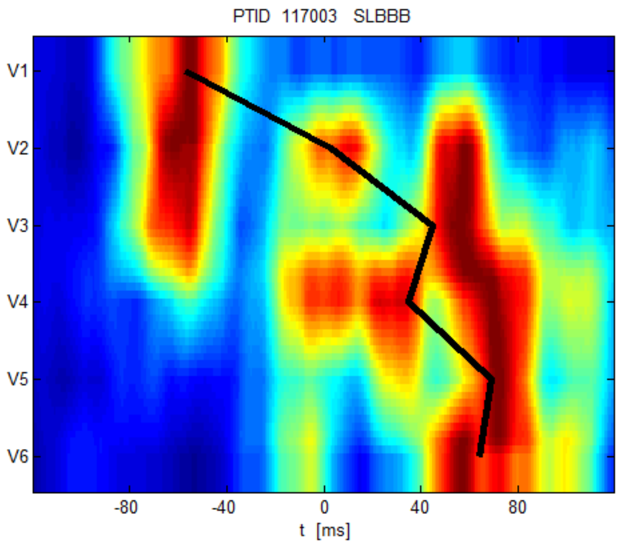
PTID 117003 SLBBB
V1
V2
V3
V4
V5
V6
-80
-40
0
40
80
t [ms]

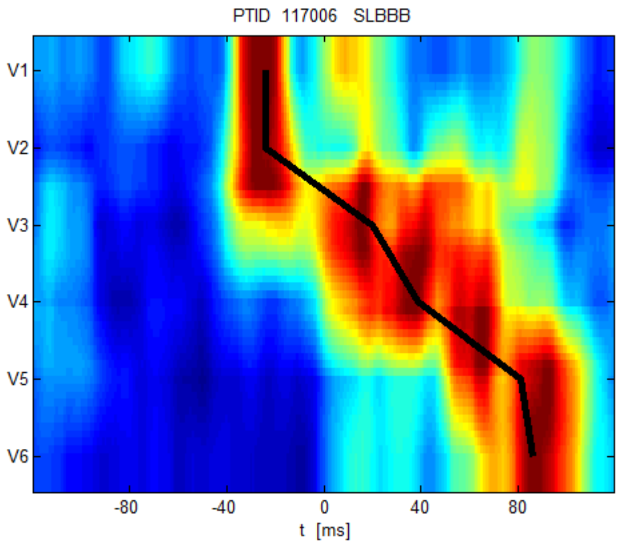
PTID 117006 SLBBB
V1
V2
V3
V4
V5
V6
-80
-40
0
40
80
t [ms]

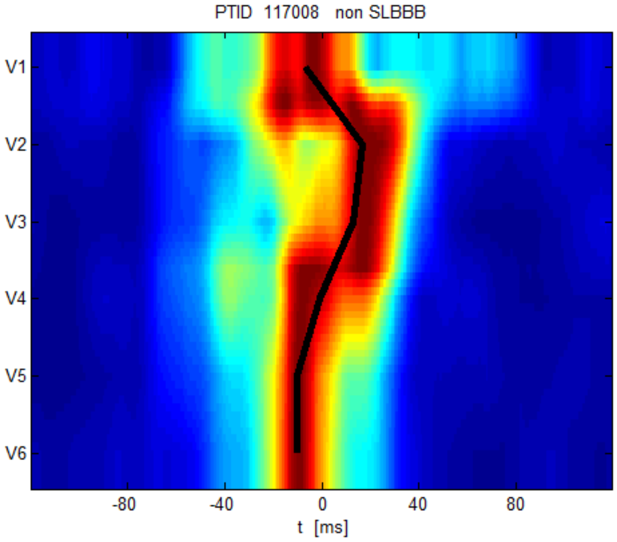
PTID 117008 non SLBBB
V1
V2
V3
V4
V5
V6
-80
-40
0
40
80
t [ms]

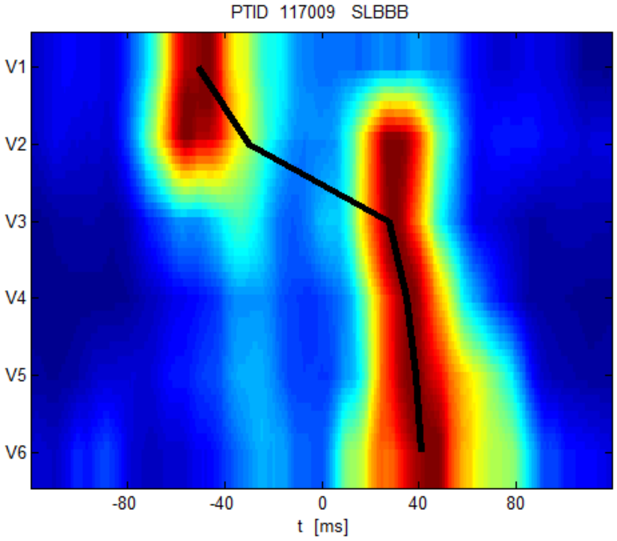
PTID 117009 SLBBB
V1
V2
V3
V4
V5
V6
-80
-40
0
40
80
t [ms]

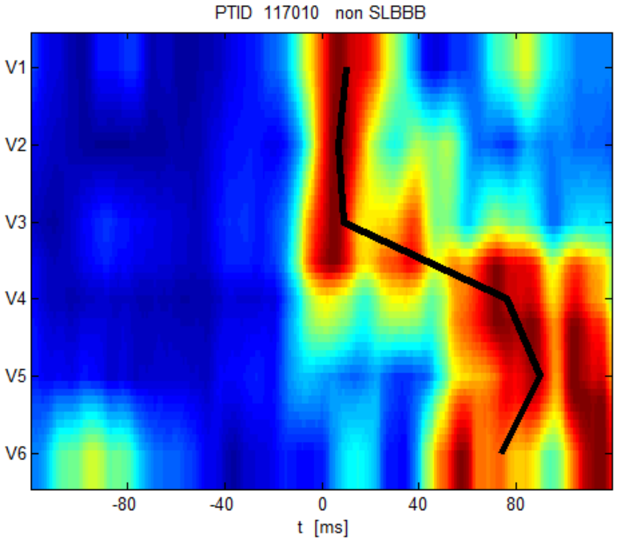
PTID 117010 non SLBBB
V1
V2
V3
V4
V5
V6
-80
-40
0
40
80
t [ms]

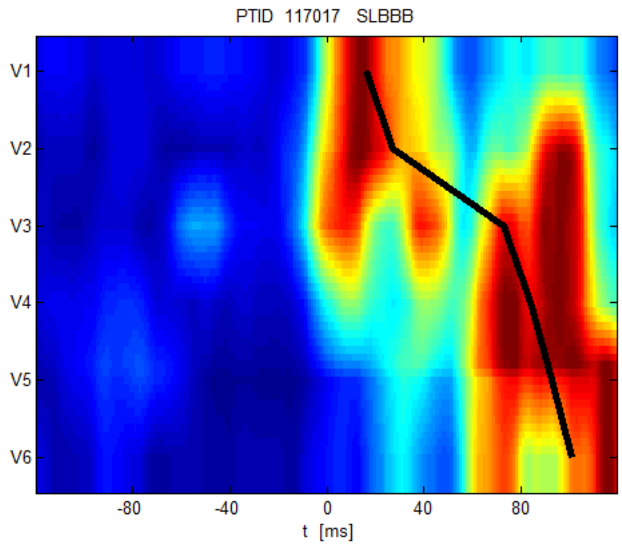
PTID 117017 SLBBB
V1
V2
V3
V4
V5
V6
-80
-40
0
40
80
t [ms]

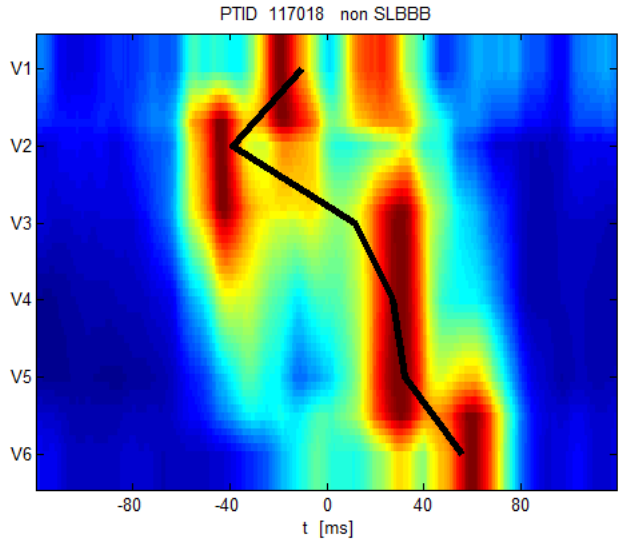
PTID 117018 non SLBBB
V1
V2
V3
V4
V5
V6
-80
-40
0
40
80
t [ms]

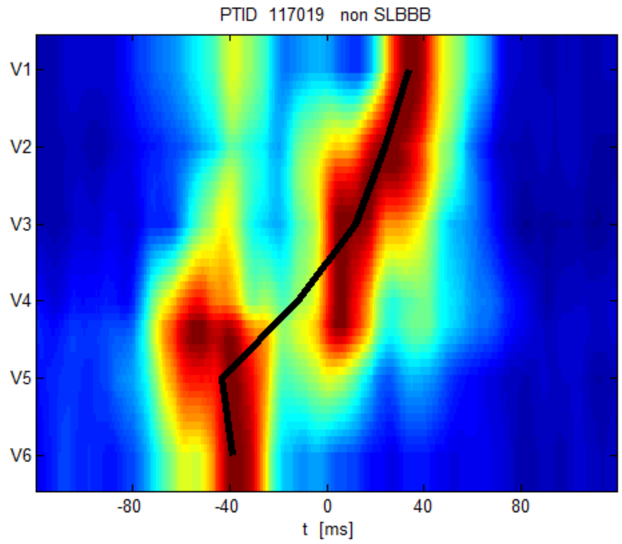
PTID 117019 non SLBBB
V1
V2
V3
V4
V5
V6
-80
-40
0
40
80
t [ms]

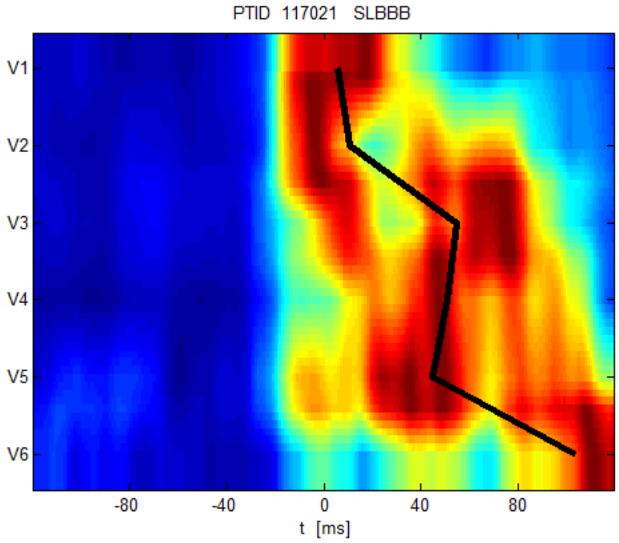
PTID 117021 SLBBB
V1
V2
V3
V4
V5
V6
-80
-40
0
40
80
t [ms]

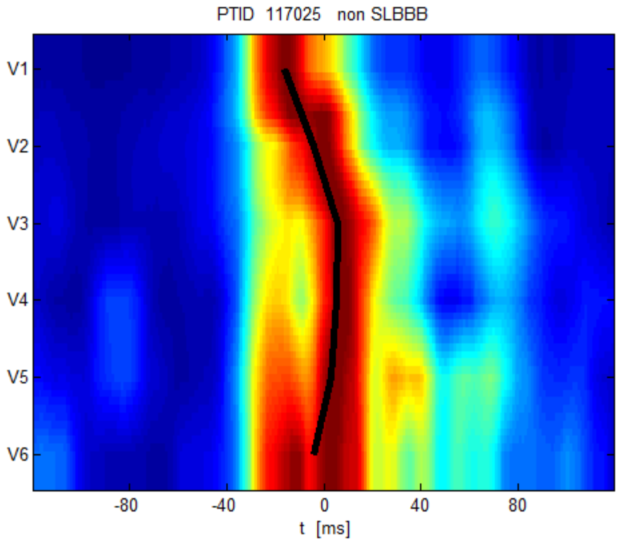
PTID 117025 non SLBBB
V1
V2
V3
V4
V5
V6
-80
-40
0
40
80
t [ms]

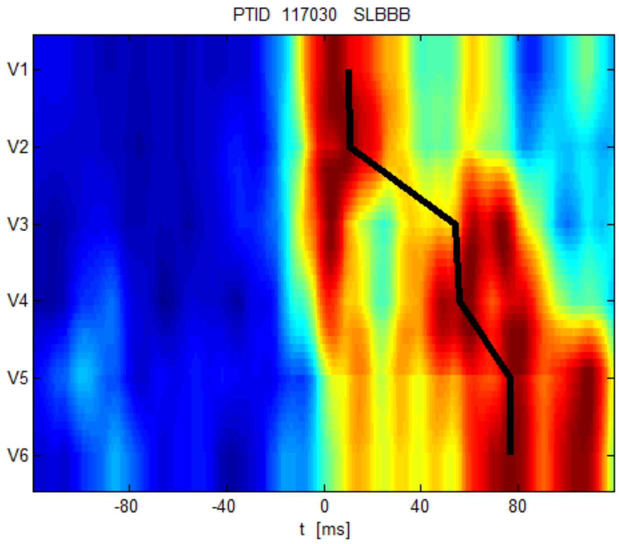
PTID 117030 SLBBB
V1
V2
V3
V4
V5
V6
-80
-40
0
40
80
t [ms]

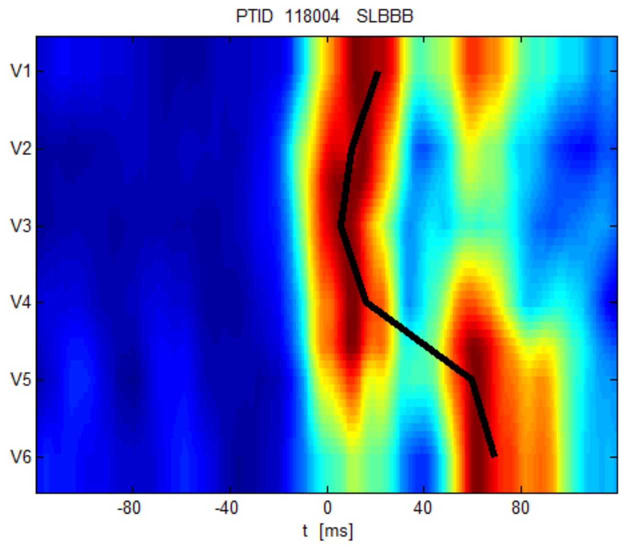
PTID 118004 SLBBB
V1
V2
V3
V4
V5
V6
-80
-40
0
40
80
t [ms]

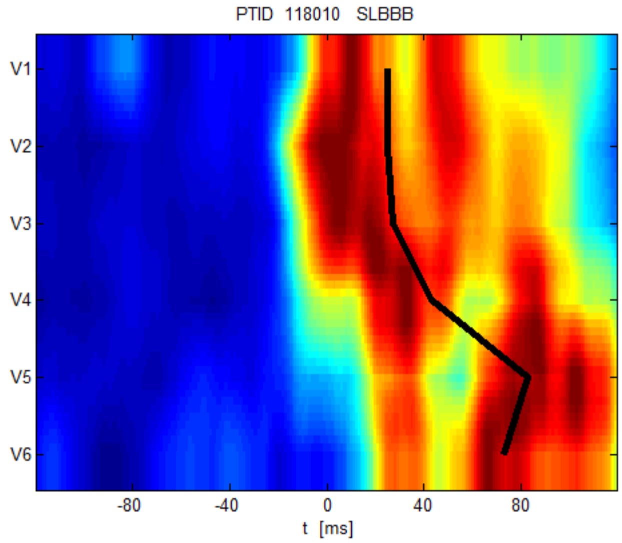
PTID 118010 SLBBB
V1
V2
V3
V4
V5
V6
-80
-40
0
40
80
t [ms]

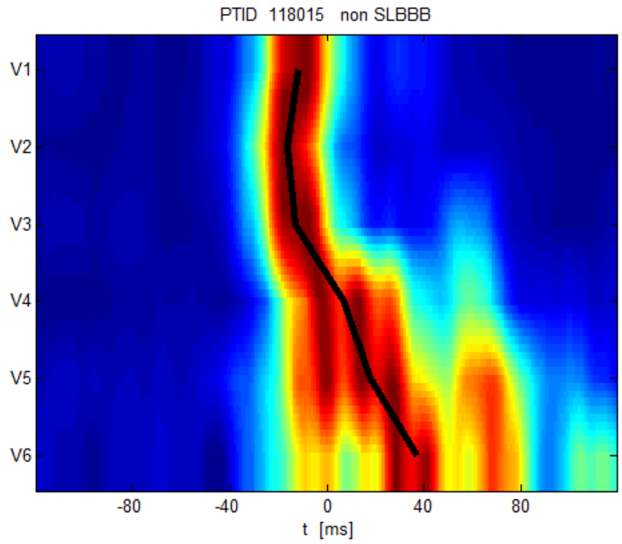
PTID 118015 non SLBBB
V1
V2
V3
V4
V5
V6
-80
-40
0
40
80
t [ms]

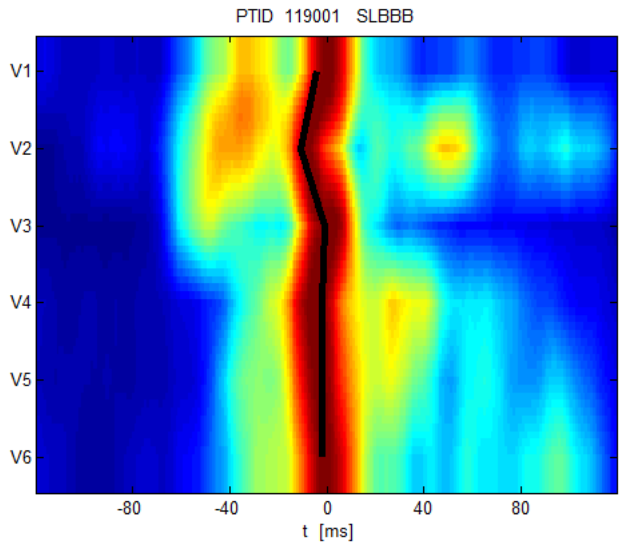
PTID 119001 SLBBB
V1
V2
V3
V4
V5
V6
-80
-40
0
40
80
t [ms]

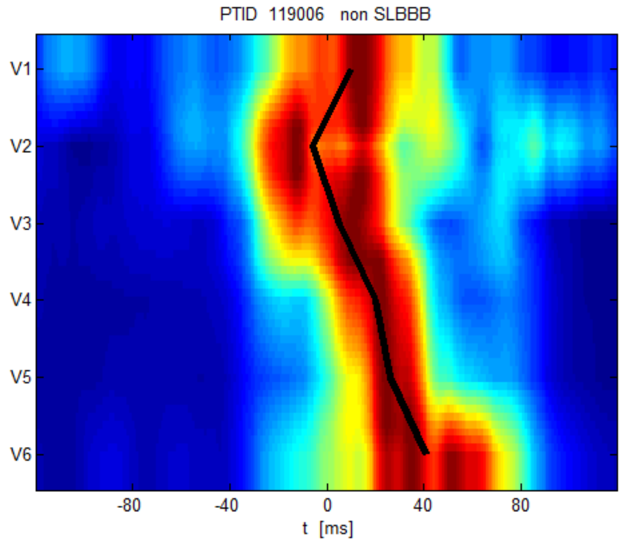
PTID 119006 non SLBBB
V1
V2
V3
V4
V5
V6
-80
-40
0
40
80
t [ms]

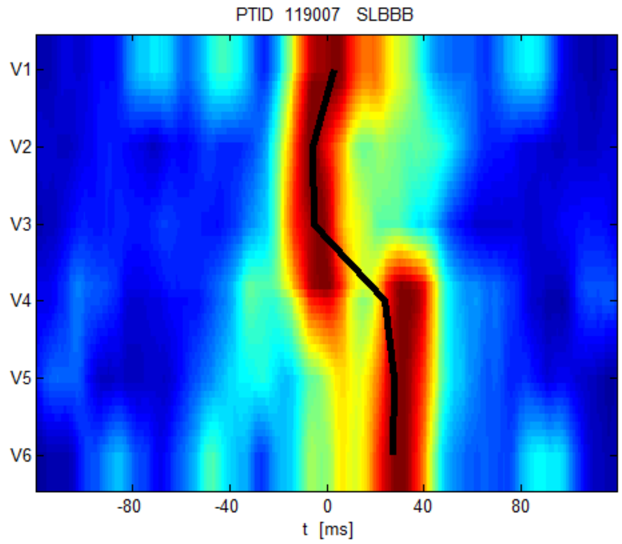
PTID 119007 SLBBB
V1
V2
V3
V4
V5
V6
-80
-40
0
40
80
t [ms]

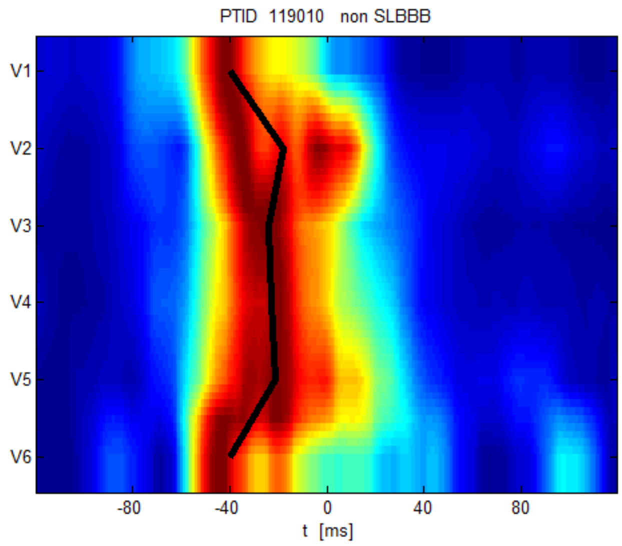
PTID 119010 non SLBBB
V1
V2
V3
V4
V5
V6
-80
-40
0
40
80
t [ms]

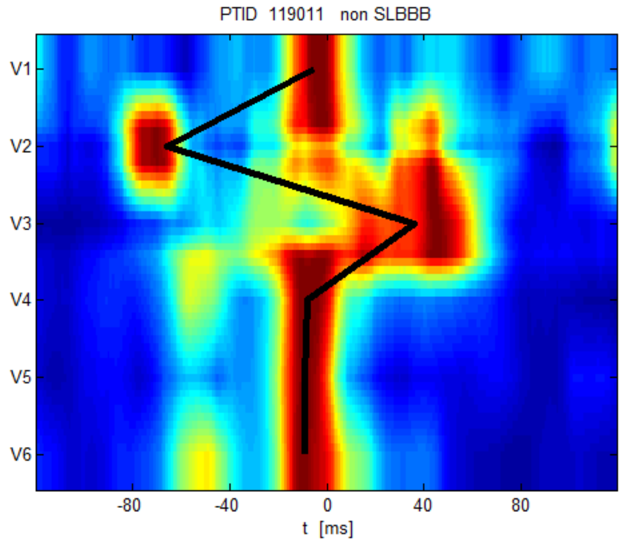
PTID 119011 non SLBBB
V1
V2
V3
V4
V5
V6
-80
-40
0
40
80
t [ms]

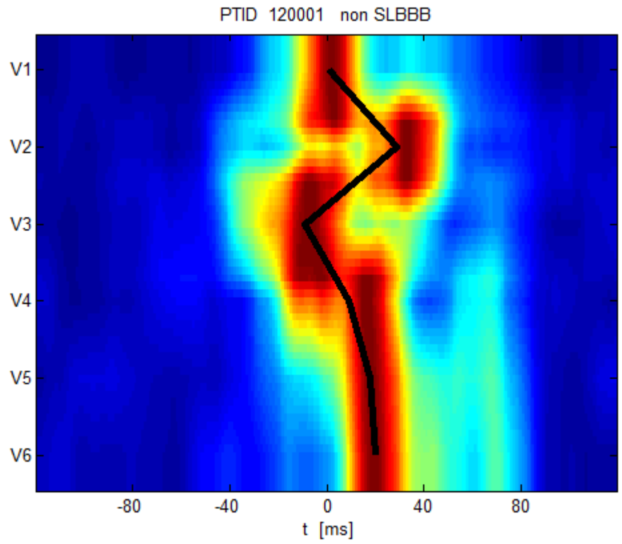
PTID 120001 non SLBBB
V1
V2
V3
V4
V5
V6
-80
-40
0
40
80
t [ms]

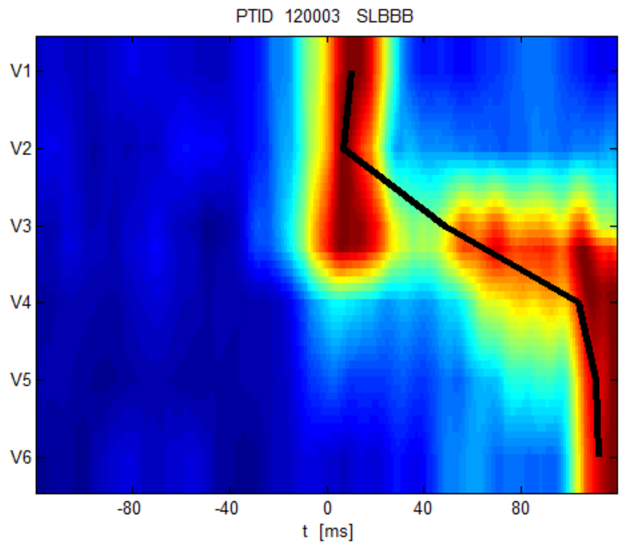
PTID 120003 SLBBB
V1
V2
V3
V4
V5
V6
-80
-40
0
40
80
t [ms]

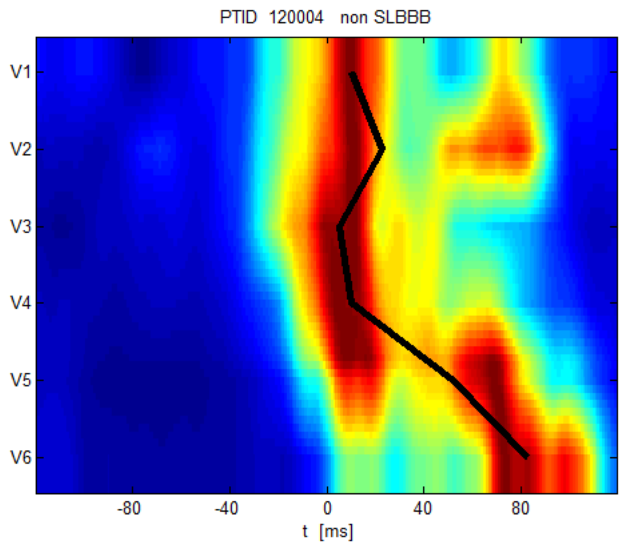
PTID 120004 non SLBBB
V1
V2
V3
V4
V5
V6
-80
-40
0
40
80
t [ms]

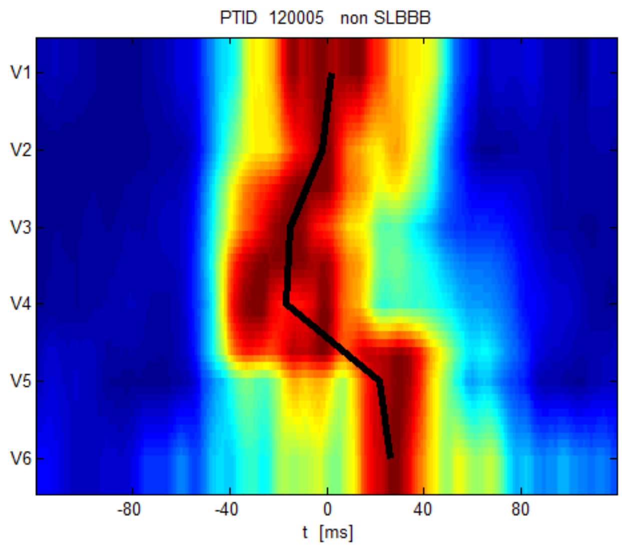
PTID 120005 non SLBBB
V1
V2
V3
V4
V5
V6
-80
-40
0
40
80
t [ms]

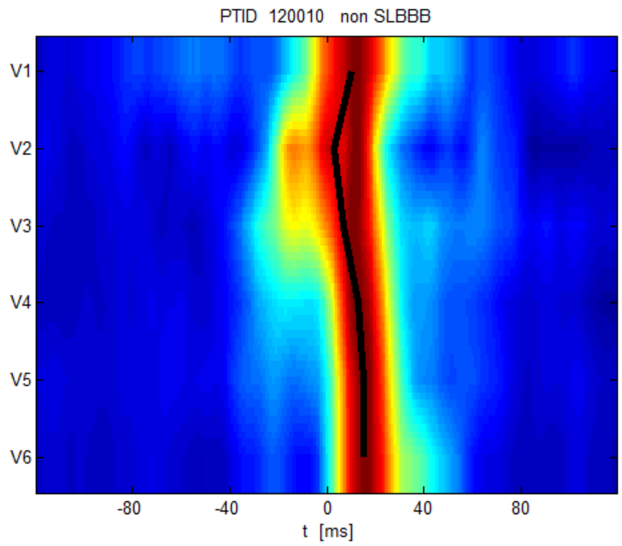
PTID 120010 non SLBBB
V1
V2
V3
V4
V5
V6
-80
-40
0
40
80
t [ms]

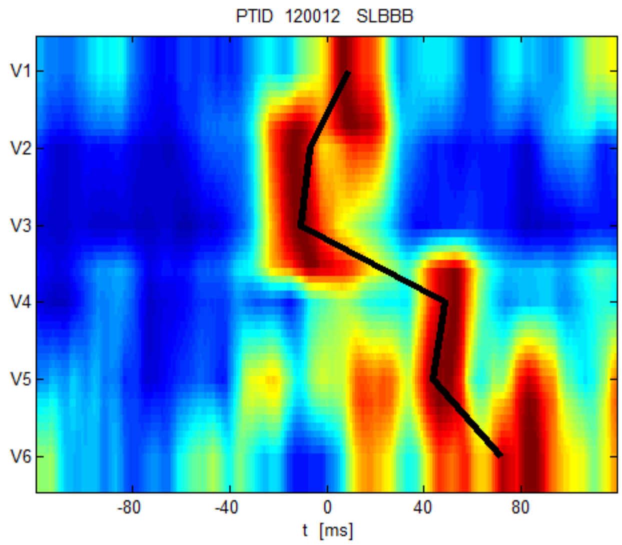
PTID 120012 SLBBB
V1
V2
V3
V4
V5
V6
-80
-40
0
40
80
t [ms]

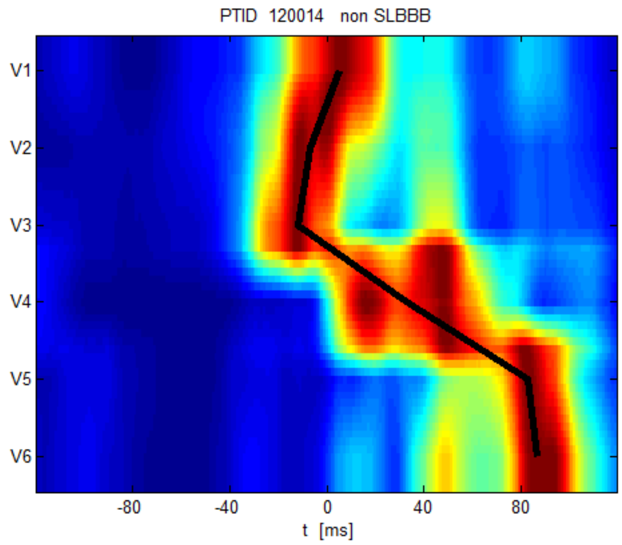
PTID 120014 non SLBBB
V1
V2
V3
V4
V5
V6
-80
-40
0
40
80
t [ms]

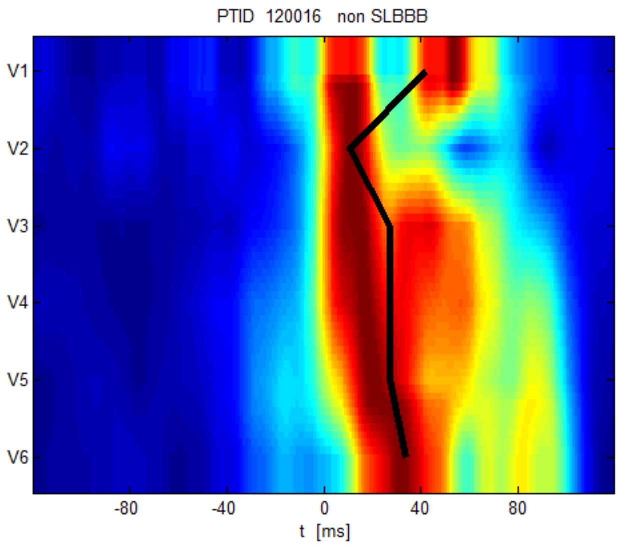
PTID 120016 non SLBBB
V1
V2
V3
V4
V5
V6
-80
-40
0
40
80
t [ms]

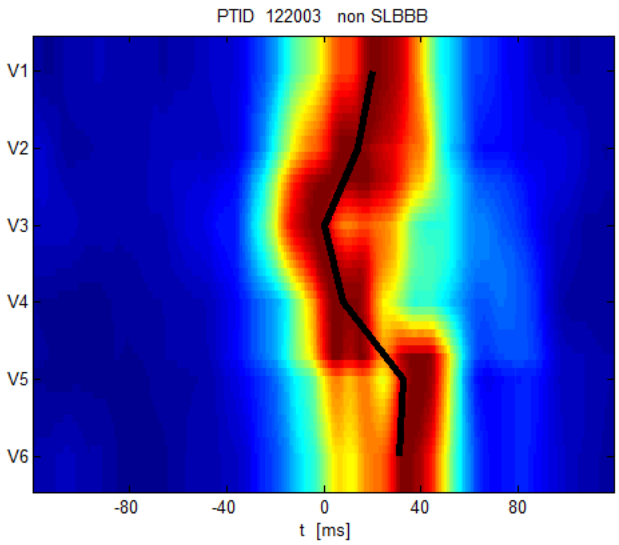
PTID 122003 non SLBBB
V1
V2
V3
V4
V5
V6
-80
-40
0
40
80
t [ms]

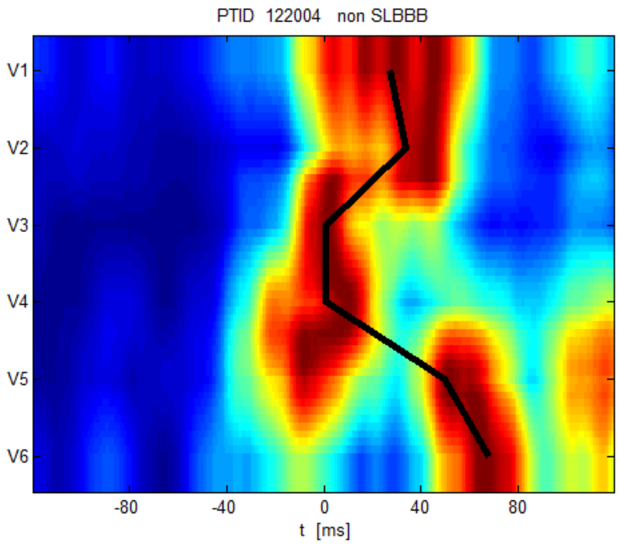
PTID 122004 non SLBBB
V1
V2
V3
V4
V5
V6
-80
-40
0
40
80
t [ms]

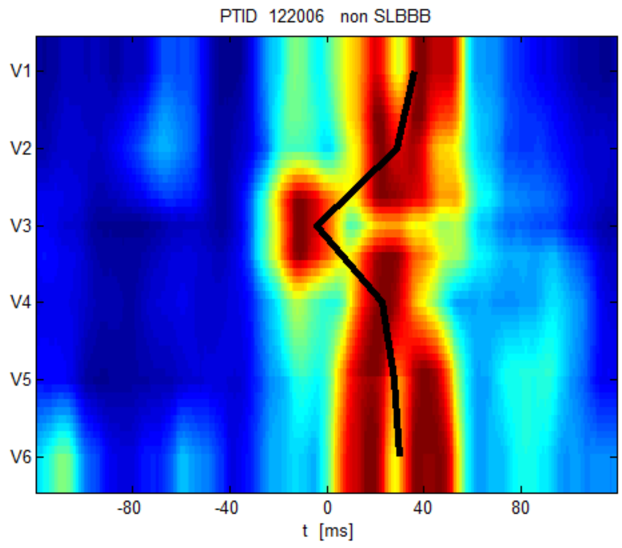
PTID 122006 non SLBBB
V1
V2
V3
V4
V5
V6
-80
-40
0
40
80
t [ms]

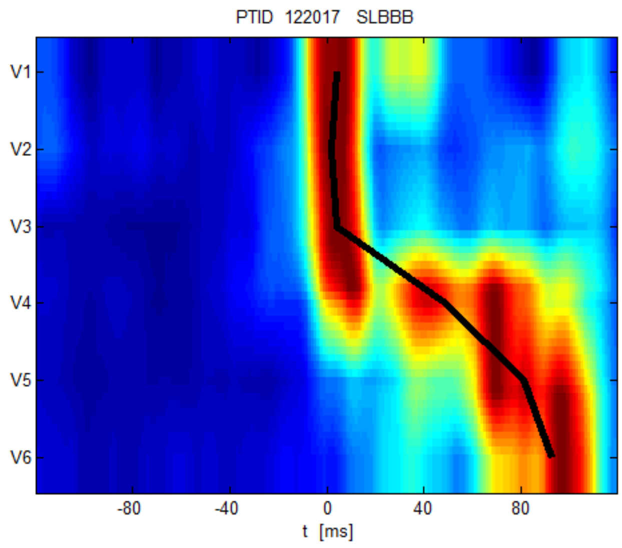
PTID 122017 SLBBB
V1
V2
V3
V4
V5
V6
-80
-40
0
40
80
t [ms]

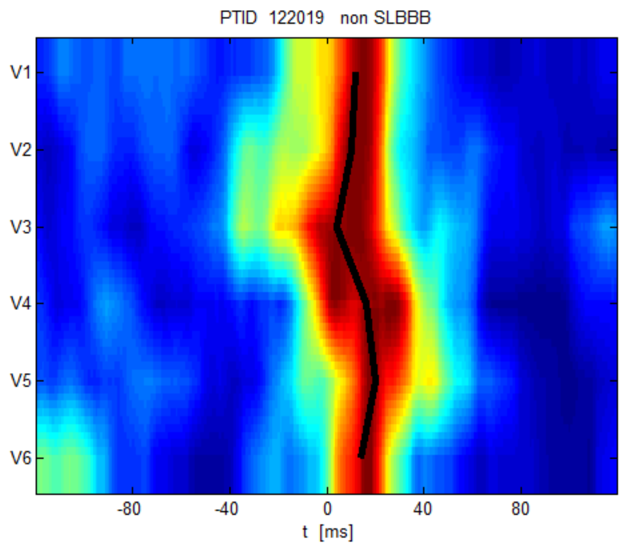
PTID 122019 non SLBBB
V1
V2
V3
V4
V5
V6
-80
-40
0
40
80
t [ms]

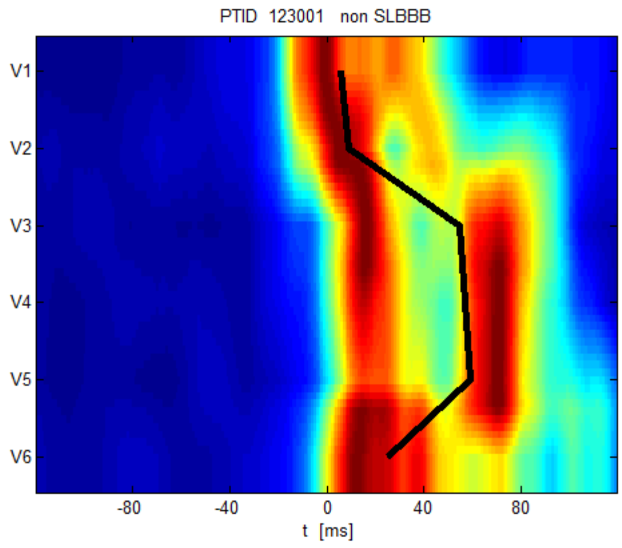
PTID 123001 non SLBBB
V1
V2
V3
V4
V5
V6
-80
-40
0
40
80
t [ms]

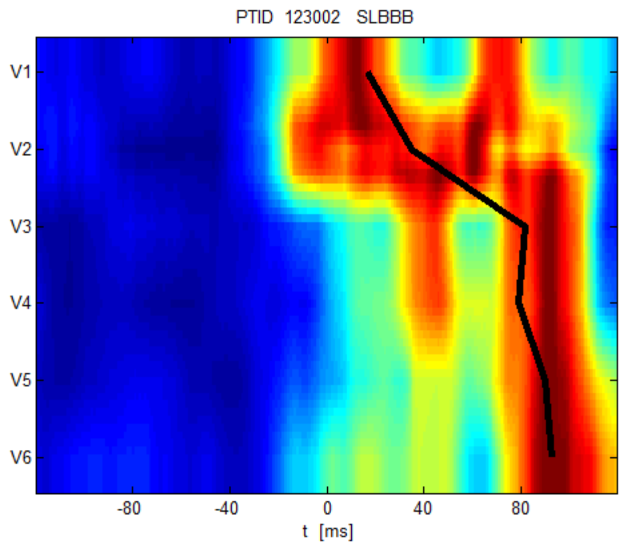
PTID 123002 SLBBB
V1
V2
V3
V4
V5
V6
-80
-40
0
40
80
t [ms]

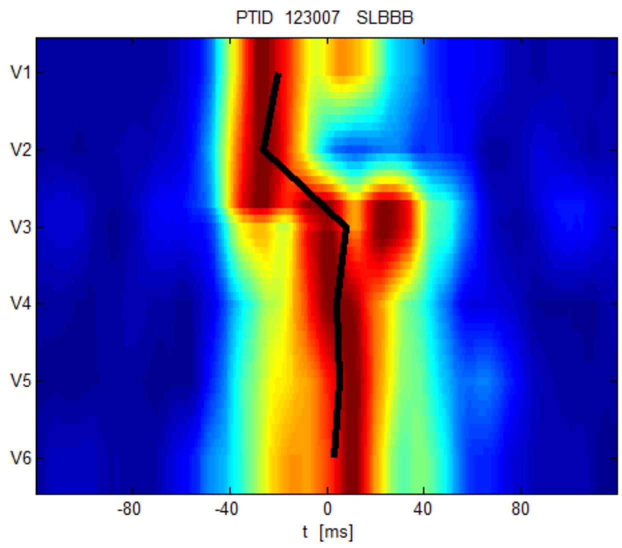
PTID 123007 SLBBB
V1
V2
V3
V4
V5
V6
-80
-40
0
40
80
t [ms]

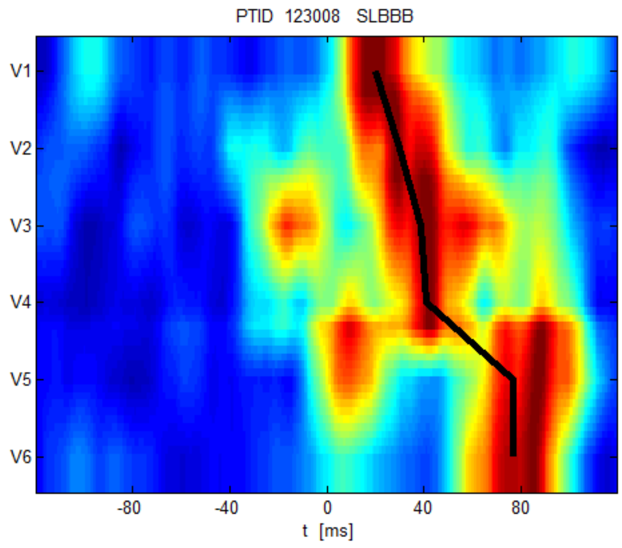
PTID 123008 SLBBB
V1
V2
V3
V4
V5
V6
-80
-40
0
40
80
t [ms]

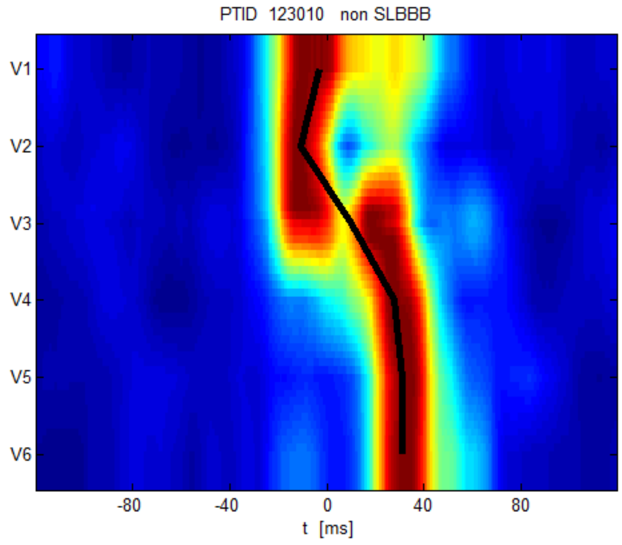
PTID 123010 non SLBBB
V1
V2
V3
V4
V5
V6
-80
-40
0
40
80
t [ms]

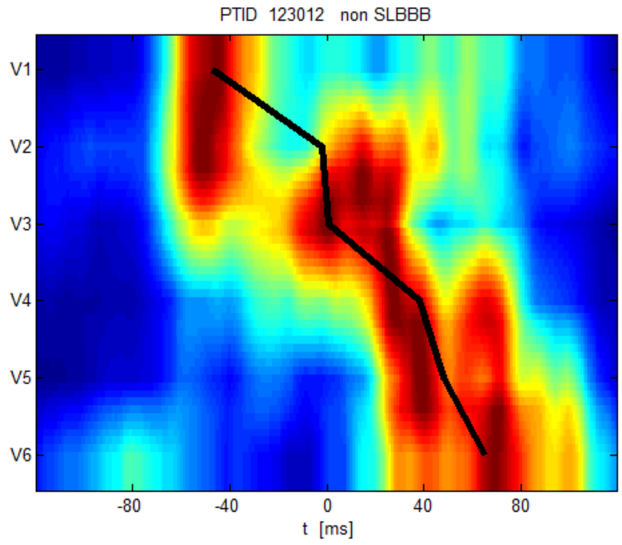
PTID 123012 non SLBBB
V1
V2
V3
V4
V5
V6
-80
-40
0
40
80
t [ms]

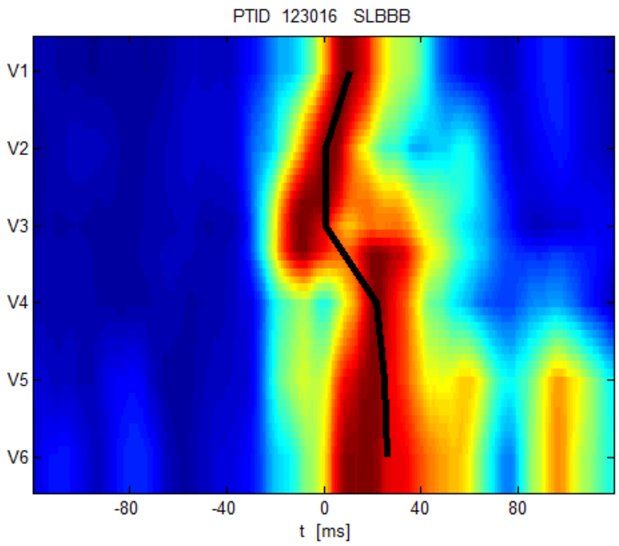
PTID 123016 SLBBB
V1
V2
V3
V4
V5
V6
-80
-40
0
40
80
t [ms]

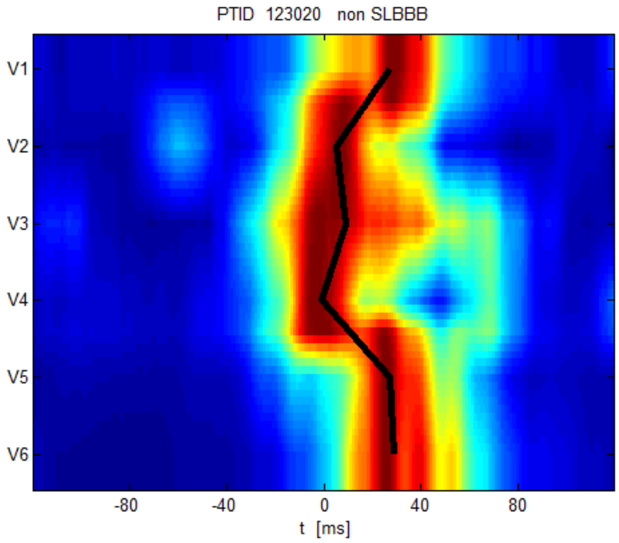
PTID 123020 non SLBBB
V1
V2
V3
V4
V5
V6
-80
-40
0
40
80
t [ms]

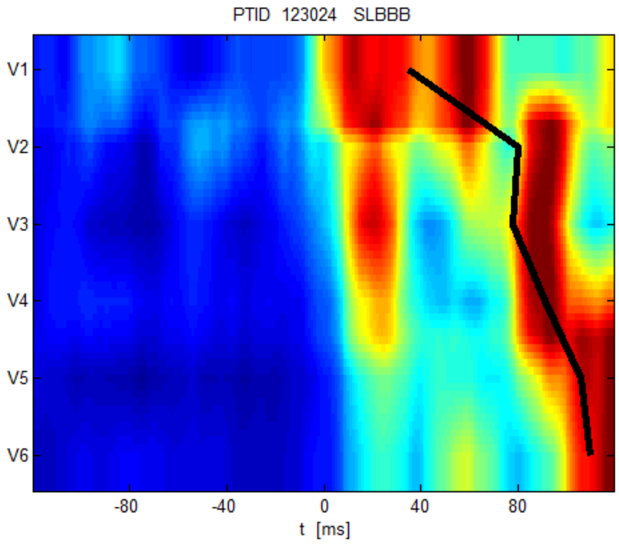
PTID 123024 SLBBB
V1
V2
V3
V4
V5
V6
-80
-40
0
40
80
t [ms]

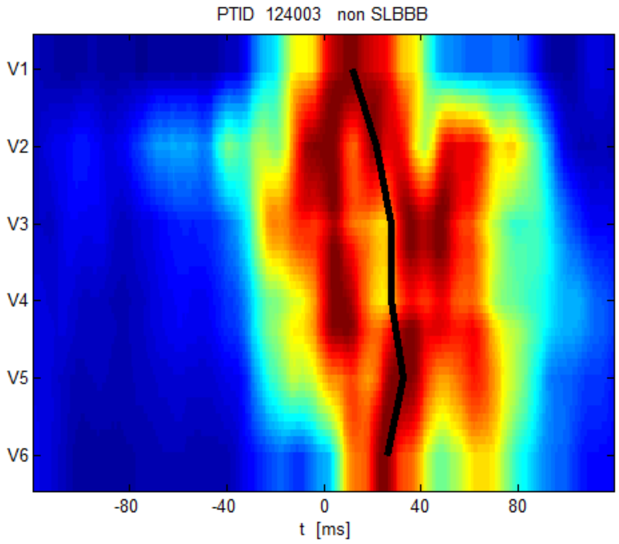
PTID 124003 non SLBBB
V1
V2
V3
V4
V5
V6
-80
-40
0
40
80
t [ms]

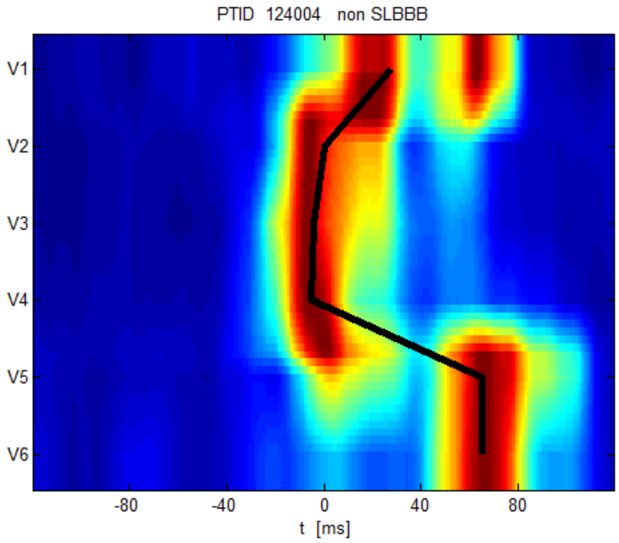
PTID 124004 non SLBBB
V1
V2
V3
V4
V5
V6
-80
-40
0
40
80
t [ms]

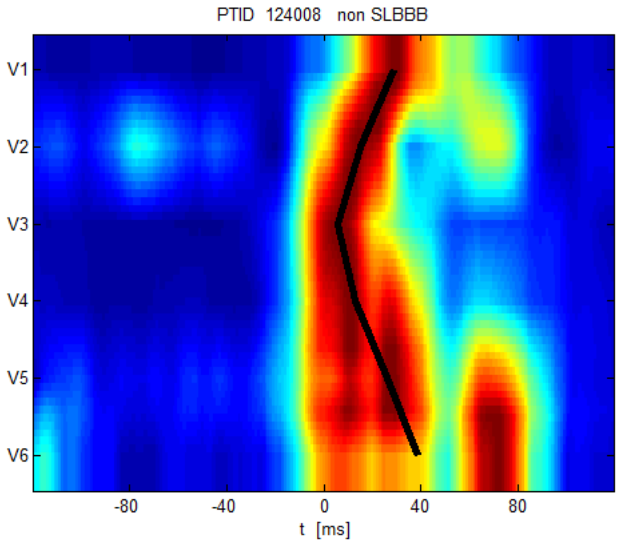
PTID 124008 non SLBBB
V1
V2
V3
V4
V5
V6
-80
-40
0
40
80
t [ms]

PTID 125008 SLBBB

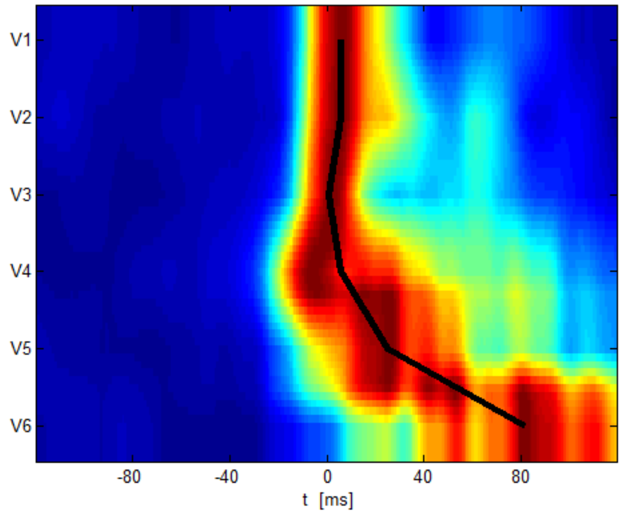

PTID 125010 non SLBBB

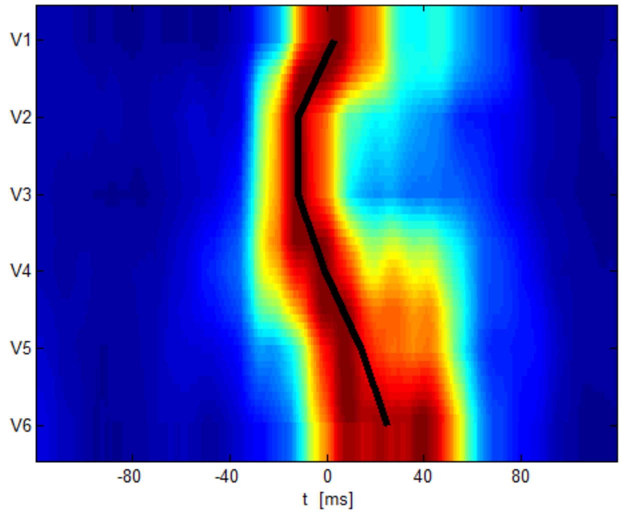

PTID 125011 non SLBBB

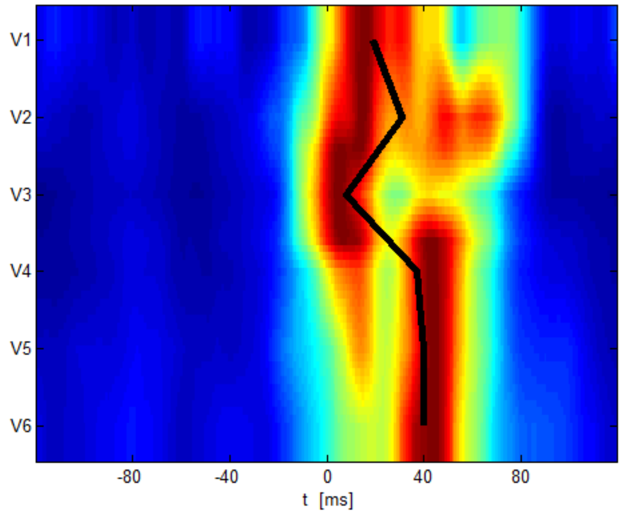

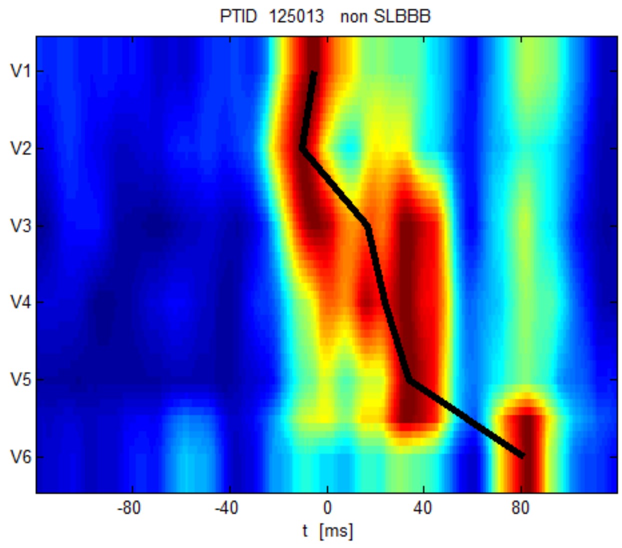
PTID 125013 non SLBBB
V1
V2
V3
V4
V5
V6
-80
-40
0
40
80
t [ms]

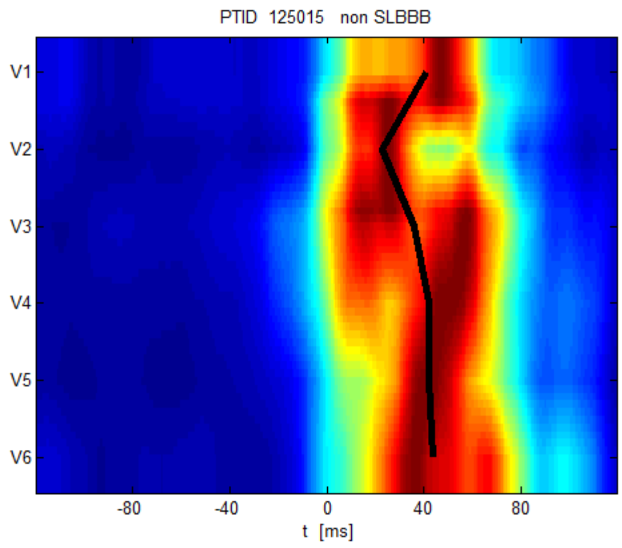
PTID 125015 non SLBBB
V1
V2
V3
V4
V5
V6
-80
-40
0
40
80
t [ms]

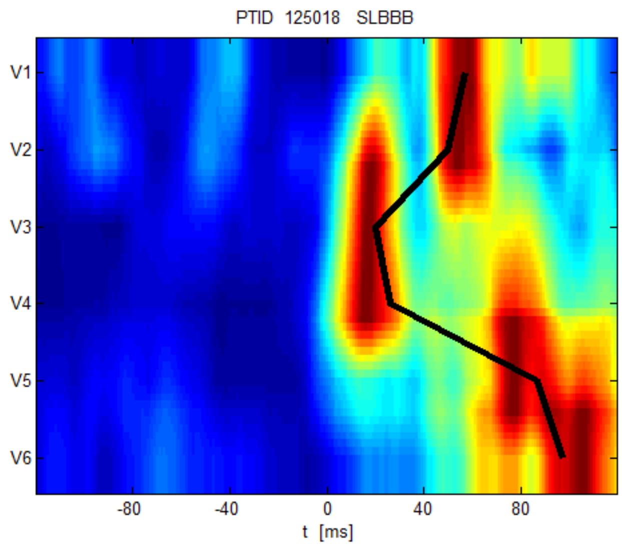
PTID 125018 SLBBB
V1
V2
V3
V4
V5
V6
-80
-40
0
40
80
t [ms]

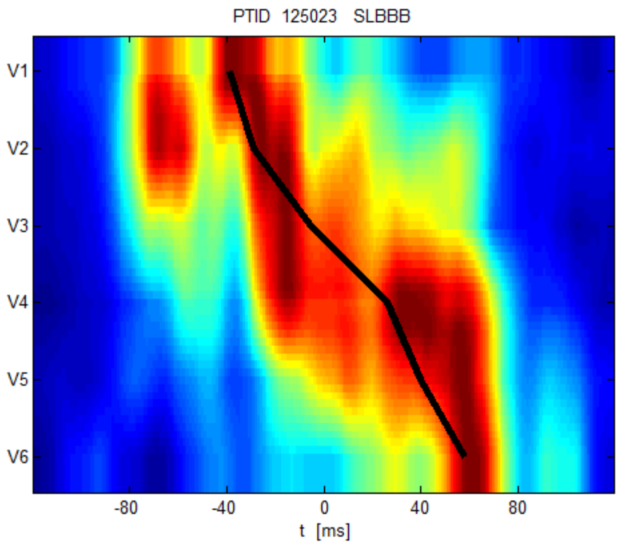
PTID 125023 SLBBB
V1
V2
V3
V4
V5
V6
-80
-40
0
40
80
t [ms]

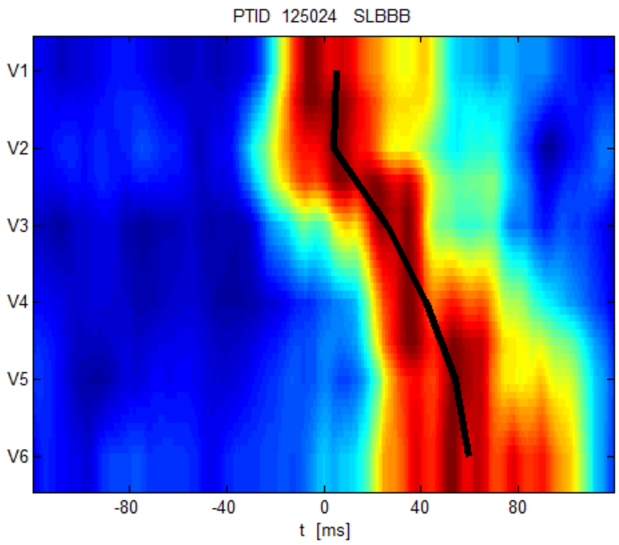
PTID 125024 SLBBB
V1
V2
V3
V4
V5
V6
-80
-40
0
40
80
t [ms]
